# Homoleptic Bismuth Alkynes: Isolable Reagents for Selective Alkynyl Radical Transfer

**DOI:** 10.1002/anie.202519525

**Published:** 2025-11-23

**Authors:** Gargi Kundu, Felix Debbeler, Sascha Reith, Frerik Wurm, Alicia Casitas, Crispin Lichtenberg

**Affiliations:** ^1^ Fachbereich Chemie Philipps‐Universität Marburg Hans‐Meerwein‐Str. 4 35032 Marburg Germany; ^2^ mar.quest | Marburg Center for Quantum Materials and Sustainable Technologies 35032 Marburg Germany

**Keywords:** Alkynyl radical, Bismuth alkynes, C–C coupling, Radical borylation, Thermal activation

## Abstract

Alkynyl radicals represent highly attractive building blocks in synthetic chemistry but keep posing considerable challenges to researchers in the field. While their existence in interstellar space, in combustion processes, and under matrix‐isolation conditions has been documented, access under typical wet‐chemical conditions remains limited. In this context, bismuth compounds are promising candidates as their ability to engage in controlled radical reactions has recently been brought into the focus of research efforts. Here, we present the first synthesis and full characterization of a series of homoleptic bismuth alkyne compounds, Bi(C≡CR)_3_ (R = alkyl, silyl, aryl). The isolable compounds readily and selectively release alkynyl radicals, as demonstrated by EPR spectroscopic studies. This reactivity has been harnessed in Glaser‐type C–C homocoupling reactions, as well as in C–C, C–B, C–Se, and C–Te bond‐forming events with external substrates, including transformations that commonly proceed through polar reaction pathways. The bismuth compounds complement existing strategies for the wet‐chemical generation of alkynyl radicals in that they are transition‐metal‐free, avoid toxic components, proceed under mild conditions, can be operated via thermal activation, and do not require any additives or co‐reagents.

## Graphical abstract

The first series of fully characterized homoleptic alkynyl bismuth compounds Bi(C≡CR)_3_ is presented. Facile homolytic Bi–C bond cleavage grants access to alkynyl radicals, which have been employed in selective C–X bond formation (X = C, B, Se, Te). This complements existing strategies for alkynyl radical generation as it proceeds under mild and purely thermal conditions, without co‐reagents or additives, and in the absence of transition metals.

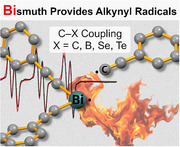

## Introduction

Alkynyl groups represent highly attractive substituents in synthetic chemistry due to their hydrophobicity, low steric profile, and rigid structural properties.^[^ ^1^, ^2^ ^]^ As an important asset, they show a reactivity on the border between a spectator moiety and a reactive site. This makes them inert enough to be incorporated in complex structural motifs of natural products and pharmaceuticals,^[^ ^3^, ^4^, ^5^ ^]^ but reactive enough to serve as valuable building blocks in organic synthesis, materials science, and bio‐imaging.^[^ ^6^, ^7^, ^8^, ^9^, ^10^, ^11^ ^]^ While the introduction of alkynyl groups has long been focused on polar reaction pathways exploiting reactions between nucleophiles and electrophiles, recent endeavors have revealed the potential of radical reactions in this field.^[^ ^5^, ^10^ ^]^ The majority of these radical approaches relies on photochemical reaction conditions and/or the use of transition metals (including the lanthanoids) in order to enable the desired reactivity. Importantly, many of these examples do not aim at the challenging generation of alkynyl radicals,^[^ ^5^, ^10^ ^]^ but rather provide alkynyl species as targets of radical attacks.

Copper acetylides have been suggested early‐on to act as sources of alkynyl radicals^[^ ^12^ ^]^ in the well‐established Glaser coupling.^[^ ^13^, ^14^ ^]^ Only recently, the release of alkynyl radicals from copper acetylides has been demonstrated. Notably, however, photochemical conditions were required to achieve this goal.^[^ ^15^ ^]^ Generally, methods for the generation of alkynyl radicals that exploit thermal modes of activation are rare.^[^ ^10^ ^]^ These intriguing approaches tend to rely on harsh reaction conditions (e.g., temperatures of 120–140 °C), the presence of superstoichiometric amounts of an external base or a redox reagent, and the use of bromo‐ or iodoalkynes.^[^ ^10^, ^16^, ^17^ ^]^ The radical nature of the reactions (albeit plausible) has not directly been demonstrated.^[^ ^10^, ^16^, ^17^ ^]^ Thus, there is a lack of simple, storable, and readily available reagents that act as reliable sources of thermally generated alkynyl radicals under mild conditions and in the absence of co‐reagents.

Recent advances in bismuth chemistry have underscored the remarkable potential of this heavy and nonradioactive p‐block element to engage in controlled radical reactions, paving the way for innovative strategies that offer alternatives to the traditional approaches in radical organic synthesis and small‐molecule activation.^[^ ^18^, ^19^, ^20^, ^21^, ^22^, ^23^, ^24^, ^25^, ^26^, ^27^, ^28^, ^29^, ^30^, ^31^, ^32^, ^33^, ^34^, ^35^, ^36^, ^37^, ^38^, ^39^, ^40^ ^]^ In view of the tendency of bismuth compounds to engage in homolytic bond cleavage reactions, we became interested in bismuth alkynyl compounds. A small number of heteroleptic Bi(III) compounds with one alkynyl substituent has been reported, but their reactivity with respect to R_2_Bi–CCR’ bond cleavage is poorly explored, and no radical transformations have been noted in this context to date.^[^ ^41^, ^42^, ^43^, ^44^, ^45^, ^46^, ^47^, ^48^, ^49^, ^50^ ^]^ While the synthesis and properties of molecular additive‐free complexes BiR_3_ (R = alkyl, aryl, allyl, cyclopentadienyl) are well‐studied, the structure and bonding of homoleptic Bi(C≡CR)_3_ compounds have not been uncovered so far. As a matter of fact, Lahcini's group in 2024 demonstrated the use of tri(phenylethynyl)bismuth as a pre‐catalyst for the stereoselective ring‐opening polymerization of *rac*‐lactide via (suggested) alcoholysis; however, a yield and a structural characterization of tri(phenylethynyl)bismuth or investigations of its properties were not provided.^[^ ^51^ ^]^

Here, we report the synthesis, isolation, and full characterization of the first series of homoleptic bismuth alkynyl compounds, Bi(C≡CR)_3_. Investigations into their reactivity reveal a portfolio of peerless additive‐free alkynyl radical transfer reactions by main group compounds and can be triggered by thermal or photochemical activation.

## Results and Discussion

### Synthesis and Characterization of Bismuth Alkynes

**Scheme 1:**
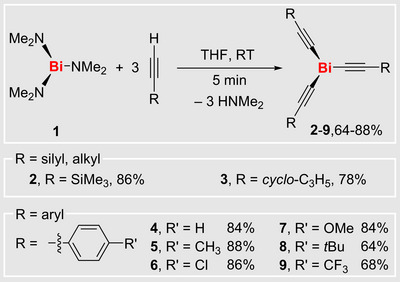
Synthesis of bismuth alkynes **2**–**9**.

The addition of three equivalents of an alkyne to a THF solution of the bismuth amide Bi(NMe_2_)_3_ (**1**)^[^ ^52^ ^]^ at room temperature yields the corresponding bismuth alkynes Bi(C≡CR)_3_ in straightforward reactions with isolated yields of 64%–88% (Scheme 1). The methodology was applied to eight bismuth alkynes, including a silyl‐substituted (R = SiMe_3_ (**2**)), an aliphatic (*cyclo*‐C_3_H_6_) (**3**)), and six aromatic derivatives (R = 4‐R’‐C_6_H_4_; R’ = H (**4**), Me (**5**), Cl (**6**), OMe (**7**), *t*Bu (**8**), CF_3_ (**9**)). Importantly, this synthetic approach avoids a chromatographic work‐up and readily facilitates the isolation of target compounds such as Bi(C≡CPh)_3_, which require polar media like THF, acetonitrile or pyridine to be sufficiently solubilized and tend to be contaminated with salt by‐products when prepared from BiCl_3_ and alkynyl lithium reagents in common salt elimination approaches.^[^ ^51^ ^]^

Compounds **2**–**9** were isolated as colorless powders and analyzed by NMR spectroscopy in solution, revealing the expected signal patterns. As a general feature in the ^13^C NMR spectra, the bismuth‐bound carbon atoms resonate in the relatively broad, but still characteristic range of 87.5–122.9 ppm as broad singlets (fwhm ≈ 59–97 Hz) due to quadrupolar broadening by the ^209^Bi nucleus with a nuclear spin of *I* = 9/2. In comparison to the free alkynes, a low‐field shift in the range of 24–39 ppm is observed, depending on the specific compound (Table S1).

**Figure 1:**
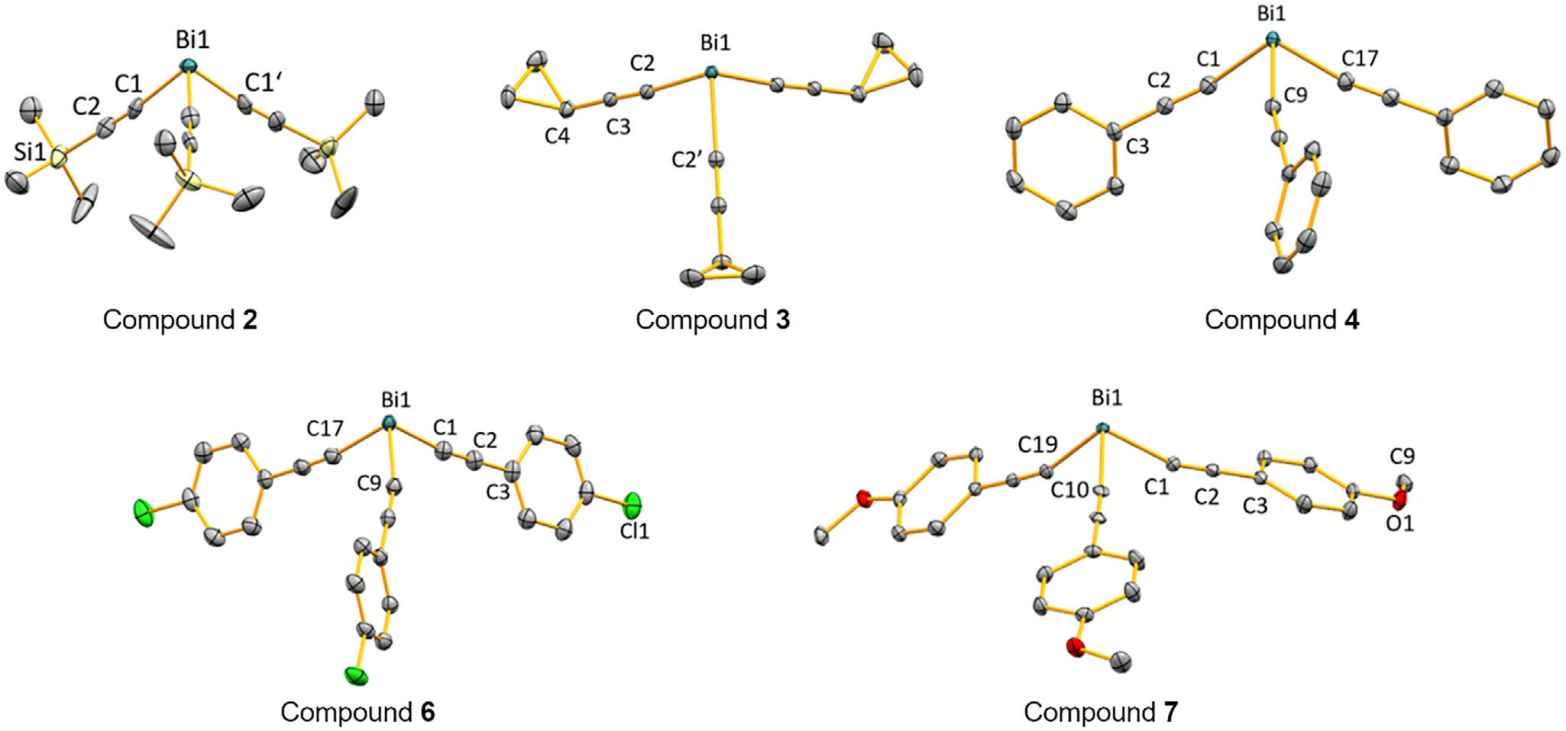
Molecular structures of **2**, **3**, **4**, **6**, and **7** in the solid state. Displacement ellipsoids are shown at the 50% probability level. THF molecules that show weak contacts (according to distance criteria) to the bismuth atoms of **4** and **6** and hydrogen atoms are omitted for clarity. Selected bond lengths [Å] and angles [°] are given as follows. Compound **2**: Bi1–C1 2.206(13), C1–C2 1.208(16), C2–Si1 1.864(13); C1–Bi1–C1’ 90.2(5), Bi1–C1–C2 174.5(11), C1–C2–Si1 178.1(13). Compound **3**: Bi1–C2 2.216(4), C2–C3 1.204(5), C3–C4 1.442(5); C2–Bi1–C2’ 91.29(13), C3–C2–Bi1 175.5(3), C3–C4–C5 120.0(3). Compound **4**: Bi1–C1 2.2208(19), C1–C2 1.206(3), C2–C3 1.438(3); C1–Bi1–C9 91.46(7), C2–C1–Bi1 169.40(17), C3–C2–C1 177.4(2). Compound **6**: Bi1–C1 2.211(3), C1–C2 1.203(4), C2–C3 1.450(4); C1–Bi1–C9 93.28(12), C2–C1–Bi1 166.9(3), C3‐C2‐C1 179.6(3). Compound **7**: Bi1–C1 2.242(4), Bi1–C19 2.244(4), Bi1–C10 2.199(4), C1–C2 1.202(6), C2–C3 1.442(6); C2–C1–Bi1 163.8(3), C2–C1–Bi1 166.9(3), C20–C19–Bi1 161.7(3), C1–Bi1–C19 91.80(14), C1–Bi1–C10 92.86(14), C10–Bi1–C19 93.15(14).

Compounds **2**, **3**, **4**, **6**, and **7** were investigated by single‐crystal X‐ray diffraction analyses (Table 1, Figure 1, and Supporting Information). All compounds show a trigonal pyramidal coordination geometry with angle sums around the bismuth center ranging from 270.7°–277.8°, translating into C–Bi–C angles close to 90° (specifically: 88.8–94.8°). The Bi–C≡C angles range from 164.6°–175.5°, i.e., they differ — in some cases considerably — from the ideally expected value of 180°.

**Table 1:** Bond lengths and angles of the bismuth alkynes (**2**, **3**, **4**, **6,** and **7**) as determined from single‐crystal X‐ray analyses.

Bi(C≡CR)_3_	C–Bi–C angle [°]	Bi–C–C angle [°]	Bi–C bond length [Å]
**2**: R = TMS	90.2(4)	174.4(11)	2.206(13)
**3**: R = *cyclo*‐C_3_H_5_	91.29(13)	175.5(3)	2.216(4)
**4**: R = Ph	89.34(7) ‐ 94.79(7)	167.28 (av)	2.2154(19) ‐ 2.2208(19)
**6**: R = 4‐Cl‐C_6_H_4_	88.77(12) ‐ 93.50(11)	169.55 (av)	2.211(3) ‐ 2.219(3)
**7**: R = 4‐OMe‐C_6_H_4_	91.80(14) ‐ 92.86 (12)	164.63 (av)	2.199(4) ‐ 2.244(4)

### Release of Alkynyl Radicals from Bismuth Complexes

**Scheme 2:**
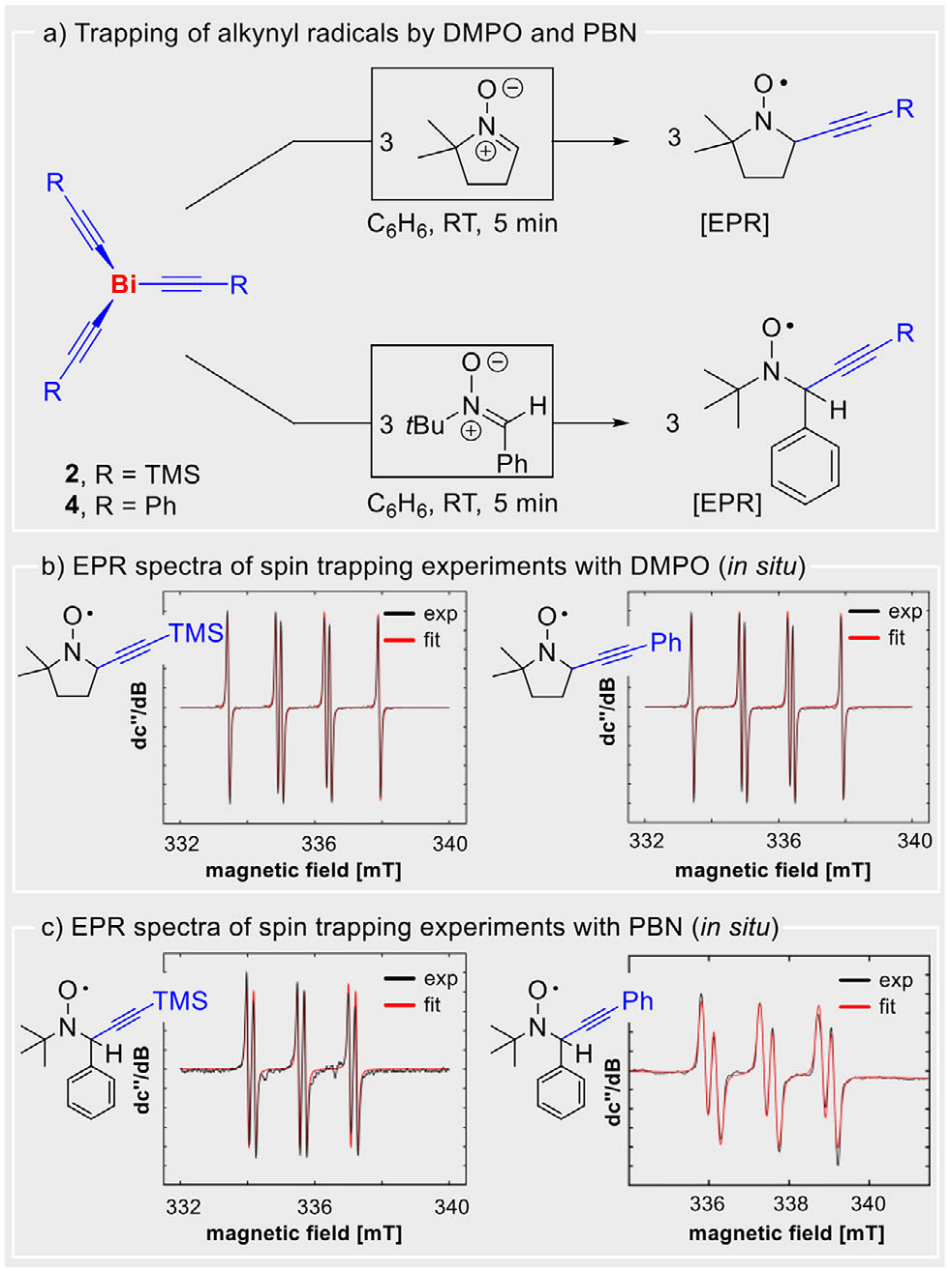
a) Trapping of alkynyl radicals released from Bi(C≡CSiMe_3_)_3_ and Bi(C≡CPh)_3_, using DMPO (dimethyl‐1‐pyrroline‐*N*‐oxide, top) and PBN (*N*‐*tert*‐butyl‐α‐phenyl‐nitrone, bottom) as a spin trap at room temperature. b) **Left**: Experimental (black) and simulated (red) continuous‐wave (CW) X‐band EPR spectra of a solution containing 1 equiv. [Bi(C≡CSiMe_3_)_3_] (*c* = 4*10^−2^ mol L^−1^) and 3 equiv. DMPO in benzene (0.5 mL). The observed resonance shows coupling constants of a(^14^N) = 40.4 MHz (14.4 G, 1.44 mT), a(^1^H) = 44.7 MHz (15.9 G, 1.59 mT) and a g_iso_ value of 2.0052. **Right**: Experimental (black) and simulated (red) continuous‐wave (CW) X‐band EPR spectra of a solution containing 1 equiv. [Bi(C≡CPh)_3_] (*c* = 4*10^−2^ mol L^−1^) and 3 equiv. DMPO in benzene (0.5 mL). The observed resonance shows coupling constants of a(^14^N) = 40.3 MHz (14.4 G, 1.44 mT), a(^1^H) = 44.6 MHz (15.9 G, 1.59 mT) and a g_iso_ value of 2.0052. c) **Left**: Experimental (black) and simulated (red) continuous‐wave (CW) X‐band EPR spectra of a solution containing 1 equiv. [Bi(C≡CSiMe_3_)_3_] (*c* = 4*10^−2^ mol L^−1^) and 3 equiv. PBN in benzene (0.5 mL). The observed resonance shows coupling constants of a(^14^N) = 42.5 MHz (15.1 G, 1.51 mT), a(^1^H) = 6.04 MHz (2.15 G, 0.215 mT) and a g_iso_ value of 2.0053. **Right**: Experimental (black) and simulated (red) continuous‐wave (CW) X‐band EPR spectra of a solution containing 1 equiv. Bi(C≡CPh)_3_ (*c* = 2·10^−2^ mol L^−1^) and 3 equiv. PBN in THF. The observed resonance shows coupling constants of a(1 × ^14^N) = 40.8 MHz (14.6 G, 1.46 mT), a(1 × ^1^H) = 7.91 MHz (2.82 G, 0.282 mT), and a g_iso_ value of 2.0051.

Some of the homoleptic bismuth alkynyl compounds presented in this work proved to be remarkably inert towards oxygen and moisture. Compounds **2** and **4** did not show any sign of degradation when a solution in C_6_D_6_ or CDCl_3_ was exposed to air for 24 h, as confirmed by NMR spectroscopic analysis. For long‐term storage, no decomposition was observed, when bismuth alkynyl compounds with aromatic substituents (**4**–**7**, **9**) were kept at ambient temperature under inert conditions. For the long‐term storage of compounds **2** and **3** with their silyl/alkyl substituents and the electron‐rich aryl species **8** temperatures of –30 °C under argon are advisable to avoid degradation. At elevated temperatures of 60–80 °C in solution, all the bismuth alkynyl species **2**–**9** undergo decomposition, which is notable after minutes or hours, depending on the nature of the compound, the solvent, and the exact temperature (see Supporting Information). In view of reports on facile Bi–X bond homolysis of molecular bismuth compounds (X = C, N, O, Mn),^[^ ^26^, ^30^, ^31^, ^34^, ^36^, ^53^ ^]^ the release of alkynyl radicals from **2**–**9** was hypothesized to be the major decomposition pathway under inert conditions. Based on DFT calculations, low homolytic Bi–C bond dissociation energies of 55 and 69 kcal·mol^−1^ are predicted for compounds **2**–**9** (Supporting Information), as opposed to large values of 129 kcal·mol^−1^ that have been reported for the homolytic cleavage of the C(sp)–H bond in phenylacetylene, for instance.^[^ ^54^ ^]^ Thus, EPR spectroscopic experiments with selected bismuth alkynyl compounds (**2** and **4**) were carried out in the presence of the spin traps DMPO (dimethyl‐1‐pyrroline‐*N*‐oxide) and PBN (*N*‐*tert*‐butyl‐α‐phenyl‐nitrone) at ambient temperature (Scheme 2). Indeed, straightforward and highly selective radical transfer was confirmed in all cases, even in the dark and without unwanted side products in the form of other trapped radicals being detectable (Scheme 2). Notably, reports on alkynyl radicals detected in spin trap experiments are extremely rare and are so far limited to transition metal complexes and photochemical approaches. In addition, these studies describe significant side reactions in the trapping experiments or lack a more detailed interpretation.^[^ ^15^, ^55^, ^56^, ^57^ ^]^ For instance, previously reported EPR spectroscopic data obtained during attempts to trap the [PhC≡C]^•^ radical (released from copper reagents under photochemical conditions (Xe lamp)), indicated significant contamination of the trapped radical with side products, which were assigned to unwanted oxidation reactions.^[^ ^15^ ^]^

**Scheme 3:**
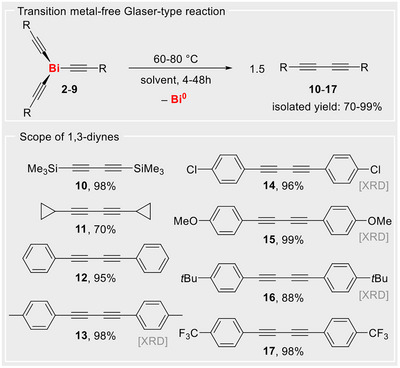
Synthesis of 1,3‐diynes **10**–**17** from well‐defined bismuth complexes Bi(C≡CR)_3_ (**2**–**9**). [XRD]: Single‐crystal X‐ray analyses available (Supporting Information).

Spurred on by the facile generation of alkynyl radicals from bismuth alkynyl complexes Bi(C≡CR)_3_, the behavior of compounds **2**–**9** in solution was further investigated. Under optimized conditions (heating in solution to 60–80 °C for 4‐48 h), the bismuth alkynyl compounds afforded the corresponding 1,3‐diynes **10–17** in high isolated yields, along with the formation of Bi^0^ (Scheme 3, for details see Supporting Information). These transformations represent Glaser‐type coupling reactions, which are key protocols when it comes to the synthesis of 1,3‐diynes — valuable building blocks in the generation of polymers, macrocycles, and rigid supramolecular architectures. Glaser‐type coupling reactions typically rely on the presence of transition metal catalysts^[^ ^15^, ^58^ ^]^ and/or the presence of (super‐)stoichiometric amounts of a base.^[^ ^58^, ^59^ ^]^ In contrast, our work demonstrates a straightforward, transition metal‐ and base‐free method for synthesizing 1,3‐diynes via radical homocoupling of bismuth alkynes.

The synthesis of unsymmetrical 1,3‐diynes from several combinations of two different bismuth alkynes Bi(C≡CR)_3_ in a 1:1 molar ratio was also investigated. As a result, mixtures of compounds were obtained, reflecting values close to the statistically expected ratios of homo‐ and hetero‐C–C‐coupling products (for details see Scheme S4 and Table S5), which is in congruency with a radical pathway for these coupling reactions.

### Proof of Principle: Intermolecular Alkynyl Radical Transfer

**Scheme 4:**
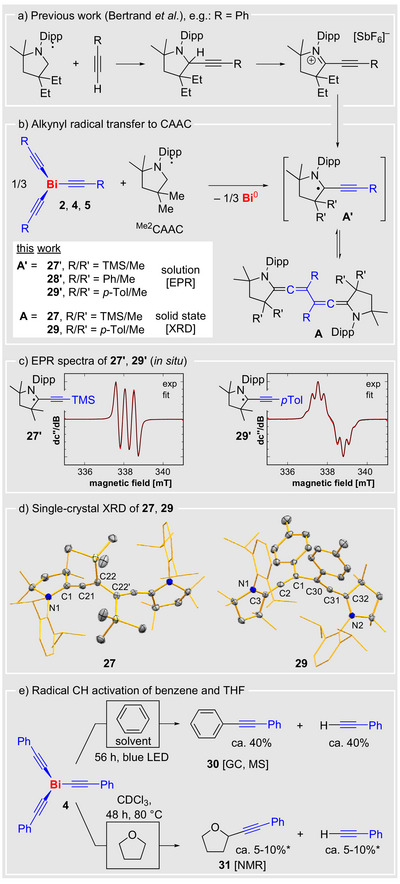
a) Alkynyl radical transfer to CAAC. Three‐step literature procedure to yield compounds of type **A’**/**A** with one‐electron‐reduction as the key step.^[^ ^60^ ^]^ b) This work granting access to compounds of type **A’**/**A** via alkynyl radical transfer reaction. c) Experimental (black) and simulated (red) continuous‐wave (CW) X‐band EPR spectra of **27′** (left) and **29′** (right) obtained by combining **2** and **5**, respectively, with 3 equiv. of CAAC in benzene at room temperature. **27′**: coupling constant = a(1 × ^14^N) = 12.7 MHz, g_iso_ = 2.0019. **29′**: coupling constants = a(1 × ^14^N) = 11.8 MHz, a(2 × ^1^H) = 7.65 MHz, a(3 × ^1^H) = 7.54 MHz, a(2 × ^1^H) = 2.63 MHz, g_iso_ = 2.0019. d) The molecular structures of **27** (left) and **29** (right) in the solid state. Hydrogen atoms are omitted for clarity. Displacement ellipsoids are shown at the 50% probability level. Selected bond lengths (Å) and angles (°): **27**: N1–C1 1.407(3), C1–C21 1.317(4), C21–C22 1.318(4), C22–C22’ 1.522(5); N1–C1–C21 127.3(2), C1–C21–C22 168.9(3), C21–C22–C22’ 123.9(2). **29**: N1–C3 1.391(2), C3–C2 1.320(3), C1–C2 1.321(3), C1–C30 1.518(3); N1–C3–C2 126.95(17), C3–C2–C1 165.19(19), C2–C1–C30 120.24(17). e) Radical C–H activation of benzene and THF with alkynyl radicals released from **4**; *: Glaser‐type C–C homocoupling leads to **12** as the main product in these reactions.

As a next step, we aimed to transfer alkynyl radicals from bismuth complexes Bi(C≡CR)_3_ to external substrates. In earlier work, Bertrand and co‐workers demonstrated that the reduction of alkynyl iminium salts derived from cyclic (alkyl)(amino) carbenes (CAACs) yields propargyl/allenyl radicals through a three‐step synthetic sequence, which dimerize in an equilibrium process depending on their substitution patterns (Scheme 4a).^[^ ^60^ ^]^ We envisioned that the transfer of an alkynyl radical to a CAAC should generate these species in a fundamentally different synthetic approach, specifically in a radical one‐step C–C coupling reaction. The addition of ^Me2^CAAC to compounds **3**, **4**, or **5** in THF or benzene resulted in an immediate color change to intensely dark red (Scheme 4b). Investigations of the reaction mixture by EPR spectroscopy and HR‐MS analysis confirmed the formation radical species **27′–29′**, which are closely related to previously published species with slightly modified substitution patterns at the CAAC and the alkynyl moiety (Scheme 4c).^[^ ^60^ ^]^ The dimerization products **27** and **29** were unambiguously identified by single‐crystal X‐ray diffraction analyses (Scheme 4d). The equilibrium between monomeric radical species **27′**/**29′** and the dimeric diamagnetic compounds **27**/**29** is in good agreement with previous studies.^[^ ^60^ ^]^ Additional observations confirm the ability of bismuth alkynyl complexes to undergo radical type reactions. The irradiation of a benzene solution of Bi(C≡CPh)_3_ (**4**) with a blue LED (*λ* = 460 nm) for 56 h led to the formation of diphenyl acetylene and phenyl acetylene (ca. 40% yield) as a result of C–H activation of benzene,^[^ ^61^ ^]^ along with minor amounts of the C–C homocoupling product, as shown by GC and HR‐MS analyses (Scheme 4e and Supporting Information). Similarly, the C–H activation of THF was observed, albeit in low yields of 5%–10%, when **4** was heated in THF for 48 h (Scheme 4e and Supporting Information).^[^ ^62^ ^]^ The same type of reaction has recently been reported for the visible light induced alkynylation of α‐C–H bonds of ethers with alkynyl bromides, which give the similar THF alkylation products.^[^ ^63^ ^]^ The observations on the generation of **27′**–**29′** as well as benzene and THF activation provide evidence for the viability of intermolecular alkynyl radical transfer reactions.

### Alkynyl Radical Transfer for the Synthesis of Alkynyl Aryl Seleno and Telluro ethers, Alkynyl–EPh (E = Se, Te)

In order to further exploit these reaction pathways, synthetic access to alkynyl aryl chalcogenides was targeted. These have attracted considerable interest in organic synthesis as vinyl precursors, dienophiles, or as substrates in click reactions^[^ ^64^, ^65^, ^66^, ^67^ ^]^ and in drug discovery due to their roles as antinociceptives or antioxidants.^[^ ^68^, ^69^, ^70^, ^71^ ^]^ Major routes to alkynyl chalcogenides include i) transition‐metal catalyzed reactions of alkynyl halides, alkynyl boronic acid esters, or alkynes with dichalcogenides or chalcogenyl halides and related substrates,^[^ ^72^, ^73^, ^74^, ^75^, ^76^ ^]^ ii) reactions of (air‐sensitive) alkynyl organometallics with organochalcogenyl halides operating via polar reaction pathways,^[^ ^77^, ^78^ ^]^ and iii) approaches relying on the utilization of harsh reaction conditions or strong bases.^[^ ^64^, ^79^, ^80^, ^81^ ^]^ In this study, we report a simple synthetic protocol to access alkynyl chalcogenides by the reaction of Bi(C≡CR)_3_ **2**–**9** with diphenyl dichalcogenides E_2_Ph_2_ (E = Se, Te), which we propose to occur via radical pathways. Specifically, the bismuth alkynes **2**–**9** were reacted with diphenyl dichalcogenides E_2_Ph_2_ (E = Se, Te). In this regard, diphenyl diselenide (Se_2_Ph_2_) is well‐known to undergo homolytic Se–Se bond cleavage in the presence of radical initiators.^[^ ^82^, ^83^ ^]^

**Scheme 5:**
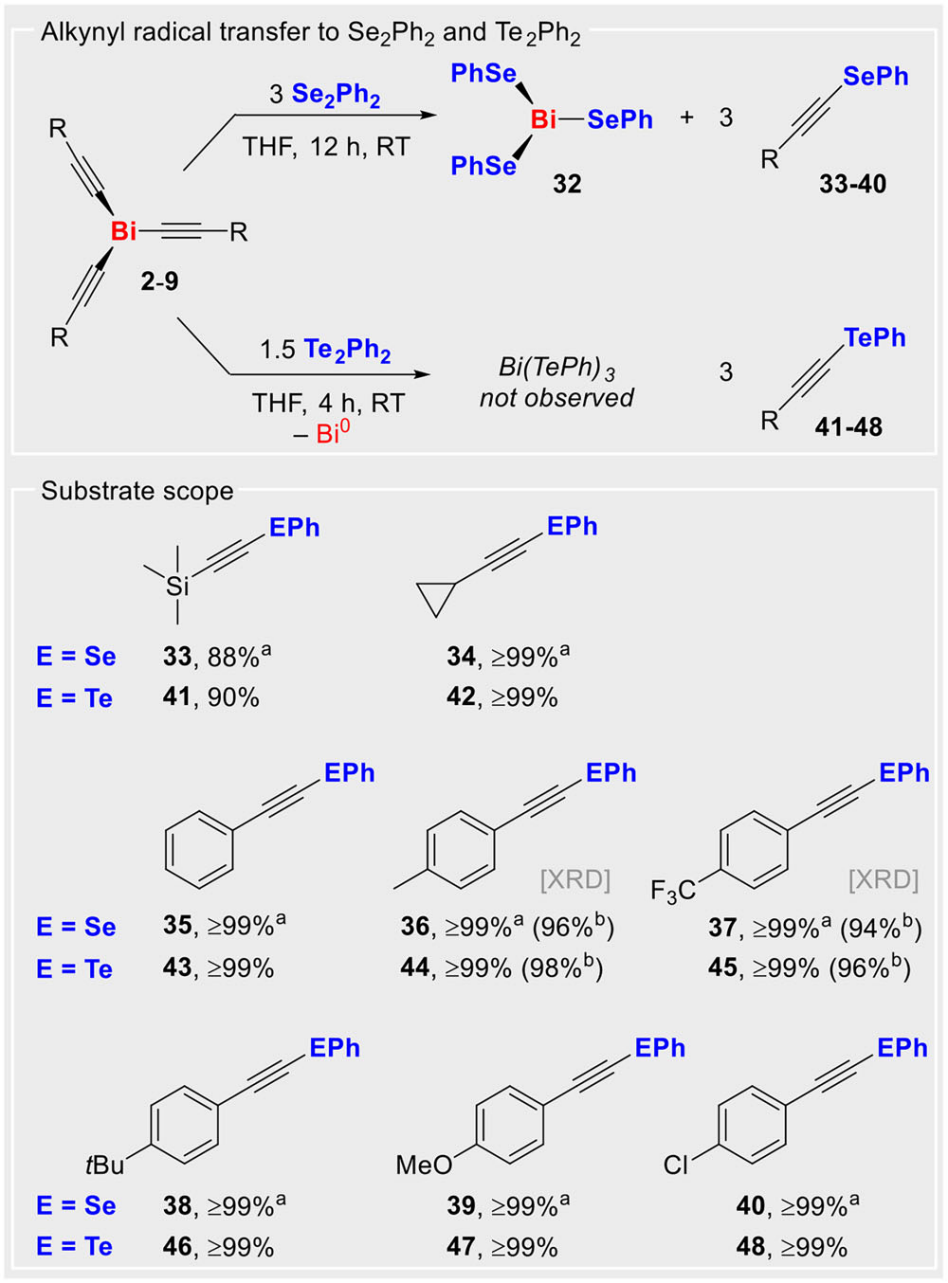
Transfer of the alkynyl radical to the dichalcogenides E_2_Ph_2_ (E = Se, Te) to give of the alkynyl chalcogenides **33**–**40** (E = Se) and **41**–**48** (E = Te) with spectroscopic yields. a) based on amount of starting material **2**–**9**. b) isolated yield. [XRD]: Single‐crystal X‐ray analyses available for **36** and **37** (Supporting Information).

When the bismuth alkynes **2**–**9** were reacted with Se_2_Ph_2_ in a 1:3 molar ratio for 12 h at room temperature, the desired alkynyl selenides **33**–**40** were obtained along with Bi(SePh)_3_ (**32**) in near‐quantitative yields (Scheme 5). A simple extraction yielded the pure isolated alkynyl selenides. The formation of the literature‐known^[^ ^84^ ^]^ Bi(SePh)_3_ was unambiguously identified by X‐ray diffraction analysis and ⁷⁷Se NMR spectroscopy. As a proof of principle, two alkynyl selenides, namely **36** (R = 4‐Me‐C_6_H_4_) and **37** (R = 4‐CF_3_‐C_6_H_4_), were isolated and fully characterized (Supporting Information). The remaining six alkynyl chalcogenides were characterized by ⁷⁷Se NMR spectroscopy and mass spectrometry (Supporting Information).

Furthermore, we also investigated the reaction of bismuth alkynes Bi(C≡CR)_3_ with 1.5 equiv. of diphenyl ditelluride to synthesize the alkynyl aryl tellurides **41**–**48** (Scheme 5). These reactions proceeded faster compared to those with the selenium analog, Se_2_Ph_2_. Notably, the formation of Bi(TePh)_3_ was not observed, likely due to the homolytically labile nature of the Bi–Te bonds in this species under the reaction conditions. Instead, a black precipitate (Bi^0^) was formed and separated by simple filtration to give the desired products in high yields of 90%–99%. Among the products, two alkynyl tellurides were isolated and fully characterized, namely compound **44** (R = 4‐Me‐C_6_H_4_) and compound **45** (R = 4‐CF_3_‐C_6_H_4_). The formation of the remaining six tellurides was confirmed by ^12^⁵Te NMR spectroscopy and mass spectrometry.

### Alkynyl Boronic Esters via Radical Pathways (C–B coupling)

**Scheme 6:**
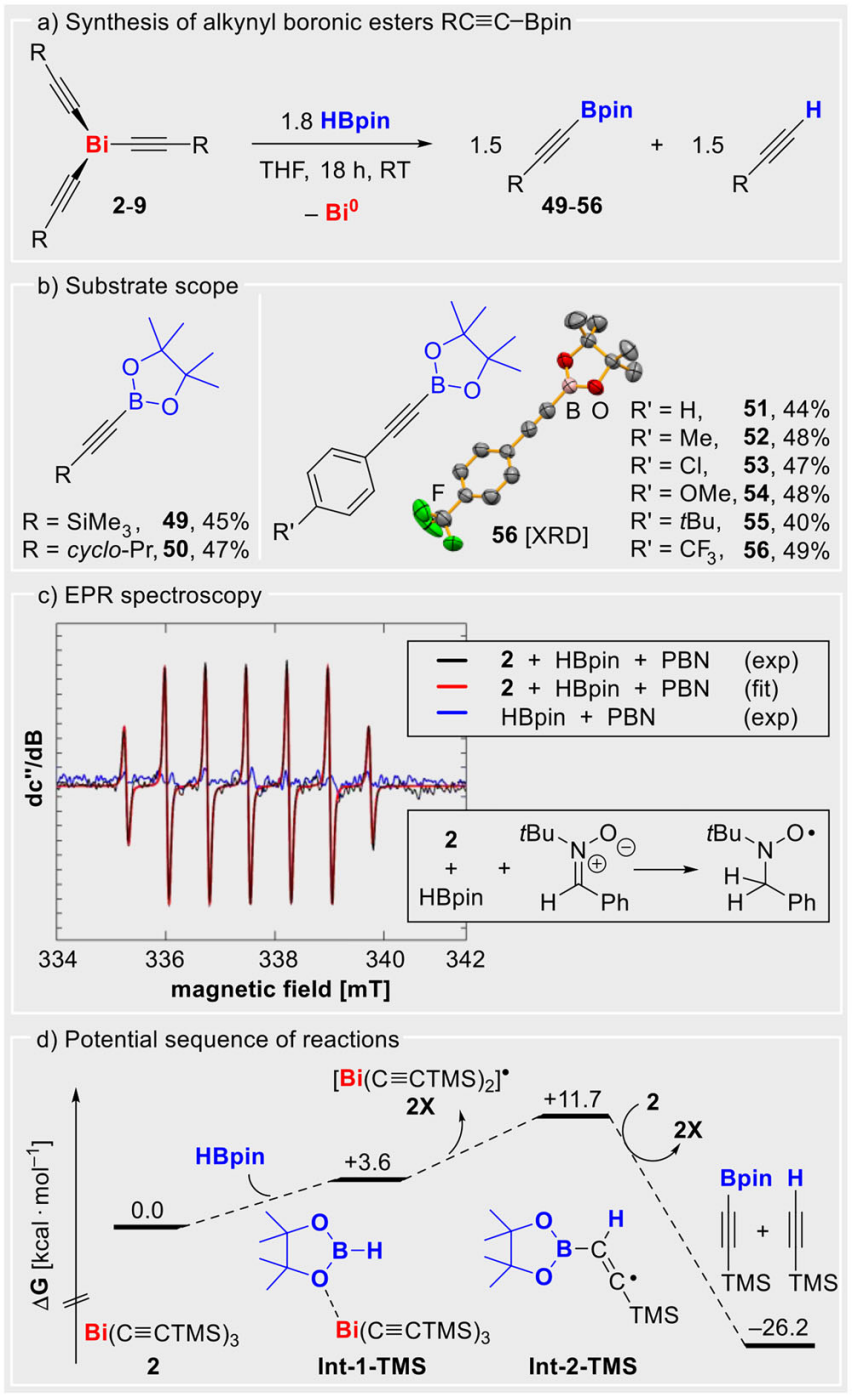
a) and b) Synthesis and scope of alkynyl boronic esters from Bi(C≡CR)_3_ (**2**–**9**) and HBpin. c) EPR spectroscopic reaction monitoring. Parameters for EPR spectroscopy: Experimental (black) and simulated (red) continuous‐wave (CW) X‐band EPR spectra of a solution containing 1 equiv. Bi(C≡CSiMe_3_)_3_ (*c* = 2.67·10^−2^ mol L^−1^), 1.5 equiv. HBpin, and 3 equiv. PBN in THF, measured at room temperature immediately after preparation. The observed resonance shows coupling constants of a(1 × ^14^N) = 41.9 MHz, a(2 × ^1^H) = 20.8 MHz, and a g_iso_ value of 2.0051, in agreement with the literature.^[^ ^89^ ^]^ d) Thermodynamic reaction profile determined by DFT calculations, suggesting radical‐assisted H atom transfer as a key step of the radical synthesis of alkynyl boronic esters (for details see Supporting Information).

Alkynyl boronic esters are widely applicable in organic synthesis, with the pinacol derivatives (RC≡C)Bpin being the most attractive targets.^[^ ^85^ ^]^ We aimed to explore the reactivity of the alkynyl radical source Bi(C≡CR)_3_ toward pinacol borane (HBpin), which represents one of the common substrates for the synthesis of (RC≡C)Bpin. The addition of 1.8 equiv. of HBpin to the alkynyl bismuth compounds **2**–**9** in THF at room temperature afforded 1:1 mixture of the free alkynes and the desired alkynyl boronic esters (Scheme 6). Quantitative conversion of the bismuth compounds was observed after 18 h reaction time, with isolated yields of 40%–49% for the boronic esters (calculated relative to Bi(C≡CR)_3_, 50% being the maximum theoretical yield).

While transition metal‐catalyzed transformations for the synthesis of alkynyl boronic esters have been reported to follow polar reaction pathways,^[^ ^86^, ^87^, ^88^ ^]^ the reactions between Bi(C≡CR)_3_ and HBpin create an unusual scenario. Hydride transfer from HBpin to poorly Lewis acidic Bi(C≡CR)_3_ or the deprotonation of HBpin by poorly Brønsted basic Bi(C≡CR)_3_ seem unfeasible. Instead, hydrogen atom abstraction from HBpin or related intermediates, while unprecedented in this context, appeared to be reasonable. To gain insights into mechanistic aspects of this transformation, the reaction between Bi(C≡CSiMe_3_)_3_ (**2**) and HBpin was monitored by EPR spectroscopy in the presence of the radical trap PBN. At early stages of the reaction, the trapped alkynyl radical that is detected in the absence of HBpin (*cf*. Scheme 2c) was not observed. Instead, the radical resulting from H atom transfer to PBN was unambiguously identified as the sole species (black and red trace in Scheme 6c). Importantly, this species was not observed when HBpin and PBN were investigated in the absence of **2** under otherwise identical conditions (blue trace in Scheme 6c). At later stages of the reaction, EPR spectra showed mixtures of the species resulting from alkynyl radical and H atom transfer to the spin trap PBN.

DFT calculations were performed in order to rationalize the important H atom abstraction step of the sequence of reactions leading to the alkynyl boronic esters **49**–**56** (Scheme 6a,d). The substrate HBpin features a calculated homolytic bond dissociation energy (BDE) of 99.7 kcal·mol^−1^ for the B–H bond, while a value of only 90.6 kcal·mol^−1^ was determined for the C–H bonds in the pinacol backbone (Supporting Information). As the methyl groups of HBpin remain untouched in the reaction, a more complex scenario was envisioned. The possibility of pre‐coordination of HBpin by alkynyl bismuth species was tested. Indeed, adduct formation between **2** and HBpin to give **Int‐1‐TMS** by weak O→Bi interactions is predicted to be only mildly endergonic (*ΔG_rel_* = +3.6 kcal·mol^−1^) and should be feasible in equilibrium. Intramolecular alkynyl radical transfer from Bi to B (followed by dissociation of the radical pair) to give [Bi(C≡CSiMe_3_)_2_]^•^ (**2X**) and [HB(C≡CSiMe_3_)pin]^•^ (with a tetrahedrally coordinated B atom) was probed. However, the latter radical species showed H atom migration from the boron atom to the adjacent carbon atom in the geometry optimization to give the intermediate **Int‐2‐TMS** (*ΔG_rel_* = +8.1 kcal·mol^−1^). The BDE of the C(sp^2^)–H bond in **Int‐2‐TMS** amounts to only 27.7 kcal·mol^−1^ (Supporting Information) so that reaction of **Int‐2‐TMS** with another equivalent of **2** would yield **2X** and the products Me_3_SiC≡CBpin (**49**) and HC≡CSiMe_3_ in an overall exergonic reaction (*ΔG* = –26.2 kcal·mol^−1^). Qualitatively identical results were obtained for the reaction of compound **4** with HBpin (with a lower energy of **Int‐2‐Ph** due to better delocalization of spin density). Thus, by modeling a partial, but important sequence of the reaction leading to alkynyl boronic esters (Scheme 6a,d), we tentatively suggest a radical‐assisted H atom abstraction to rationalize the course of these transformations.

In addition, it was demonstrated that the synthesis of alkynyl boronic esters from Bi(C≡CR)_3_ via radical pathways can also be conducted under photochemical conditions (Supporting Information). These results strongly point towards a radical mechanism for the synthesis of alkynyl boronic esters from HBpin and Bi(C≡CR)_3_, which is unprecedented to the best of our knowledge, and complementary to more established pathways involving polar group transfer reactions.

## Conclusion

The synthesis, full characterization, and detailed reactivity study of the first set of simple, homoleptic alkynyl bismuth compounds Bi(C≡CR)_3_ is presented. These studies establish the complexes Bi(C≡CR)_3_ as a readily accessible platform for the reliable generation of alkynyl radical species in the absence of any catalysts or co‐reagents. The release of alkynyl radicals can be triggered by visible‐light‐irradiation with a blue LED but, more importantly, it is also generally feasible by thermal excitation under moderate conditions—in many cases, even room temperature is sufficient. The compounds Bi(C≡CR)_3_ enable facile Glaser‐type C–C coupling. Highly selective alkynyl radical transfer to external substrates could be realized with cyclic alkyl(amino)carbenes, dichalcogenides, and pinacol borane, offering shortened synthetic pathways, transition metal‐ and base‐free conditions, or radical reactions where polar reaction pathways typically dominate. In the challenging field of alkynyl radical chemistry, the complexes Bi(C≡CR)_3_ represent a tunable, reliable, and operationally simple source of (RC≡C)^•^ radicals. This complements existing methods for alkynyl radical generation, which rely on photochemical conditions, the use of transition metals and toxic components, (superstoichiometric amounts of) co‐reagents, or harsh reaction conditions. It is anticipated that compounds of the type Bi(C≡CR)_3_ will readily be utilized by the synthetic community for valuable future applications in stoichiometric and catalytic alkynyl radical transfer reactions.

## Supporting Information

Please see the Supporting Information for experimental details, crystallographic details, and the relevant spectroscopic data. Deposition Numbers < url href=“2483707”> (for **2**), 2483714 (for **3**), 2483709 (for **4**), 2483718 (for **6**), 2483715 (for **7**), 2483719 (for **13**), 2483708 (for **15**), 2483716 (for **16**), 2483717 (for **27**), 2483711 (for **29**), 2483712 (for **36**), 2483710 (for **37**), and 2483713 (for **56**)</url > contain the supporting crystallographic data for this paper. These data are provided free of charge by the joint Cambridge Crystallographic Data Centre and Fachinformationszentrum Karlsruhe < url href=“http://www.ccdc.cam.ac.uk/structures”>Access Structures service</url > . Additional references cited within the Supporting Information.^[^ ^90^, ^91^, ^92^, ^93^, ^94^, ^95^, ^96^, ^97^, ^98^, ^99^, ^100^, ^101^, ^102^, ^103^, ^104^, ^105^, ^106^, ^107^, ^108^, ^109^, ^110^, ^111^, ^112^, ^113^, ^114^, ^115^, ^116^, ^117^, ^118^, ^119^ ^]^

## Conflict of Interests

The authors declare no conflict of interest.

## Supporting information



Supporting Information

## Data Availability

The data that support the findings of this study are available in the supplementary material of this article.
