# Supplementary material for: Homoleptic Bismuth Alkynes: Isolable Reagents for Selective Alkynyl Radical Transfer
Source: Angew Chem Int Ed Engl. 2025 Nov 23;65(4):e19525. doi: 10.1002/anie.202519525 (PMC12828473; doi:10.1002/anie.202519525)
Supplement: Supplementary file 1 — Supporting Information [file ANIE-65-e19525-s001.pdf]

Table of Contents

1. General experimental information 2
2. Synthesis of the homoleptic bismuth alkynes **2-9** 3
3. NMR and mass spectra of compounds **2-9** 7
4. Alkynyl radicals detection in EPR spectroscopic spin trap experiments 22
5. Synthesis and characterization of C–C homocoupling products **10-17** 26
6. Synthesis and MS data of C–C cross-coupling products 47
7. Abstraction of (C≡CR)$^{\bullet}$ radicals from Bi(C≡CR)$_3$ with CAAC 55
8. Reactions with THF and benzene 62
9. Synthesis of alkynyl chalcogenides (E = Se, Te) 63
10. Reactions of alkynyl bismuth compounds with HBpin 87
11. Bonding parameters of from single-crystal XRD data 106
12. Crystallographic details 116
13. Molecular structures of **13-16**, **36**, **37**, **56** 121
14. Computational details 123

1. General experimental information

All air- and moisture-sensitive steps have been carried out under an inert atmosphere of purified argon (5.0) using standard Schlenk techniques and a glovebox (GS systems) up to the point, when an aqueous workup was started (if applicable). Solvents were dried over the appropriate drying agents, distilled, and stored over 3 or 4 Å molecular sieves. Bismuth (III) chloride was sublimed prior to use. $Bi(NMe_2)_3$ and ^{Me2}CAAC was prepared according to literature protocols.[52,90] Commercially available Bismuth (III) bromide was used as received. NMR spectra were recorded on Bruker AV400, AV500, or Bruker 500 HD spectrometers and referenced against residual ^{1}H, ^{13}C, ^{11}B, ^{19}F, ^{29}Si, ^{77}Se and ^{125}Te signals of the respective deuterated solvent. ^{1}H, ^{13}C, and ^{29}Si chemical shifts are reported relative to $SiMe_4$ using the residual solvent peak of the solvent as a secondary standard. ^{19}F and ^{11}B chemical shifts are reported relative to $CFCl_3$ and BF_3 as an external standard, respectively. ^{77}Se and ^{125}Te NMR chemical shifts are reported relative to $SeMe_2$ (plus 5 % C_6D_6) and $TeMe_2$ (plus 5 % C_6D_6) as external standards. NMR spectra were recorded at ambient temperature (typically 300 K), if not otherwise noted. X-band EPR spectroscopic measurements were carried out at a given temperature (room temperature, if not otherwise indicated), using a Bruker ELEXSYS E580 CW/FT EPR spectrometer. The spectral simulations were performed using MATLAB 9.6 (2019a) and the EasySpin 5.2.25 toolbox.[91] Cyclic voltammograms were recorded using a *Gamry Interface 1010 potentiostat*. A standard three-electrode cell configuration was employed using a platinum disk working electrode, a platinum wire counter electrode, and a silver wire, separated by a *Vycor*® tip, serving as the reference electrode. Concentrations of 1 mM of analyte and 0.1 M nBu$_4$N[PF$_6$], which acted as electrolyte, were used in the default measurement setup. The [FeCp$_2$] / [FeCp$_2$]$^+$ (Fc/Fc$^+$) redox couple was utilized as an internal standard. Peak potentials and currents of the second cycle of each measurement were determined using the *Gamry Framework^{TM} Data Acquisition* Software (ver. 7.9.0). Elemental analyses were performed on a Vario Micro Cube by Elementar Analysesysteme GmbH. HR-ESI mass spectra were acquired with an Orbitrap Q Exactive plus mass spectrometer (Thermo Fischer Scientific), with a resolution set to 140,000. HR-FD/FI/LIFDI mass spectra were acquired with an AccuTOF GCv 4G (JEOL) Time of Flight (TOF) mass spectrometer. An internal or external standard was used for drift time correction. LIFDI-mass spectra were recorded on a JEOL Accu TOF GCv time-of-flight mass spectrometer. Single-crystal X-ray diffraction data were recorded on a Bruker D8 Quest diffractometer equipped with a Photon 100 detector using Mo−Kα-radiation.

2. Synthesis of the homoleptic bismuth alkynes 2-9

Synthesis of [Bi(C≡CSiMe$_3$)$_3$] (2). One equivalent of Bi(NMe$_2$)$_3$ (0.200 g, 0.58 mmol) was dissolved in THF (5 mL) and 3 equivalents of trimethylsilylacetylene (0.190 g, 1.9 mmol) were added. The color of the reaction mixture immediately changed from yellow to colorless, and the mixture was immediately dried under reduced pressure to obtain white-colored powder as a pure product **2**. Colorless crystals suitable for single-crystal X-ray diffraction analysis were obtained after keeping the concentrated THF solution at room temperature for 48 h. Yield: 250 mg, 0.50 mmol, 86%.

^{1}H NMR (500 MHz, 298 K, C$_6$D$_6$): δ = 0.10 (s, 27 H, Si(C*H*$_3$)$_3$) ppm. **^{13}C{^{1}H} NMR (126 MHz, 298 K, C$_6$D$_6$):** δ = 0.25 (Si(*C*H$_3$)$_3$), 120.5 (fwhm = 61.62 Hz, Bi-C≡*C*SiMe$_3$), 122.9 (Bi-*C*≡CSiMe$_3$) ppm. **^{29}Si{^{1}H} NMR (126 MHz, 298 K, CDCl$_3$):** δ = 18.41(Bi-C≡C*Si*Me$_3$) ppm. **HR-MS (LIFDI, pos.):** Calc. for(^{12}C$_{15}$^{1}H$_{28}$^{209}Bi^{28}Si$_3$)$^+$([**2**+H]$^+$): m/z = 502.13026, found m/z = 502.13365. **Elemental analysis (%),** calc. for C$_{15}$H$_{27}$Bi$_1$Si$_3$ (500.62 gmol^{-1}): C 35.99, H 5.44; found: C 35.69, H 5.32.

Synthesis of [Bi(C≡C*cyclo*Pr)$_3$] (3). One equivalent of Bi(NMe$_2$)$_3$ (0.200 g, 0.58 mmol) was dissolved in THF (5 mL) and 3 equivalents of *cyclo*propylacetylene (0.125 g, 1.9 mmol) were added. The color of the reaction mixture immediately changed from yellow to colorless, and the mixture was immediately dried under reduced pressure to obtain a white powder as a pure product **3** (the drying process has to be monitored carefully, as exposure to reduced pressure can also foster the decomposition of **3**). Colorless crystals suitable for single-crystal X-ray diffraction analysis were obtained by allowing the concentrated THF solution with a hexane layer to stand at room temperature for 24 hours. Yield: 185 mg, 0.46 mmol, 78%.

^{1}H NMR (500 MHz, 298 K, CDCl$_3$): δ = 0.74-0.77 (d, *J* = 6.7 Hz, 3*4 H, CH(C*H*$_2$)$_2$), 1.37 (m, 3*1 H, C*H*(CH$_2$)$_2$) ppm. **^{13}C{^{1}H} NMR (126 MHz, 298 K, CDCl$_3$):** δ = 0.9 (CH(*C*H$_2$)$_2$), 8.8 (*C*H(CH$_2$)$_2$), 87.5 (fwhm = 97.3 Hz, Bi-*C*) 118.0 (Bi-C*C*) ppm. **HR-MS (LIFDI, pos.):** Calc. for (^{12}C$_{10}$^{1}H$_9$^{209}Bi)$^+$ ([**3** – HC≡C*cyclo*Pr]$^{+\bullet}$): m/z = 338.0502, found m/z = 338.0522. Calc. for (^{209}Bi)$^+$ ([**3** – 3 C≡C*cyclo*Pr]$^+$): m/z = 208.9799, found m/z = 208.9798.

Synthesis of [Bi(C≡CPh)$_3$] (4). One equivalent of Bi(NMe$_2$)$_3$ (0.200 g, 0.58 mmol) was dissolved in THF (5 mL) and 3 equivalents of phenylacetylene (0.194 g, 1.9 mmol) were added. The color of the reaction mixture immediately changed from yellow to colorless, and the mixture was immediately under reduced pressure to obtain a white powder as a pure product **4**. Colorless crystals suitable for single-crystal X-ray diffraction analysis were obtained after keeping the concentrated THF solution at –30 °C for 48 h. Yield: 250 mg, 0.48 mmol, 84%.

^{1}H NMR (500 MHz, 298 K, Py-d$_5$): δ = 7.20 (m, 3*3 H, Ph-*H*), 7.53 (m, 3*2 H, Ph-*H*) ppm. **^{1}H NMR (500 MHz, 298 K, CD$_2$Cl$_2$):** 7.34 (d, 3*3 H, Ph-*H*), 7.53 (m, 3*2 H, Ph-*H*) ppm. **^{13}C{^{1}H}**

NMR (126 MHz, 298 K, Py-d$_5$): δ = 111.9 (fwhm = 75.3 Hz, Bi-C≡*C*Ph), 119.9 (Bi-*C*≡CPh), 125.7 (*ipso*-Ar-*C*), 128.8 (*m*-Ar-*C*), 129.4 (*o*-Ar-*C*), 132.9 (*p*-Ar-*C*) ppm. **HR-MS (LIFDI, pos.):** Calc. for ($^{12}C_{16}{}^{1}H_{10}{}^{209}Bi)^+$ ([**4** – (C≡CPh)]$^+$): m/z = 411.0582, found 411.0597. Calc. for ($^{12}C_{8}{}^{1}H_{5}{}^{209}Bi)^{\bullet+}$ ([**4** – 2 (C≡CPh)]$^+$): m/z = 310.0190, found 310.0184. **Elemental analysis (%),** calc. for $C_{24}H_{15}Bi$ (512.36 gmol^{-1}): C 56.26, H 2.95; found: C 55.68, H 3.01.

Synthesis of [Bi(C≡C(*p*Tol))$_3$] (5). One equivalent of Bi(NMe$_2$)$_3$ (0.200 g, 0.58 mmol) was dissolved in THF (5 mL) and 3 equivalents of 4-ethynyltoluene (0.220 g, 1.9 mmol) were added. The color of the reaction mixture immediately changed from yellow to colorless, and the mixture was immediately dried under reduced pressure to obtain a colorless powder as a pure product **5**. Yield: 285 mg, 0.51 mmol, 88%.

^{1}H NMR (500 MHz, 298 K, CDCl$_3$): δ = 2.36 (s, 3*3 H, C*H*$_3$), 7.13 (d, *J* = 7.85 Hz, 3*2 H, 2,6-*p*Tol), 7.42 (d, *J* = 8.12 Hz, 3*2 H, 3,5-*p*Tol) ppm. **^{13}C{^{1}H} NMR (126 MHz, 298 K, CDCl$_3$):** δ = 21.5 (*C*H$_3$), 102.5 (fwhm = 61.0 Hz, Bi-*C*≡C(*p*Tol)), 113.7 (*Bi*-C*C*(*p*Tol)), 119.9 (1-*p*Tol), 128.9 (3,5-*p*Tol), 132.2 (4-*p*Tol), 138.8 (2,6-*p*Tol) ppm. **HR-MS (LIFDI, pos.):** Calc. for ($^{12}C_{27}{}^{1}H_{21}{}^{209}Bi^{35})^+$ ([**5**]$^+$): m/z = 554.1442, found m/z = 554.1437. **Elemental analysis (%),** calc. for $C_{27}H_{21}Bi$ (554.45 gmol^{-1}): C 58.49, H 3.82; found: C 58.09, H 4.05.

Synthesis of [Bi(C≡C(4-Cl-C$_6$H$_4$))$_3$] (6). One equivalent of Bi(NMe$_2$)$_3$ (0.200 g, 0.58 mmol) was dissolved in THF (5 mL) and 3 equivalents of 1-chloro-4-ethynylbenzene (0.260 g, 1.9 mmol) were added. The color of the reaction mixture immediately changed from yellow to colorless, and the mixture was immediately dried under reduced pressure to obtain a colorless powder as a pure product **6**. Colorless crystals suitable for single-crystal X-ray diffraction analysis were obtained after keeping the concentrated THF solution at room temperature for 24 h. Yield: 310 mg, 0.50 mmol, 86%.

^{1}H NMR (500 MHz, 298 K, THF-d$_8$): δ = 7.29 (m, 3*2 H, 3,5-(4-Cl-C$_6$H$_4$)), 7.40 (m, 3*2 H, 2,6-(4-Cl-C$_6$H$_4$)) ppm. **^{13}C{^{1}H} NMR (126 MHz, 298 K, THF-d$_8$):** δ = 110.0 (Bi-C≡*C*(4-Cl-C$_6$H$_4$)), 117.1 (fwhm = 64.0 Hz, Bi-*C*≡C(4-Cl-C$_6$H$_4$)), 124.1 (3,5-(4-Cl-C$_6$H$_4$)), 129.4 (4-(4-Cl-C$_6$H$_4$)), 134.1 (1-(4-Cl-C$_6$H$_4$)), 134.4 (2,6-(4-Cl-C$_6$H$_4$)) ppm. **HR-MS (LIFDI, pos.):** Calc. for ($^{12}C_{16}{}^{1}H_{8}{}^{35}Cl_2)^+$ ([**6**-(Bi-CC-4-Cl-C$_6$H$_4$)]$^+$): m/z = 269.9998, found m/z = 269.9999 (mass for homocoupling product is observed). **Elemental analysis (%),** calc. for $C_{24}H_{12}BiCl_3$ (615.69 gmol^{-1}): C 46.82, H 1.96; found: C 46.28 H 2.39.

Synthesis of [Bi(C≡C(4-OMe-C$_6$H$_4$))$_3$] (7). One equivalent of Bi(NMe$_2$)$_3$ (0.200 g, 0.58 mmol) was dissolved in THF (5 mL) and 3 equivalents of 4-ethynylanisole (0.251 g, 1.9 mmol) were added. A yellow-colored precipitate formed immediately. The liquid phase was decanted and

the yellow solid was dried after to obtain a pure product **7**. Colorless crystals suitable for single-crystal X-ray diffraction analysis were obtained after keeping the concentrated THF solution at room temperature for 48 h. Yield: 295 mg, 0.49 mmol, 84%.

^{1}H NMR (500 MHz, 298 K, $CDCl_3$): δ = 3.81 (s, 3*3 H, OCH_3), 6.82 (d, *J* = 8.82 Hz, 3*2 H, Ar-*m-H*), 7.47 (d, *J* = 8.73 Hz, 3*2 H, Ar-*o-H*) ppm. **^{13}C{^{1}H} NMR (126 MHz, 298 K, $CDCl_3$):** δ = 55.2 (OCH_3), 101.6 (fwhm = 62.0 Hz, Bi-*C*≡CAr), 113.8 (Bi-C≡*C*Ar), 113.9 (*m-Ar*), 115.1 (*ipso-Ar*), 133.8 (*o-Ar*), 159.8 (*p-Ar*) ppm. **HR-MS (LIFDI, pos.):** Calc. for $(^{12}C_{27}{}^1H_{21}{}^{209}Bi^{16}O_3)^+$ ([**7**]$^+$): m/z = 602.1289, found m/z = 602.1279. **Elemental analysis (%),** calc. for $C_{27}H_{21}BiO_3$ (600.81 gmol^{-1}): C 53.83, H 3.51; found: C 53.65, H 3.75.

Synthesis of [Bi(C≡C(4-*t*Bu-C_6H_4))$_3$] (8). One equivalent of Bi(NMe_2)$_3$ (0.200 g, 0.58 mmol) was dissolved in THF (5 mL) and 3 equivalents of 1-butyl-4-ethynylbenzene (0.300 g, 1.9 mmol) were added. The color of the reaction mixture immediately changed from yellow to colorless, and the mixture was immediately dried under reduced pressure to obtain a colorless powder as a pure product **8**. Yield: 260 mg, 0.38 mmol, 65%.

^{1}H NMR (500 MHz, 298 K, $CDCl_3$): δ = 1.31 (3, 3*9 H, C(C*H*$_3$)$_3$), 7.31 (d, *J* = 8.31 Hz, 3 *2 H, *m*-Ar), 7.47 (d, *J* = 8.31 6 H, *o*-Ar) ppm. **^{13}C{^{1}H} NMR (126 MHz, 298 K, $CDCl_3$):** δ = 31.2 (C(CH_3)$_3$), 34.8 (*C*(CH_3)$_3$), 101.6 (fwhm = 63.0 Hz, Bi-*C*≡CAr), 103.1 (Bi-C≡*C*Ar), 119.5 (*ipso-Ar*), 125.1 (*m-Ar*), 132 (*o-Ar*), 151.8 (*p-Ar*) ppm. **HR-MS (LIFDI, pos.):** Calc. for $(^{12}C_{36}{}^1H_{39}{}^{209}Bi)^+$ ([**8**]$^+$): m/z = 680.2850, found m/z = 680.2834. **Elemental analysis (%),** calc. for $C_{20}H_{31}BiCl_3N_1$ (680.29 gmol^{-1}): C 63.52, H 5.78; found: C 63.22, H 6.27.

Synthesis of [Bi(C≡C(4-CF_3-C_6H_4))$_3$] (9). One equivalent of Bi(NMe_2)$_3$ (0.200 g, 0.58 mmol) was dissolved in THF (5 mL) and 3 equivalents of 1-ethynyl-4-(trifluoromethyl)benzene (0.323 g, 1.9 mmol) were added. The color of the reaction mixture immediately changed from yellow to colorless, and the mixture was immediately under reduced pressure to obtain colorless powder as a pure product **9**. Yield: 285 mg, 0.40 mmol, 68%.

^{1}H NMR (500 MHz, 298 K, $CDCl_3$): δ = 7.57-7.66 (m, 3 *4 H, Ar) ppm.**^{19}F{^{1}H} NMR (126 MHz, 298 K, $CDCl_3$):** δ = –62.9 (CF_3) ppm. **^{13}C{^{1}H} NMR (126 MHz, 298 K, CD_2Cl_2):** δ = 108.4 (fwhm = 59.13 Hz, Bi–*C*≡CAr), 112.3 (Bi-C≡*C*Ar), 124.5 (q, $^1J_{C-F}$ = 271.8 Hz, CF_3), 125.6 (q, $^3J_{C-F}$ = 3.8 Hz, *m*-Ar), 127.00 (*ipso-Ar*), 130.8 (q, $^2J_{C-F}$ = 32.4 Hz, *p-Ar*), 133.0 (*o-Ar*) ppm. **HR-MS (LIFDI, pos.):** Calc. for $(^{12}C_{27}{}^1H_{12}{}^{209}Bi^{19}F_9{}^+$ ([**9**]$^+$): m/z = 716.0594, found m/z = 716.0587. **Elemental analysis (%)**, calc. for $C_{27}H_{12}Bi_1F_9(C_4H_8O)_{0.3}$ (737.99 gmol^{-1}): C 45.90, H 1.97; found: C 45.71, H 2.31.

Table S1. Selected ^{13}C NMR chemical shifts of terminal alkynes H*C*≡CR relevant to this work.

R	δ (H*C*≡CR) [ppm]	solvent	reference
$SiMe_3$	89.9	$CDCl_3$	this work
cyclo-Pr	63.3	$CDCl_3$	[92]
Ph	83.3	$CDCl_3$	this work
p-Me-C_6H_4	75.9	$CDCl_3$	[93]
p-Cl-C_6H_4	78.2	$CDCl_3$	[94]
p-OMe-C_6H_4	76.0	$CDCl_3$	[94]
p-O*t*Bu-C_6H_4	76.5	$CDCl_3$	[93]
p-CF_3-C_6H_4	82.2	$CDCl_3$	this work

3. NMR and mass spectra of compounds 2-9

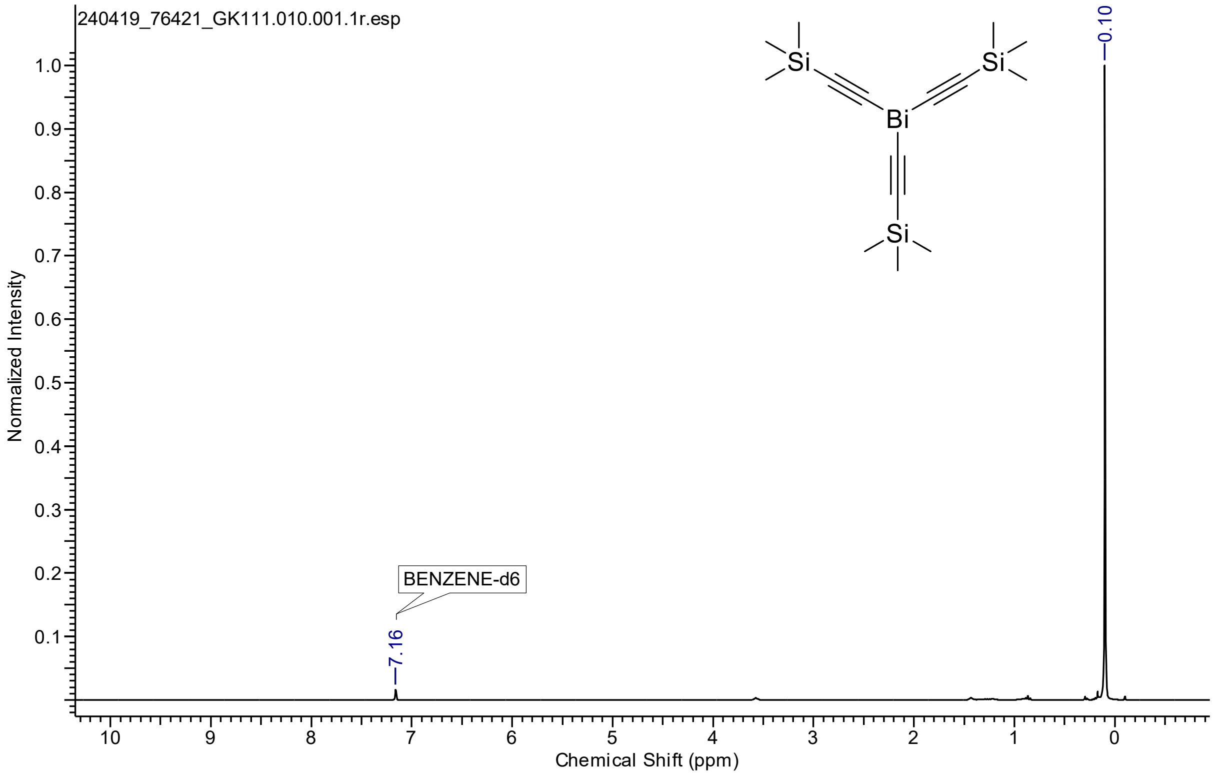

Figure S1. ^{1}H NMR spectrum of **2** in C_6D_6.

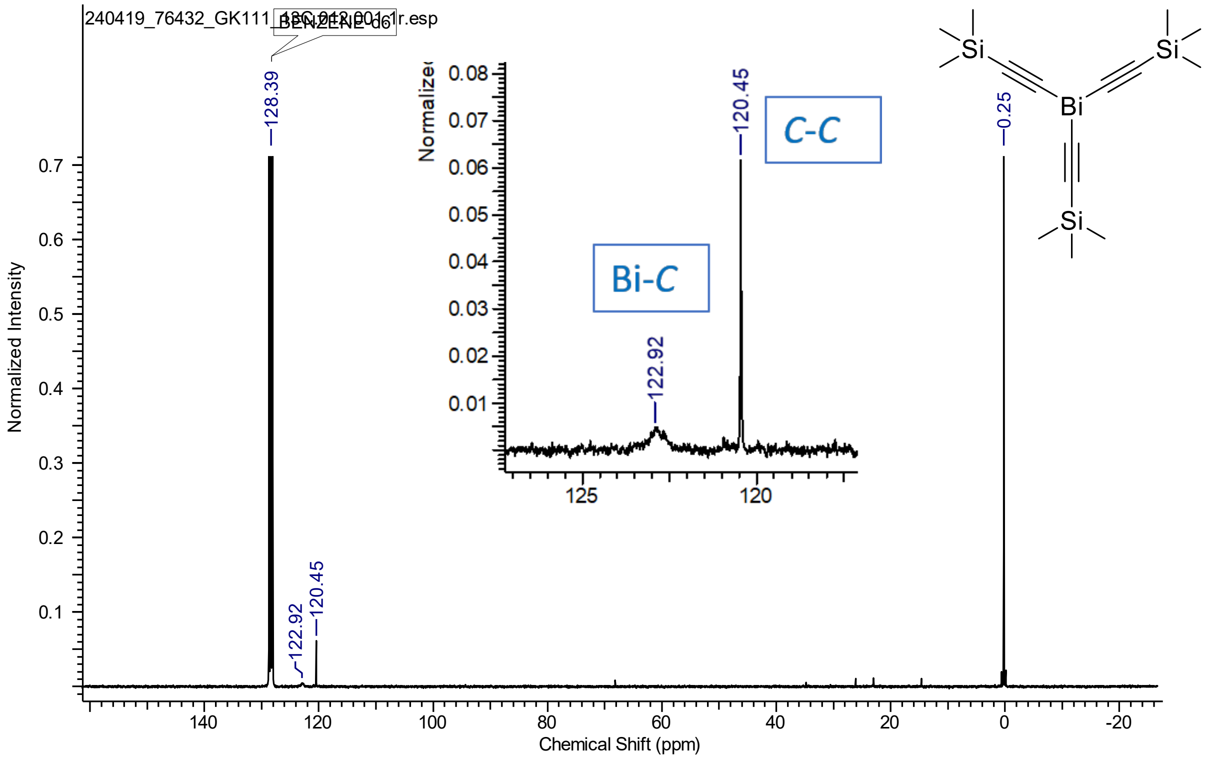

Figure S2. ^{13}C NMR spectrum of **2** in C_6D_6.

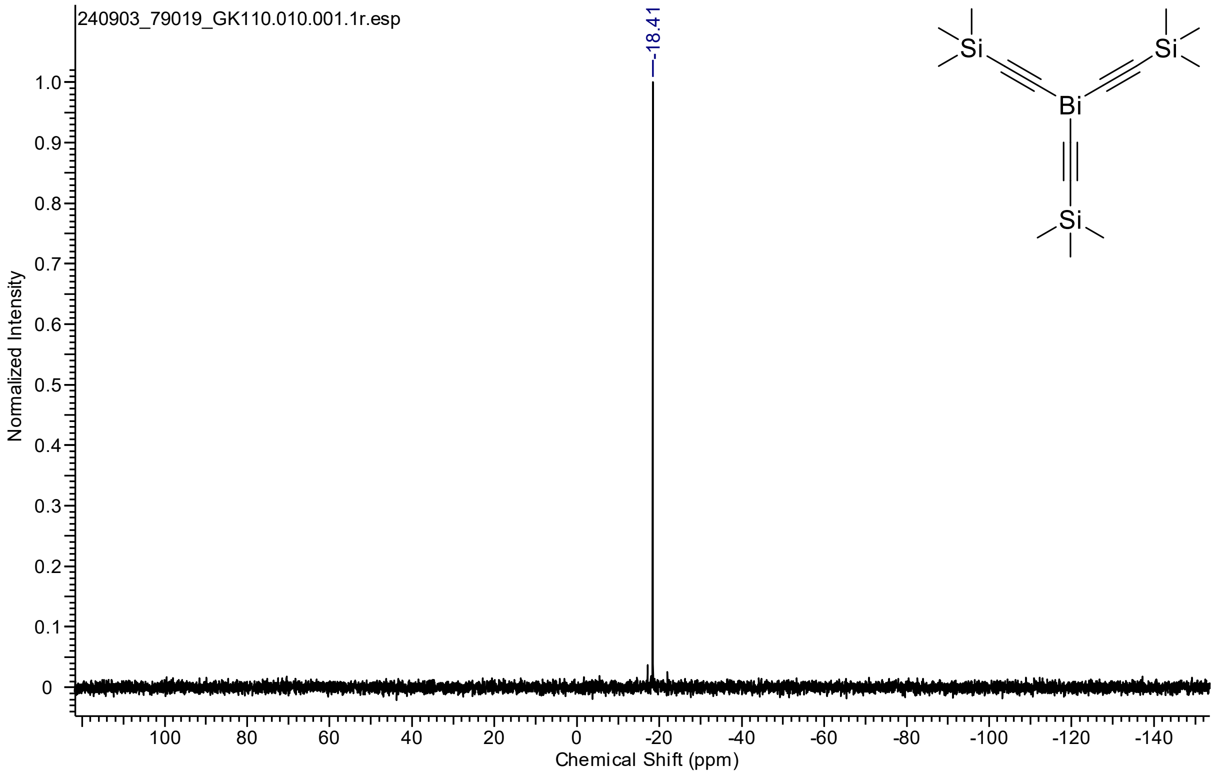

Figure S3. ^{28}Si NMR spectrum of **2** in C_6D_6.

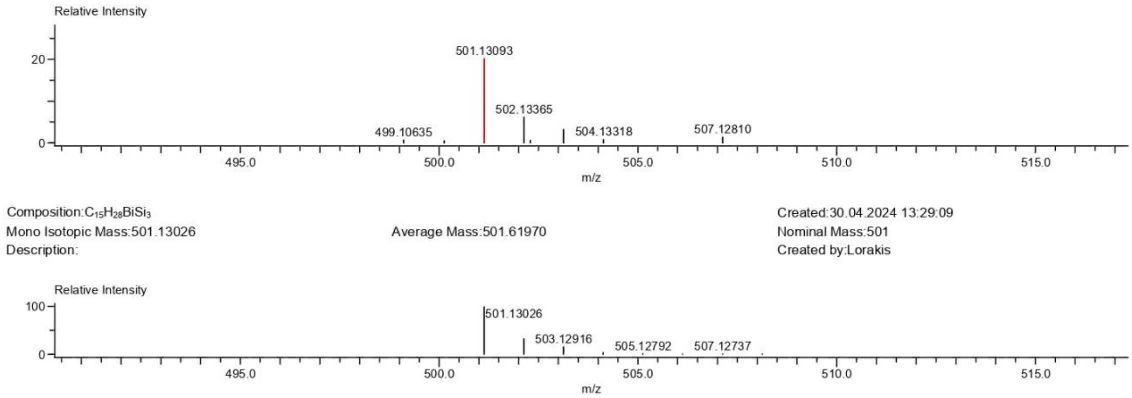

Mass	Intensity	Relative Intensity	Calc. Mass	Mass Difference [mDa]	Mass Difference [ppm]	Possible Formula	Unsaturation Number
501.13093	28059.35	20.07	501.13026	0.67	1.33	$^{12}C_{15}{}^{1}H_{28}{}^{209}Bi_{1}{}^{28}Si_{3}$	5.5

Figure S4. HR-MS spectrum of **2**.

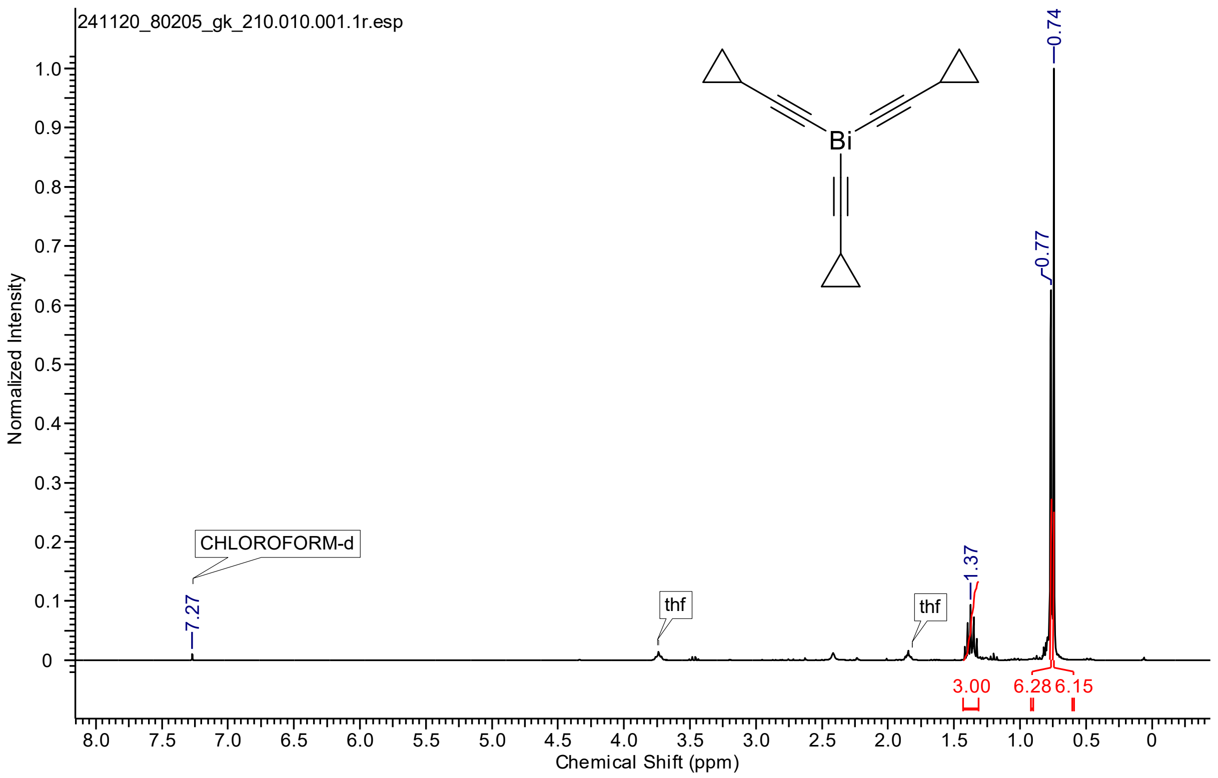

Figure S5. ^{1}H NMR spectrum of **3** in $CDCl_3$.

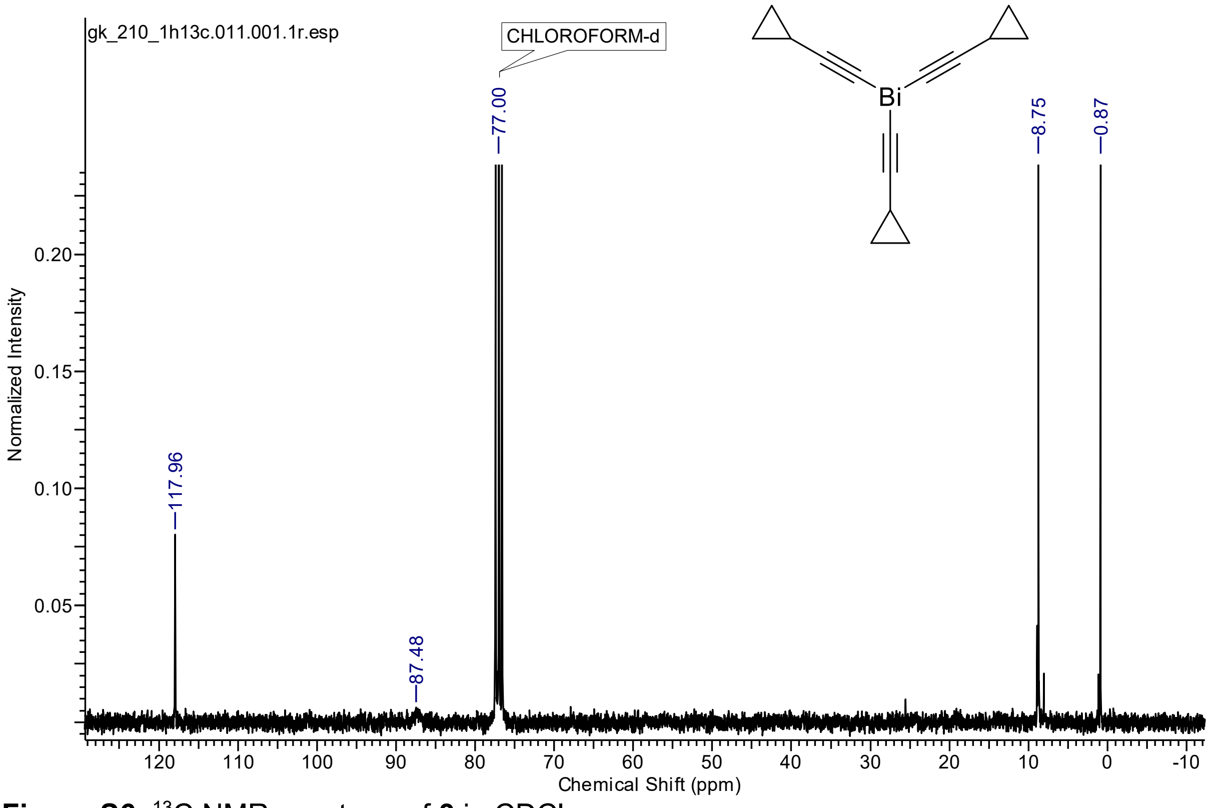

Figure S6. ^{13}C NMR spectrum of **3** in $CDCl_3$.

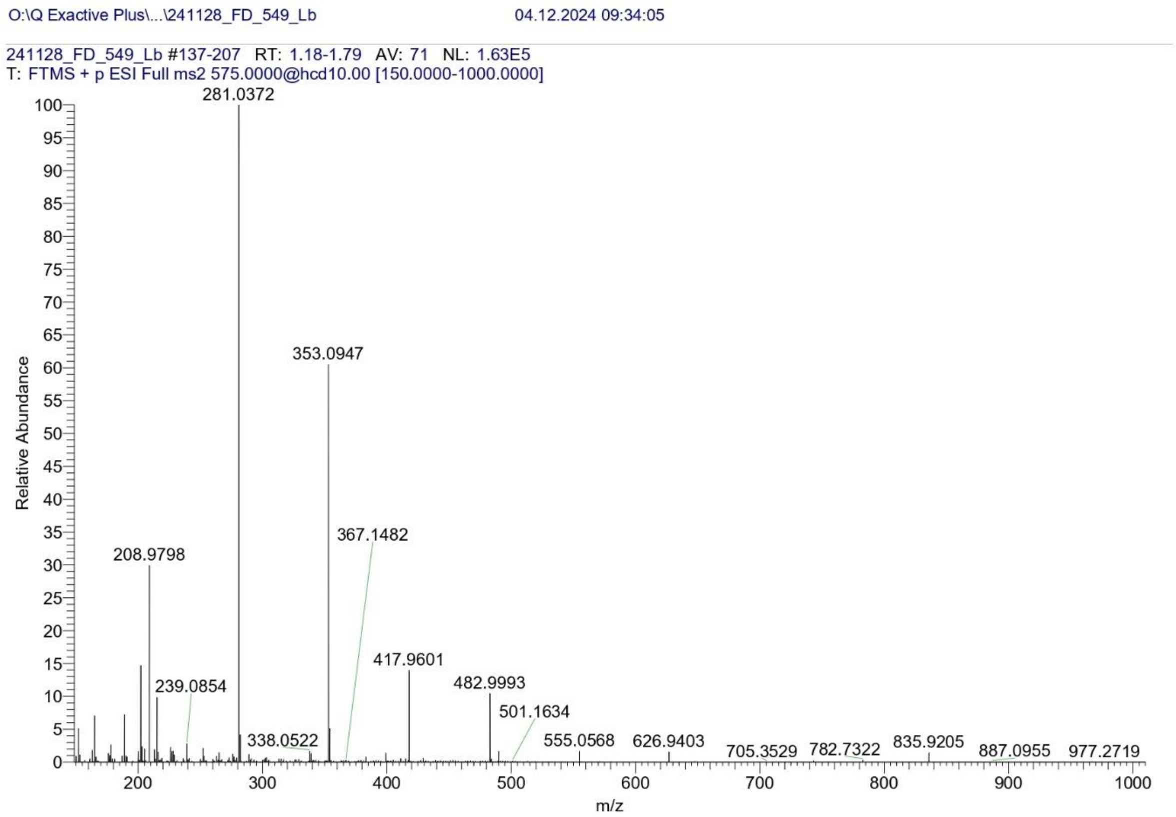

Figure S7. HR-MS spectrum of **3.**

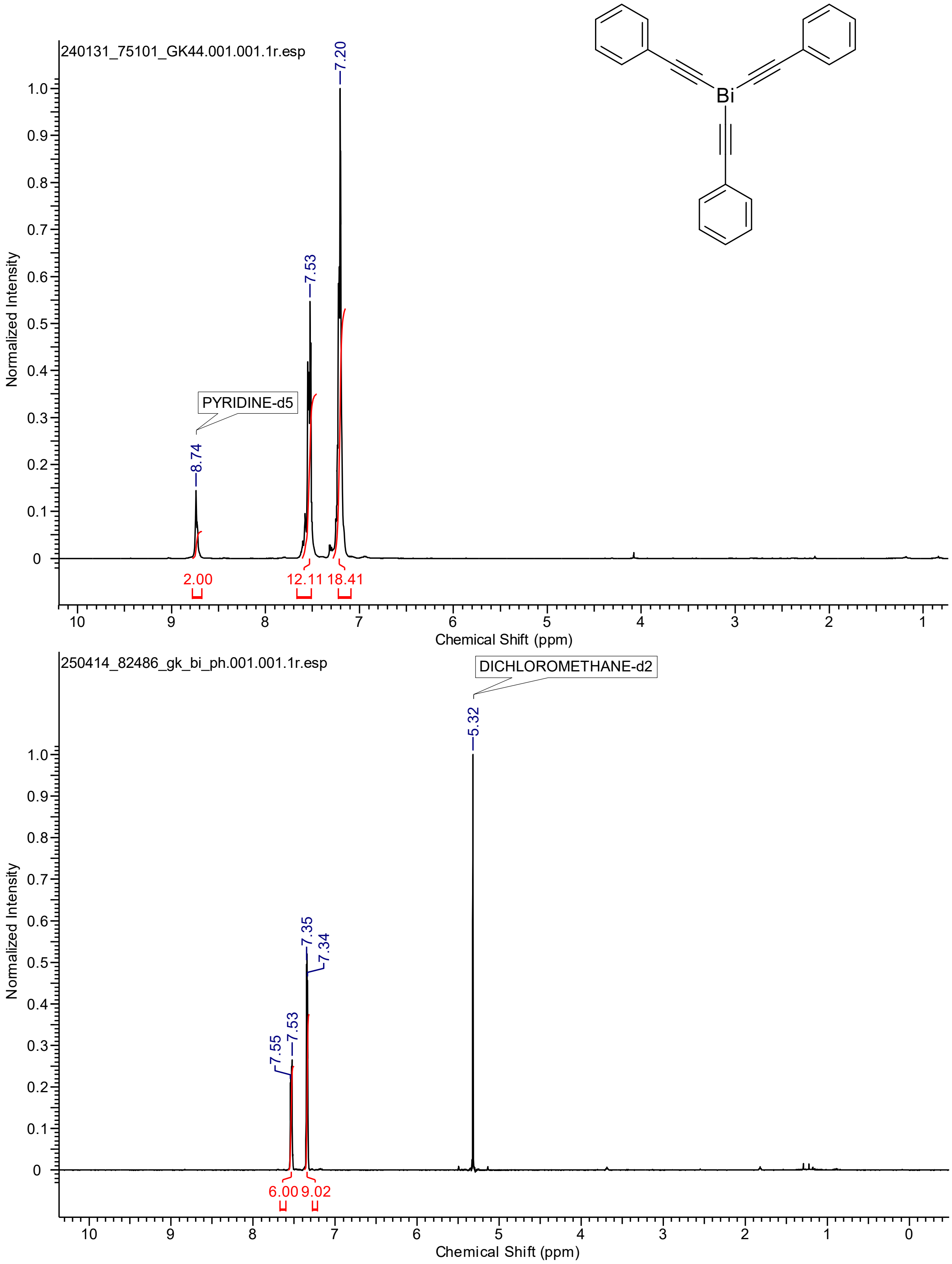

Figure S8. ^{1}H NMR spectrum of **4** in pyridine-d_5 (top) and CD_2Cl_2 (bottom). In the spectrum recorded in pyridine-d_5, the product signals overlap with those of the solvent pyridine-d_5.

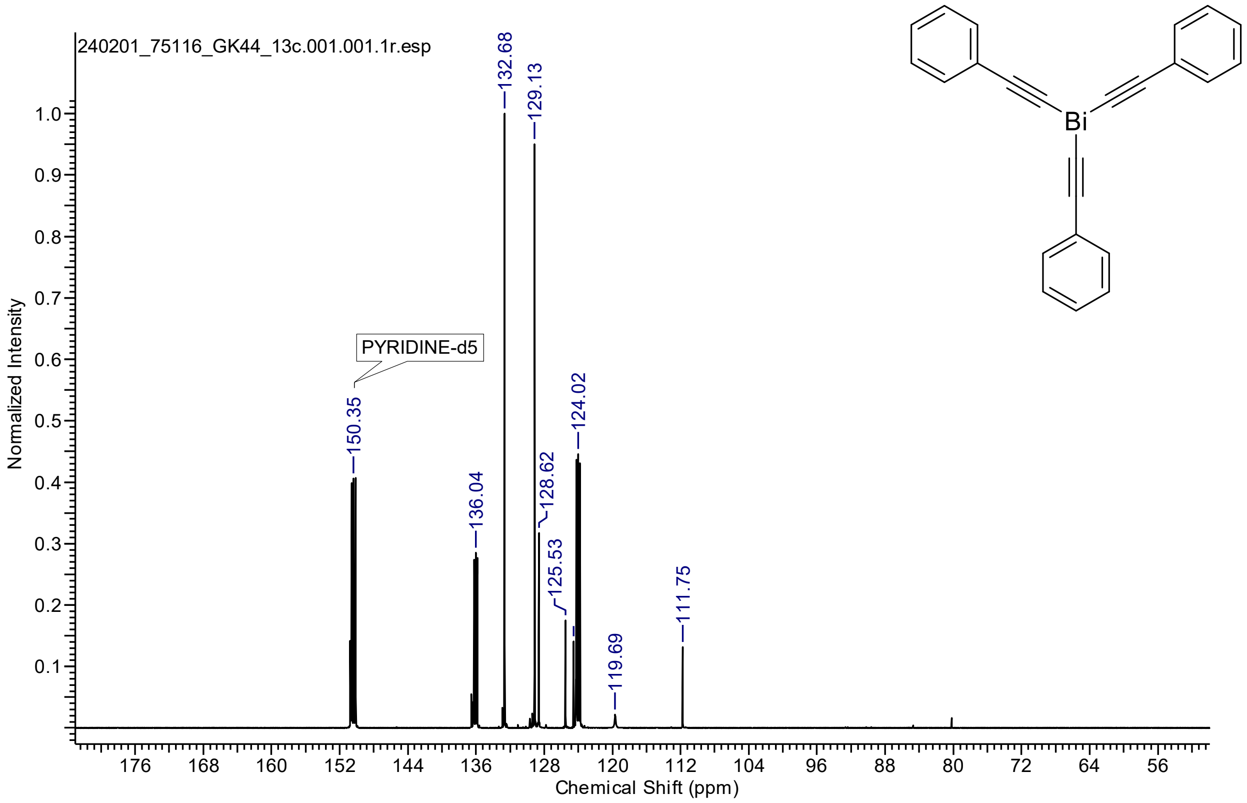

Figure S9. ^{13}C NMR spectrum of **4** in pyridine-d_5.

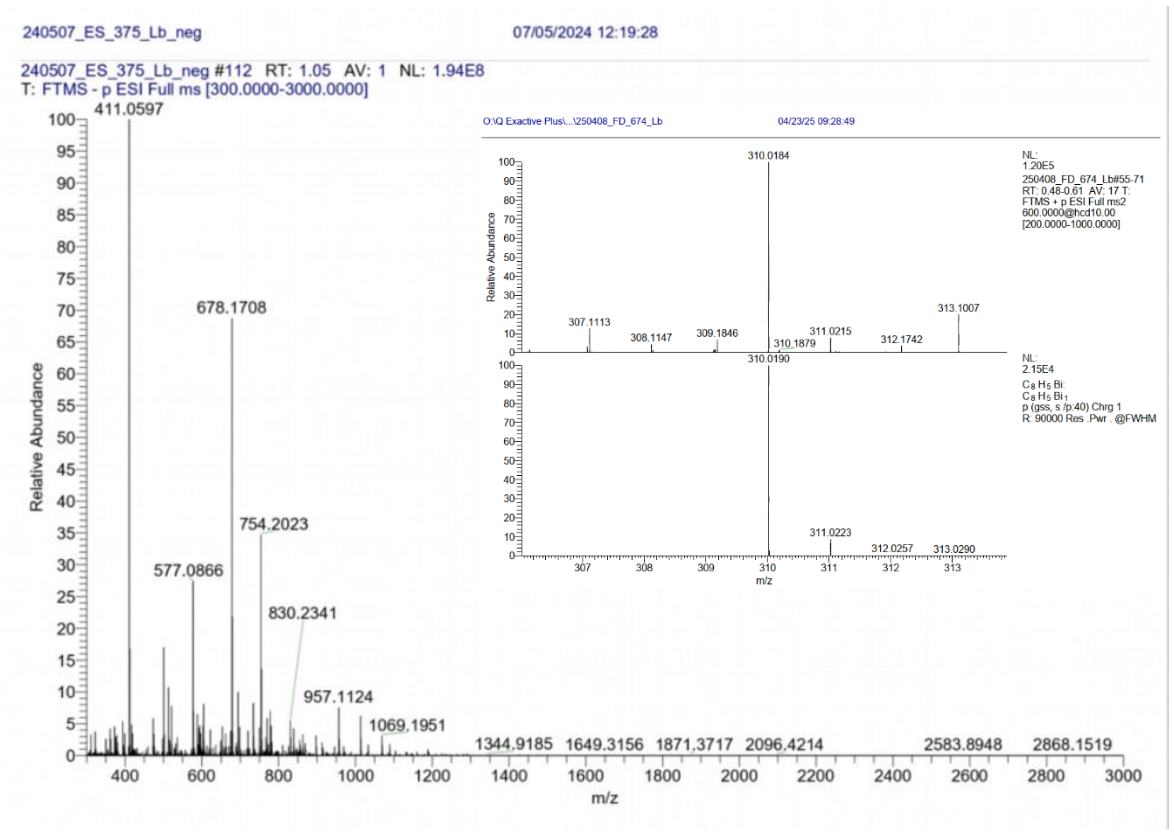

Figure S10. HR-MS spectrum of **4** and inlet showing signal for [BiC≡CPh]$^{•+}$.

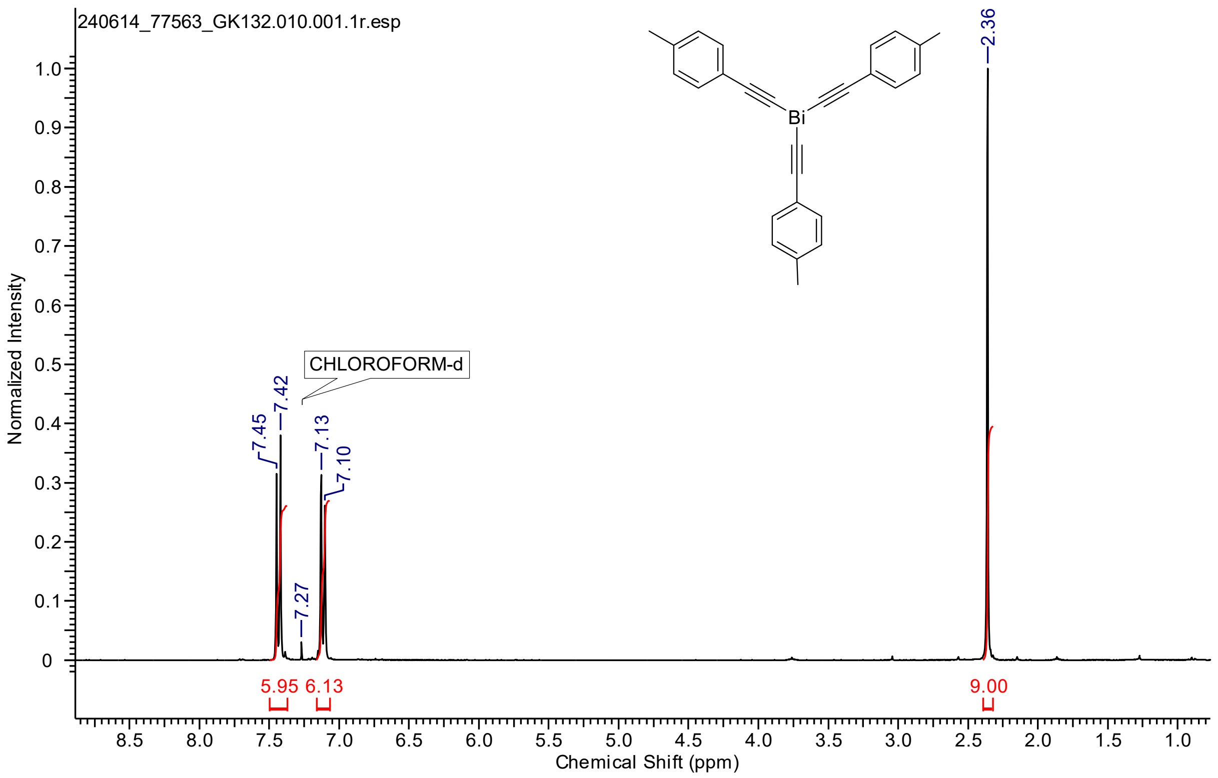

Figure S11. ^{1}H NMR spectrum of **5** in $CDCl_3$.

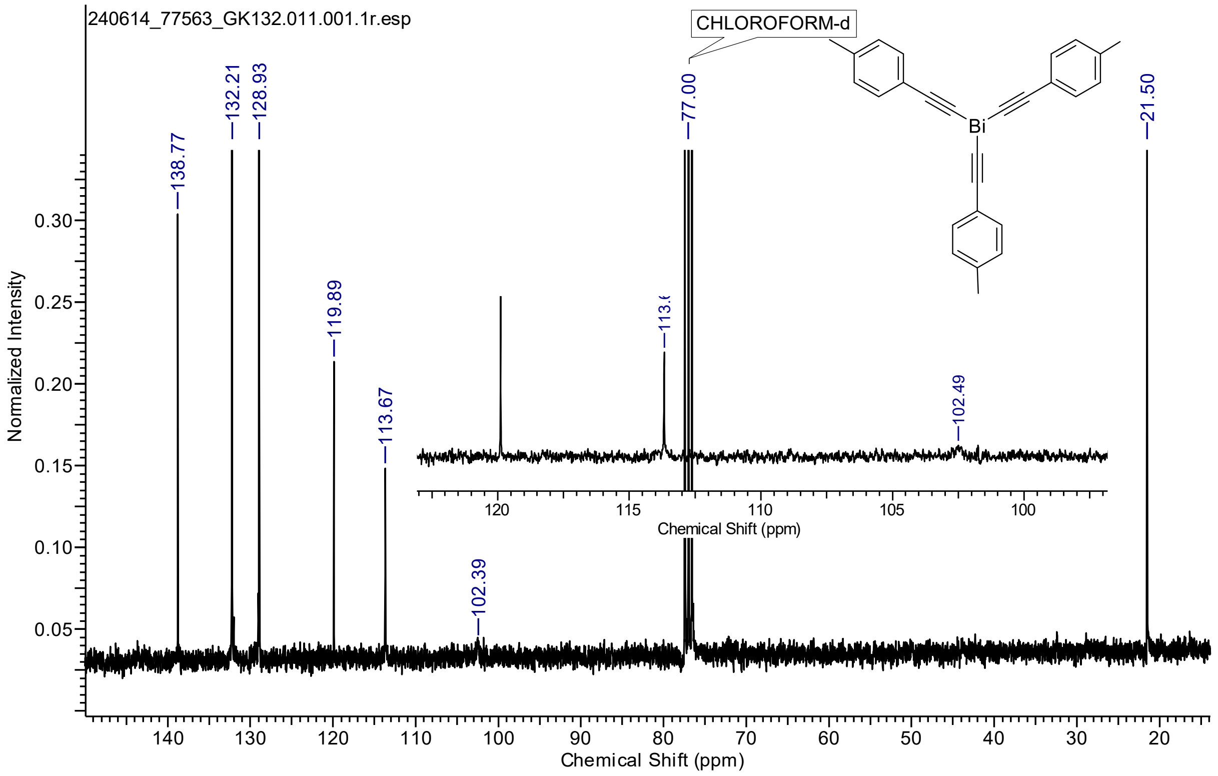

Figure S12. ^{13}C NMR spectrum of **5** in $CDCl_3$.

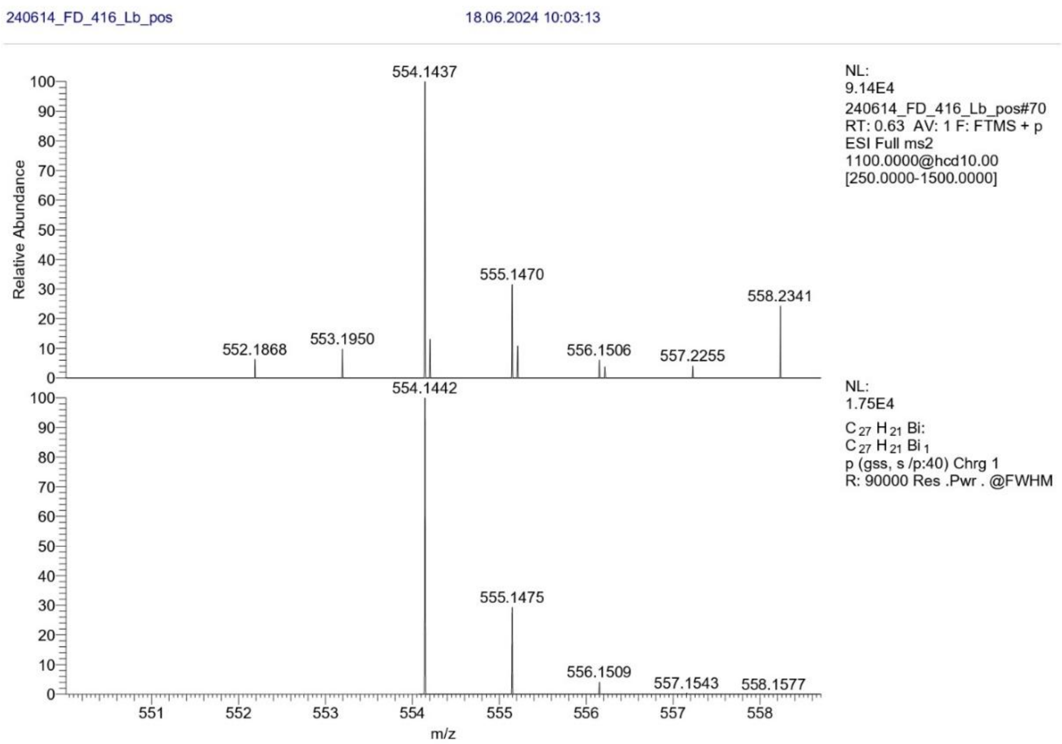

Figure S13. HR-MS spectrum of **5**.

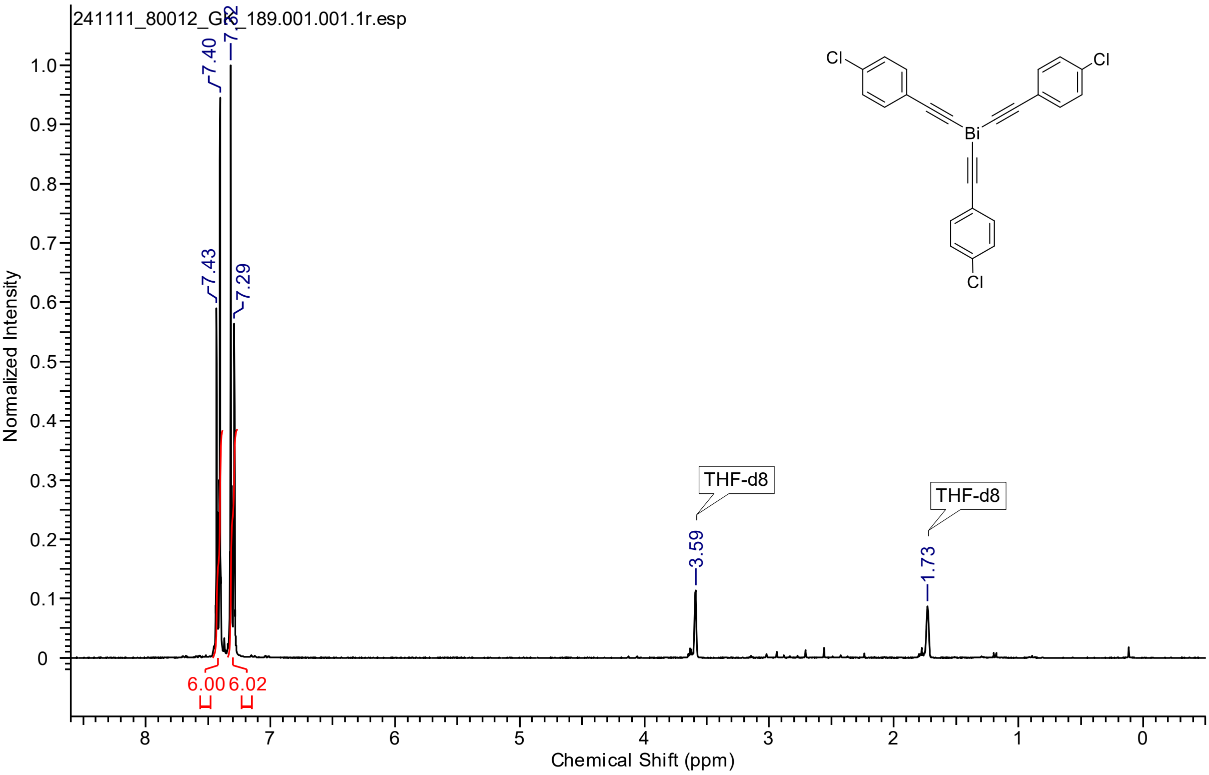

Figure S14. ^{1}H NMR spectrum of **6** in THF-d_8.

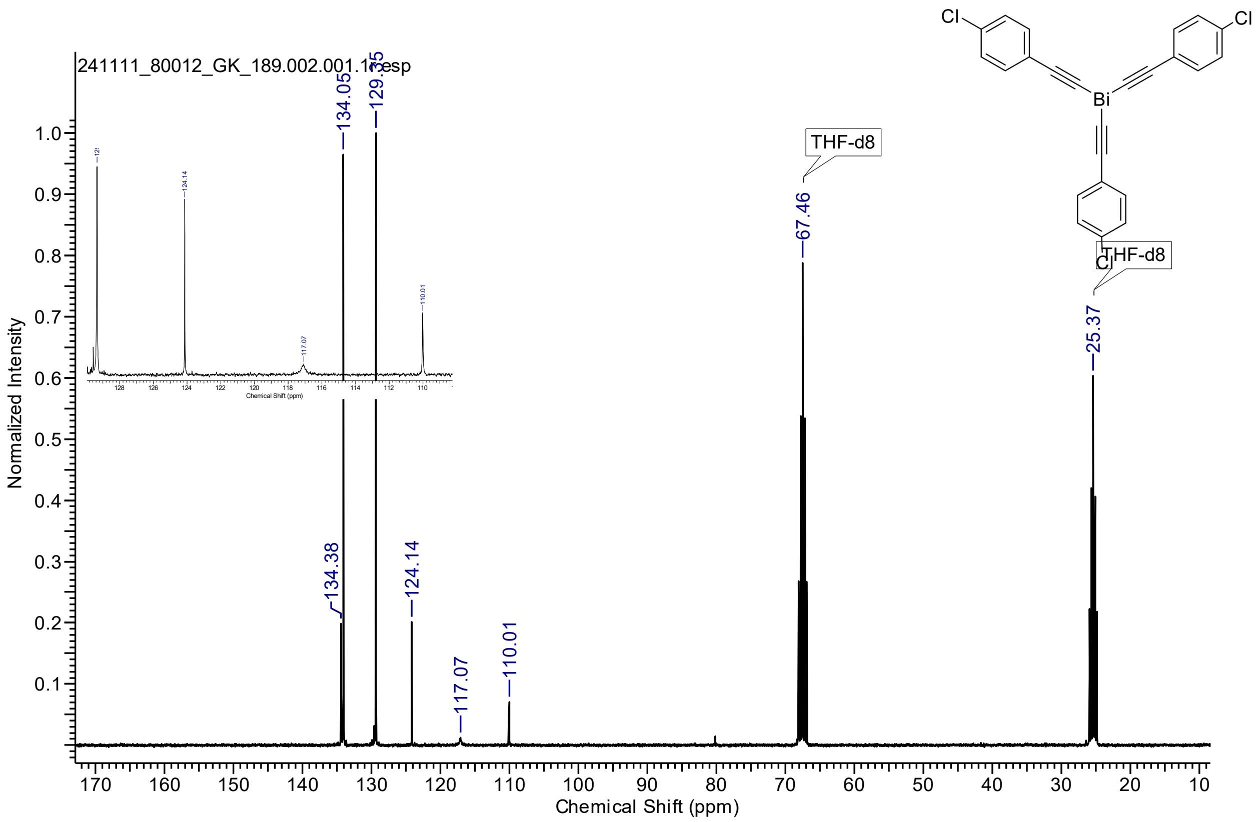

Figure S15. ^{13}C NMR spectrum of **6** in THF-d_8.

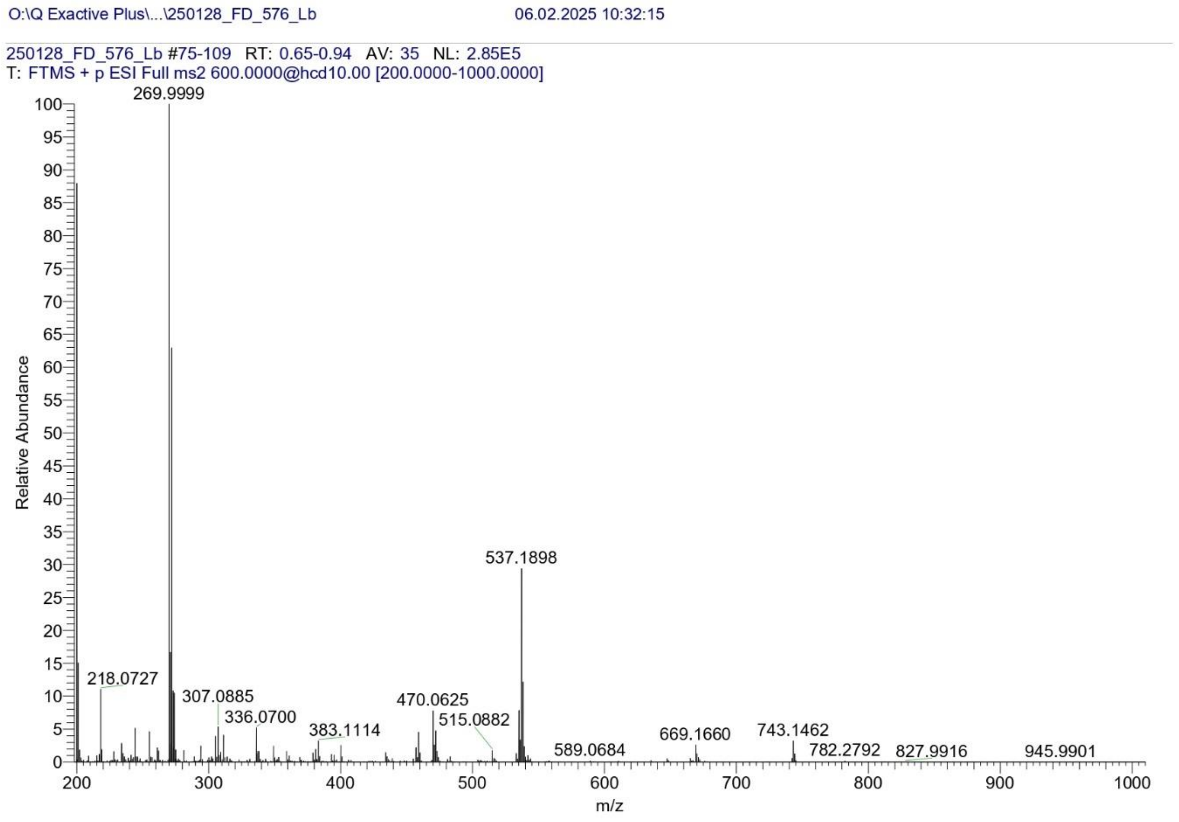

Figure S16. HR-MS spectrum of **6**.

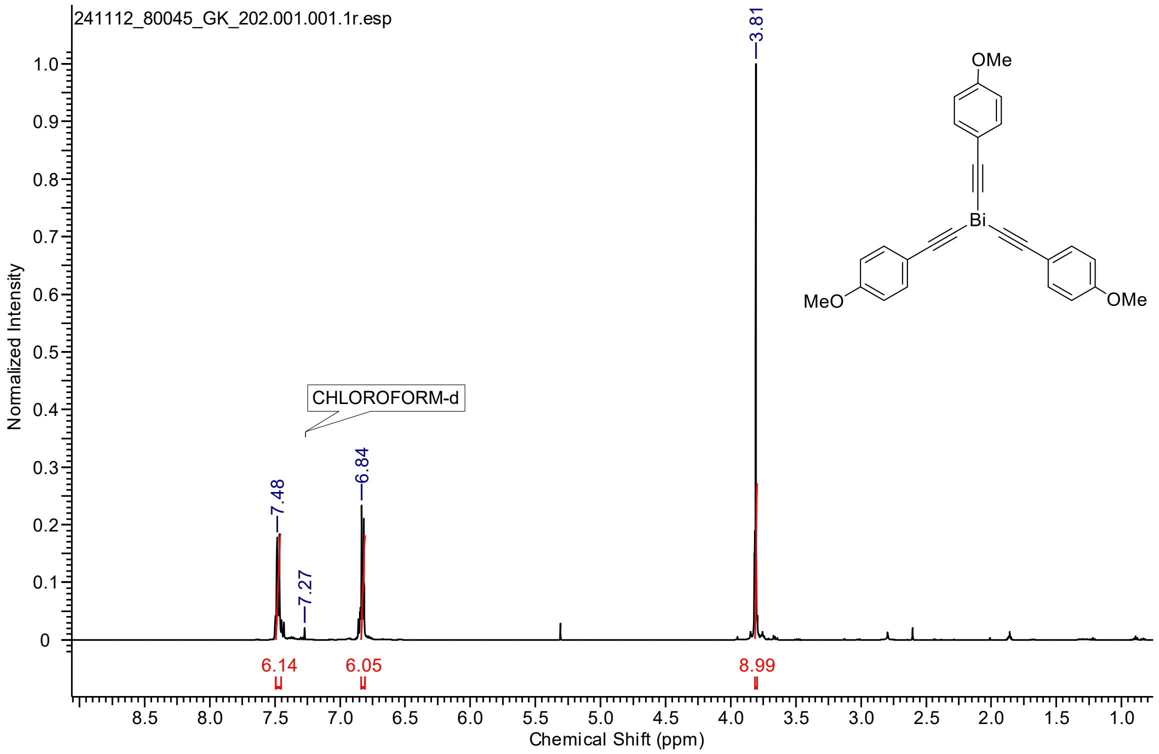

Figure S17. ^{1}H NMR spectrum of **7** in $CDCl_3$.

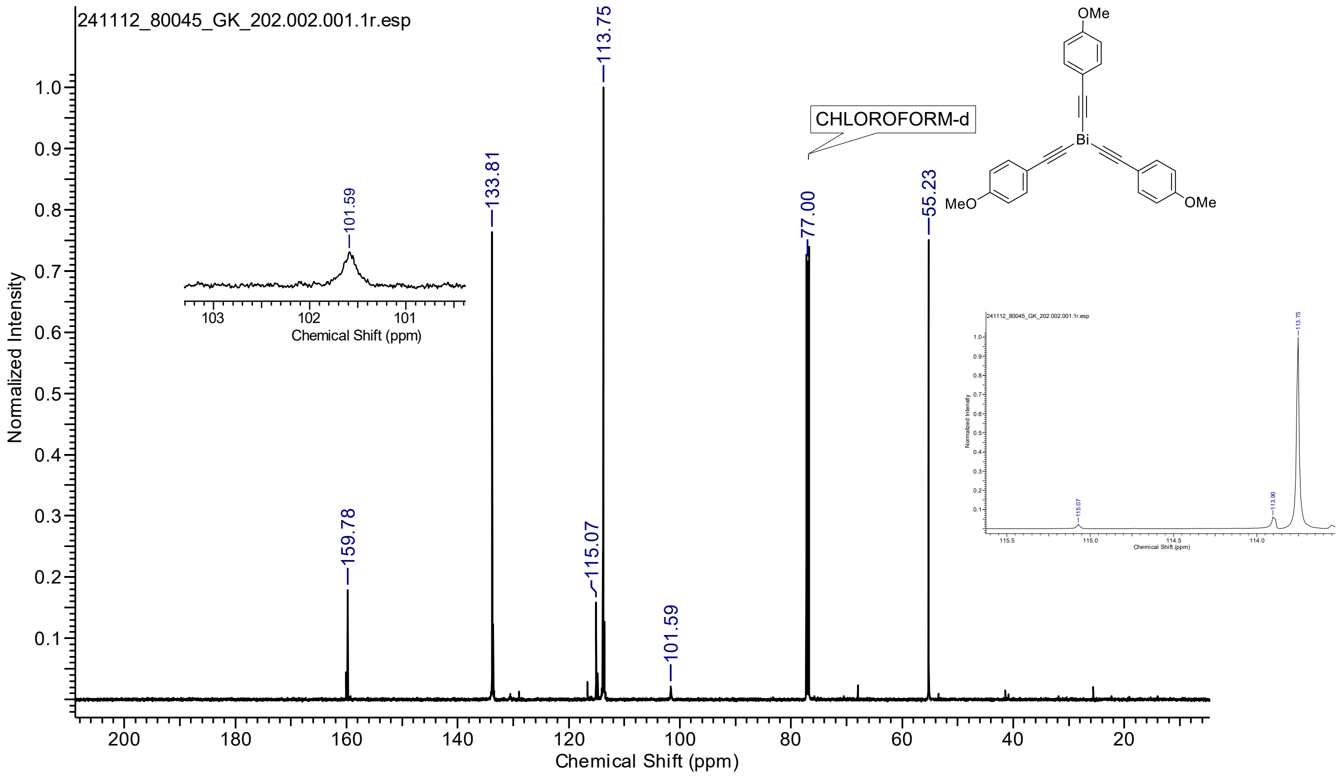

Figure S18. ^{13}C NMR spectrum of **7** in $CDCl_3$.

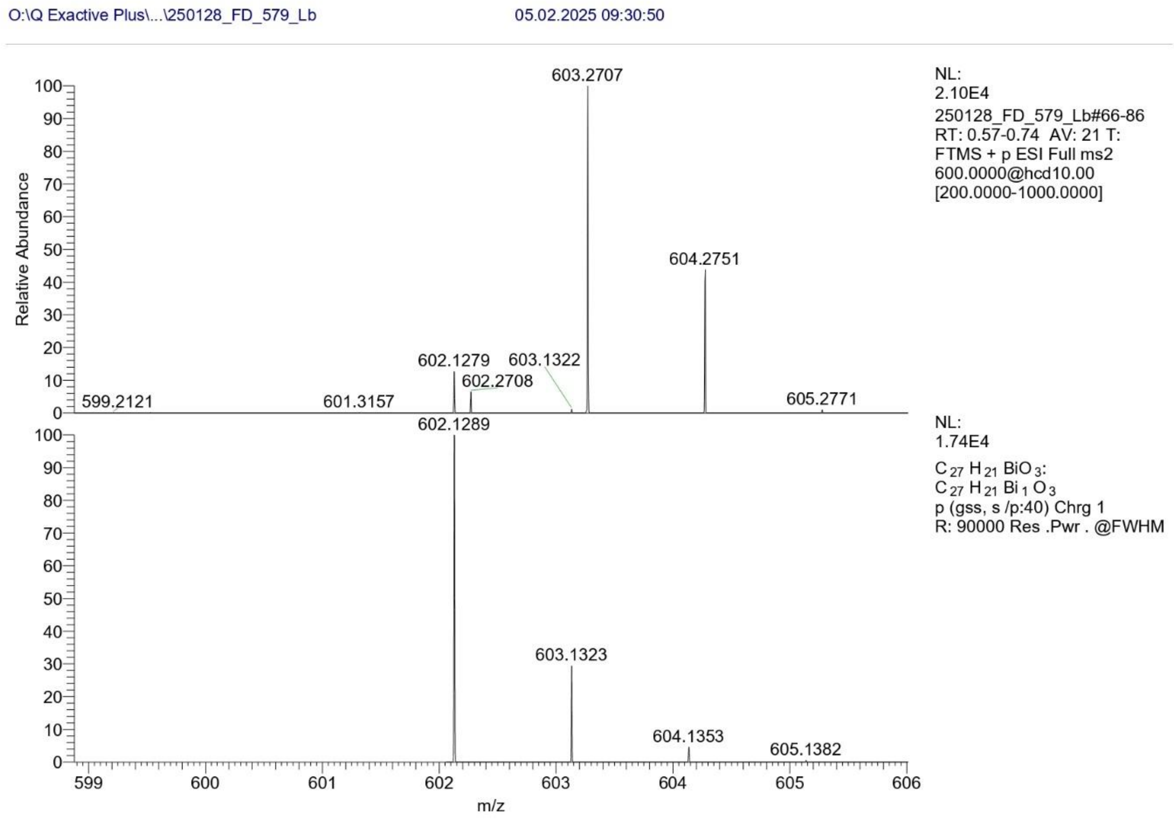

Figure S19. HR-MS spectrum of **7**.

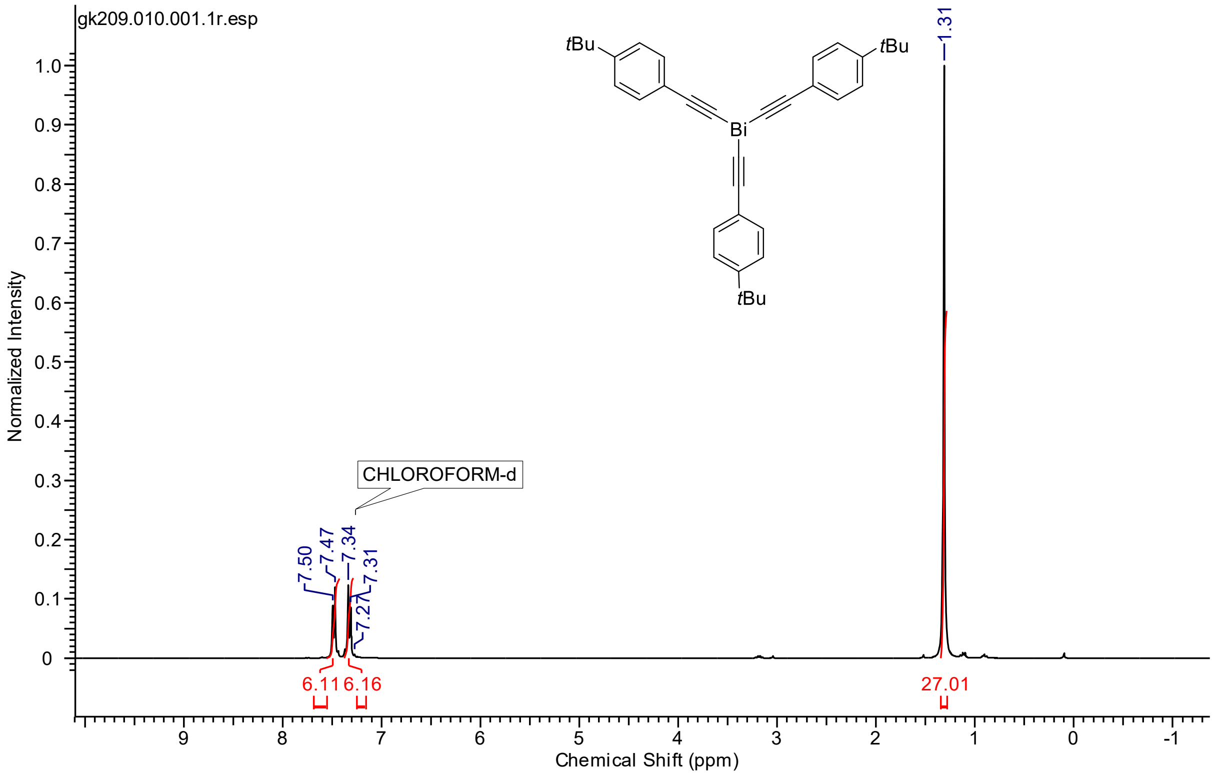

Figure S20. ^{1}H NMR spectrum of **8** in $CDCl_3$.

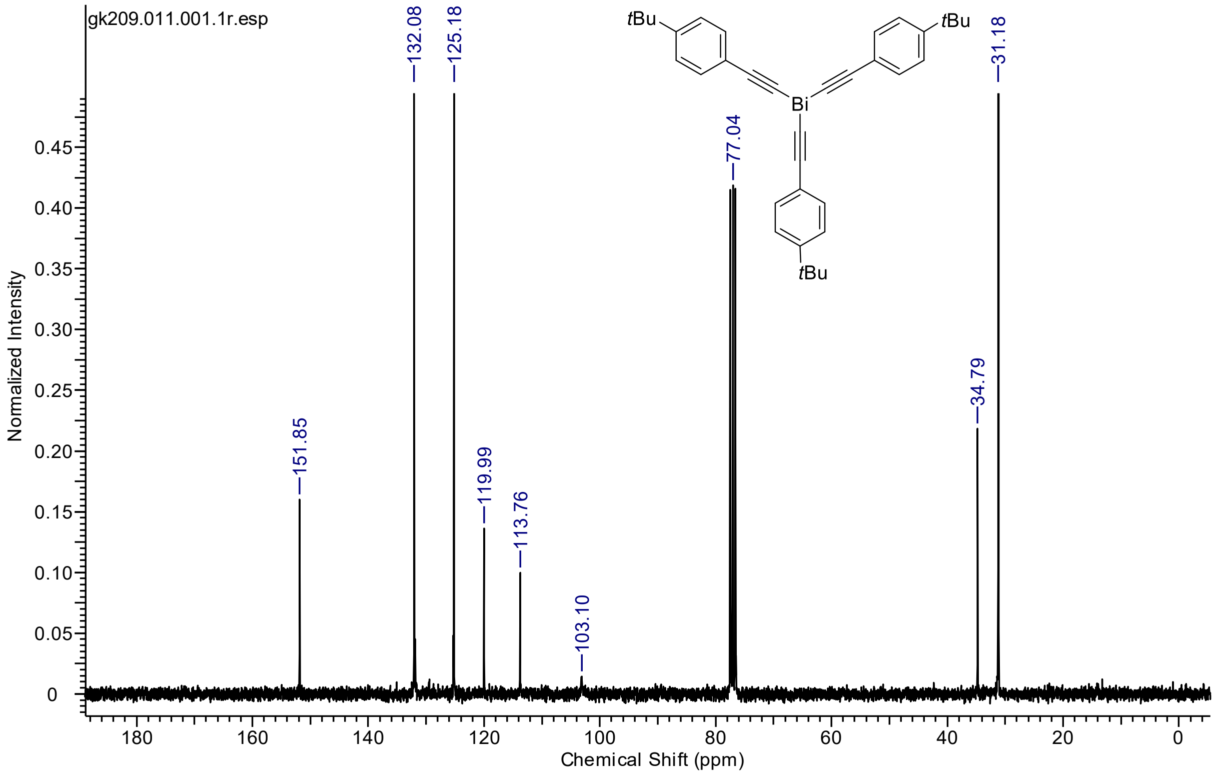

Figure S21. ^{13}C NMR spectrum of **8** in $CDCl_3$.

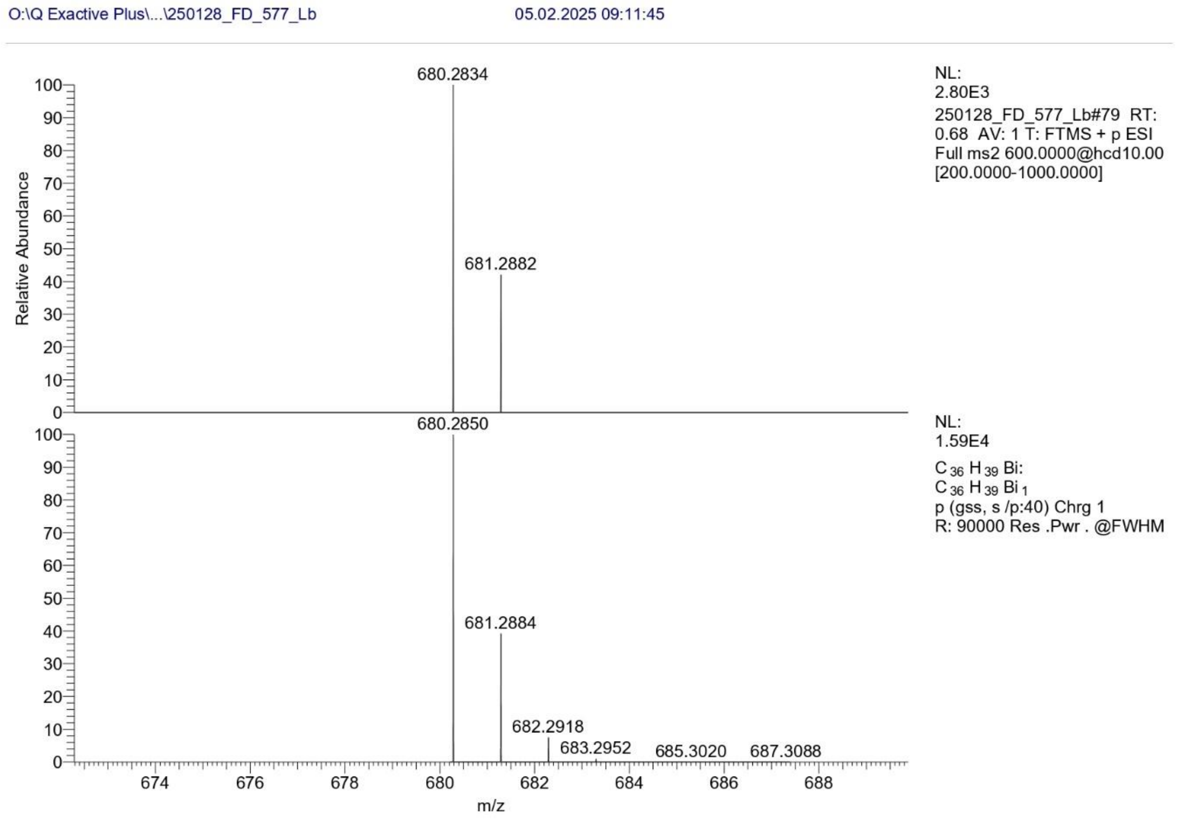

Figure S22. HR-MS spectrum of **8**.

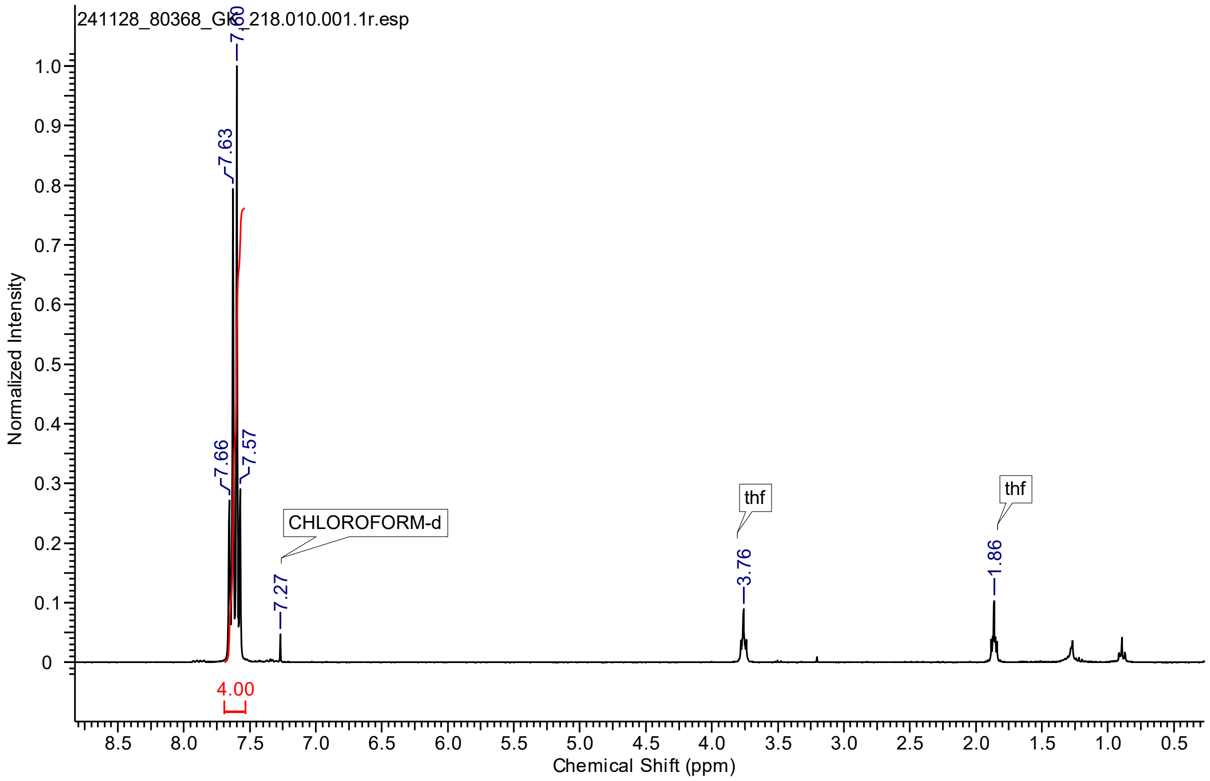

Figure S23. ^{1}H NMR spectrum of **9** inCDCl₃.

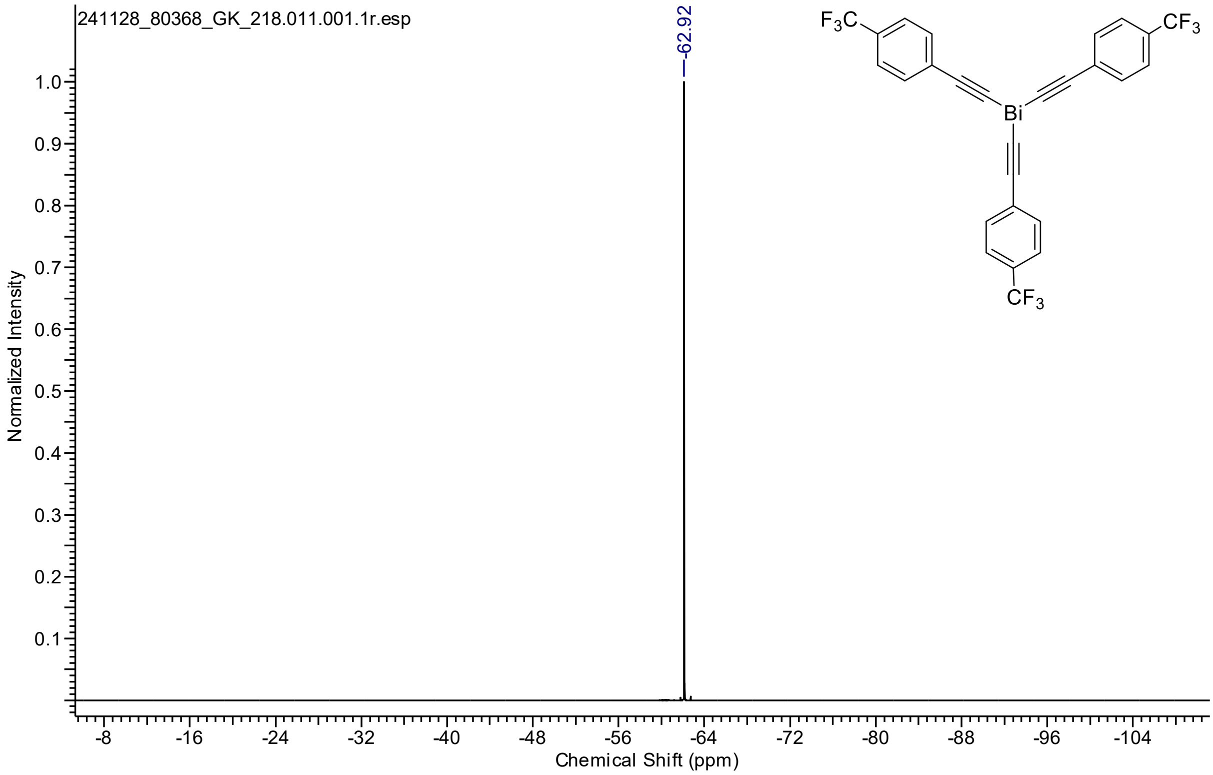

Figure S24. ^{19}F NMR spectrum of **9** inCDCl₃.

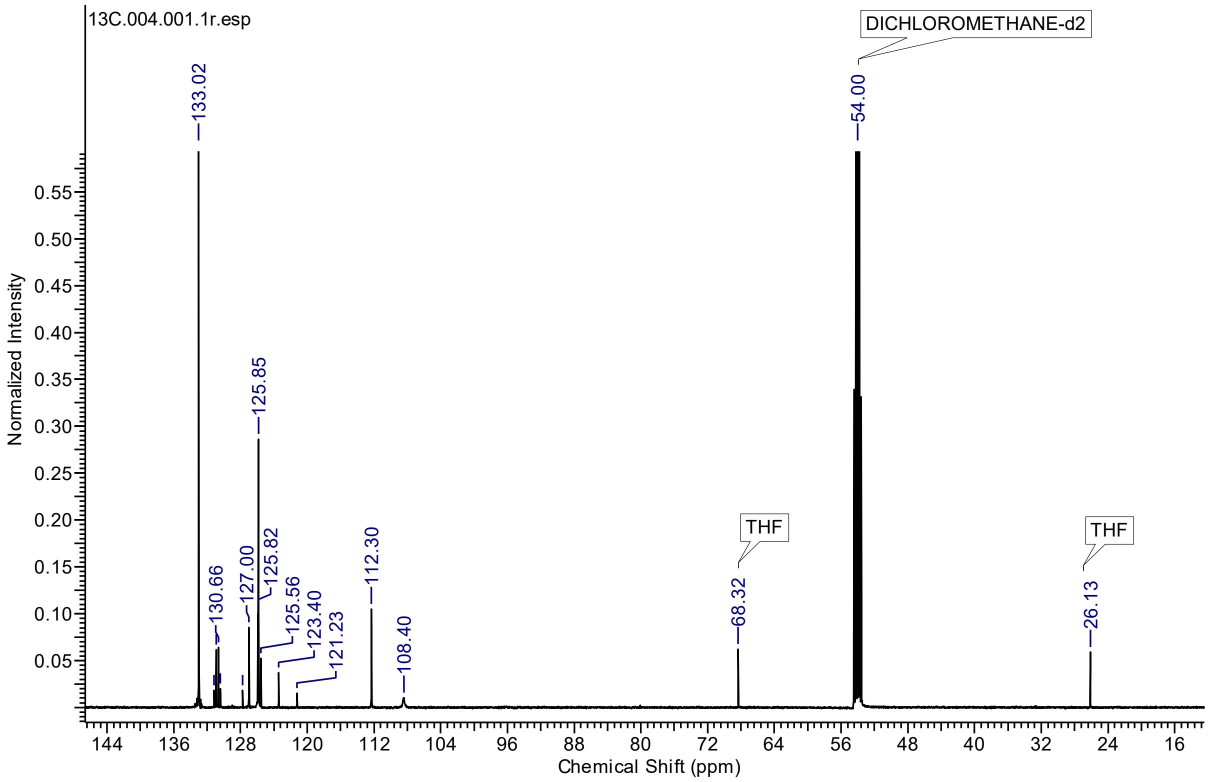

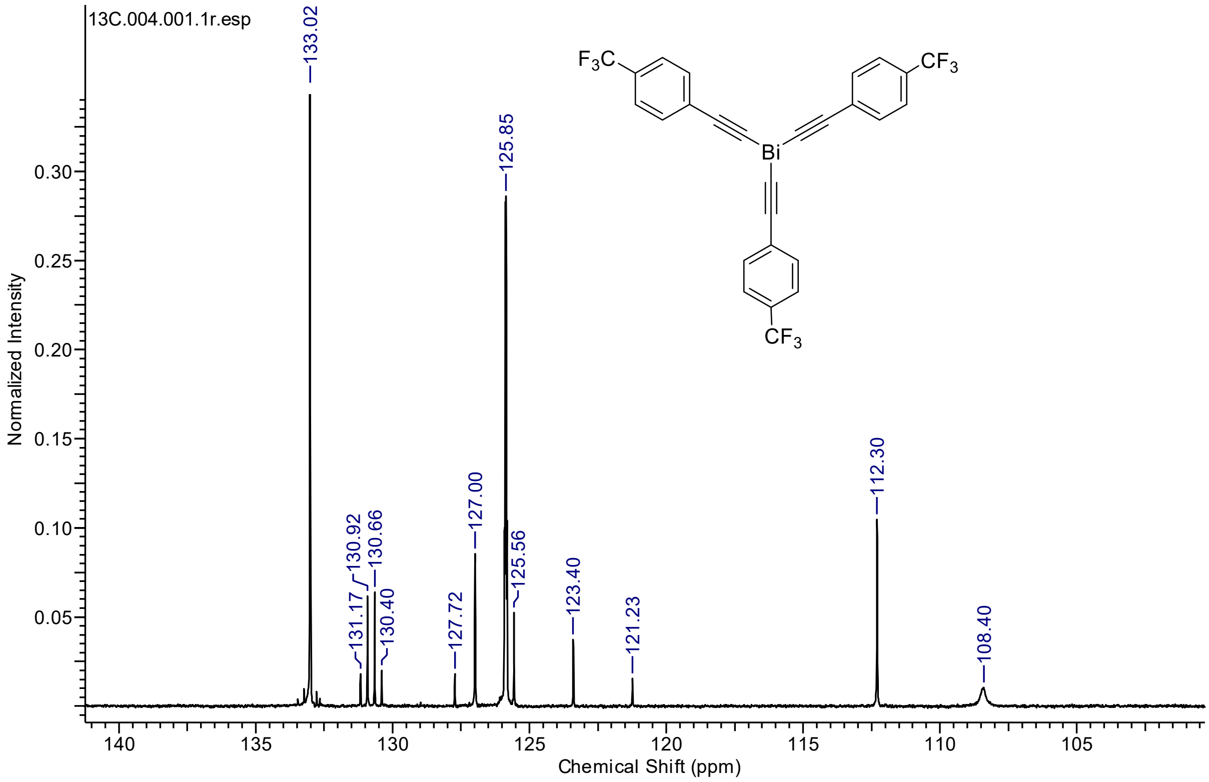

Figure S25. ^{13}C NMR spectrum of **9** in CD_2Cl_2.

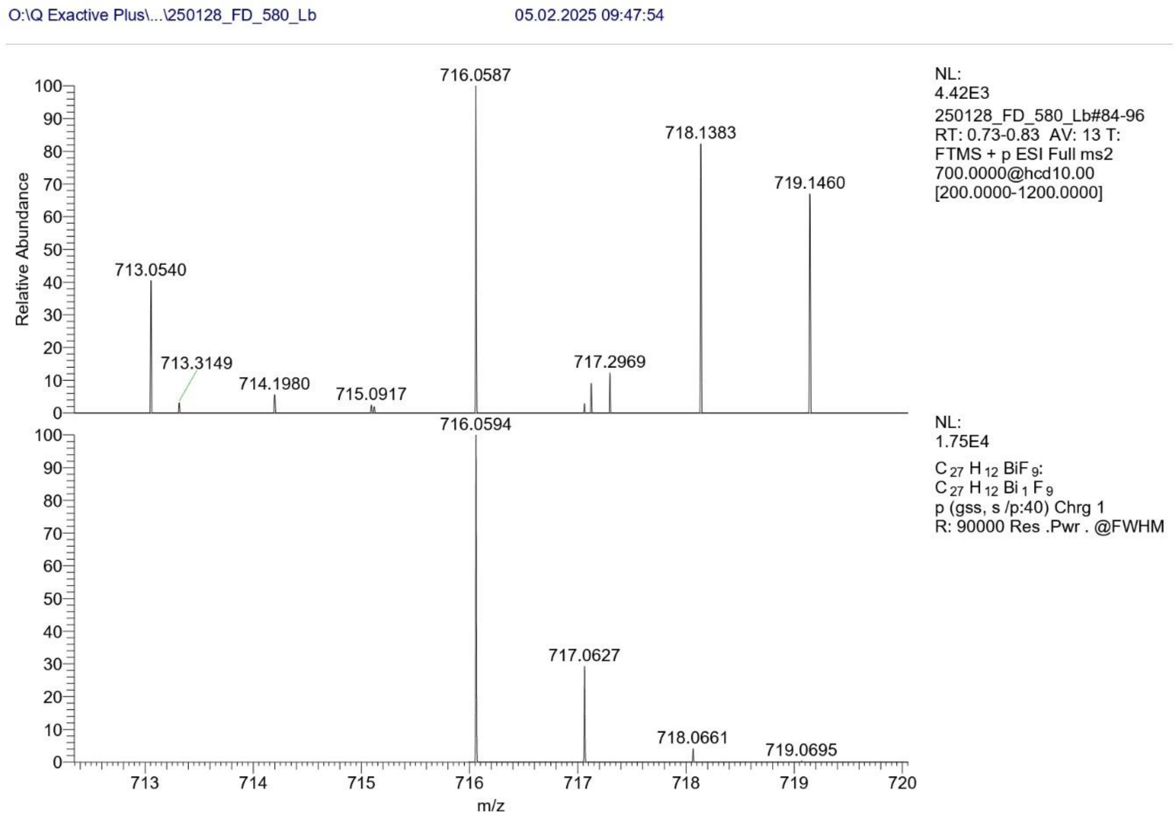

Figure S26. HR-MS spectrum of **9**.

4. Alkynyl radicals detection in EPR spectroscopic spin trap experiments

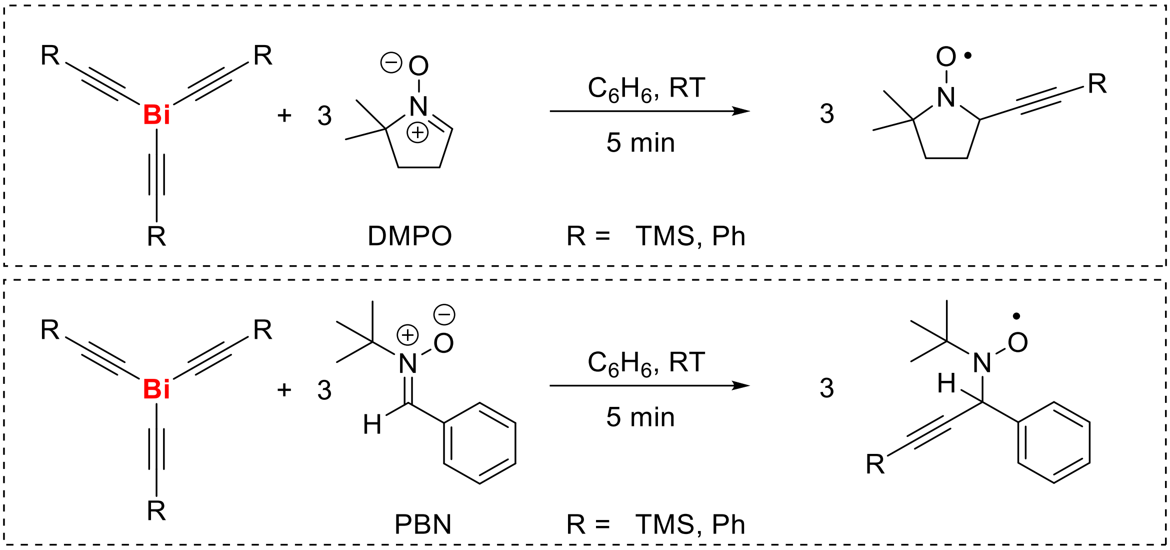

Scheme S1. Trapping of alkynyl radicals released from Bi(C≡CSiMe$_3$)$_3$ and Bi(C≡CPh)$_3$, using DMPO (dimethyl-1-pyrroline-*N*-oxide, top) and PBN (*N*-*tert*-butyl-α-phenyl-nitrone, bottom) as a spin trap.

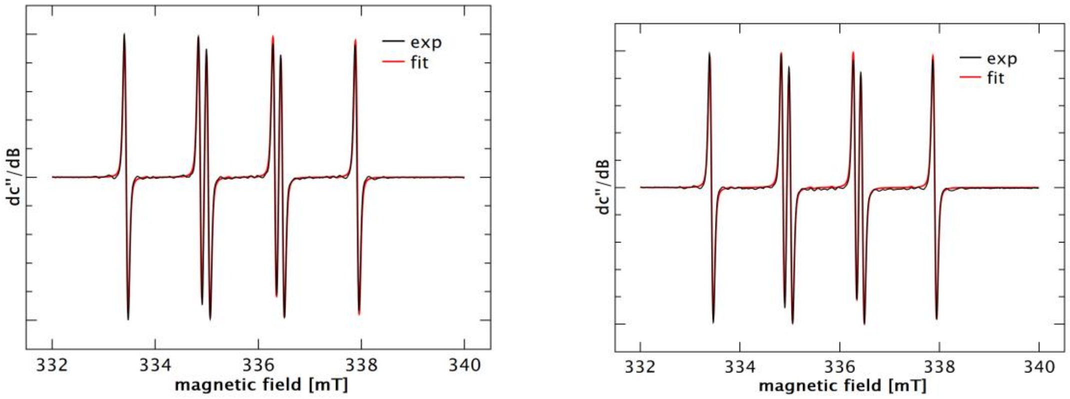

Figure S27. Left: Experimental (black) and simulated (red) continuous-wave (CW) X-band EPR spectra of a solution containing 1 equiv. [Bi(CCTMS)$_3$] (c = 2*10^{-5} mol/L) and 3 equiv. DMPO in benzene (0.5 mL). The observed resonance shows coupling constants of a(^{14}N) = 40.4 MHz (14.4 G, 1.44 mT), a(^{1}H) = 44.7 MHz (15.9 G, 1.59 mT) and a g_{iso} value of 2.0052. Spectrometer settings: microwave frequency = 9.421558 GHz, 0.05 mT modulation amplitude at 100 kHz, microwave power = 1 mW, number of accumulated scans = 1, conversion time = 2 ms. **Right:** Experimental (black) and simulated (red) continuous-wave (CW) X-band EPR spectra of a solution containing 1 equiv. [Bi(CCPh)$_3$] (c = 2*10^{-5} mol/L) and 3 equiv. DMPO in benzene (0.5 mL). The observed resonance shows coupling constants of a(^{14}N) = 40.3 MHz (14.4 G, 1.44 mT), a(^{1}H) = 44.6 MHz (15.9 G, 1.59 mT) and a g_{iso} value of 2.0052. Spectrometer settings: microwave frequency = 9.421253 GHz, 0.05 mT modulation amplitude at 100 kHz, microwave power = 1 mW, number of accumulated scans = 1, conversion time = 2 ms.

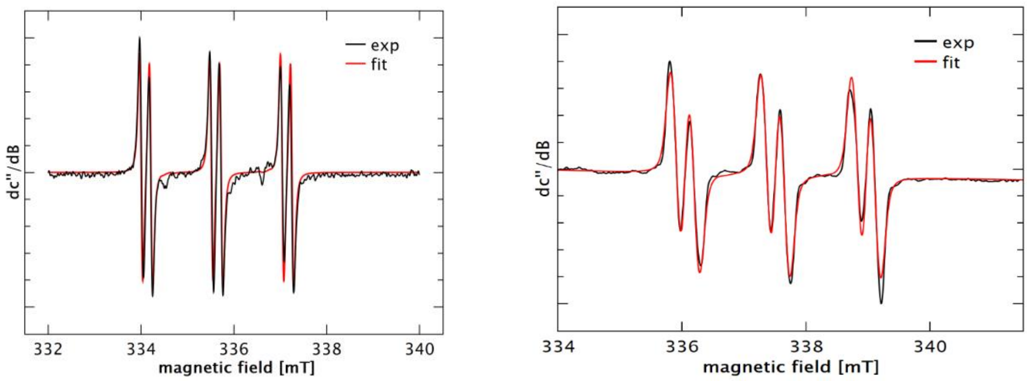

Figure S28. Left: Experimental (black) and simulated (red) continuous-wave (CW) X-band EPR spectra of a solution containing 1 equiv. [Bi(C≡CSiMe$_3$)$_3$] (c = 2*10^{-5} mol/L) and 3 equiv. PBN in benzene (0.5 mL). The observed resonance shows coupling constants of a(^{14}N) = 42.5 MHz (15.1 G, 1.51 mT), a(^{1}H) = 6.04 MHz (2.15 G, 0.215 mT) and a g_{iso} value of 2.0053. Spectrometer settings: microwave frequency = 9.420920 GHz, 0.05 mT modulation amplitude at 100 kHz, microwave power = 0.1 mW, number of accumulated scans = 1, conversion time = 2 ms. **Right:** Experimental (black) and simulated (red) continuous-wave (CW) X-band EPR spectra of a solution containing 1 equiv. Bi(C≡CPh)$_3$ (c = 2·10–2 mol/L) and 3 equiv. PBN in THF. The observed resonance shows coupling constants of a(1 × ^{14}N) = 40.8 MHz (14.6 G, 1.46 mT), a(1 × ^{1}H) = 7.91 MHz (2.82 G, 0.282 mT), and a giso value of 2.0051. Contributions to the spectrum line-width were modelled with unresolved coupling to the protons of the phenyl group with a(2 × ^{1}H) = 0.885 MHz (0.315 G, 0.0315 mT), a(2 × ^{1}H) = 0.270 MHz (0.0962 G, 0.00962 mT), a(1 × ^{1}H) = 1.80 MHz (0.641 G, 0.0641 mT), Spectrometer settings: microwave frequency = 9.473234 GHz, 0.2 mT modulation amplitude at 100 kHz, microwave power = 10 mW, number of accumulated scans = 10, conversion time = 2 ms.

Inverted spin trapping and a Forrester-Hepburn mechanism have been reported to be potential scenarios when using spin traps.[95] In inverted spin trapping, the spin trap (here: PBN or DMPO) would transfer an electron to the substrate (here: an alkynyl bismuth compound **2-9**) and then accept an anion (in this case an alkynyl anion) from the radical anion intermediate. The anodic peak potentials of PBN and DMPO have been reported to be +1.47 V and +1.63 V vs SCE, respectively,[96] which translates into +1.09 V (PBN) and +1.25 (DMPO) vs Fc/Fc$^+$.[97] We have thus studied the electrochemical behavior of the most electron-deficient alkynyl bismuth compound of the series presented in this work, Bi(C≡C-*p*-$CF_3C_6H_4)_3$ (**9**). A cathodic peak potential of –2.02 V was detected (see Chart S1a). Thus, the inverted spin trapping scenario can be ruled out for compounds **2-9** and the spin traps PBN and DMPO.

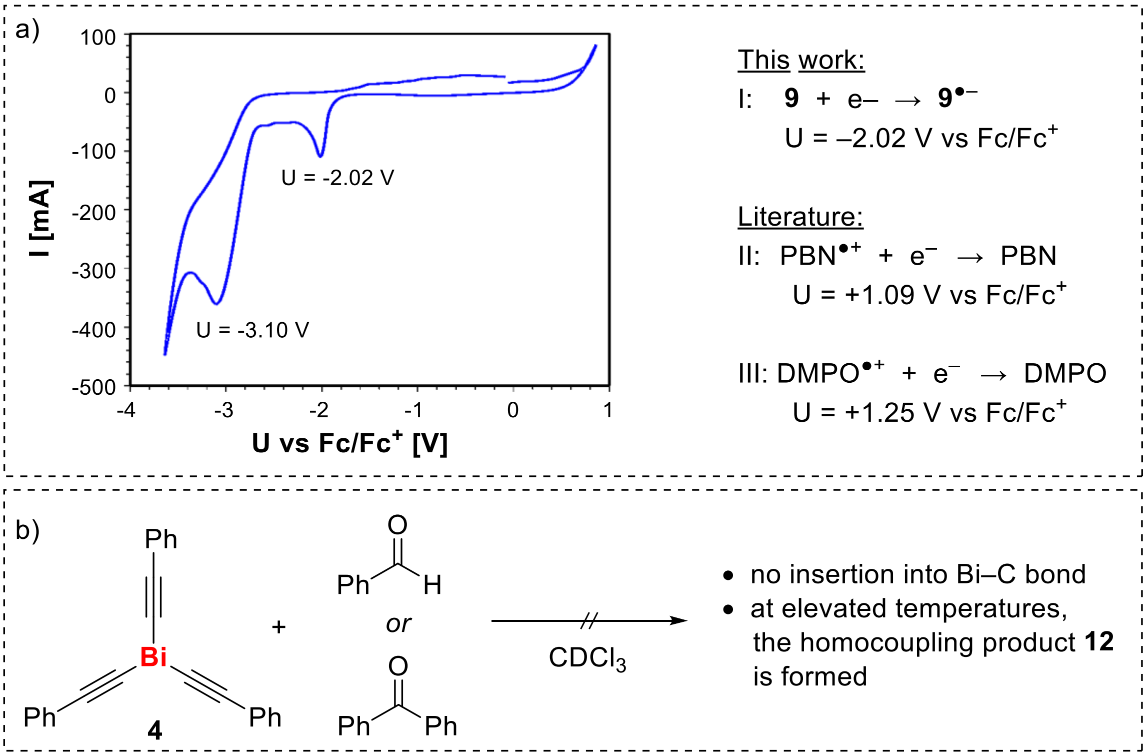

Chart S1. a) Cyclic voltammogram of Bi(C≡C-*p*-$CF_3C_6H_4)_3$ (**9**) in THF (0.1 M $[N(nBu)_4][PF_6]$) at room temperature (scan rate = 250 mV/s), referenced versus Fc/Fc$^+$ (ferrocene/ferrocenium) and literature values for the oxidation of PBN and DMPO. b) Attempted reaction of **4** with benzaldehyde and benzophenone (in two separate reactions; conditions: see text).

For the Forrester-Hepburn mechanism, an anionic ligand (here: an alkynyl anion) would be added to the spin trap. In order to evaluate the feasibility of an alkynyl anion transfer from **2-9** to an electrophile, two separate reactions of **4** (20.0 mg, 0.04 mmol) with model substrates benzophenone (7.1 mg, 0.04 mmol) and benzaldehyde (4.1 mg, 0.04 mmol) in $CDCl_3$ (0.6 mL) were performed. In both cases, no reaction was observed at room temperature. The sample containing the more reactive model substrate benzaldehyde was then heated to 60 °C, but only the formation of the Glaser-type C–C homocoupling product **12** was detected, while the aldehyde remained unreacted detected (see Chart S1b). This demonstrates that compounds **2-9** are poor alkynyl anion transfer reagents, making the Hepburn mechanism very unlikely to be relevant for the detection of radical species in reactions of **2-9** with the spin traps PBN and DMPO.

It can be concluded that radical transfer from **2** and **4** to PBN and DMPO, respectively, is responsible for the formation of the radical species detected in these experiments.

5. Synthesis and characterization of C–C homocoupling products 10-17

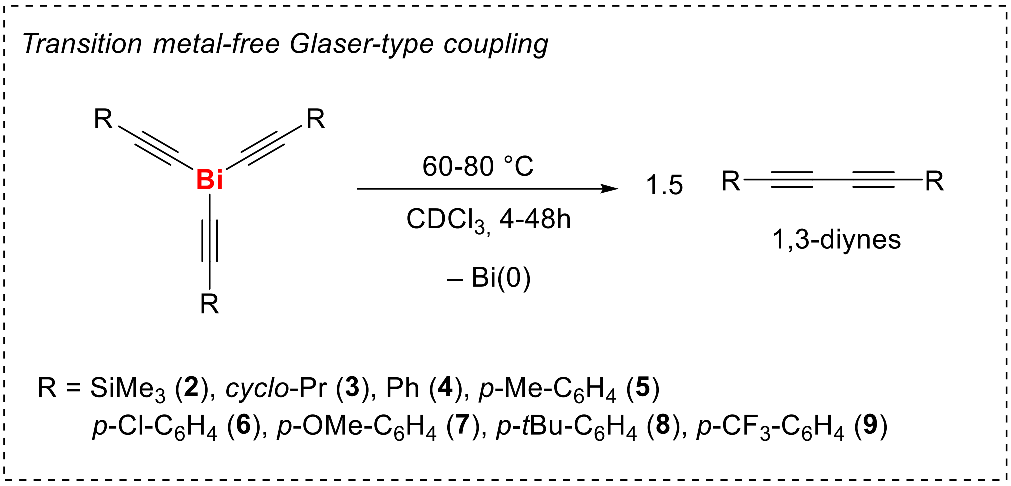

Scheme S2. Synthesis of the C–C homocoupling products **10-17**.

Depending on the substituent at the alkyne moiety and the reaction temperature, the bismuth alkynes are not stable in solution and selectively react to give the corresponding 1,3-diynes and Bi(0). In this work we have heated the compounds **2-9** to form the respective 1,3-diynes (**10-17**) via C–C homocoupling of the alkynyl radicals. In Table S2, the temperature and time needed for the complete conversion of the starting materials and the yield of the products are summarized.

General procedure: The bismuth alkynes (typically 0.05 mmol) are heated in a J.-Young NMR tube using $CDCl_3$ (typically 0.5 mL) as the solvent. After the mentioned time the solution was filtered using a fiberglass filter to remove the black Bi(0) precipitate and dried to give the solid (or liquid in the case of **11**) product as a pure 1,3-diyne. The products (**10-17**) were characterized by NMR spectroscopy (^{1}H, ^{13}C, and ^{19}F, ^{29}Si where applicable) and HR-MS spectrometry. All the NMR data is in good agreement with the literature reports.[15,58,59]

In principle, a photochemical initiation of the C–C homocoupling reaction is also possible. For instance, irradiating a solution of **4** (typically 0.05 mmol) in $CDCl_3$ (typically 0.5 mL) with a blue LED (λ = 460 nm) gave the coupling product **12** in quantitative spectroscopic yield after 18 h according to NMR spectroscopic analyses.

Table S2. Synthesis of symmetrical 1,3-diynes.

**1,3-Diyne** (compound number)	**NMR conversion**	**Isolated Yield**	**Time, Temperature**	**Solvent**
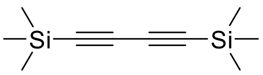 **10**	100%	98%	15 h, 70 °C	$CDCl_3$
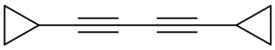 **11**	100%	70%	24 h, 65 °C	$CDCl_3$
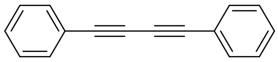 **12**	100%	95%	16 h, 60 °C	$CDCl_3$[a]
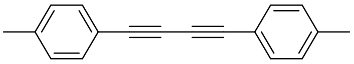 **13**	100%	98%	2 d, 80 °C	$CDCl_3$ / THF
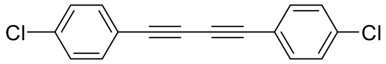 **14**	100%	96%	2 d, 80 °C	$CDCl_3$ / THF
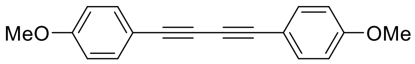 **15**	100%	99%	12 h, 60 °C	$CDCl_3$
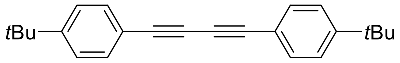 **16**	100%	88%	24 h, 70 °C	$CDCl_3$
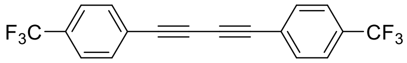 **17**	100%	98%	24 h, 80 °C	$CDCl_3$ / THF

[a]: Compound **4** is only moderately soluble in $CDCl_3$; the reaction was also performed in C_5D_5N, where quantitative formation of the 1,3-diyne was observed. In order to obtain maximum yields of the coupling product **12**, residual amounts of weakly bound THF molecules (*cf.* XRD analysis) should be removed from the starting material **4**, as α-alkynylation of THF can be observed as a minor competing reaction pathway.

In addition, a comparative study to gain insight into the stability of the bismuth complexes Bi(C≡CR)$_3$ (**2-9**) under inert conditions at elevated temperature and their reactivity to form 1,3-diynes has been performed. Thus, in Table S3 the ratio of Bi(C≡CR)$_3$: product determined by NMR spectroscopy after heating a solution of each compound Bi(C≡CR)$_3$ in $CDCl_3$ for 8 h is given. Compounds **5** (R = *p*-Me-C_6H_4), **6** (R= *p*-Cl-C_6H_4), and **9** (*p*-CF_3-C_6H_4) show no or only minor conversion when heated to 60 °C in $CDCl_3$ and were thus heated to 80 °C in $CDCl_3$ for 16 h. Under these conditions, the conversion to 1,3-diynes followed the order **9**>**6**>**5** (Table S4).

Table S3. Stability of the bismuth alkynes (**2**, **3**, **4**, **7**, and **8**).

**Bismuth alkyne**	**1,3-Diynes** (compound number)	**NMR Conversion Reactant: Product**	**Time, Temperature**	**solvent**
**2**	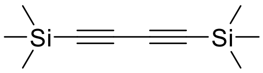 **10**	1.0 : 0<br>1.0 : 0.10	8 h, 60 °C<br>24 h, 60 °C	$CDCl_3$
**3**	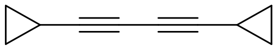 **11**	0.80 : 1.0	8 h, 60 °C	$CDCl_3$
**4**	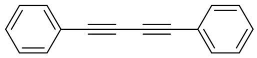 **12**	0.67 : 1.0[a]	8h, 60 °C	Pyridine
**7**	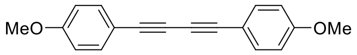 **15**	0.25:1.0	8 h, 60 °C	$CDCl_3$
**8**	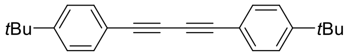 **16**	0.10:1.0	8 h, 60 °C	$CDCl_3$

[a]: Compound **4** is only moderately soluble in $CDCl_3$; the reaction was also performed in C_5D_5N, where partial signal overlap was observed. An approximated ratio of 0.67:1 (reactant : product) was deduced.

When compound **9** is heated with one equivalent of 6-iodo-1-hexene in $CDCl_3$ for 50 h, the homocoupling product **17** is the main product (49%) according to ^{1}H NMR spectroscopic analysis, but a decrease of the amount of olefin in the sample by 15% is also detected. This is in agreement with the formation of the radical $((CH_2)_4CH=CH_2)^{\bullet}$, which readily undergoes 5-exo-trig-cyclization to form a compound with a cyclopentyl structural motif.[31]

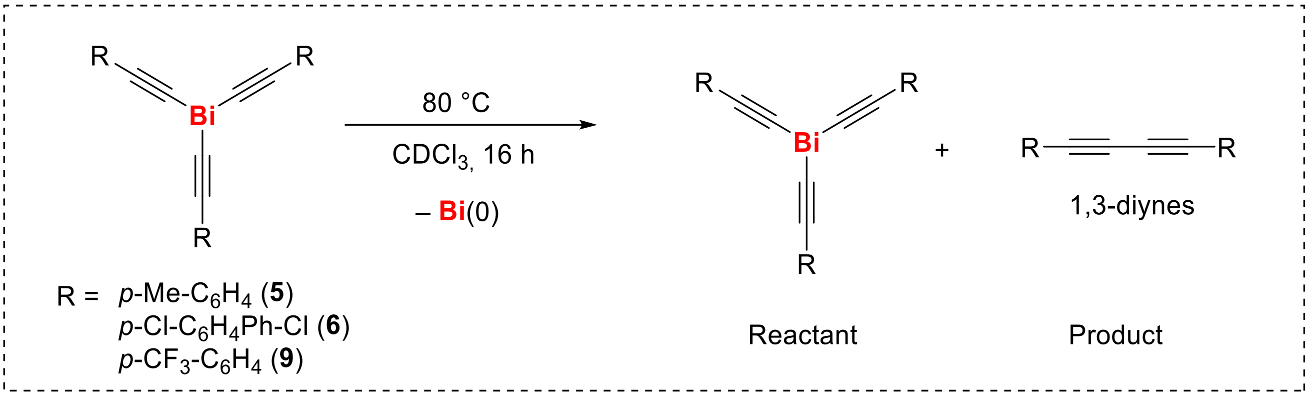

Scheme S3. Conversion of the aromatic bismuth alkynes (**5**, **6**, **7** and **9**) to the respective 1,3-diynes under identical reaction condition (80 °C for 16 h in $CDCl_3$).

Table S4. Stability of the bismuth alkynes (**5**, **6** and **9**).

**Bismuth alkynes**	**1,3-Diynes** (compound number)	**NMR Conversion Reactant: Product**	**Time, Temperature**
**5**	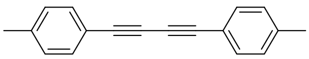 **13**	0.4:1	16 h, 80 °C
**6**	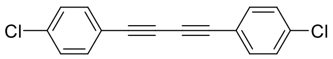 **14**	0.35:1	16 h, 80 °C
**9**	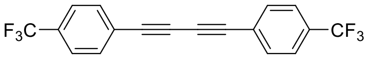 **17**	0.26:1	16 h, 80 °C

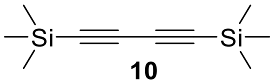

10

1,4-bis(trimethylsilyl)buta-1,3-diyne

1,4-Bis(trimethylsilyl)buta-1,3-diyne (10): White solid; **^{1}H NMR (300 MHz, $CDCl_3$):** δ = 0.21 (s, 2*9 H) ppm. **^{13}C NMR (150 MHz, $CDCl_3$)** δ = –0.31(Si*Me*$_3$), 90.1 (*C*≡C), 92.9 (C≡*C*) ppm.**^{29}Si NMR (99.36 MHz, $CDCl_3$)** δ = -17.10 (*Si*Me$_3$) ppm. **HR-MS (LIFDI, pos.):** Calc. for $(^{12}C_{10}{}^{1}H_{18}{}^{28}Si_2)^+$ ([**10**]$^+$): m/z = 194.09470, found m/z = 194.09305.

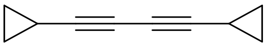

11

1,4-dicyclopropylbuta-1,3-diyne

1,4-Di*cyclo*propylbuta-1,3-diyne (11): Colorless oil; **^{1}H NMR (300 MHz, $CDCl_3$):** δ = 0.76-0.89 (m, 2*4 H, C*H*$_2$), 1.24-1.27 (m, 2*2 H, C*H*). **^{13}C NMR (150 MHz, $CDCl_3$)**: δ = 0.1 (*C*H$_2$), 8.7 (*C*H), 63.4 (*cyclo*Pr-*C*≡C), 80.0 (*C*≡C) ppm. HR-MS spectrum does not provide the exact mass of **11**.

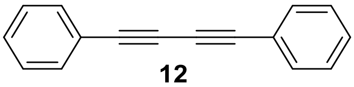

12

1,4-diphenylbuta-1,3-diyne

1,4-Diphenylbuta-1,3-diyne (12): White solid; **^{1}H NMR (300 MHz, Py-d_5):** δ = 7.20-7.22 (m, 2*3, Ph-*H*), 7.53-7.58 (m, 2*2 H, Ph-*H*) ppm. **^{13}C NMR (150 MHz, Py-d_5):** δ = 80.1 (*C*C), 84.7 (C*C*-C_{Ar}), 129.3 (*m*-Ph-*C*), 129.6 (*p*-Ph-*C*), 132.9 (*o*-Ph-*C*) ppm (the resonance for the *ipso*-Ar position overlaps with a solvent signal). **^{13}C NMR (150 MHz, CD_2Cl_2):** 77.6 (*C*C), 82.1 (C*C*-C_{Ar}), 122.2 (*ipso*-Ph-*C*), 129.1 (*m*-Ph-*C*), 129.9 (*p*-Ph-*C*), 133.1 (*o*-Ph-*C*) ppm. **HR-MS (LIFDI, pos.):** Calc. for $(^{12}C1_6{}^{1}H_{10})^+$ ([**12**]$^+$): m/z = 202.07825, found m/z = 202.07779.

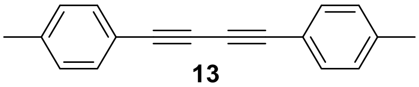

13

1,4-di-*p*-tolylbuta-1,3-diyne

1,4-Di-*p*-tolylbuta-1,3-diyne (13): White solid; **^{1}H NMR (300 MHz, $CDCl_3$):** δ = 2.38 (CH_3), 7.14-7.16 (d, *J* = 7.96 Hz, 2*2 H, Ar-*m-H*), 7.41-7.44 (d, *J* = 8.12 Hz, 2*2 H, Ar-*o-H*) ppm. **^{13}C NMR (150 MHz, $CDCl_3$):** δ = 21.6 (CH_3), 73.5(CC-C_{Ar}), 81.54 (*CC*), 118.8 (*ipso*-Ar-*C*), 129.2 (*m*-Ar-*C*), 132.4 (*o*-Ar-*C*), 139.5 (*p*-Ar-*C*) ppm. **HR-MS (LIFDI, pos.):** Calc. for $(^{12}C_{18}{}^{1}H_{14})^{+}$ ([**13**]$^{+}$): m/z = 230.1090, found m/z =230.1084.

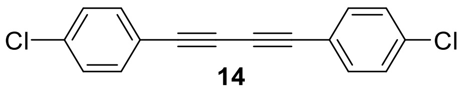

14

1,4-bis(4-chlorophenyl)buta-1,3-diyne

1,4-Bis(*p*-chlorophenyl)buta-1,3-diyne (14): White solid; **^{1}H NMR (250 MHz, $CDCl_3$):** δ = 7.32-7.34 (d, *J* = 8.69 Hz, 2*2 H, Ar-*m-H*), 7.45-7.48 (d, *J* = 8.50 Hz, 2*2 H, Ar-*o-H*) ppm . **^{13}C NMR (150 MHz, $CDCl_3$)**: δ = 73.9 (*CC*), 81.6 (CC-C_{Ar}), 121.8 (*ipso*-Ar-*C*), 128.4 (*m*-Ar-*C*), 129.2 (*o*-Ar-*C*), 132.5 (*p*-Ar-*C*) ppm. **HR-MS (LIFDI, pos.):** Calc. for $(^{12}C_{16}{}^{1}H_{8}{}^{35}Cl_{2})^{+}$ ([**14**]$^{+}$): m/z = 269.9998, found m/z =269.9995.

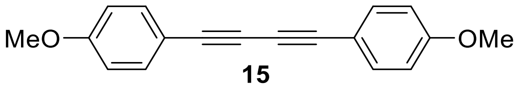

15

1,4-bis(4-methoxyphenyl)buta-1,3-diyne

1,4-Bis(*p*-methoxyphenyl)-1,3-butadiyne (15): White solid; **^{1}H NMR (300 MHz, $CDCl_3$):** δ = 3.82 (OMe), 6.84-6.87 (m, 2*2 H, Ar-*m-H*), 7.43-7.48 (m, 2*2 H, Ar-*o-H*) ppm. **^{13}C NMR (150 MHz, $CDCl_3$):** δ = 55.3 (O*Me*), 72.9 (*CC*), 81.2 (CC-C_{Ar}), 113.9 (*ipso*-Ar-*C*), 133.6 (*m*-Ar-*C*), 134 (*o*-Ar-*C*), 160.2 (*p*-Ar-*C*) ppm. **HR-MS (LIFDI, pos.):** Calc. for $(^{12}C_{18}{}^{1}H_{14}{}^{18}O_{2})^{+}$ ([**15**]$^{+}$): m/z = 262.0988, found m/z =262.0986.

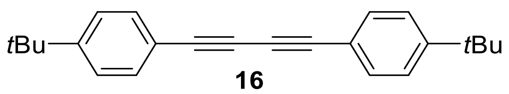

16

1,4-bis(4-(*tert*-butyl)phenyl)buta-1,3-diyne

1,4-bis(4-(*tert*-butyl)phenyl)buta-1,3-diyne (16): White solid; **^{1}H NMR (300 MHz, $CDCl_3$):** δ = 7.36-7.39 (m, 2*2 H), 7.47-7.50 (d, 2*2 H, Ar-*o*-*H*) ppm. **^{13}C NMR (150 MHz, $CDCl_3$):** δ = 31.1 (*t*Bu), 73.5 (*CC*), 81.5 (CC-C_{Ar}), 118.9 (*ipso*-Ar-*C*), 125.5 (*m*-Ar-*C*), 132.3 (*o*-Ar-*C*), 152.6 (*p*-Ar-*C*) ppm. **HR-MS (LIFDI, pos.):** Calc. for $(^{12}C_{24}{}^{1}H_{26})^{+}$ ([**16**]$^{+}$): m/z = 314.2027, found m/z =314.2029.

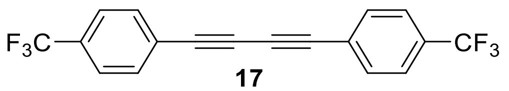

17

1,4-bis(4-(trifluoromethyl)phenyl)buta-1,3-diyne

1,4-bis(4-(trifluoromethyl)phenyl)buta-1,3-diyne (17): White solid; **^{1}H NMR (300 MHz, $CDCl_3$):** δ = 7.63 (q, *J* = 8.4 Hz, *J* = 4.0 Hz, 8 H) ppm. **^{19}F NMR (377 MHz, $CDCl_3$):** –63.0 ppm. **^{13}C NMR (150 MHz, $CDCl_3$): ^{13}C NMR (150 MHz, $CDCl_3$):** δ = 75.6 (*CC*), 80.9 (*CC*-C_{Ar}), 123.7 (q, $^{1}J_{C\text{-}F}$ = 272.8 Hz, CF_3), 125.2 (*ipso*-Ar-*C*), 125.5 (q, $^{3}J_{C\text{-}F}$ =3.82 Hz, *m*-Ar-*C*), 131.1 (q, $^{1}J_{C\text{-}F}$ = 33.4 Hz, *p*-Ar-C), 132.8 (*o*-Ar-*C*) ppm. **HR-MS (LIFDI, pos.):** Calc. for $(^{12}C_{18}{}^{1}H_{8}{}^{19}F_{6})^{+}$ ([**17**]$^{+}$): m/z = 338.0525, found m/z =338.0519.

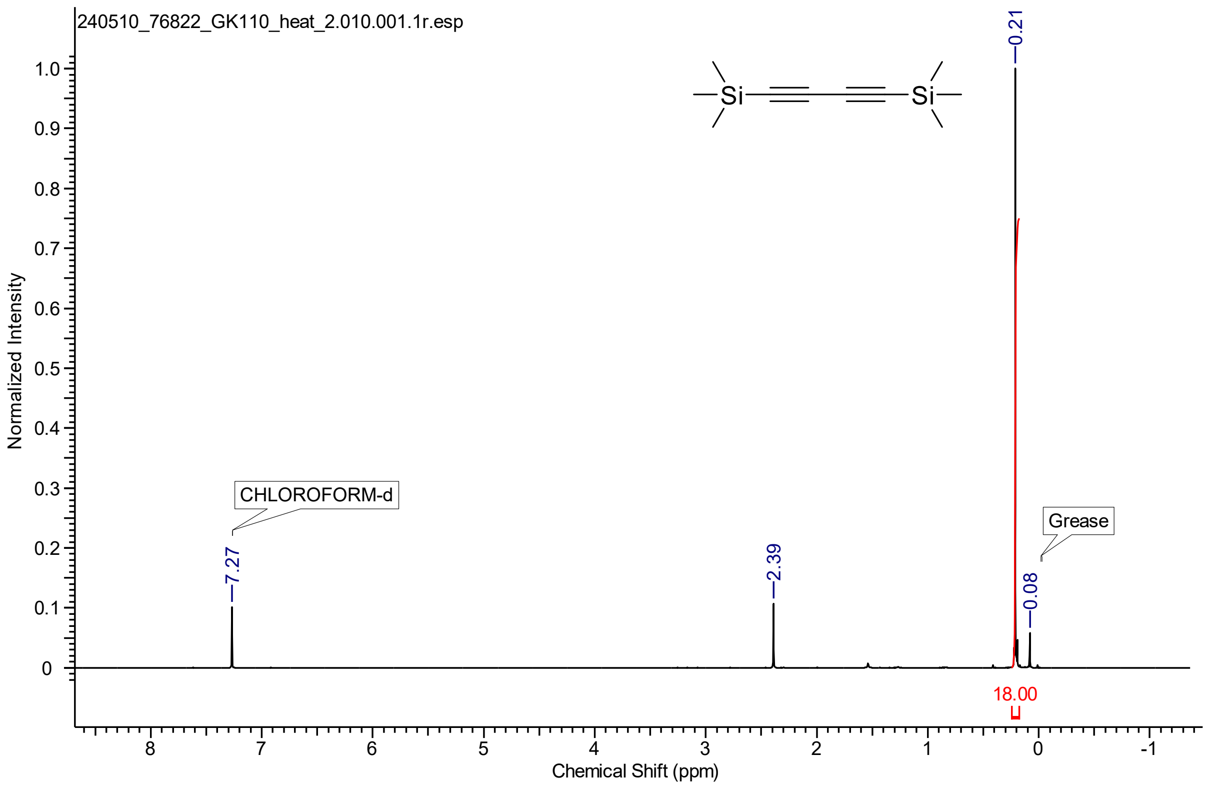

Figure S29. ^{1}H NMR spectrum of **10** in $CDCl_3$.

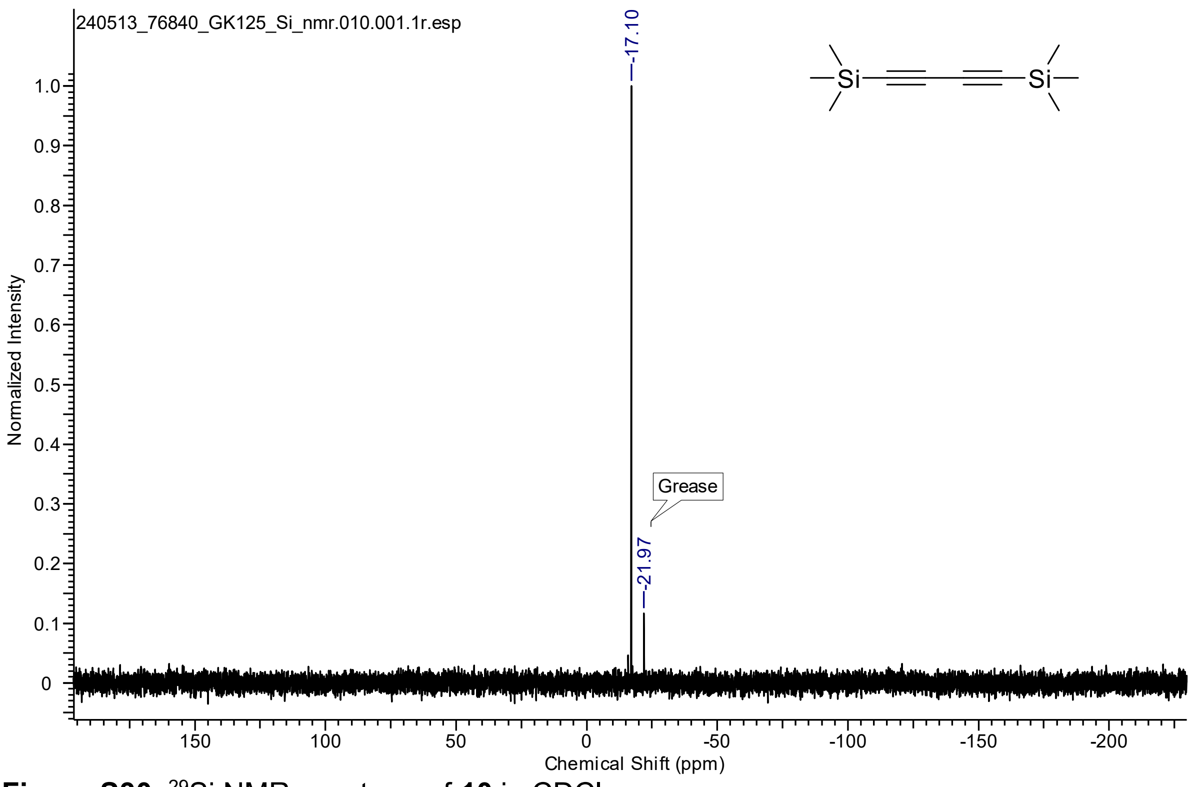

Figure S30. ^{29}Si NMR spectrum of **10** in $CDCl_3$.

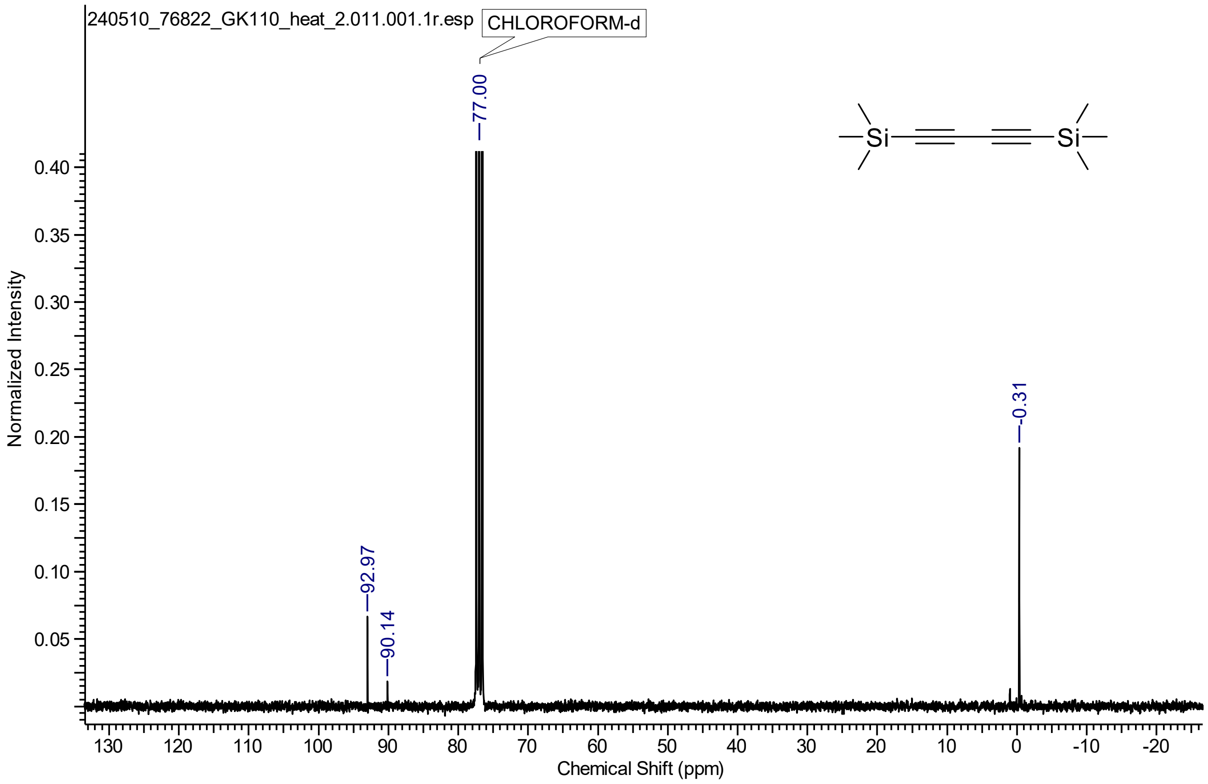

Figure S31. ^{13}C NMR spectrum of **10** in $CDCl_3$.

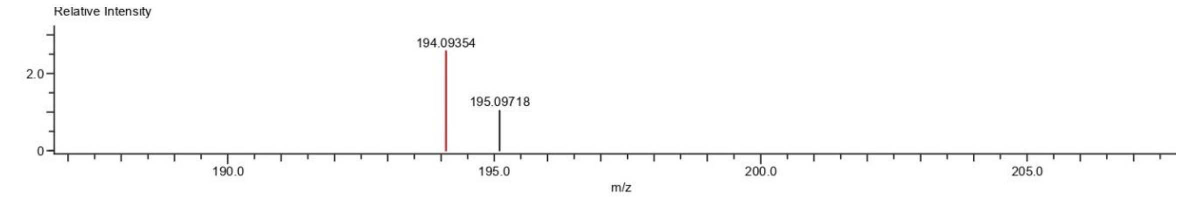

Composition: $C_{10}H_{18}Si_2$
Mono Isotopic Mass:194.09470
Description:

Average Mass:194.42092

Created:13.05.2024 08:44:10
Nominal Mass:194
Created by:Lorakis

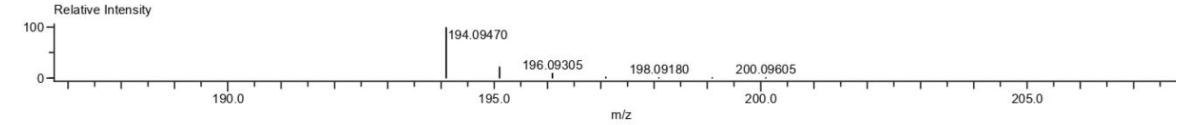

Mass	Intensity	Relative Intensity	Calc. Mass	Mass Difference [mDa]	Mass Difference [ppm]	Possible Formula	Unsaturation Number
194.09354	2052.94	2.58	194.09470	-1.16	-5.99	$^{12}C_{10}{}^{1}H_{18}{}^{28}Si_2$	4.0

Figure S32. HR-MS spectrum of **10**.

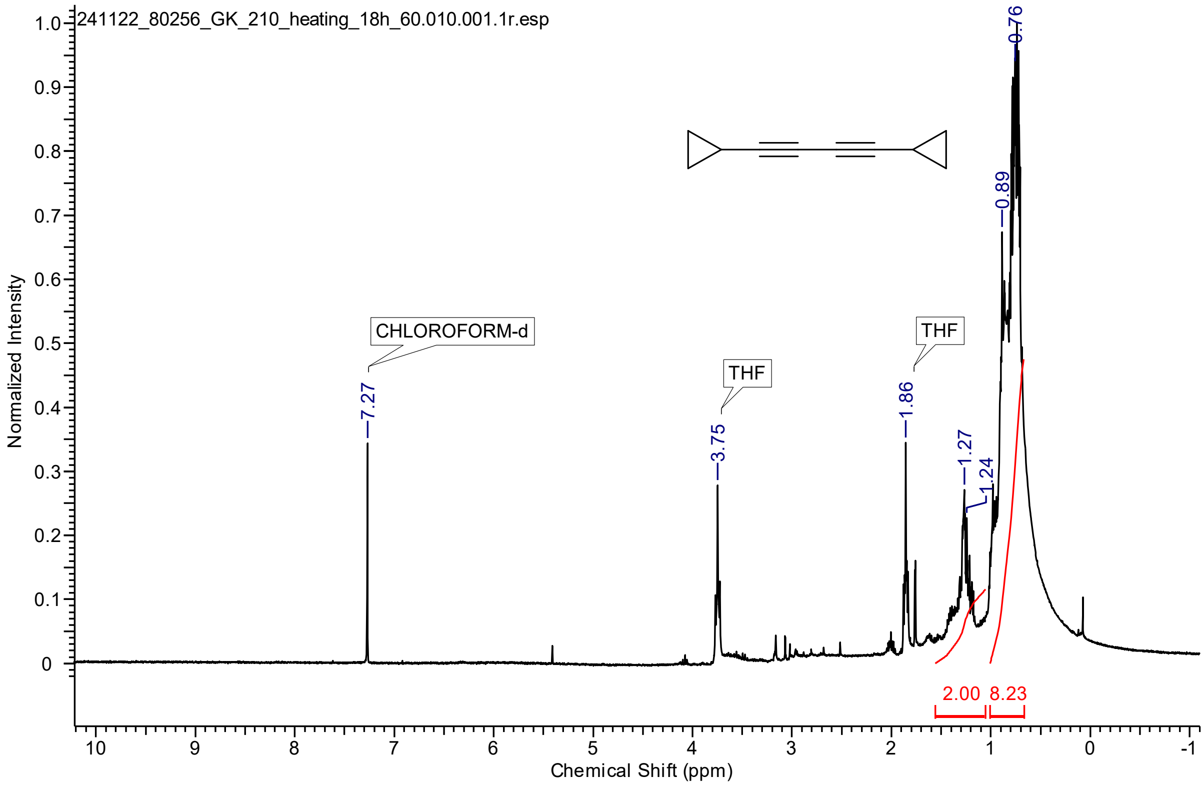

Figure S33. ^{1}H NMR spectrum of **11** in $CDCl_3$.

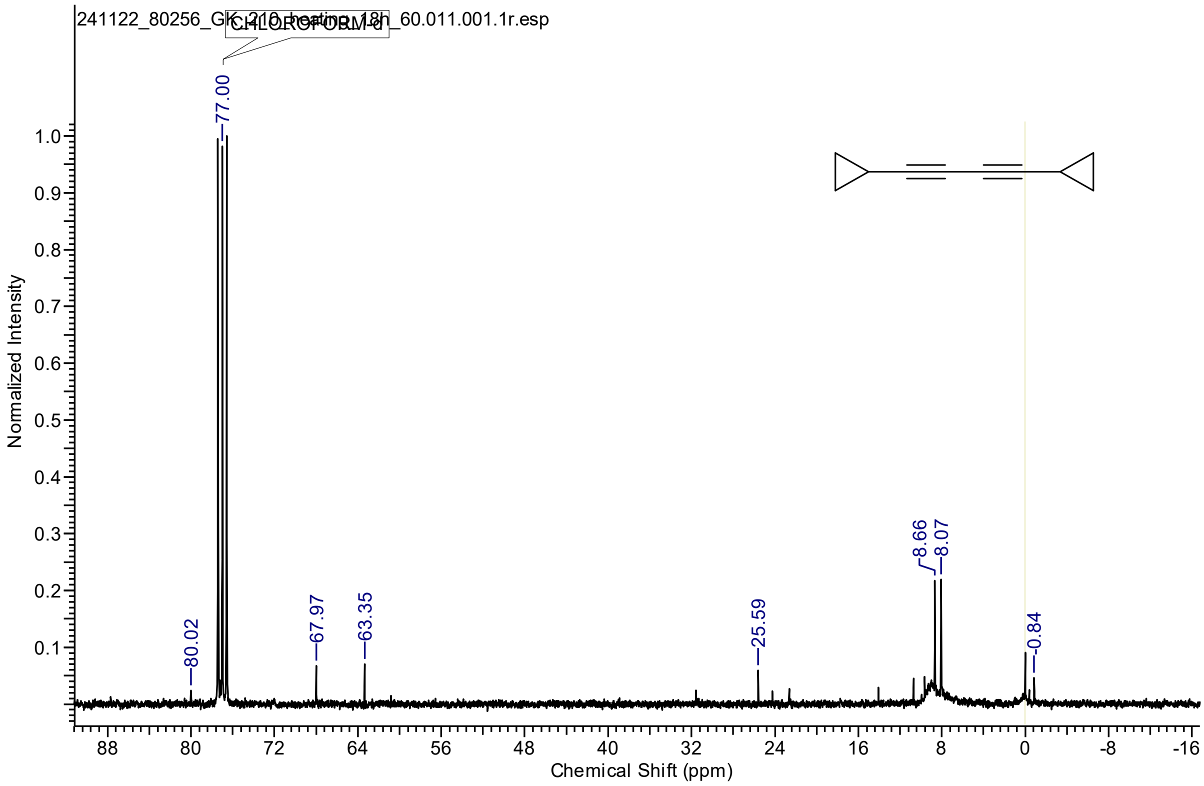

Figure S34. ^{13}C NMR spectrum of **11** in $CDCl_3$.

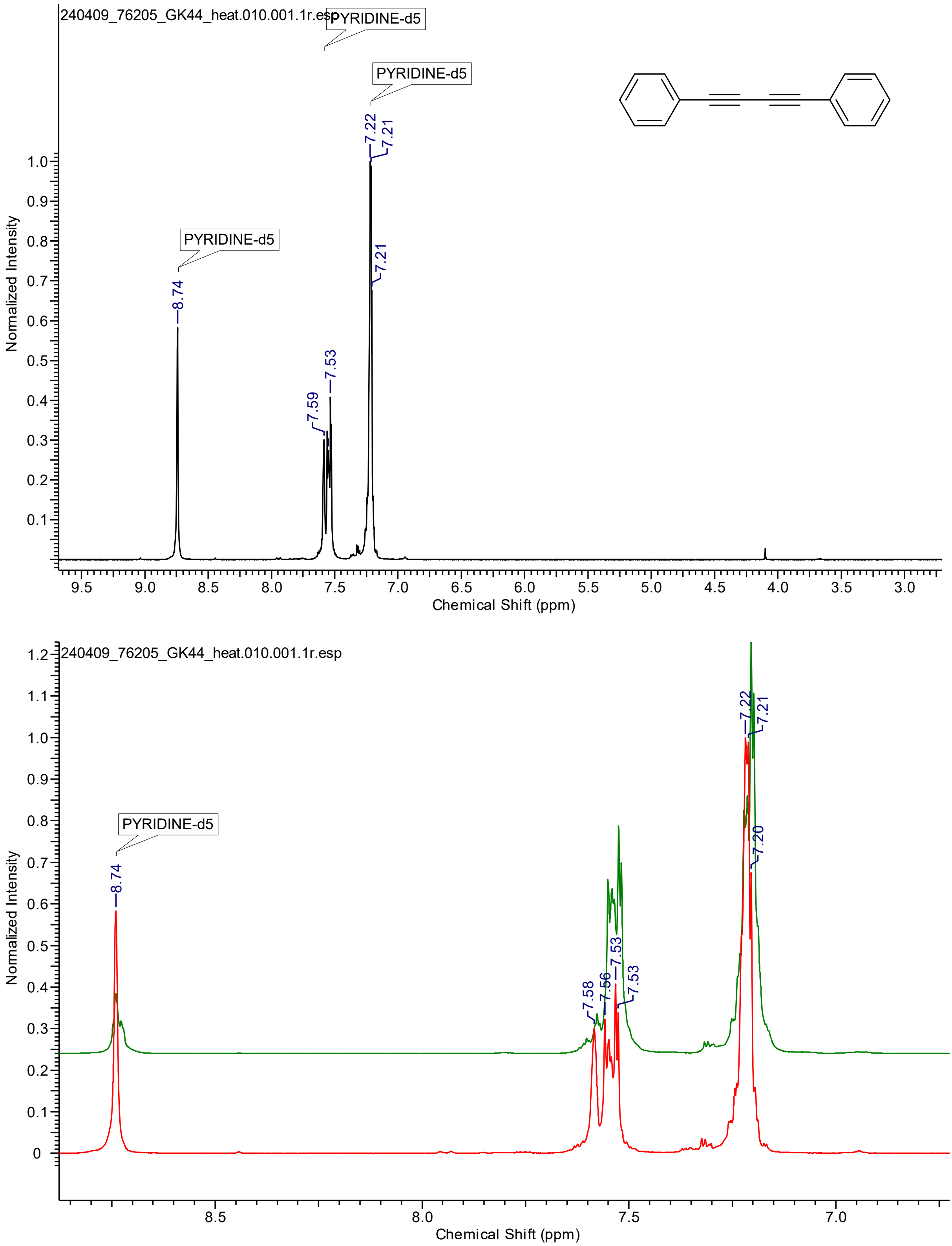

Figure S35. ^{1}H NMR spectrum of **12** in CD_2Cl_2 (top spectrum) and Py-d_5 (bottom spectrum).

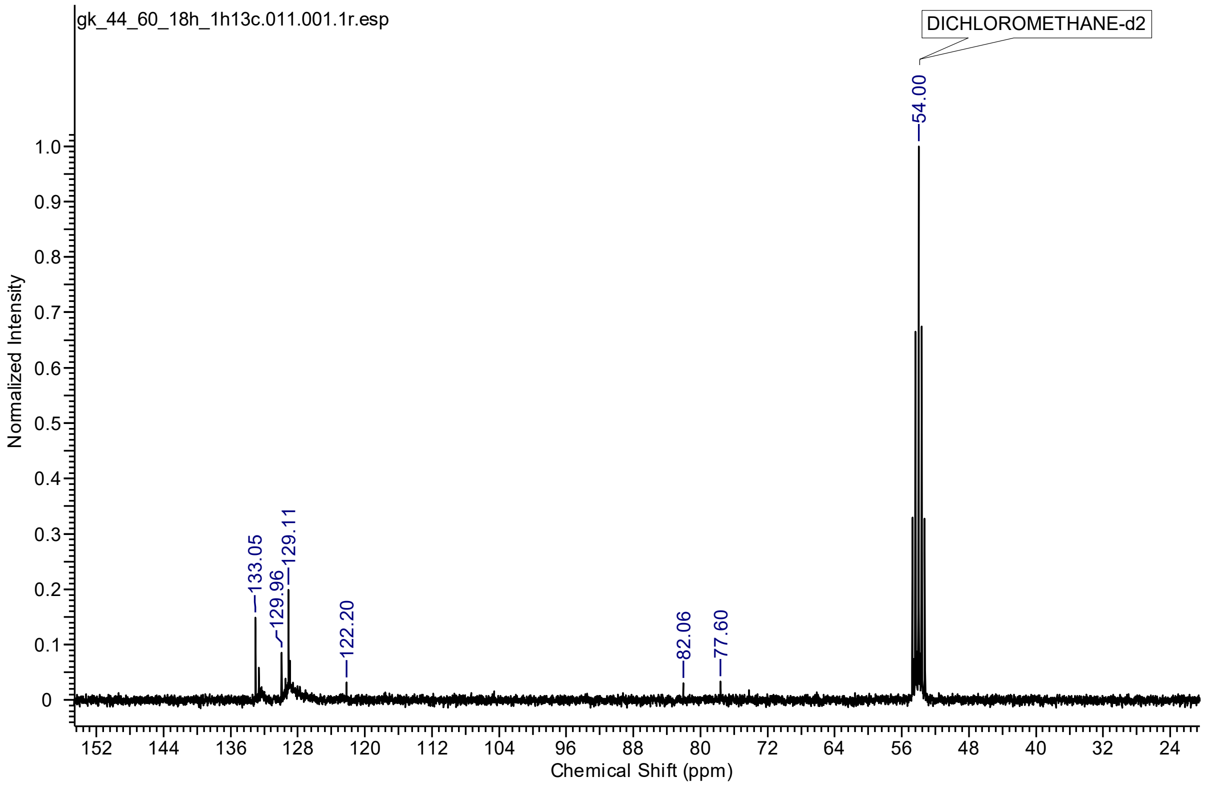

Figure S36. ^{13}C NMR spectrum of **12** in CD_2Cl_2.

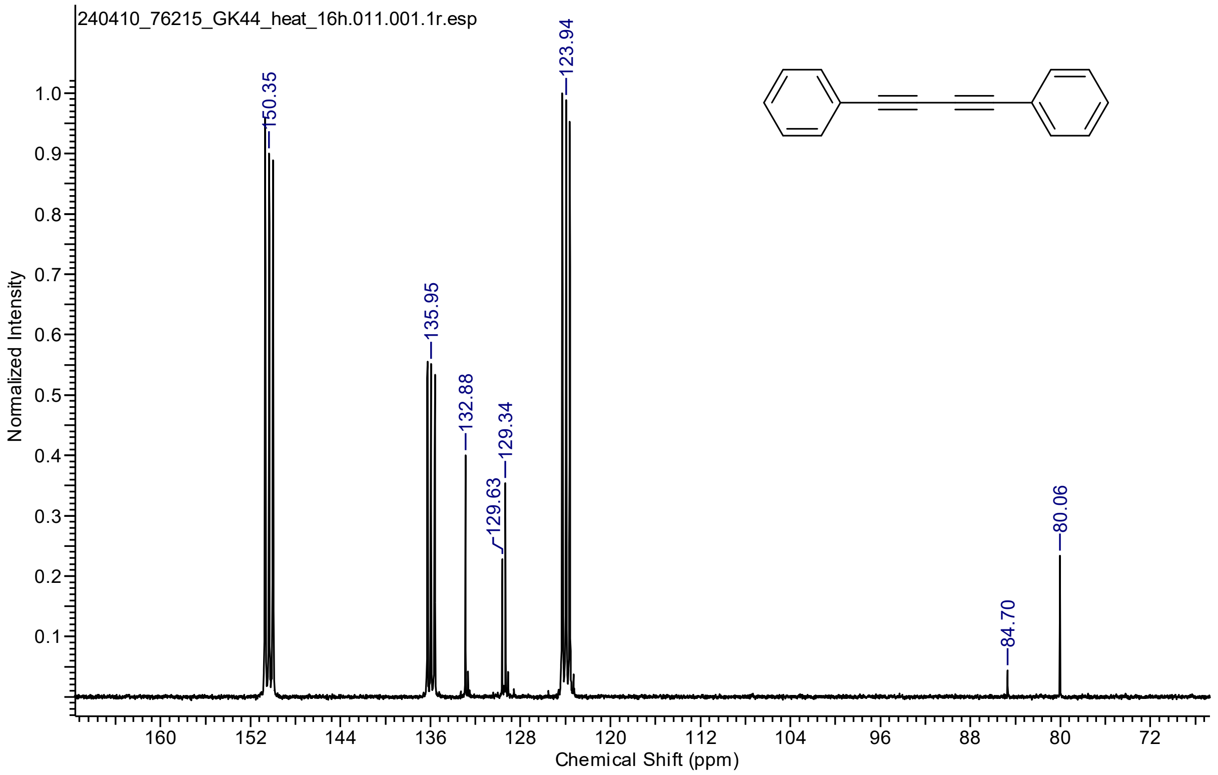

Figure S37. ^{13}C NMR spectrum of **12** in Py-d_5.

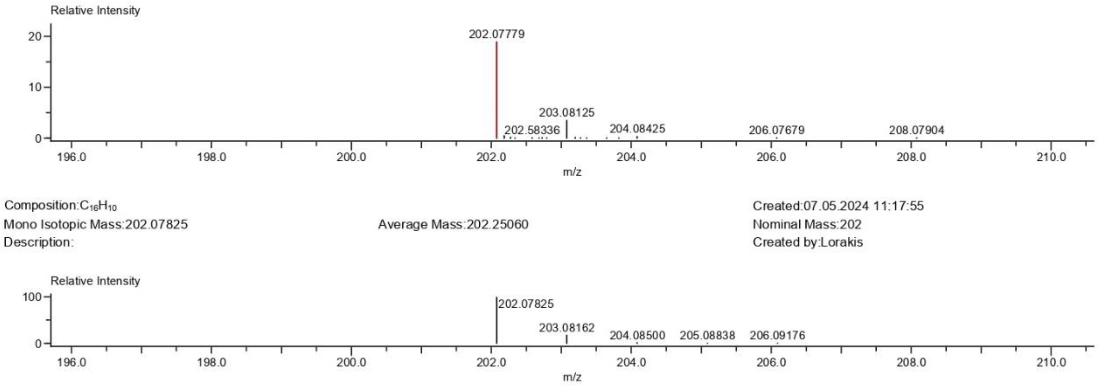

Mass	Intensity	Relative Intensity	Calc. Mass	Mass Difference [mDa]	Mass Difference [ppm]	Possible Formula	Unsaturation Number
202.07779	22103.12	18.84	202.07825	-0.46	-2.28	$^{12}C_{16}{}^{1}H_{10}$	12.0

Figure S38. HR-MS spectrum of **12**.

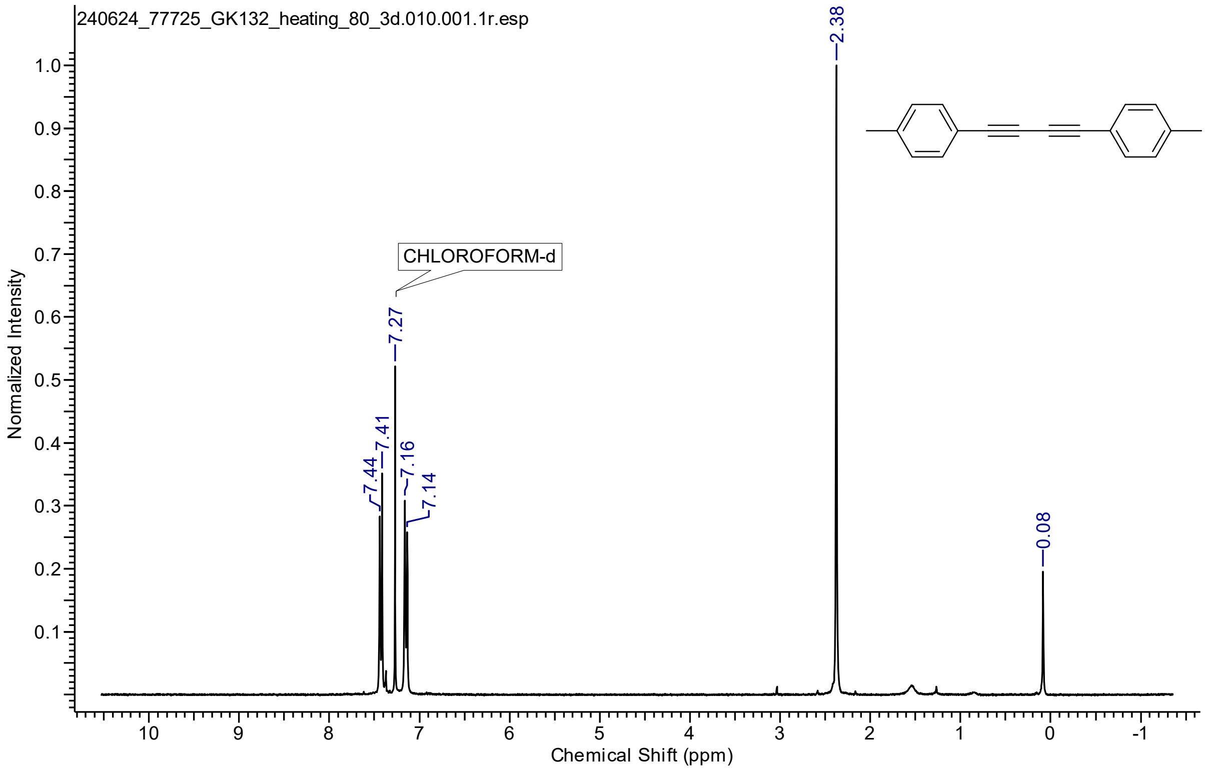

Figure S39. ^{1}H NMR spectrum of **13** in $CDCl_3$.

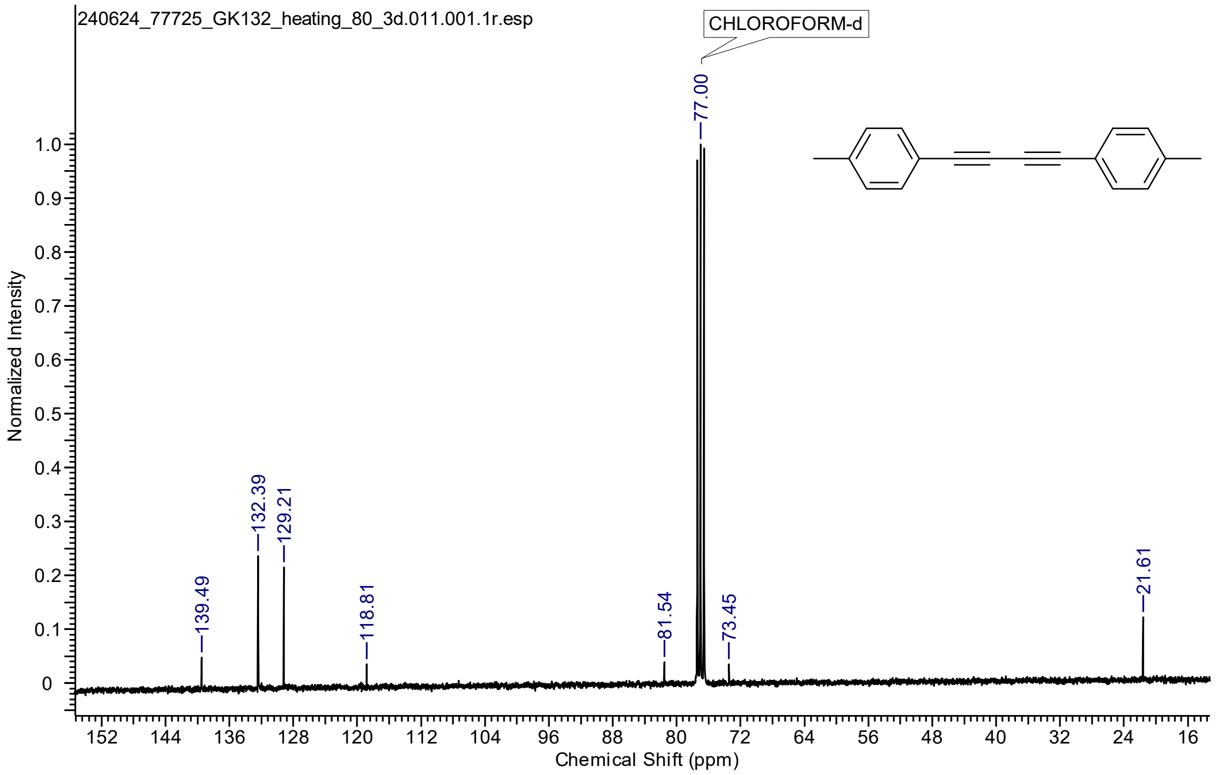

Figure S40. ^{13}C NMR spectrum of **13** in $CDCl_3$.

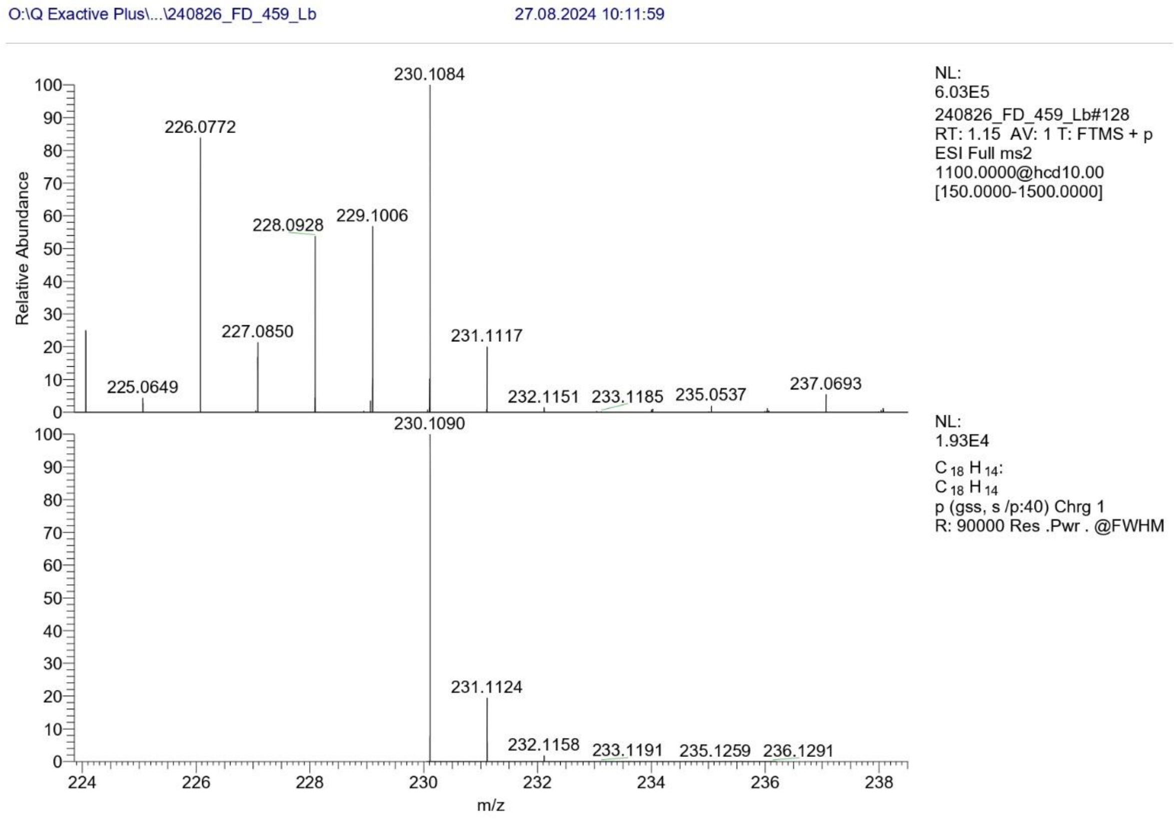

Figure S41. HR-MS spectrum of **13**.

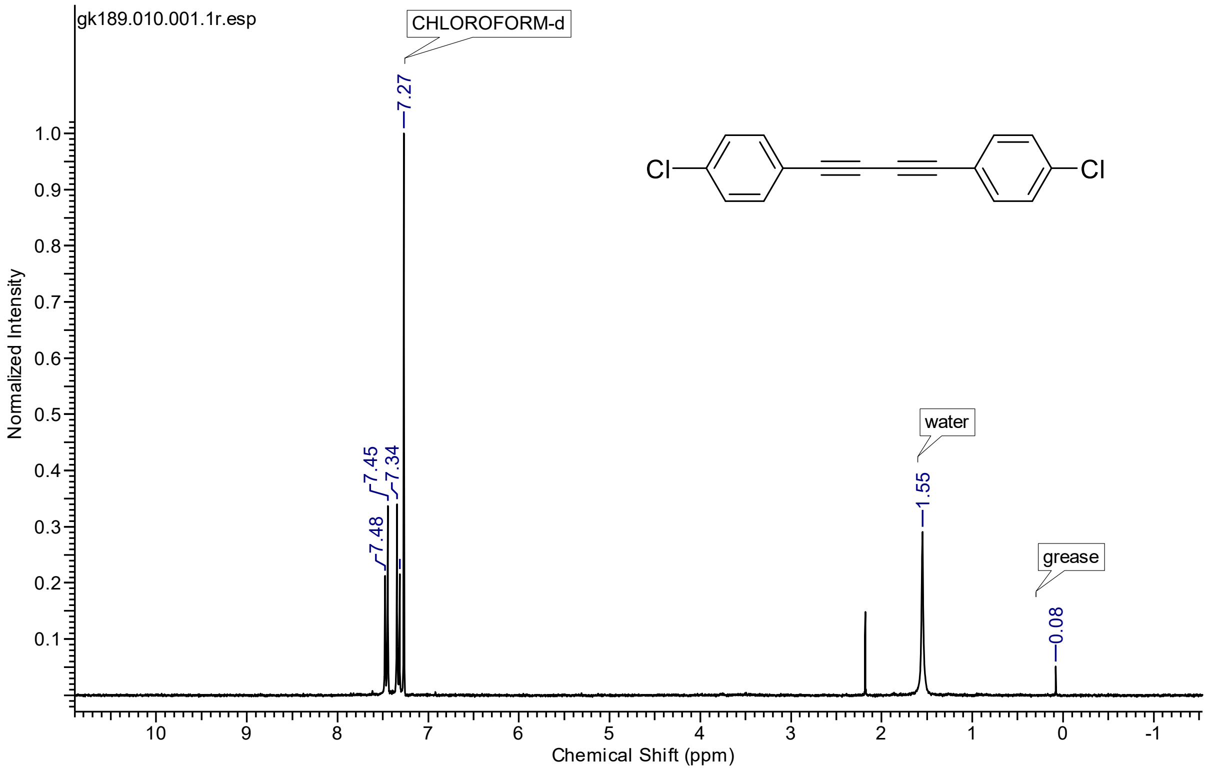

Figure S42. ^{1}H NMR spectrum of **14** in $CDCl_3$.

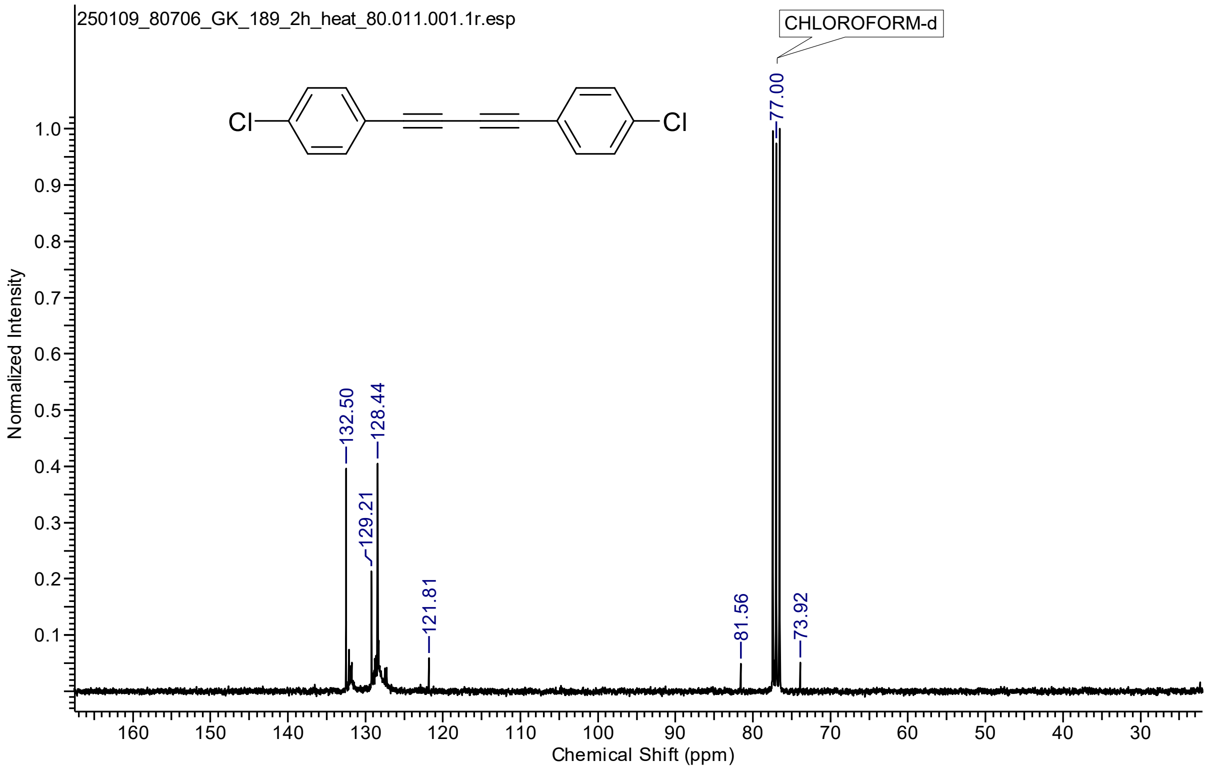

Figure S43. ^{13}C NMR spectrum of **14** in $CDCl_3$.

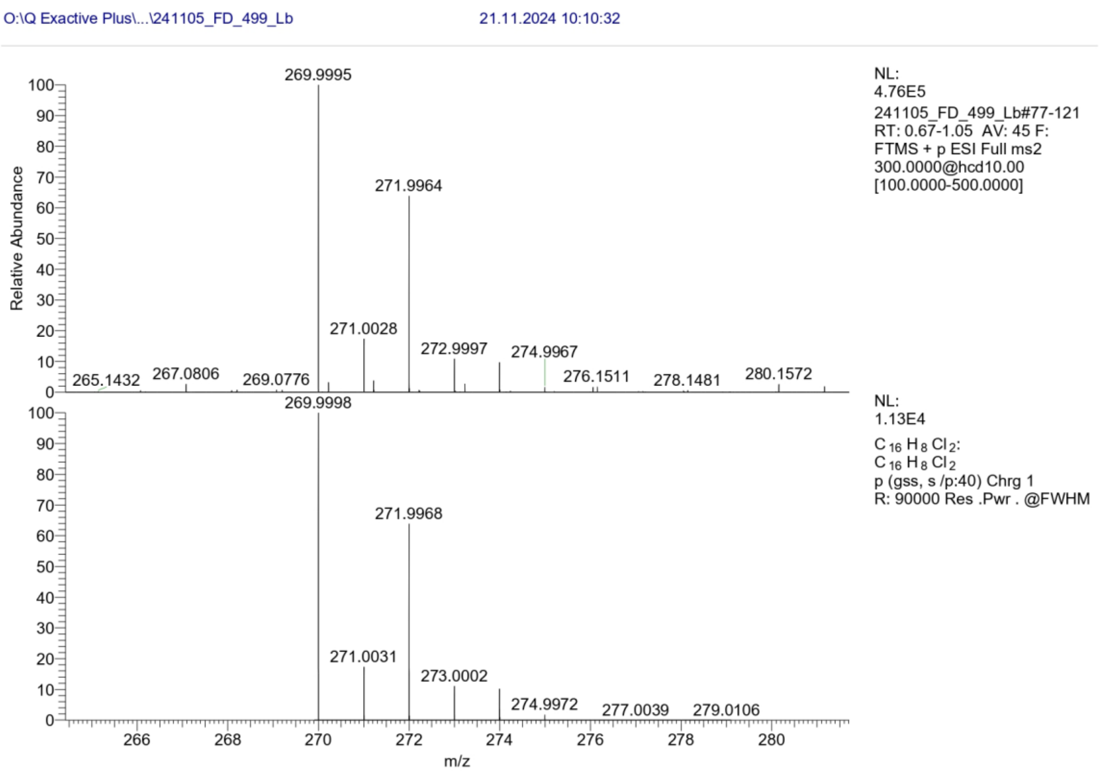

Figure S44. HR-MS spectrum of **14**.

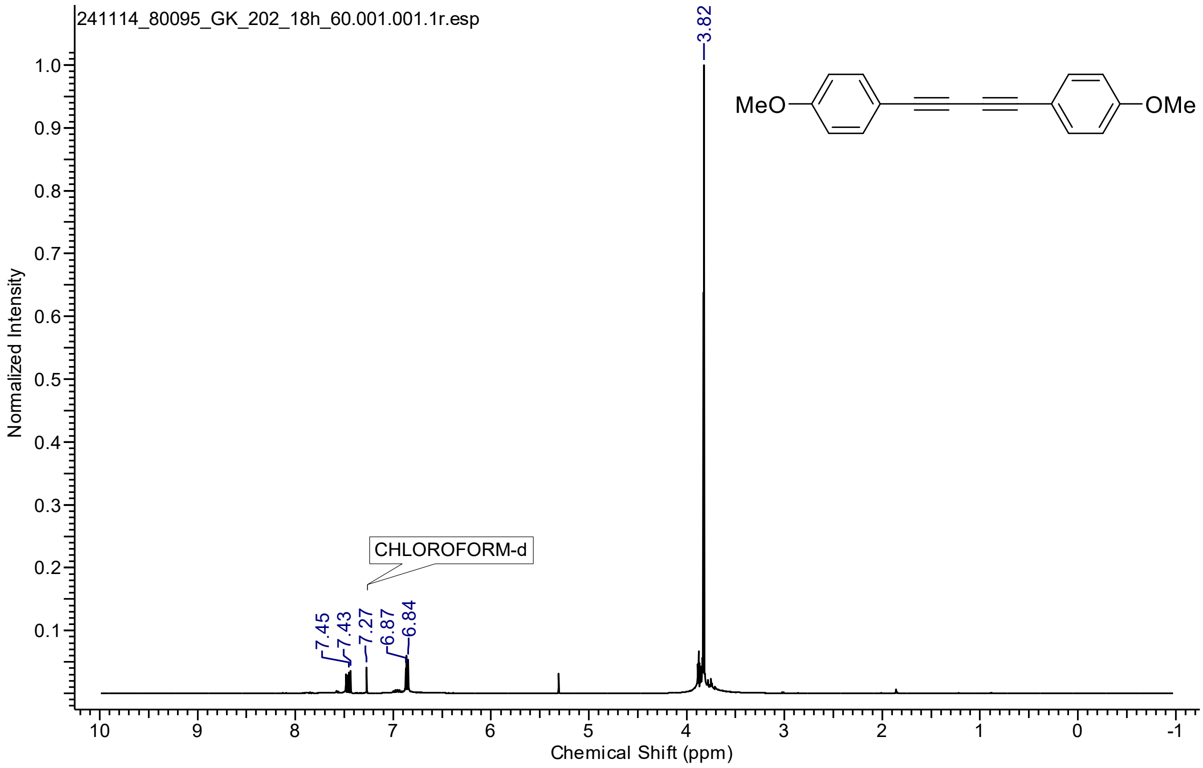

Figure S45. ^{1}H NMR spectrum of **15** in $CDCl_3$.

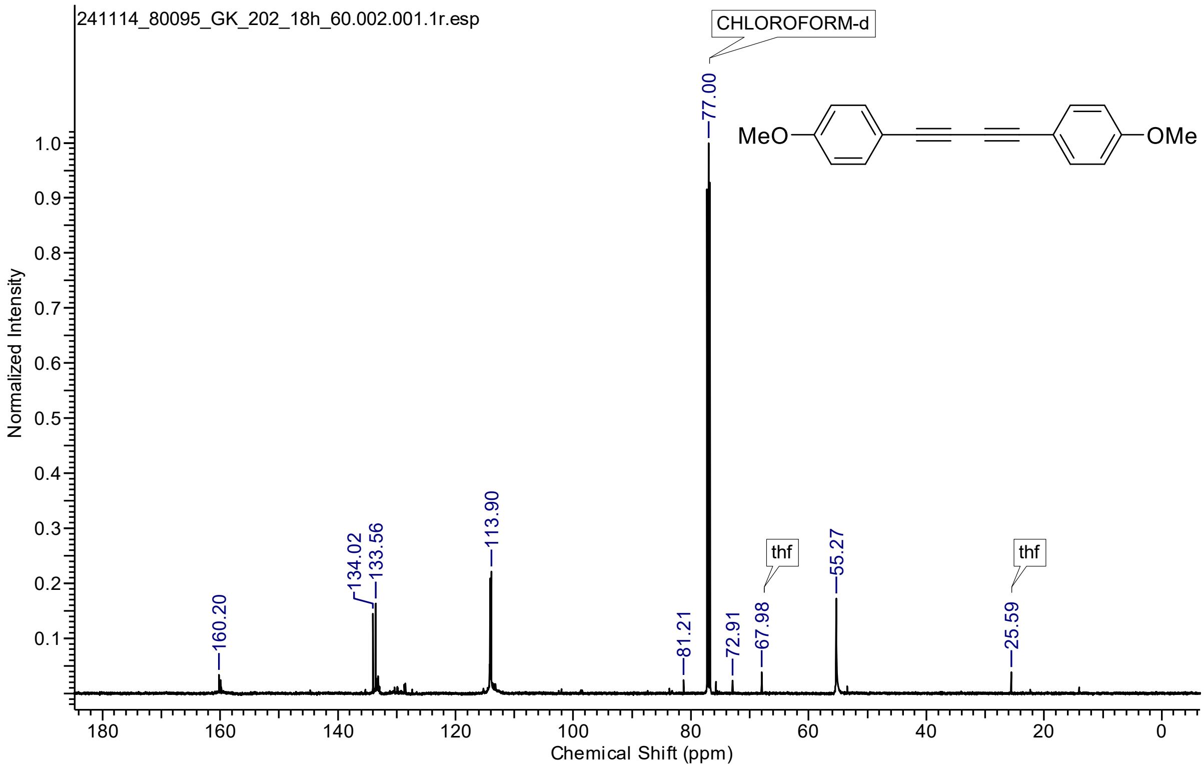

Figure S46. ^{13}C NMR spectrum of **15** in $CDCl_3$.

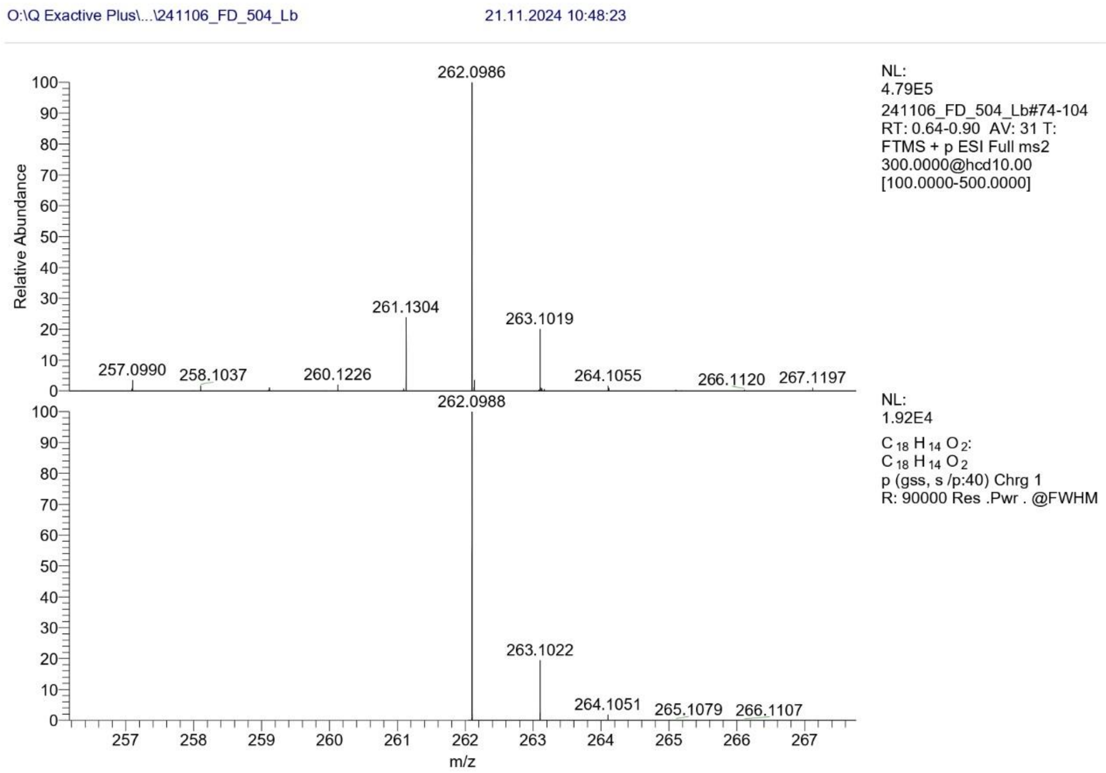

Figure S47. HR-MS spectrum of **15**.

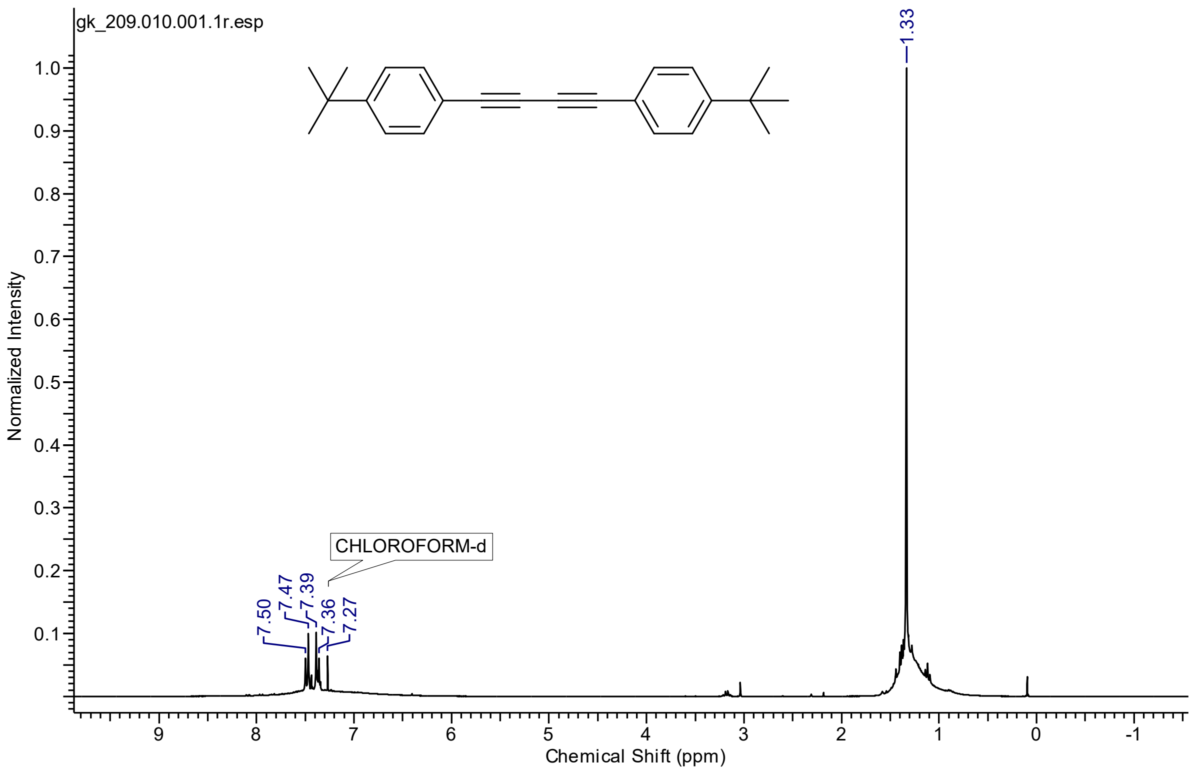

Figure S48. ^{1}H NMR spectrum of **16** in $CDCl_3$.

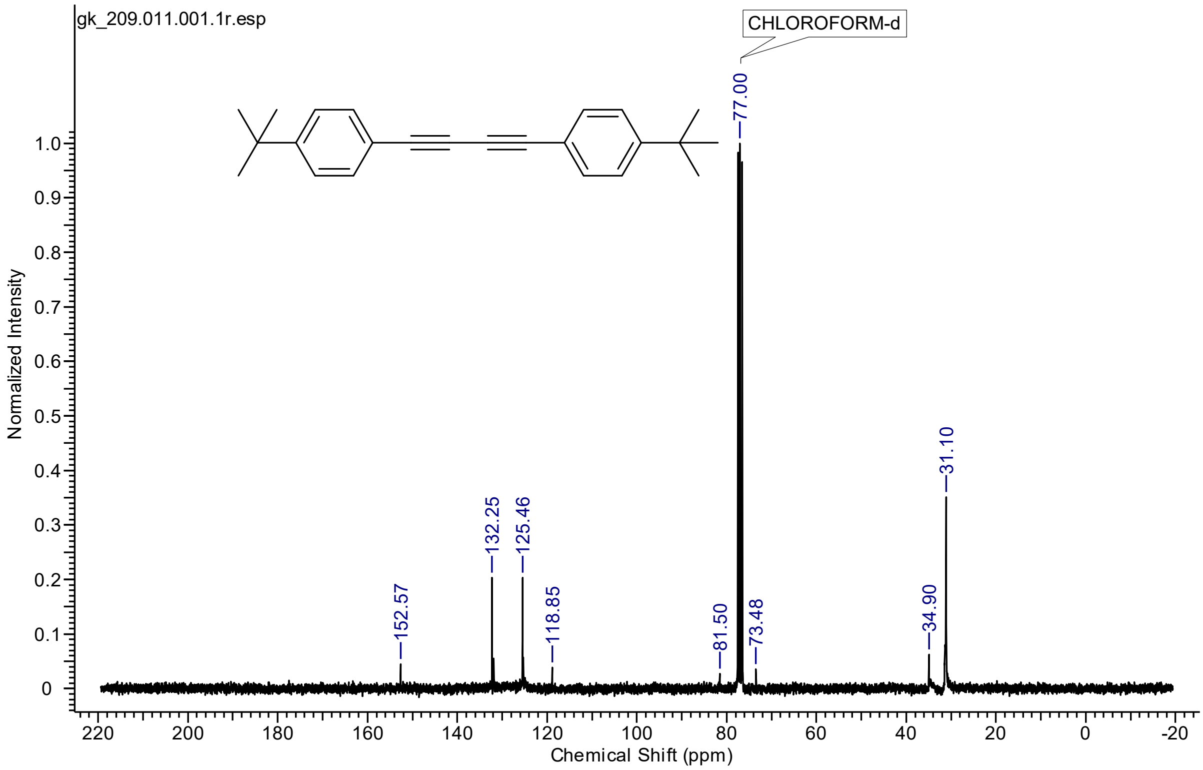

Figure S49. ^{13}C NMR spectrum of **16** in $CDCl_3$.

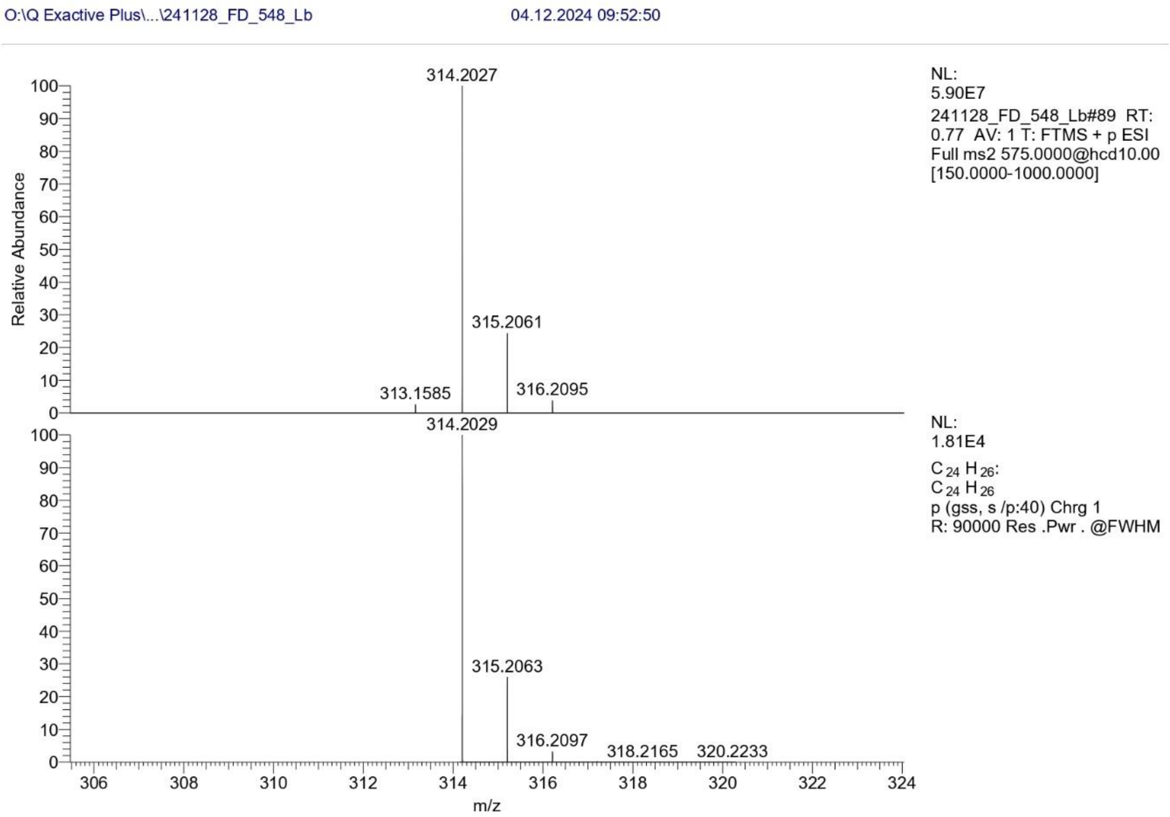

Figure S50. HR-MS spectrum of **16**.

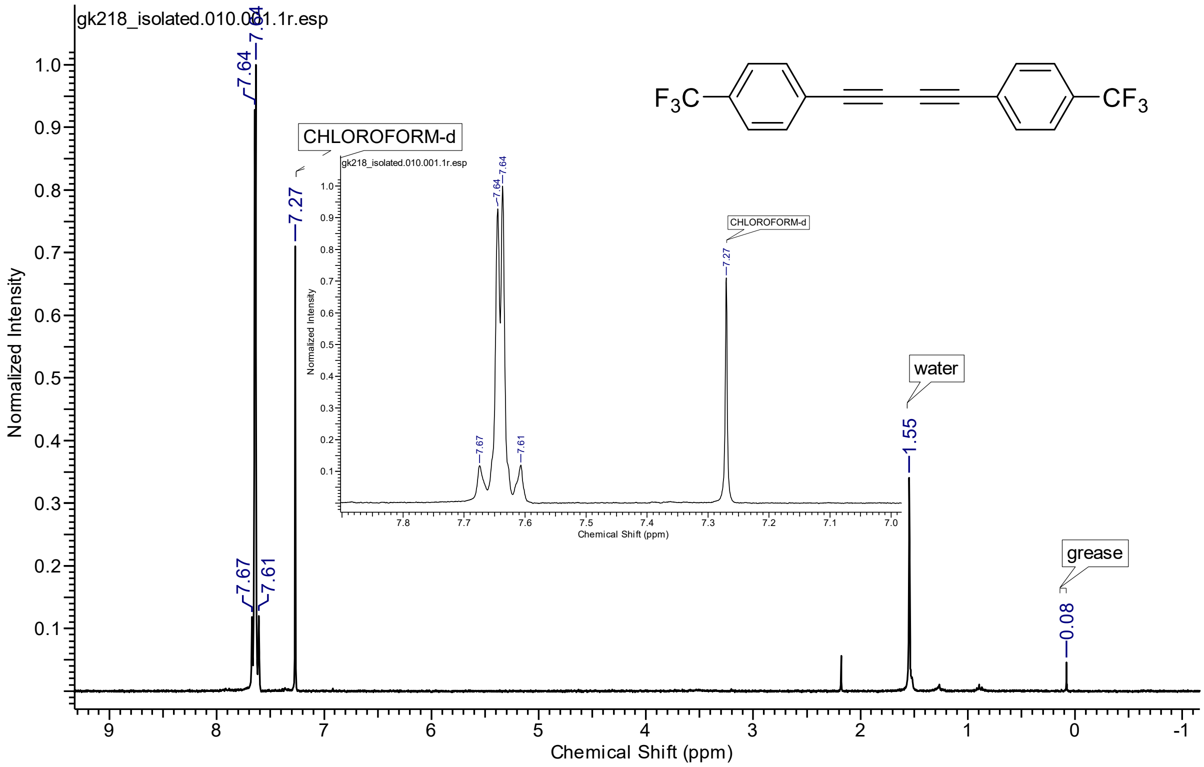

Figure S51. ^{1}H NMR spectrum of **17** in $CDCl_3$.

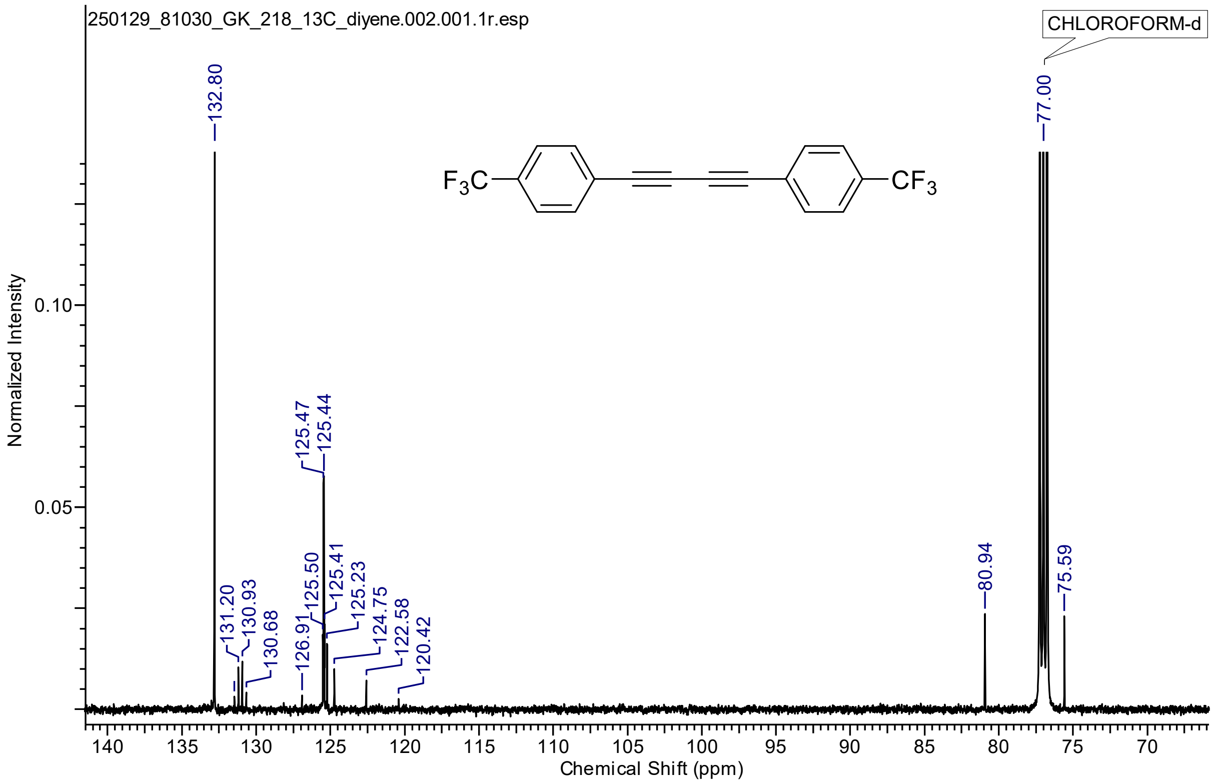

Figure S52. ^{13}C NMR spectrum of **17** in $CDCl_3$.

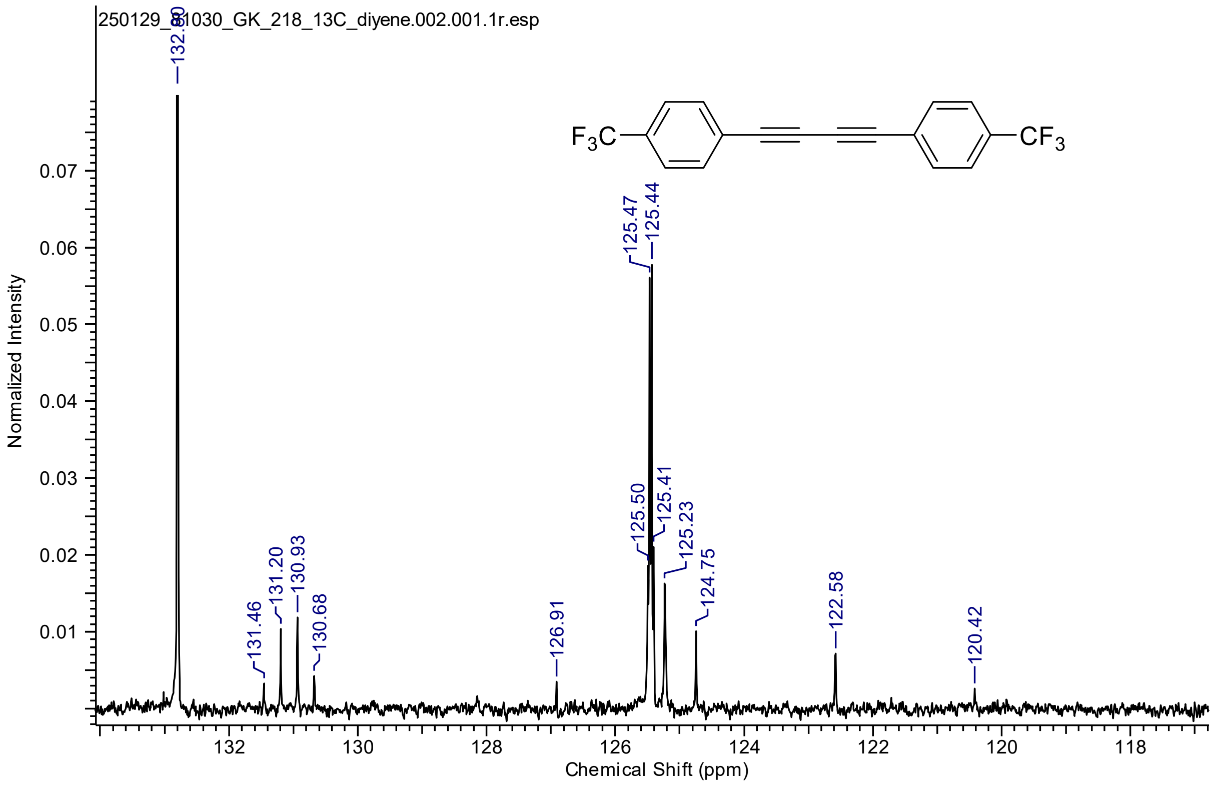

Figure S53. Zoom-in on the aromatic region of the ^{13}C NMR spectrum of **17** in $CDCl_3$.

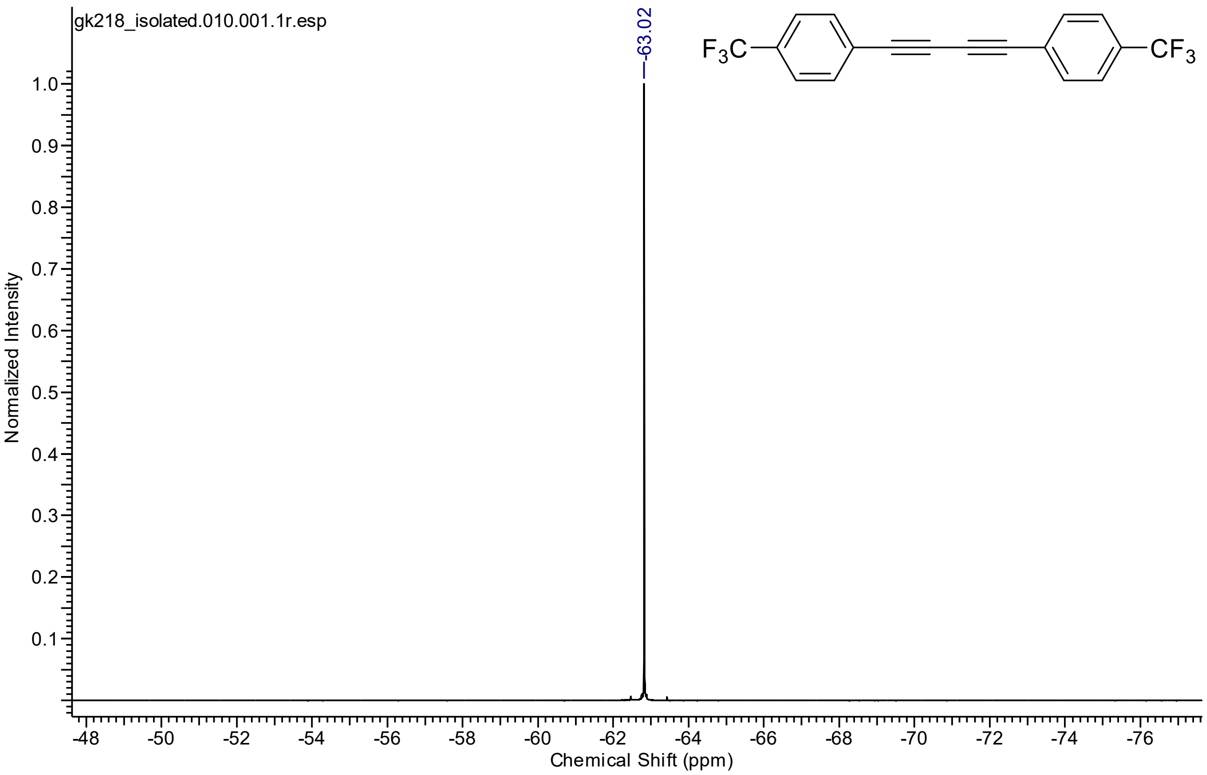

Figure S54. ^{19}F NMR spectrum of **17** in $CDCl_3$.

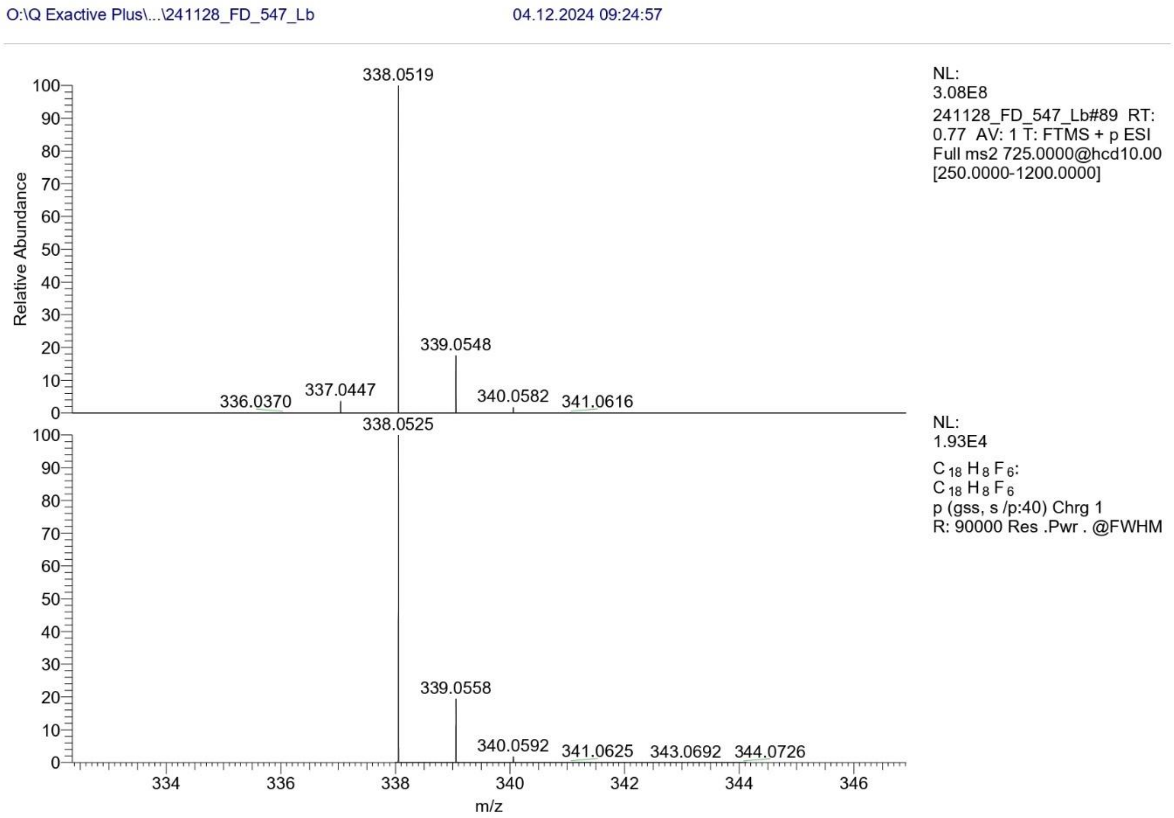

Figure S55. HR-MS spectrum of **17**.

6. Synthesis and MS data of C–C cross-coupling products

The synthesis of unsymmetrical 1,3-diynes from the reaction in solution of two different bismuth alkynes $Bi(C{\equiv}CR)_3$ was investigated. Upon heating equimolar mixtures of two distinct bismuth alkynes, high-resolution mass spectrometry (HR-MS) confirmed the formation of the expected cross-coupling products. However, significant formation of homocoupling products (~30% conversion) and, in some cases, THF α-alkynylation byproducts (up to 10% yield) was observed. Owing to the low selectivity and complexity of the product mixture, we did not proceed with the isolation of the cross-coupling products.

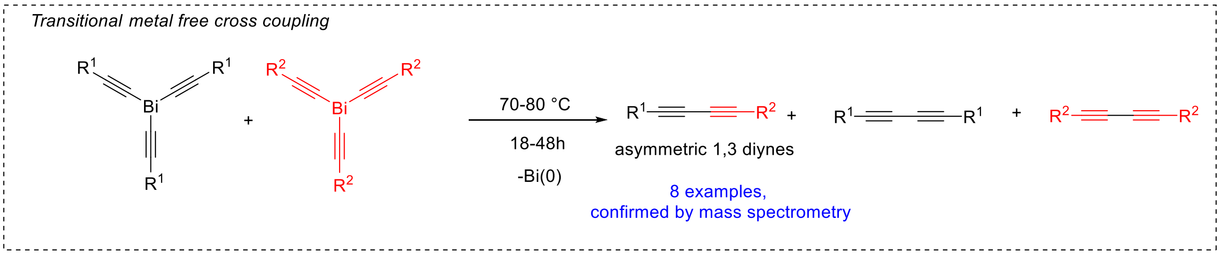

Scheme S4. Synthesis of the C–C cross-coupling products **19-26**.

General procedure: The two bismuth alkynes (1:1 molar ratio) were heated in a J.-Young NMR tube in THF or $CDCl_3$ (0.5 mL) as the solvent. After the required reaction time (see table below) the solution was filtered to remove the black Bi^0 and all volatiles were removed from the filtrate under reduced pressure to give the solid product as a pure 1,3-diyne. The temperature and time needed for the complete conversion of the starting materials is given in the table below. The solid products (**10-17**) were characterized by NMR spectroscopy and high-resolution mass spectrometry.

Table S5. Synthesis of unymmetrical 1,3-diynes.

Unsymmetric 1,3-diynes[a] (compound number)	Reaction conditions	cross-coupling: homo-coupling (a) : homo-coupling (b)
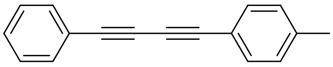 **19**	THF, 24 h, 80 °C	40 : 25 : 25[b]
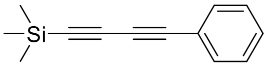 **20**	THF, 18 h, 75 °C	48 : 26 : 26
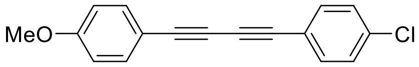 **21**	$CDCl_3$, 24 h, 80 °C	43 : 28 : 28
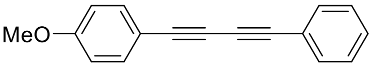 **22**	$CDCl_3$, 17 h, 80 °C	43 : 28 : 28
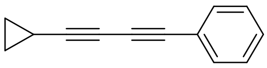 **23**	THF, 17 h, 70 °C	40 : 30 : 30[c]
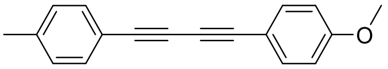 **24**	$CDCl_3$, 3d, 80 °C	38 : 26 : 26
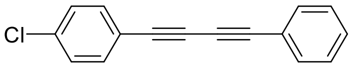 **25**	$CDCl_3$, 48 h, 80 °C	30 : 30 : 30[b]
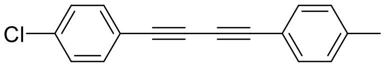 **26**	THF, 18 h, 80 °C	40 : 30 : 30[c]

[a]: Full conversion of the starting materials Bi(C≡CR)$_3$ was observed in all cases, as determined by ^{1}H NMR spectroscopy. [b]: in addition, 5% of α-alkynylated THF (from C–H activation) and 5% of free alkyne were observed. [c]: This value contains a higher uncertainty because of signal overlap in the ^{1}H NMR spectrum.

Note: Compound Bi(C≡CPh)$_3$ (**4**) can contain THF molecules that show weak interactions with the molecular complex (as observed in the molecular structure obtained from single-crystal XRD data). In cross-coupling reactions, we have in some cases observed the respective THF α-alkynylated species as a minor product (ca. 5-10%) along with the formation of free alkyne in equal amounts, especially when longer reaction times and higher reaction temperatures were necessary (e.g. heating solutions that contain **4** up to 80 °C for 24 h or more). The NMR spectra are in good agreement with the previous literature.[63,98,99]

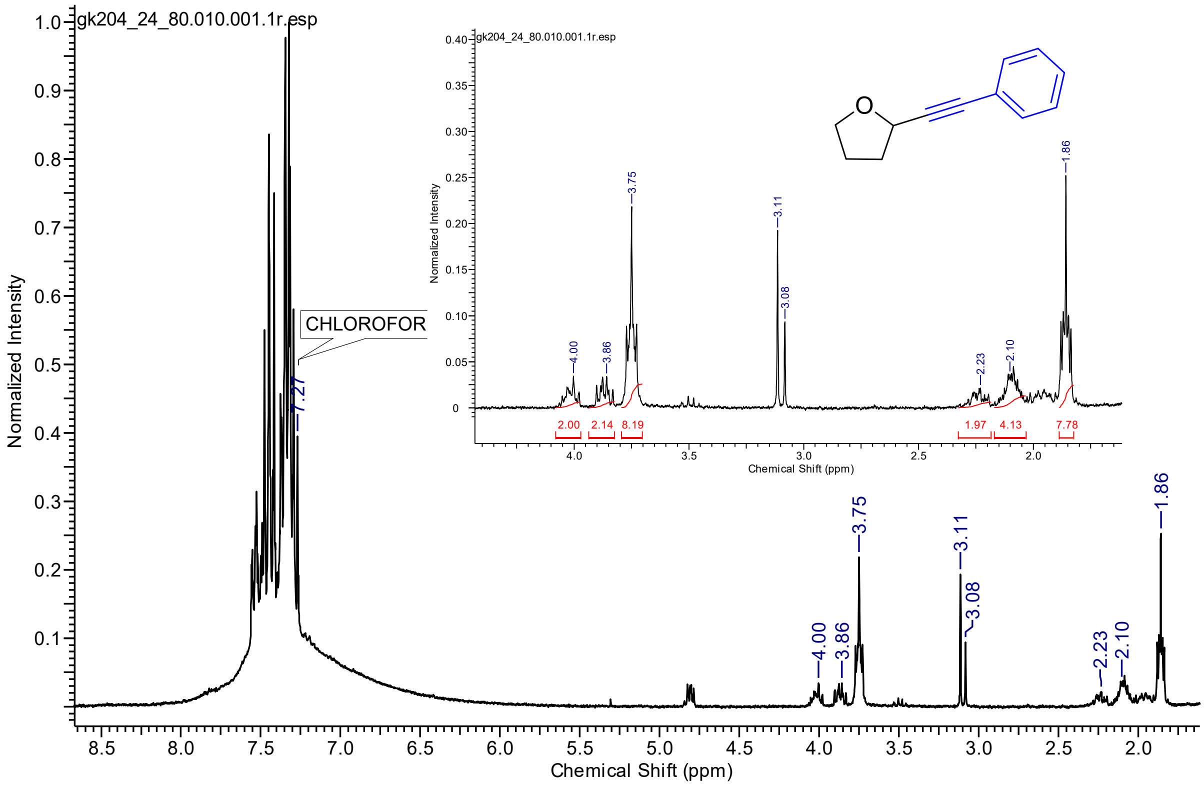

Figure S56. ^{1}H NMR of the mixture of **4** and **5** after heating at 80 °C for 24 h in $CDCl_3$.

Scope of unsymmetrical 1,3-diynes (formation also confirmed by HRMS):

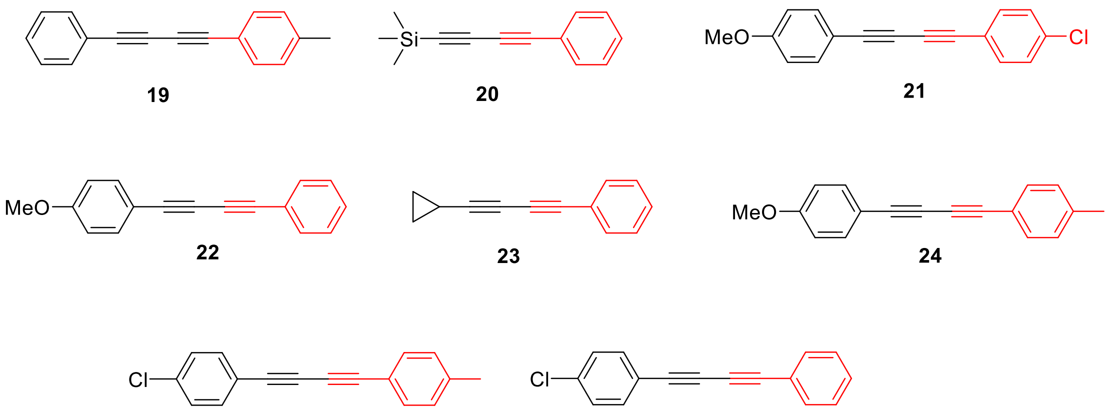

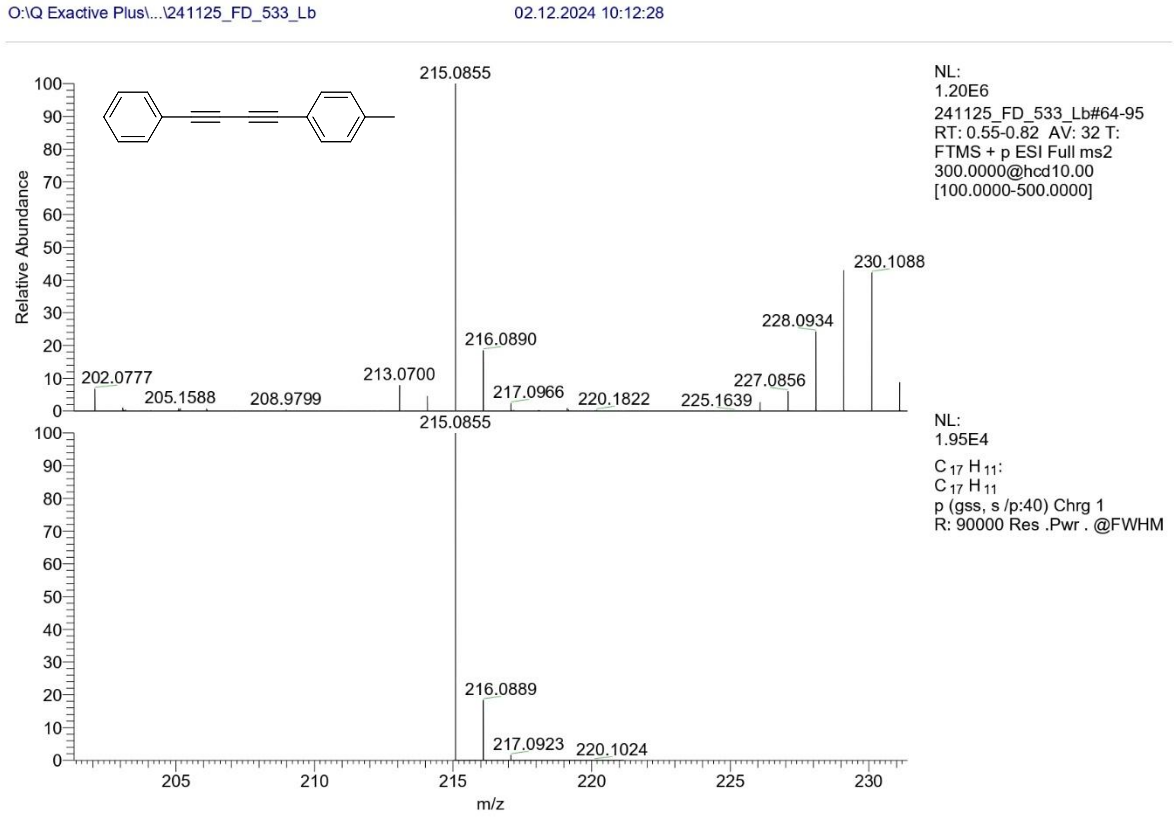

Figure S57. HR-MS spectrum of **19**.

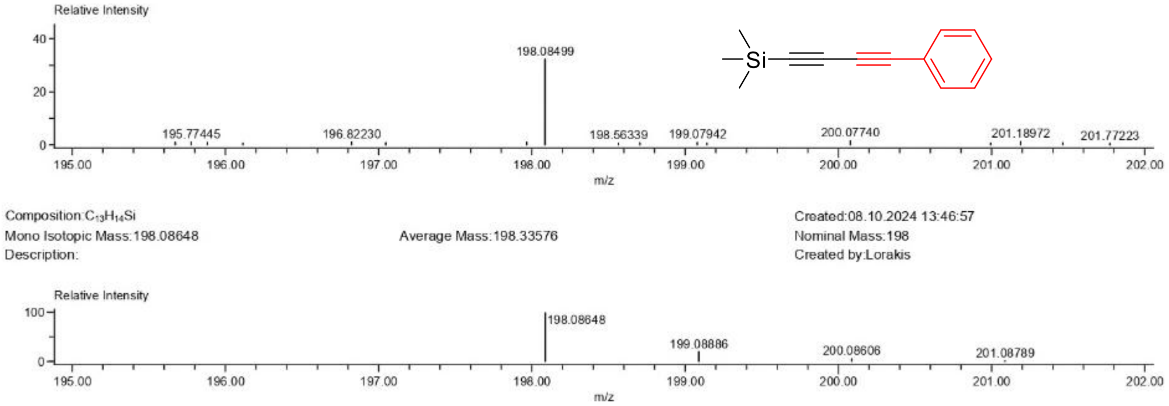

Mass	Intensity	Relative Intensity	Calc. Mass	Mass Difference [mDa]	Mass Difference [ppm]	Possible Formula	Unsaturation Number
198.08499	874.71	32.25	198.08648	-1.49	-7.53	$^{12}C_{13}{}^{1}H_{14}{}^{28}Si_1$	8.0

Figure S58. HR-MS spectrum of **20**.

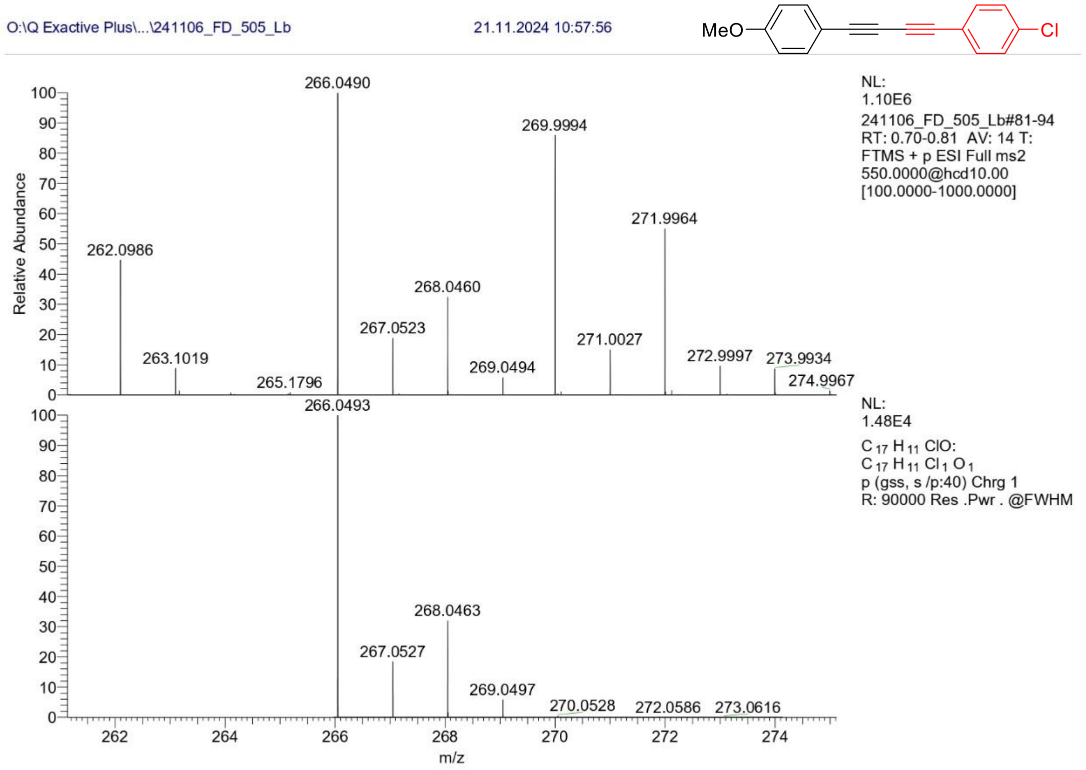

Figure S59. HR-MS spectrum of **21**.

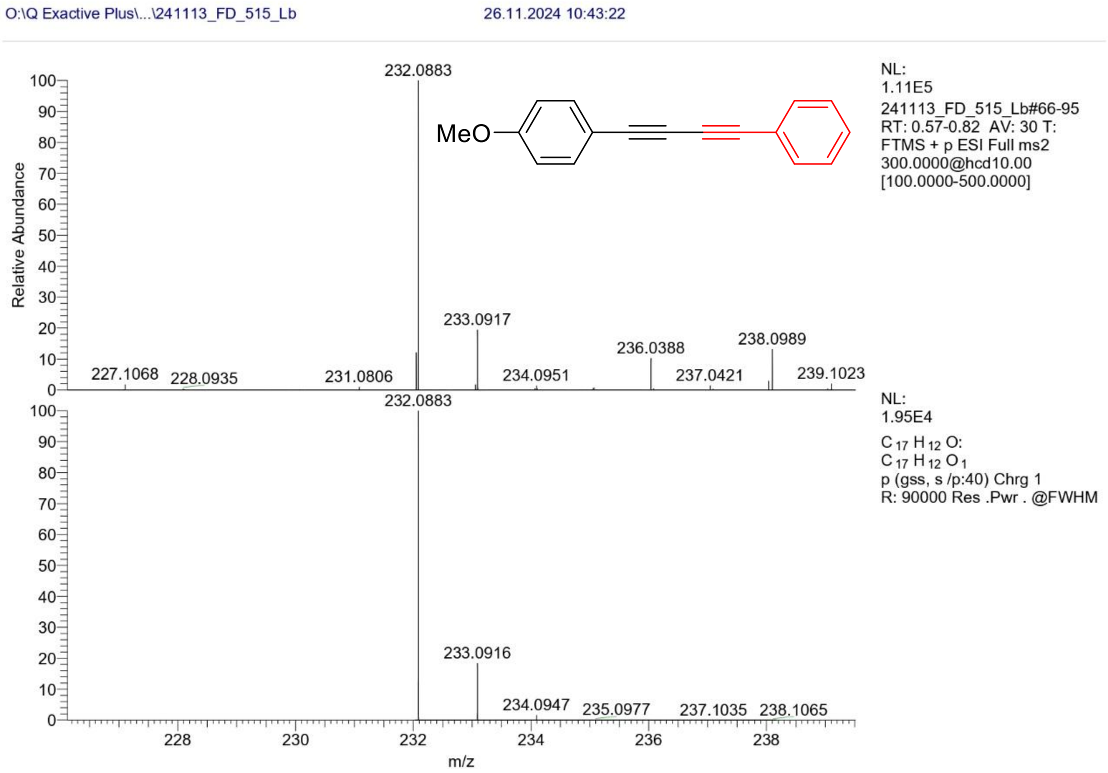

Figure S60. HR-MS spectrum of **22**.

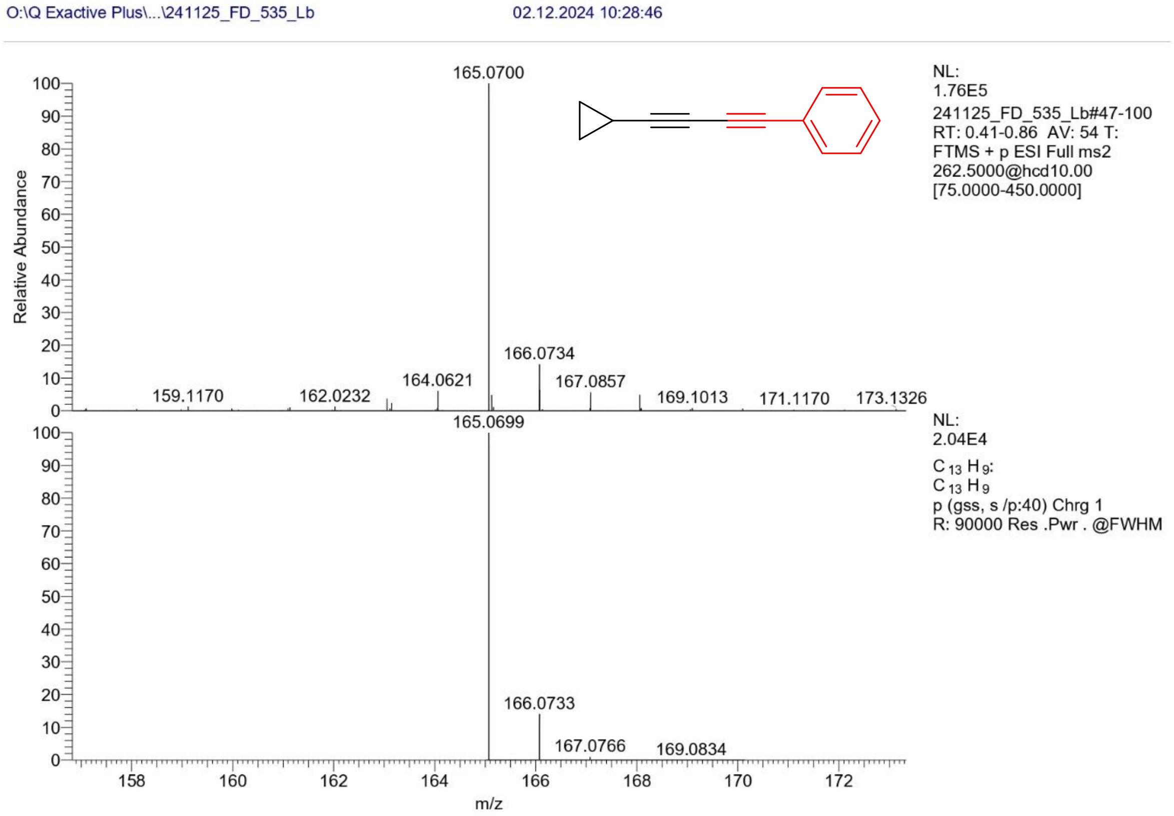

Figure S61. HR-MS spectrum of **23**.

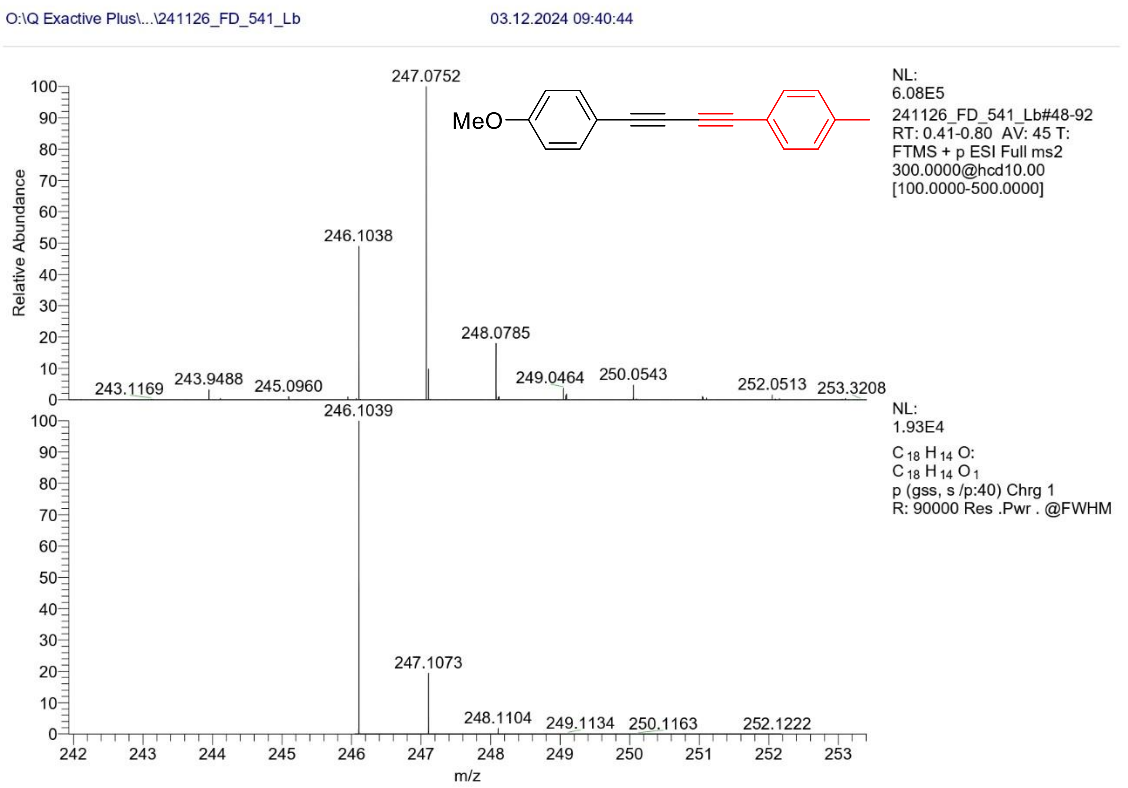

Figure S62. HR-MS spectrum of **24**.

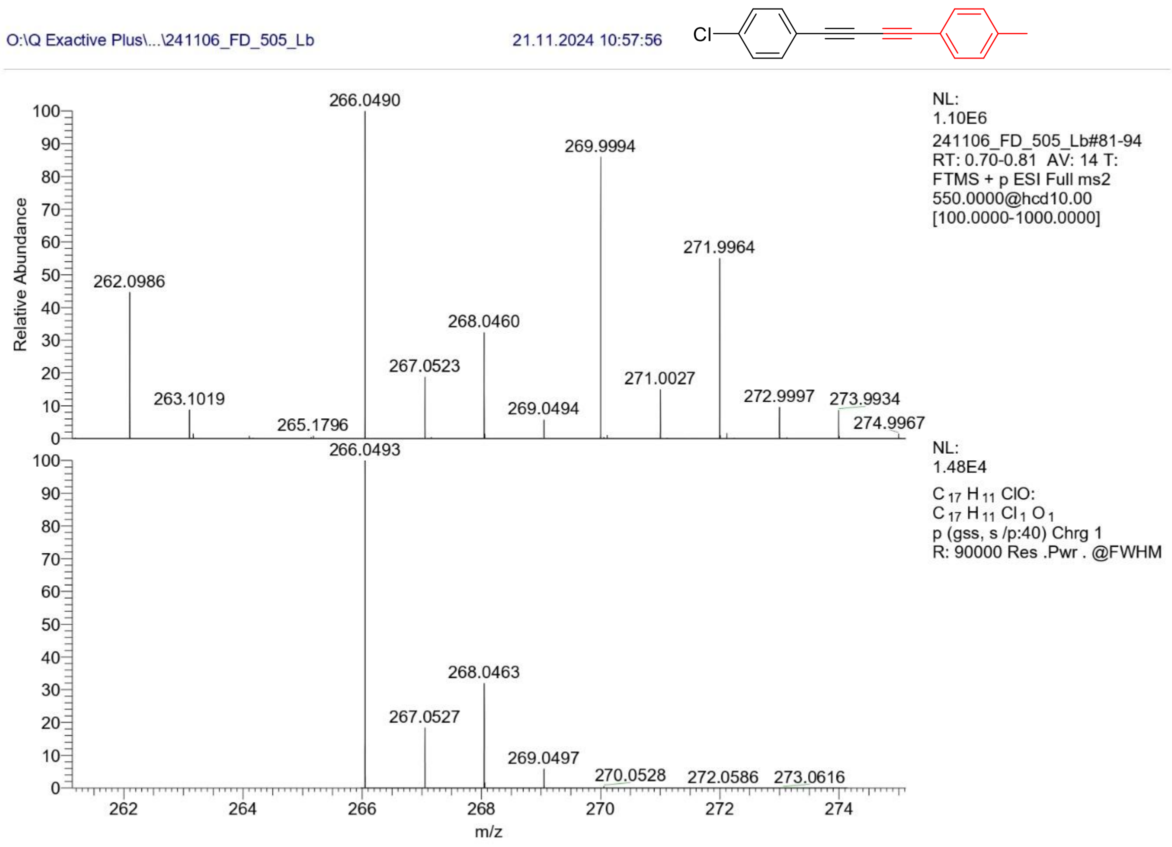

Figure S63. HR-MS spectrum of **25**.

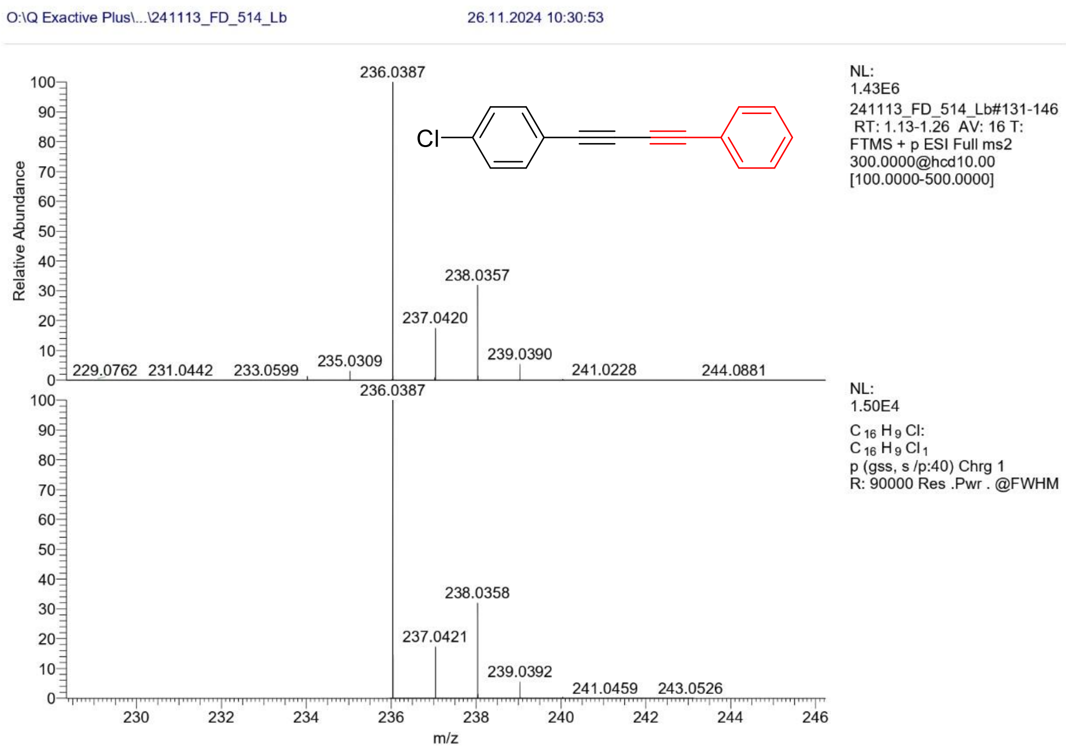

Figure S64. HR-MS spectrum of **26**.

7. Abstraction of (C≡CR)$^{\bullet}$ radicals from Bi(C≡CR)$_3$ with CAAC

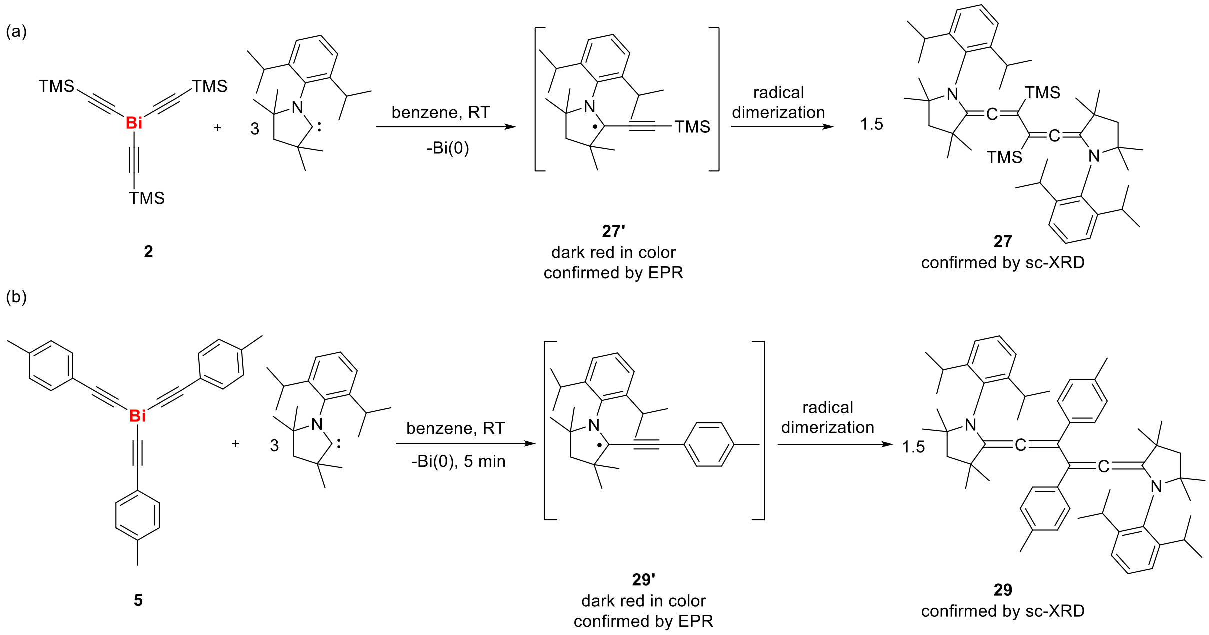

Scheme S5. Generation of short-lived amino-allenyl radicals **27'** and **29'** (confirmed by EPR spectroscopy) and identification of their dimeric forms **27**, **29** by single-crystal X-ray diffraction analyses.

Synthesis of [CAAC-CC-SiMe$_3$]$_2$ (27):

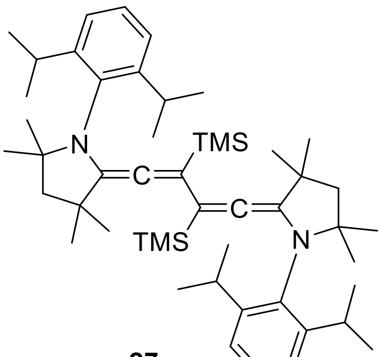

27

Compound Bi(C≡CSiMe$_3$)$_3$ (**2**) (45 mg, 0.09 mmol, 1 equiv.) was added at room temperature to a solution of 3 equivalents of ^{Me2}CAAC (86 mg, 0.27 mmol, 3 equiv.) in THF (5 mL). The color of the solution immediately changed from colorless to a dark red color. This resulting mixture was stirred for 1 h at room temperature after which all volatiles were removed under reduced pressure. The resulting solid was extracted with pentane (2 x 15 mL). The solvent was removed under reduced pressure to give **27** as brownish solid.

^{1}H and ^{13}C NMR spectra of **27** showed multiple resonances with signals in the ^{1}H NMR spectrum, suggesting equilibria between **27** and **27'**.

HR-MS (ESI, pos): [C$_{25}$H$_{40}$NSi]$^+$ calc. [M]$^+$ 383.2952; found 383.2922.

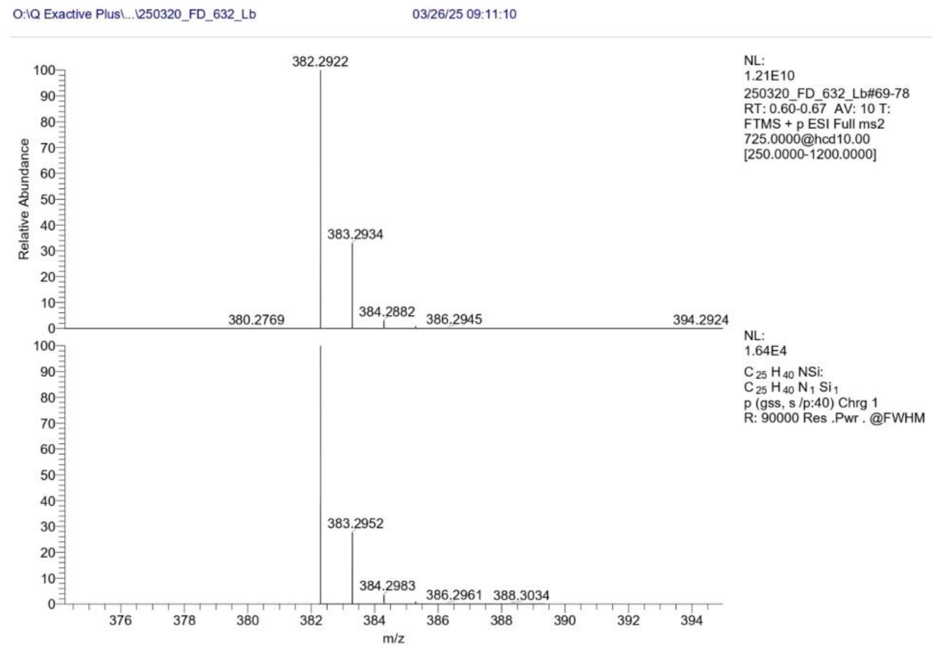

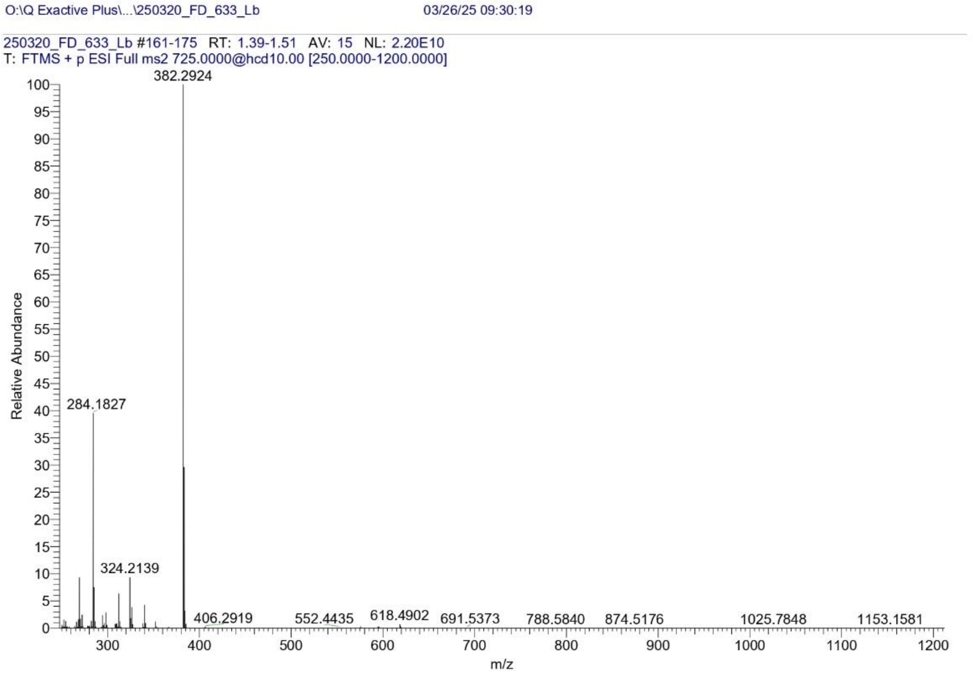

Figure S65. HR-MS spectrum of **27´**.

Synthesis of [CAAC-CC-PhMe]$_2$ (29):

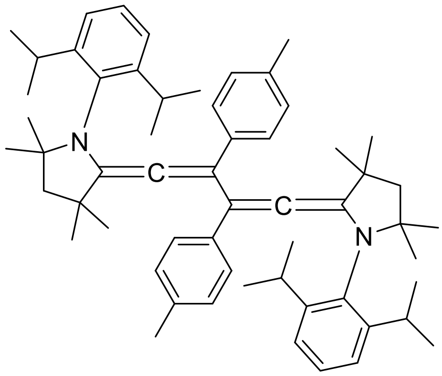

29

Compound Bi(C≡C-*p*-Me-C_6H_4)$_3$ (**5**) (50 mg, 0.09 mmol, 1 eq.) was added at room temperature to a solution of 3 equivalents of ^{Me2}CAAC (86 mg, 0.27 mmol, 3 eq.) in THF (5 mL). The color of the solution immediately changed from colorless to a dark red color. This resulting mixture was stirred for 1 h at room temperature after which all volatiles were removed under reduced pressure. The resulting solid was extracted with pentane (2 x 15 mL). The solvent was removed under reduced pressure to give **29** as brownish solid.

^{1}H and ^{13}C NMR spectra of **29** showed multiple resonances with signals in the ^{1}H NMR spectrum, suggesting equilibria between **29** and **29’**.

HR-MS (ESI, pos): $[C_{29}H_{38}N_1]^+$ calc. $[M]^+$ 400.2999; found 400.2999.

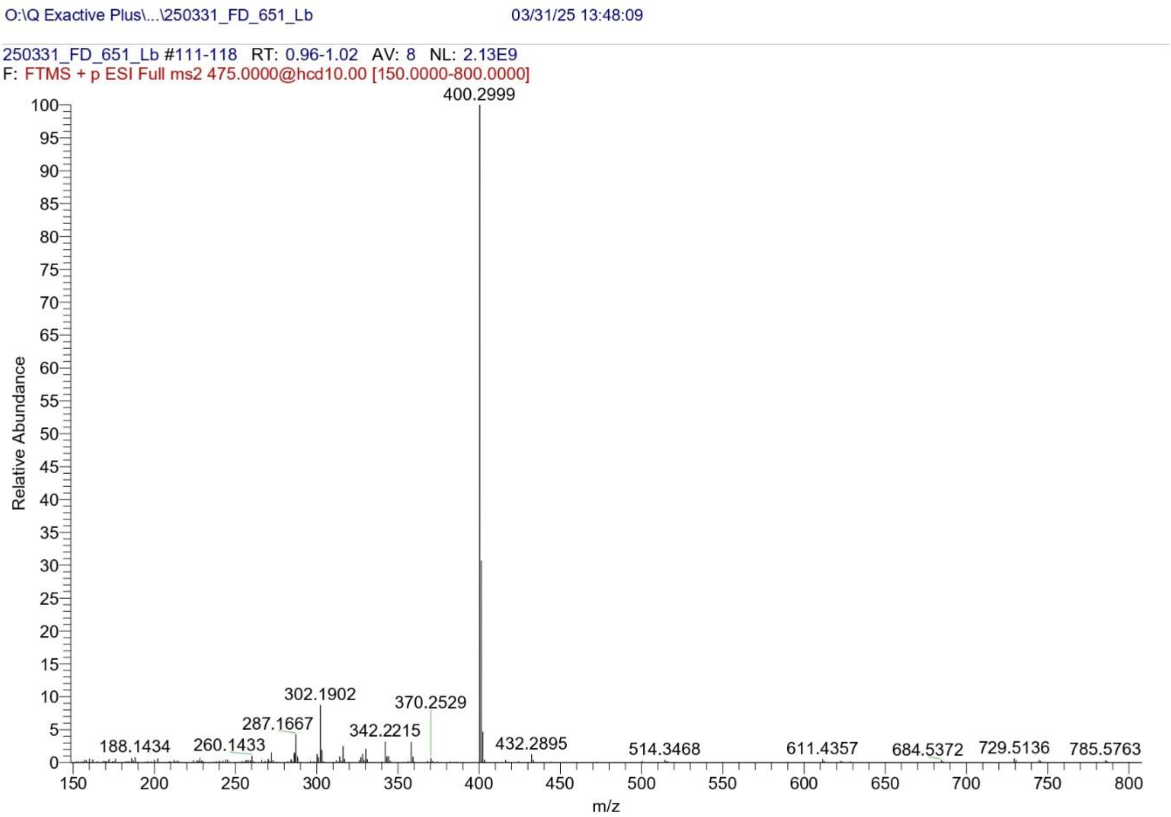

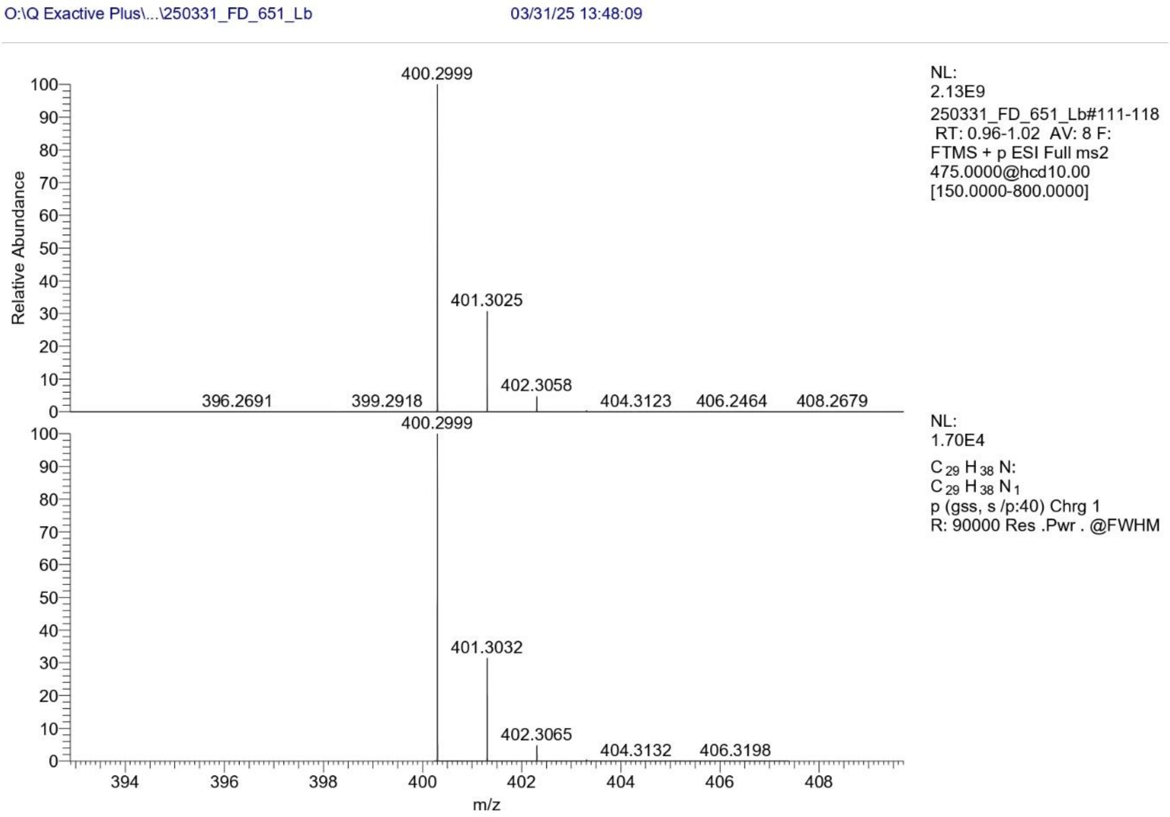

Figure S66. HR-MS spectrum of **29´**.

EPR spectroscopy:

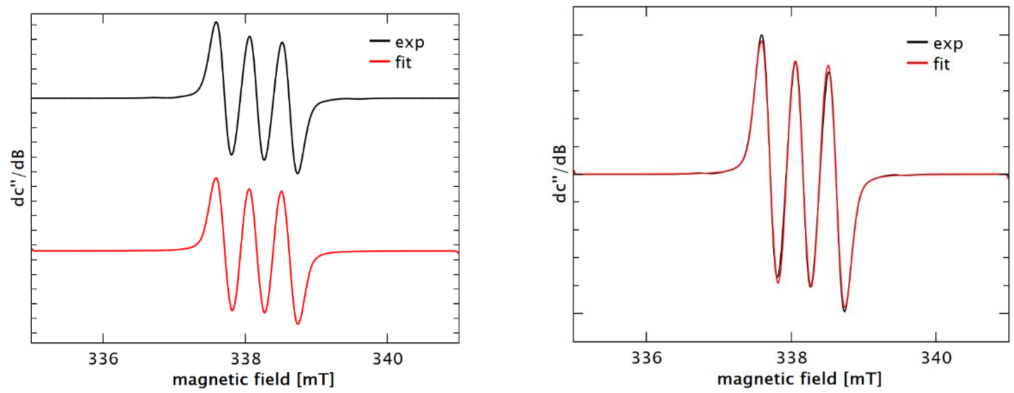

Figure S67. Experimental (black) and simulated (red) continuous-wave (CW) X-band EPR spectra of a solution containing 1 equiv. Bi(C≡CSiMe$_3$)$_3$ (c = 0.6·10^{-2} mol/L) and 3 equiv. CAAC in benzene. The observed resonance shows coupling constants of a(1 × ^{14}N) = 12.7 MHz (4.54 G, 0.454 mT) and a g_{iso} value of 2.0019. Contributions to the spectrum line-width were modelled with unresolved coupling to the protons of two methyl groups with a(3 × ^{1}H) = 2.55 MHz (0.910 G, 0.0910 mT), a(3 × ^{1}H) = 1.47 MHz (0.524 G, 0.0524 mT). Spectrometer settings: microwave frequency = 9.475777 GHz, 0.02 mT modulation amplitude at 100 kHz, microwave power = 0.1 mW, number of accumulated scans = 1, conversion time = 2 ms.

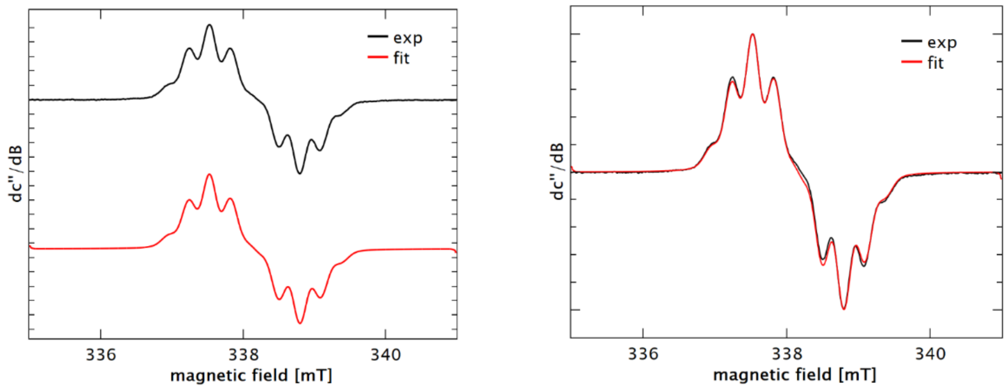

Figure S68. Experimental (black) and simulated (red) continuous-wave (CW) X-band EPR spectra of a solution containing 1 equiv. Bi(C≡C(*p*Tol))$_3$ (c = 5.4·10^{-3} mol/L) and 3 equiv. CAAC in benzene. The observed resonance shows coupling constants of a(1 × ^{14}N) = 11.8 MHz (4.21 G, 0.421 mT), a(2 × ^{1}H) = 7.65 MHz (2.73 G, 0.273 mT), a(3 × ^{1}H) = 7.54 MHz (2.69 G, 0.269 mT), a(2 × ^{1}H) = 2.63 MHz (0.939 G, 0.0939 mT), and a g_{iso} value of 2.0019. Contributions to the spectrum line-width were modelled with unresolved coupling to the protons of two methyl groups with a(3 × ^{1}H) = 1.58 MHz (0.566 G, 0.0566 mT), a(3 × ^{1}H) = 1.51 MHz (0.540 G, 0.0540 mT), Spectrometer settings: microwave frequency = 9.475791 GHz, 0.04 mT modulation amplitude at 100 kHz, microwave power = 2 mW, number of accumulated scans = 10, conversion time = 2 ms.

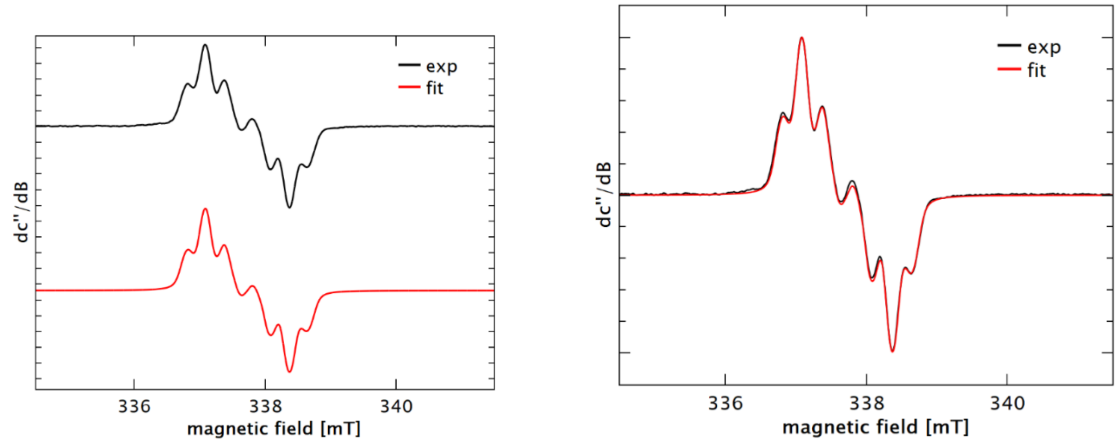

Figure S69. Experimental (black) and simulated (red) continuous-wave (CW) X-band EPR spectra of a solution containing 1 equiv. $Bi(CCPh)_3$ (c = $1.2\cdot10^{-2}$ mol/L) and 3 equiv. CAAC in benzene. The observed resonance shows coupling constants of a(1 × ^{14}N) = 11.9 MHz (4.24 G, 0.424 mT), a(1 × ^{1}H) = 6.89 MHz (2.46 G, 0.246 mT), a(2 × ^{1}H) = 7.26 MHz (2.59 G, 0.259 mT), a(2 × ^{1}H) = 2.81 MHz (1.00 G, 0.100 mT), and a g_{iso} value of 2.0018. Contributions to the spectrum line-width were modelled with unresolved coupling to the protons of two methyl groups with a(3 × ^{1}H) = 1.23 MHz (0.440 G, 0.0440 mT), a(3 × ^{1}H) = 1.28 MHz (0.459 G, 0.0459 mT), Spectrometer settings: microwave frequency = 9.463368 GHz, 0.02 mT modulation amplitude at 100 kHz, microwave power = 1 mW, number of accumulated scans = 1, conversion time = 2 ms. Experiments with THF as the solvent gave qualitatively identical results. This EPR spectrum is in good agreement with the previous results from the group of Bertrand.[60]

8. Reactions with THF and benzene

Radical C–H activation of benzene: In an argon-filled glovebox, a J. Young NMR tube was charged with Bi(C≡CPh)$_3$ (**4**) (20 mg, 0.04 mol) and benzene (0.5 mL). The solution was irradiated with a blue LED (460 nm) for 56 hours, during which a black precipitate of Bi(0) was observed. The colorless solution was filtered to remove the precipitate and subsequently used for further characterization. The formation of diphenylacetylene was confirmed by GC–MS and HR-MS analysis. GC-MS analysis showed a yield of ca. 40% of diphenylacetylene.

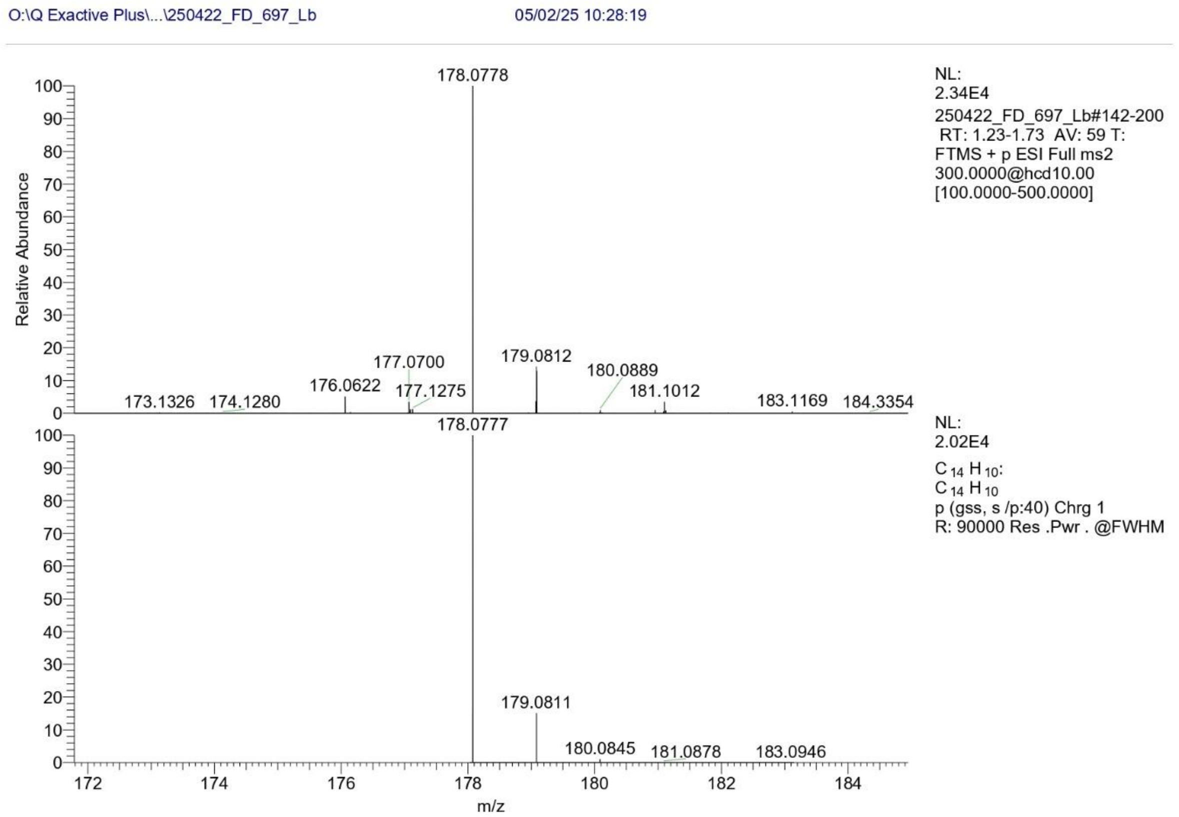

Figure S70. HR-MS spectrum of diphenylacetylene (**30**) obtained from C–H alkynylation of benzene.

Radical C–H activation of THF: Compound Bi(C≡CPh)$_3$ (**4**) can contain THF molecules that show weak interactions with the molecular complex (as observed in the molecular structure obtained from single-crystal XRD data). In cross-coupling reactions, we have in some cases observed the respective α-alkynylated THF product as a minor species (ca. 5-10%) along with the formation of free alkyne in equal amounts, especially when longer reaction times and higher reaction temperatures were necessary (e.g. heating solutions that contain **4** up to 80 °C for 24 h or more). An NMR spectrum of a reaction mixture containing the α-alkynylated THF product is shown in Figure S56.

9. Synthesis of alkynyl chalcogenides (E = Se, Te)

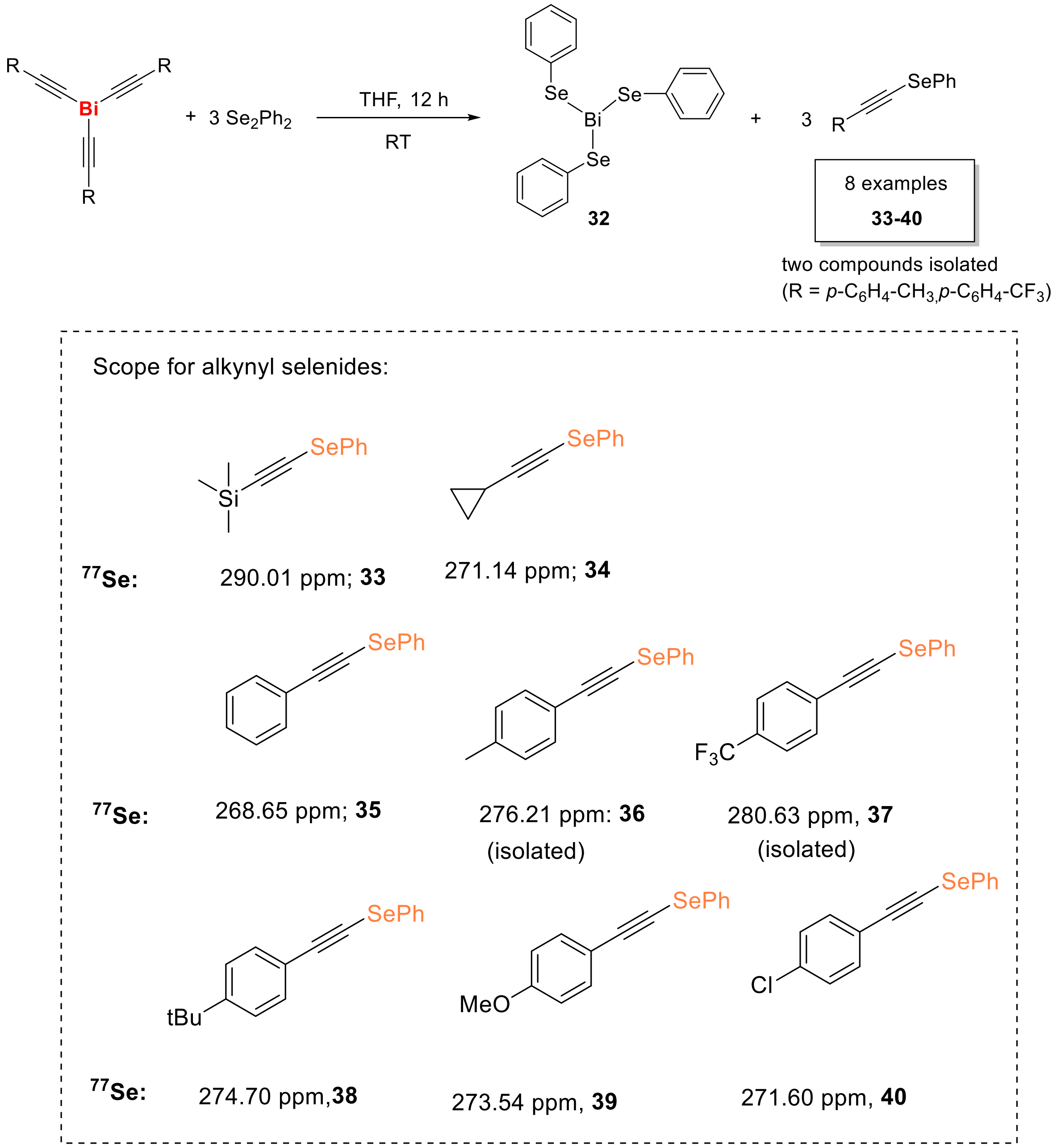

Scheme S6. Synthesis of alkynyl seleno ethers **(**RC≡C–SePh).

General procedure for the synthesis of alkynyl seleno ethers:

In an argon-filled glovebox, a 20 ml vial was loaded with Bi(C≡CR)$_3$ (0.0025 mmol, 1.00 equiv.), Se_2Ph_2 (0.750 mmol, 3.0 equiv.) and THF (3 ml). The reaction mixture was stirred at room temperature for 12 hours. After the reaction was complete, the reaction mixture was dried and the product RC≡C–SePh was extracted with hexane. After filtration and removal of all volatiles under reduced pressure, the desired product was obtained and identified by NMR spectroscopy and mass spectrometry. Notably, the orange-colored precipitate was isolated as Bi(SePh)$_3$ (confirmed by the XRD analysis and NMR spectroscopy). Bi(SePh)$_3$ has previously

been reported and our data is in agreement with the literature.[84] The final alkynyl aryl selenother products RC≡C–SePh were dried, isolated as solids and characterized by ^{77}Se NMR spectroscopy and mass spectrometry. The ^{1}H and ^{13}C NMR spectra of compounds **33**, **35**, **36**, **37**, **39**, and **40** are in good agreement with the literature.[64,75,100] Compounds **34** and **38** have not previously been reported. For compounds **36** and **37**, full characterization data including single-crystal X-ray analyses have been collected.

Representative NMR spectra and mass spectra of the alkynyl chalcogenides (E= Se) (33-40).

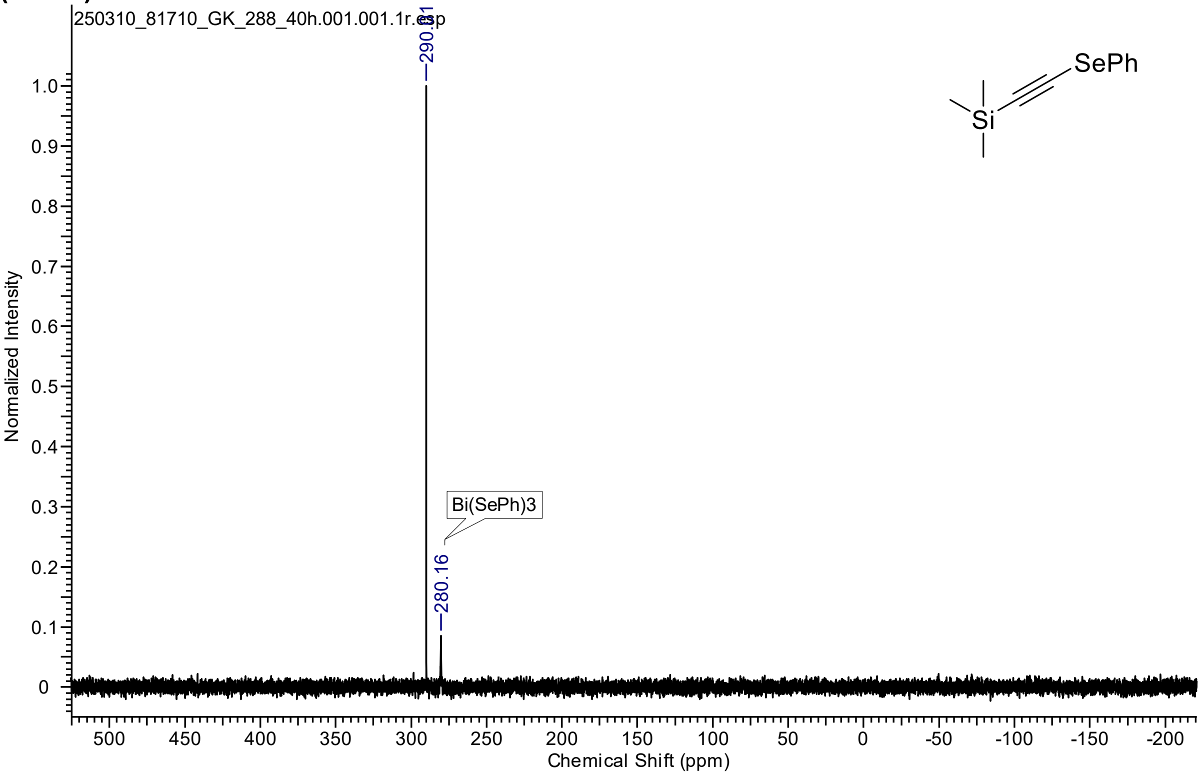

Figure S71. ^{77}Se NMR spectrum of **33** in THF.

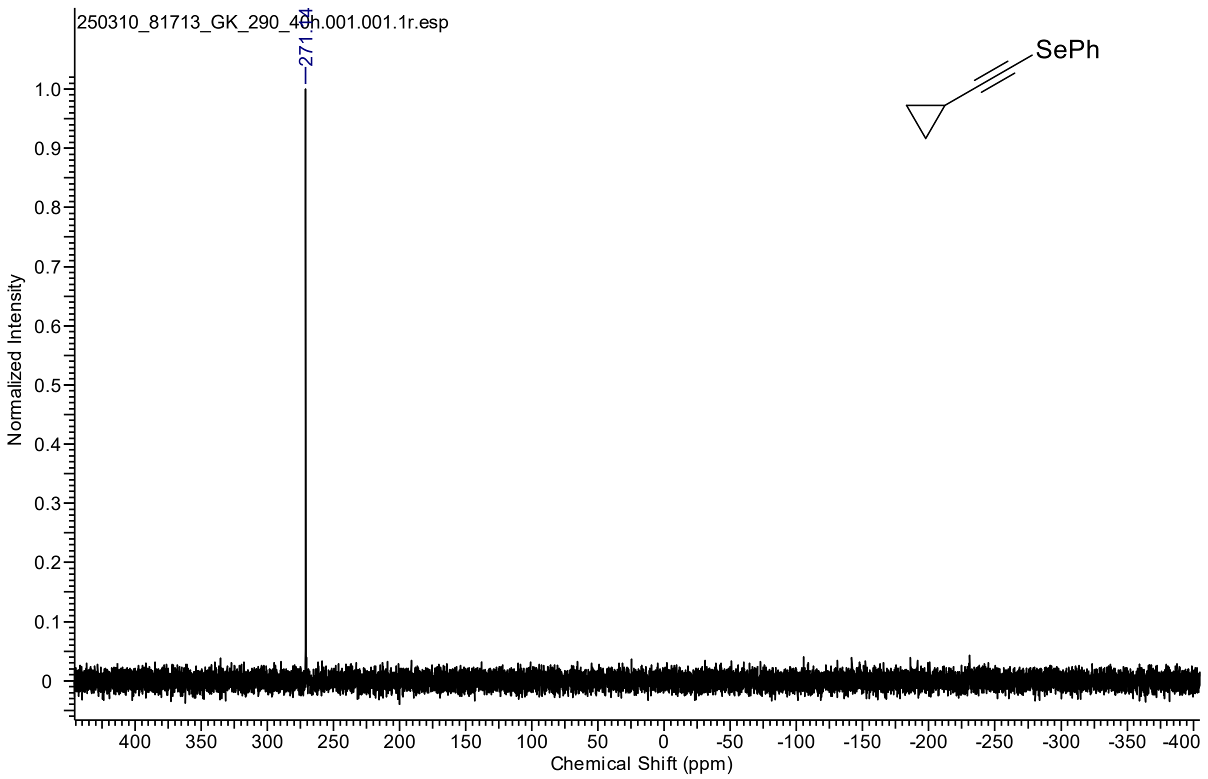

Figure S72. ^{77}Se NMR spectrum of **34** in THF.

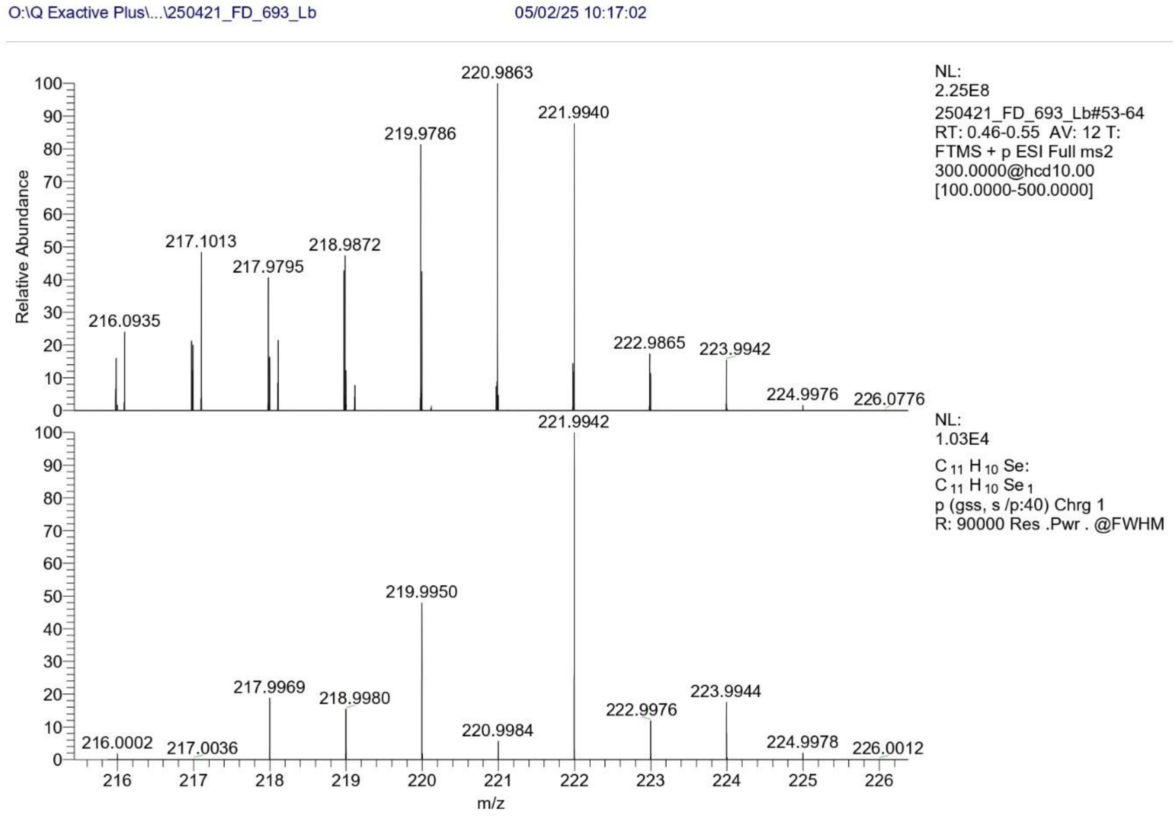

Figure S73. HR-MS spectrum of **34**.

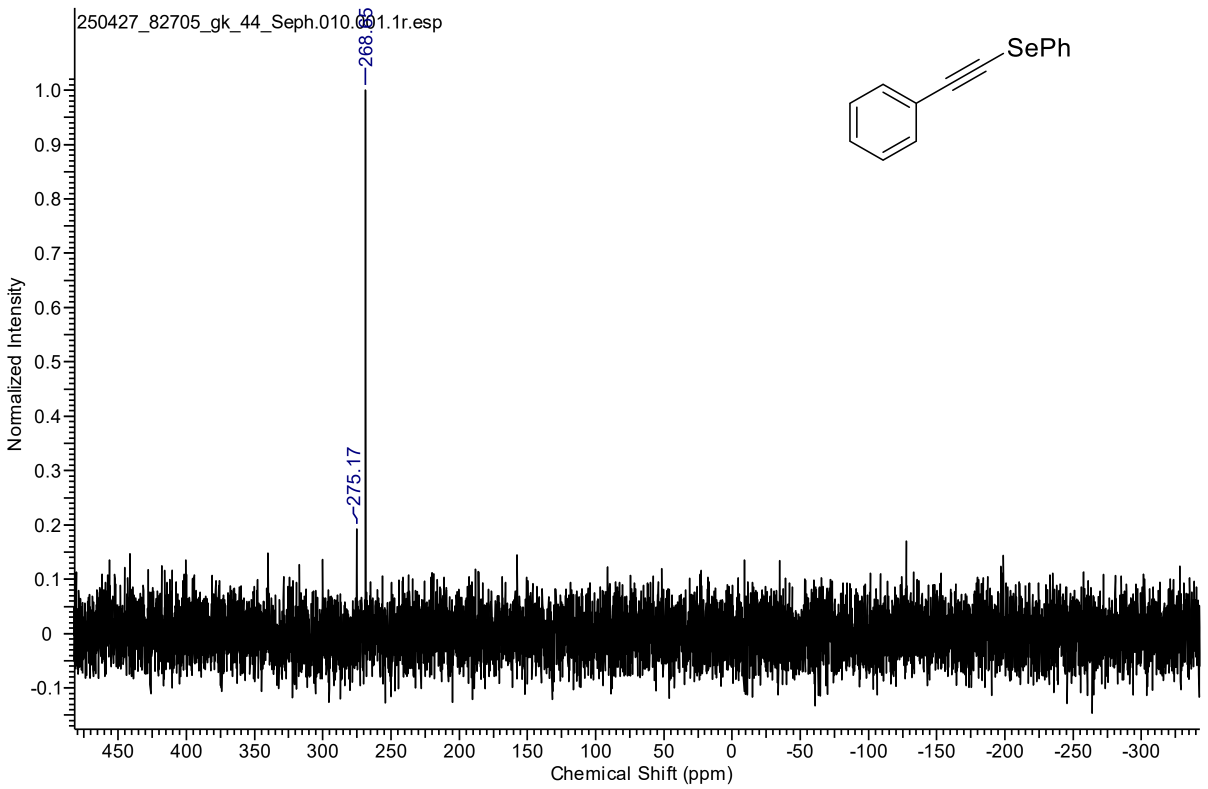

Figure S74. ^{77}Se NMR spectrum of **35** in THF.

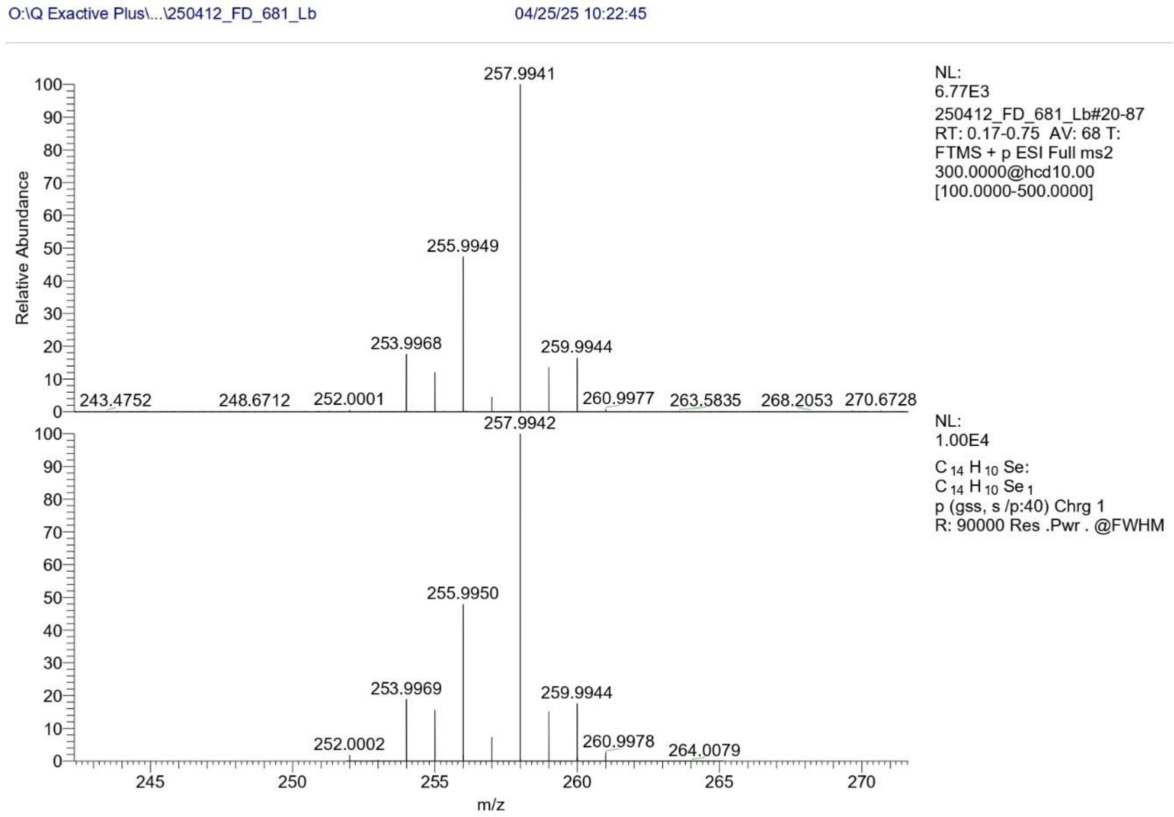

Figure S75. HR-MS spectrum of **35**.

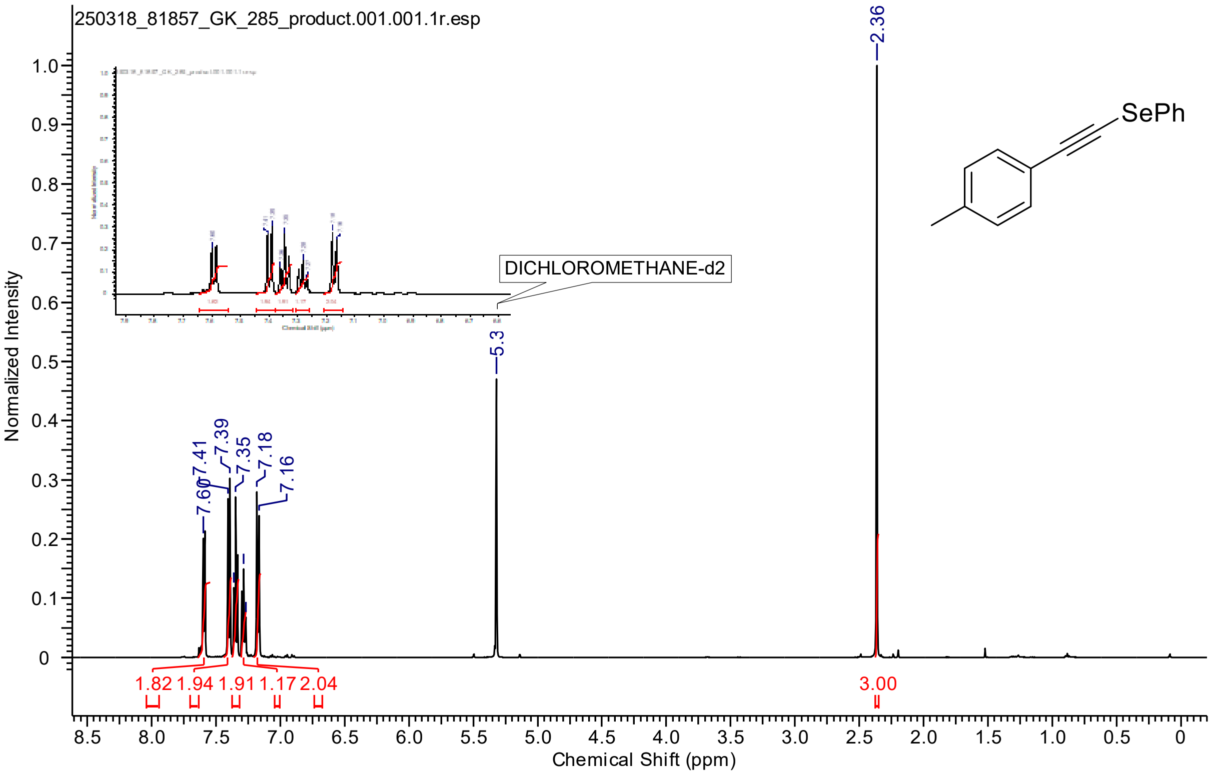

Figure S76. ^{1}H NMR spectrum of **36** in CD_2Cl_2.

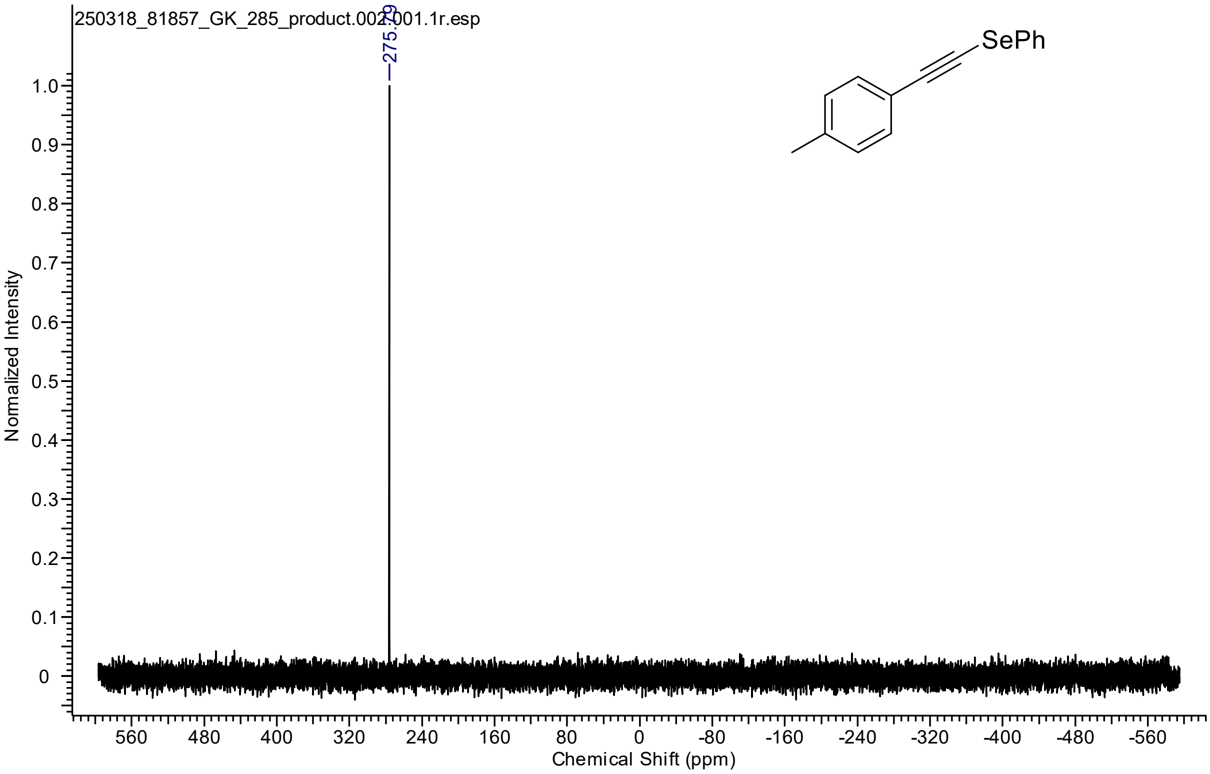

Figure S77. ^{77}Se NMR spectrum of **36** in CD_2Cl_2.

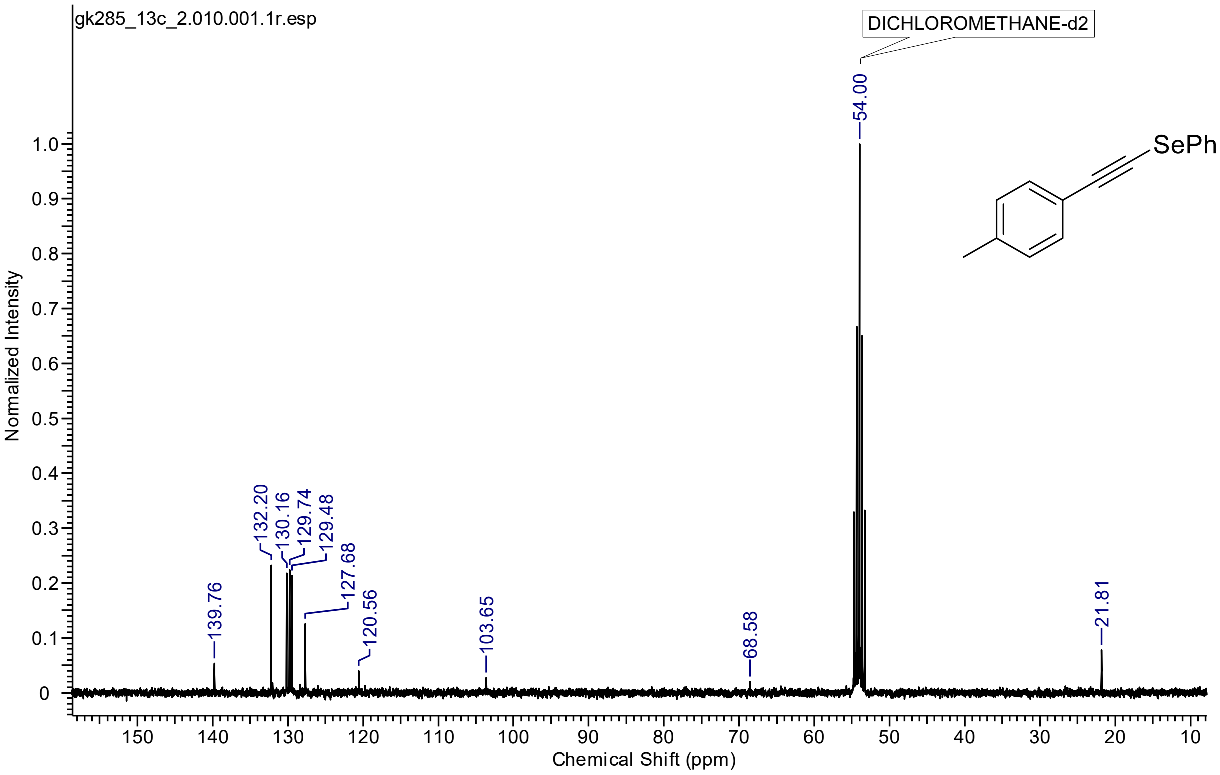

Figure S78. ^{13}C NMR spectrum of **36** in CD_2Cl_2.

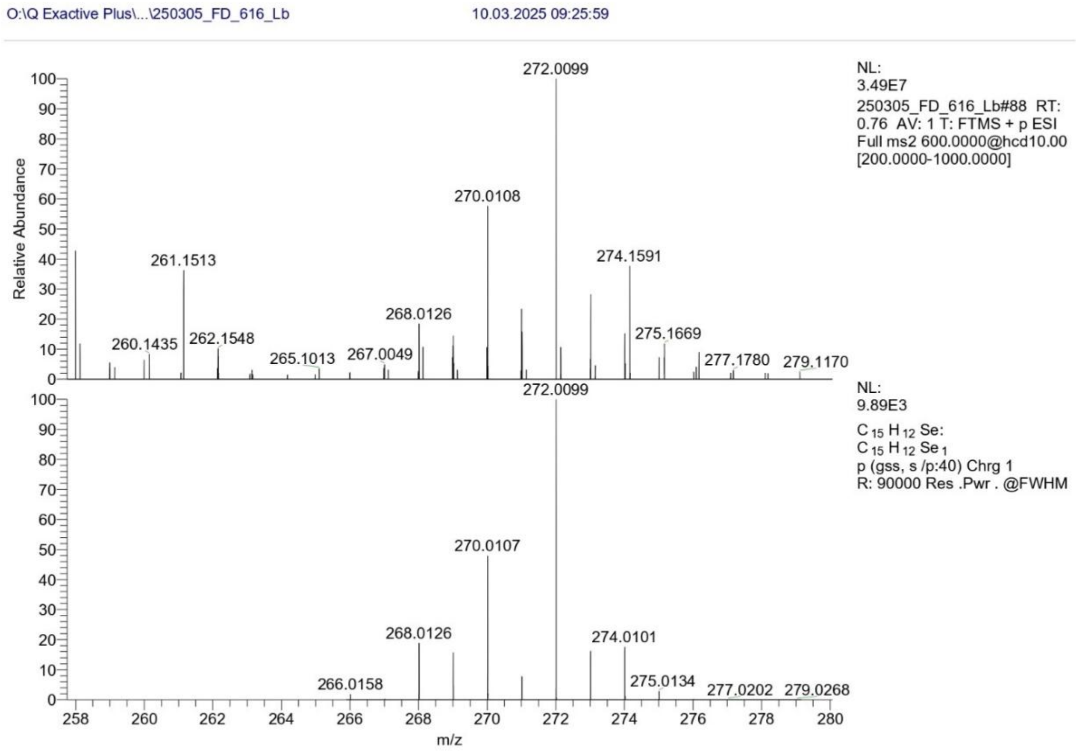

Figure S79. HR-MS spectrum of **36**.

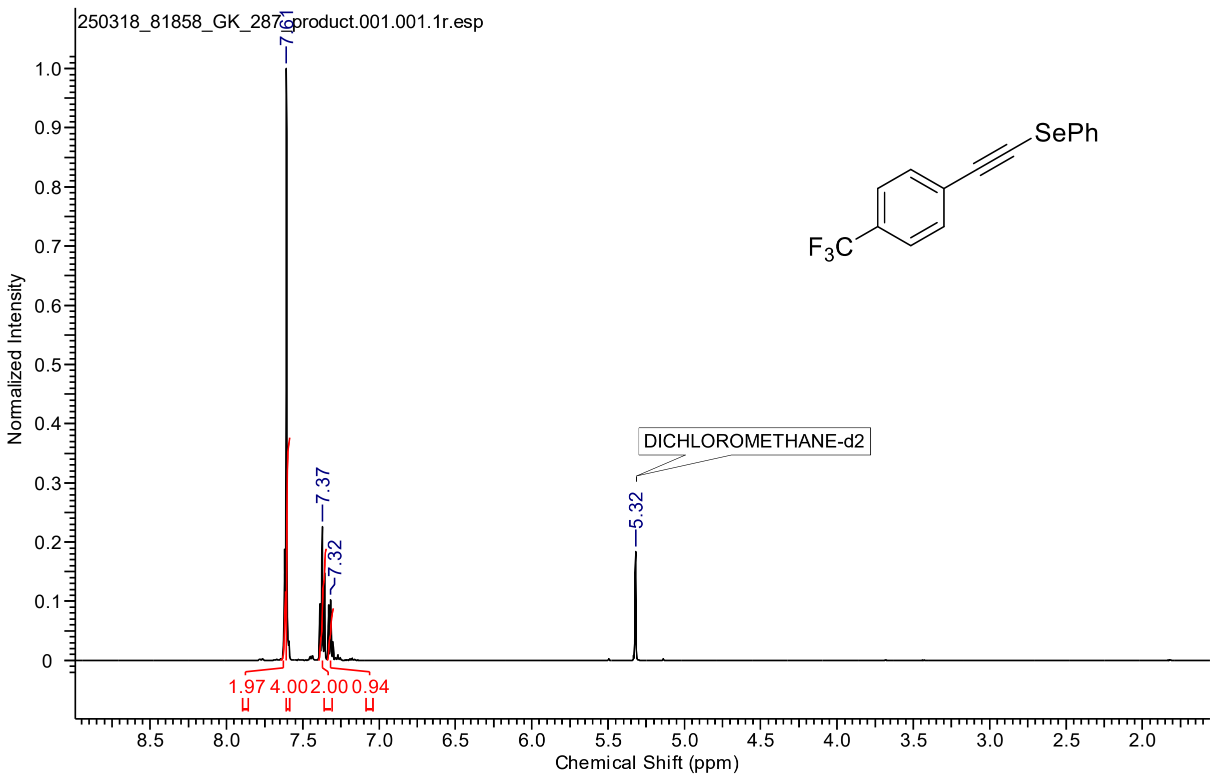

Figure S80. ^{1}H NMR spectrum of **37** in CD_2Cl_2.

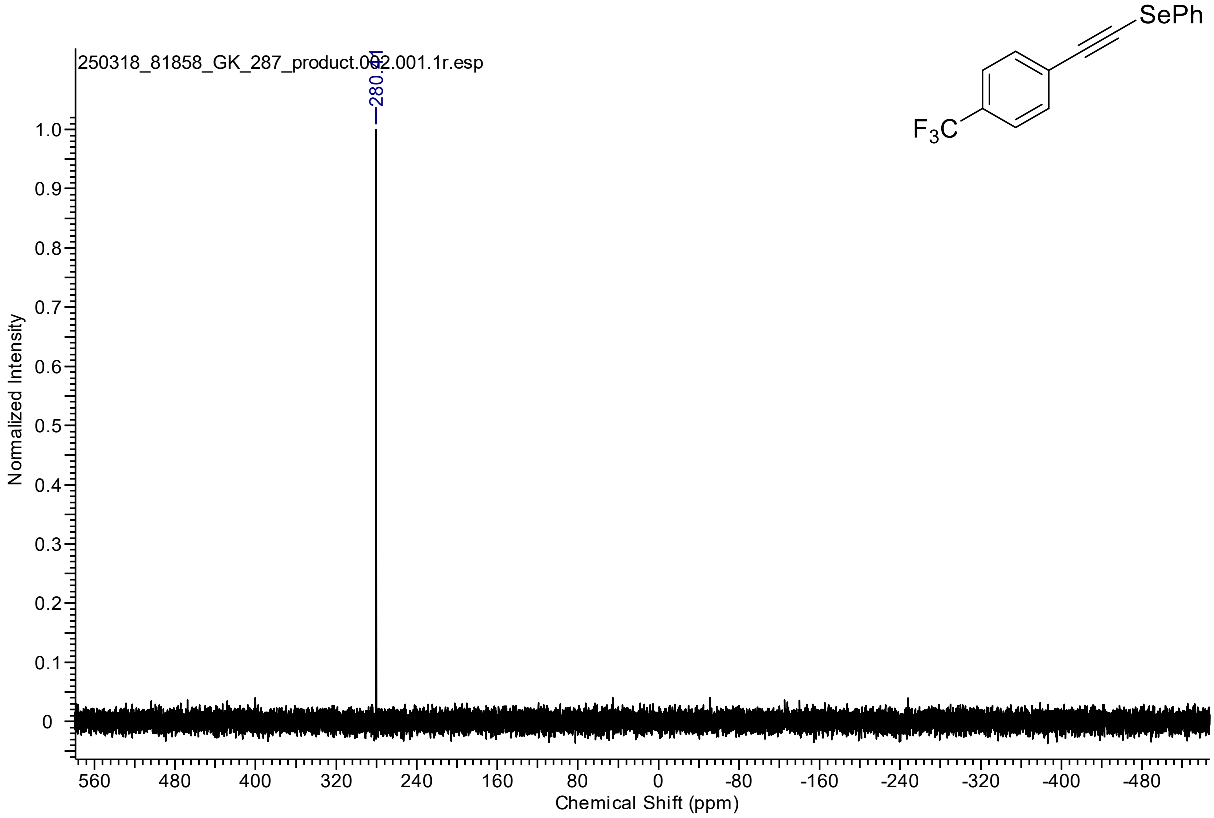

Figure S81. ^{77}Se NMR spectrum of **37** in CD_2Cl_2.

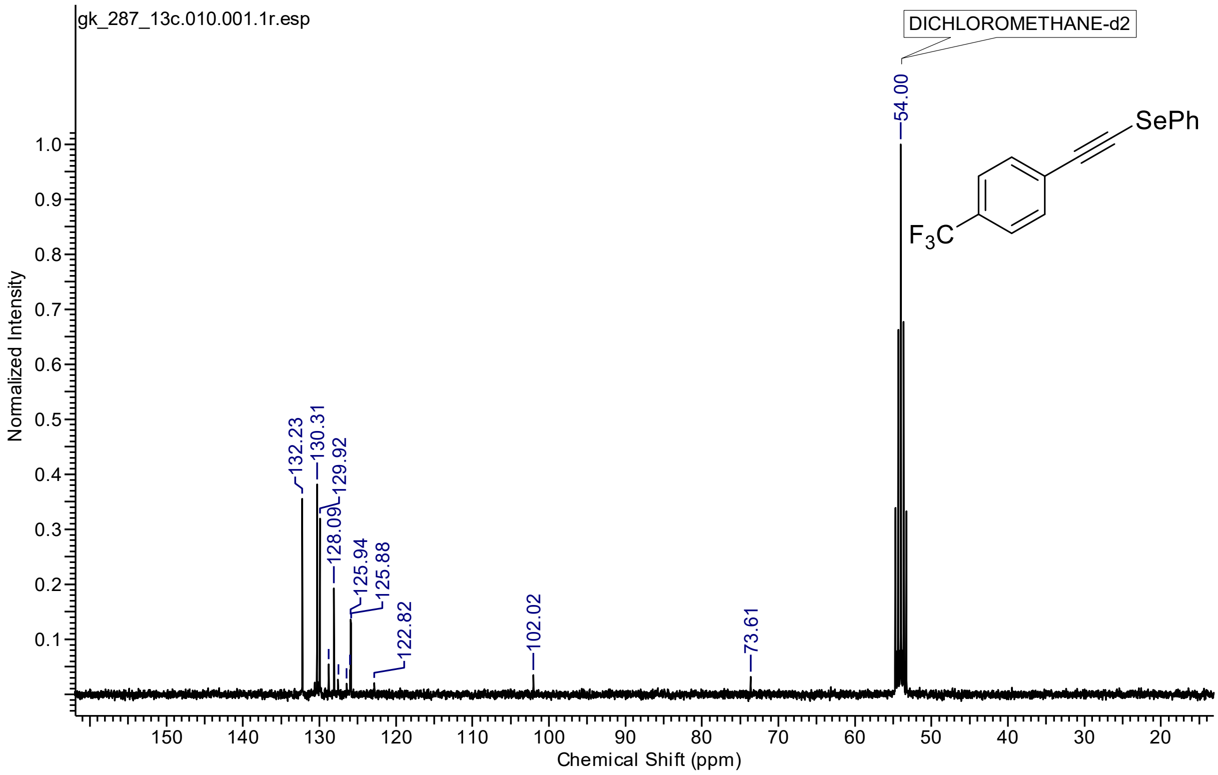

Figure S82. ^{13}C NMR spectrum of **37** in CD_2Cl_2.

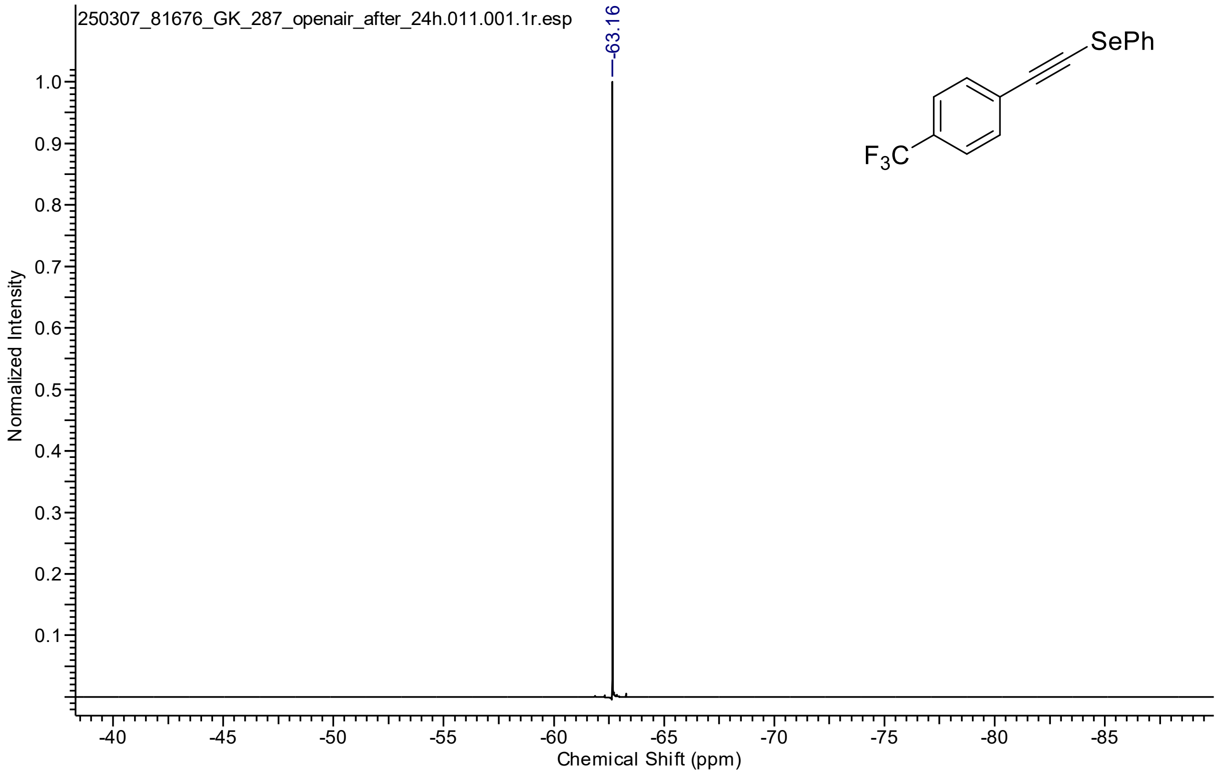

Figure S83. ^{19}F NMR spectrum of **37** in CD_2Cl_2.

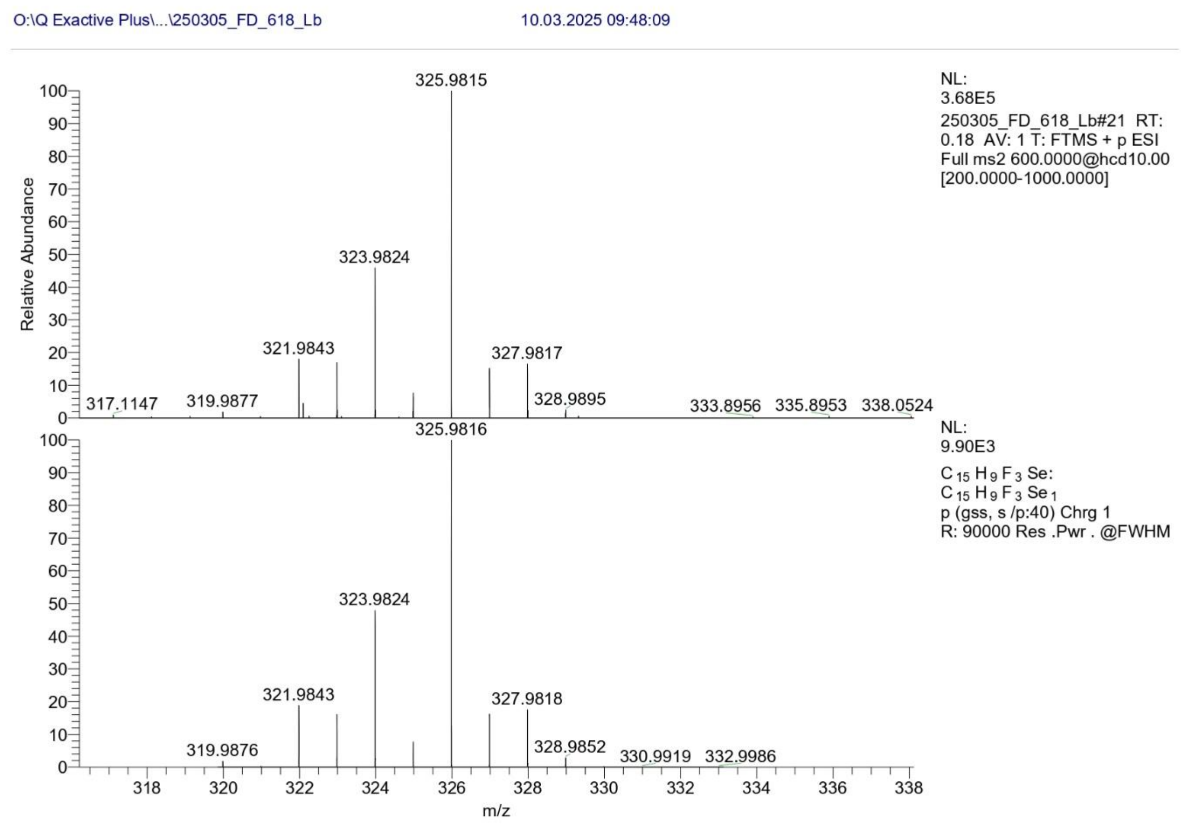

Figure S84. HR-MS spectrum of **37**.

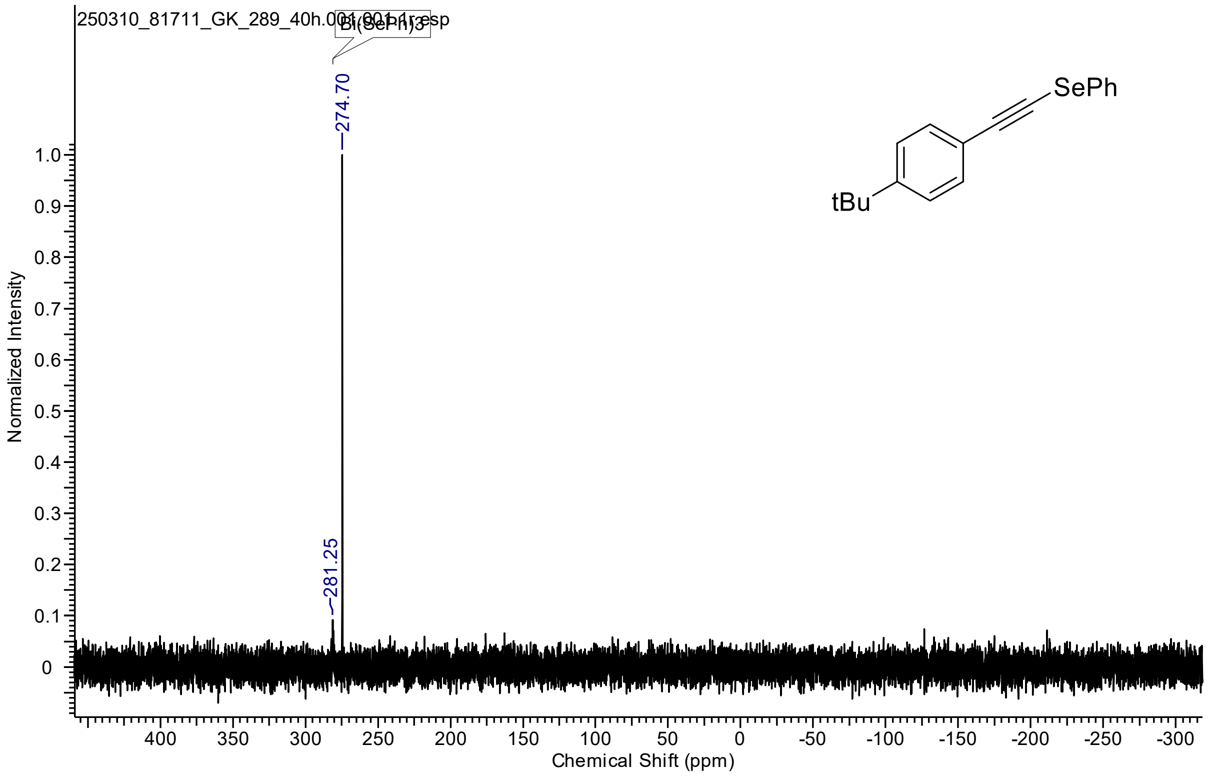

Figure S85. ^{77}Se NMR spectrum of **38** in THF.

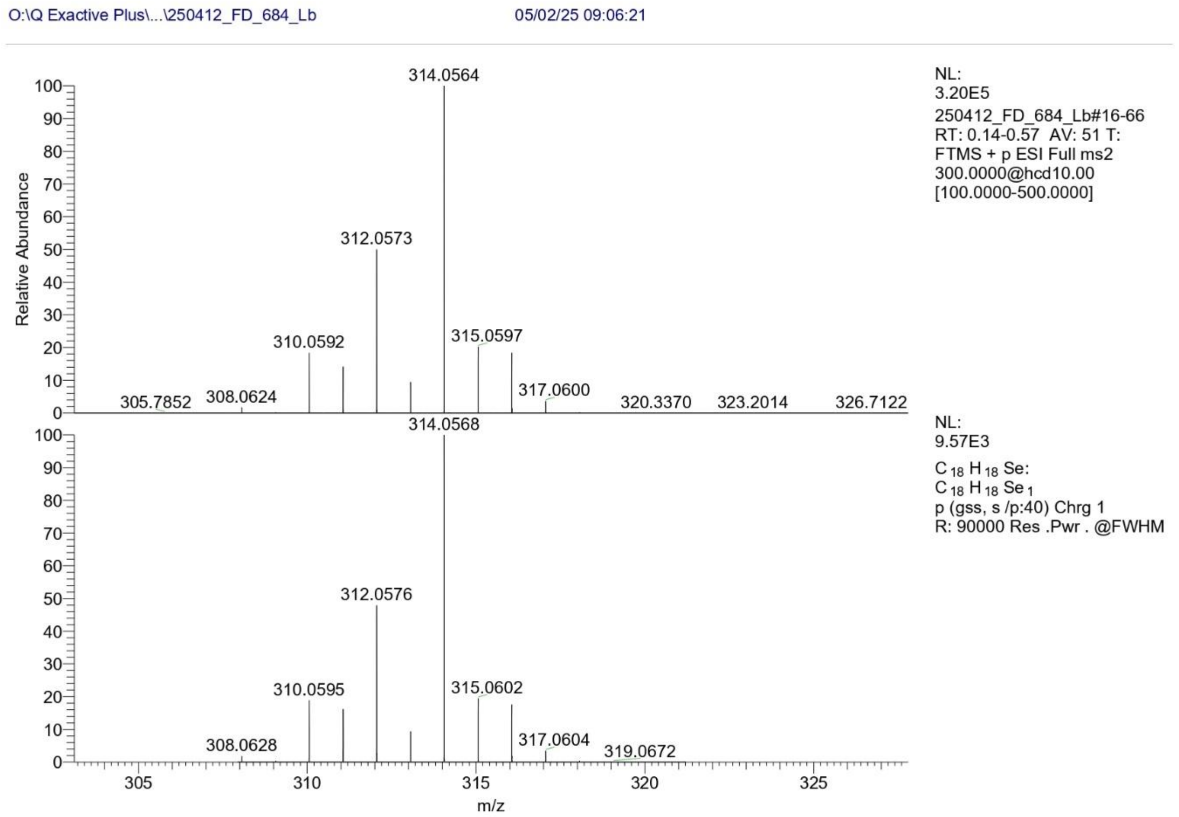

Figure S86. HR-MS spectrum of **38**.

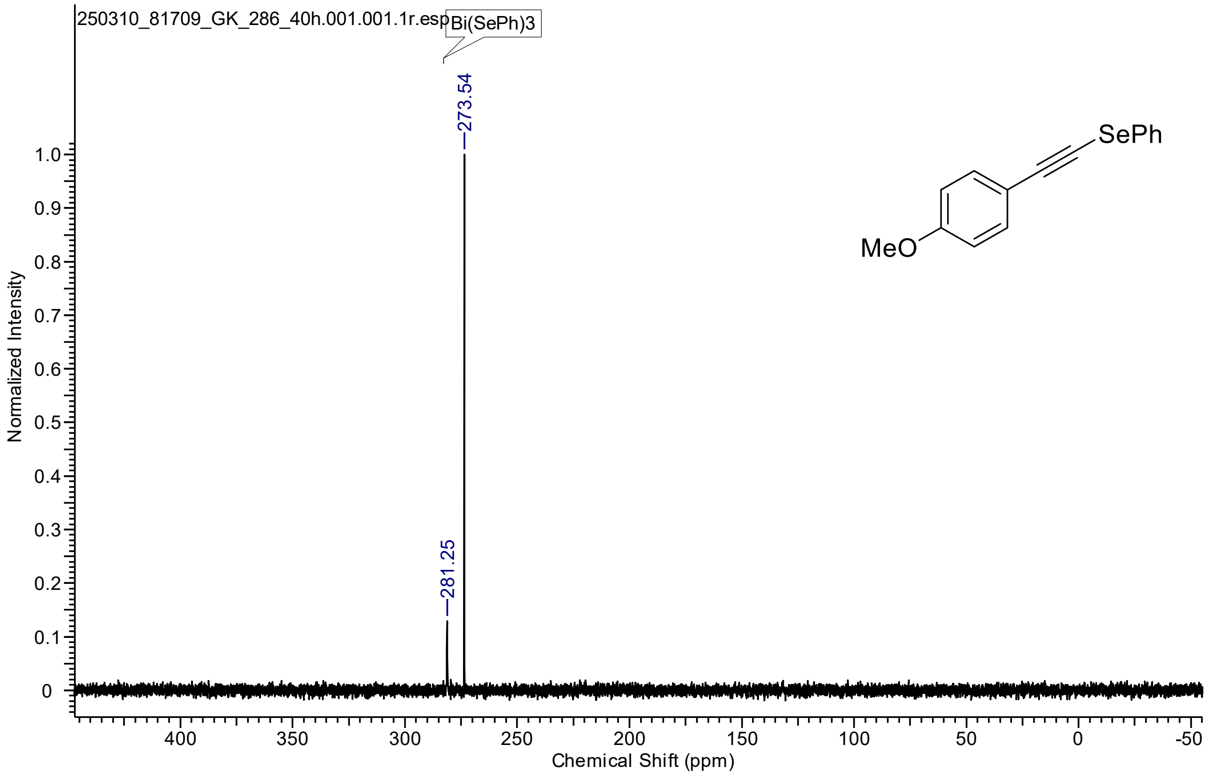

Figure S87. ^{77}Se NMR spectrum of **39** in THF.

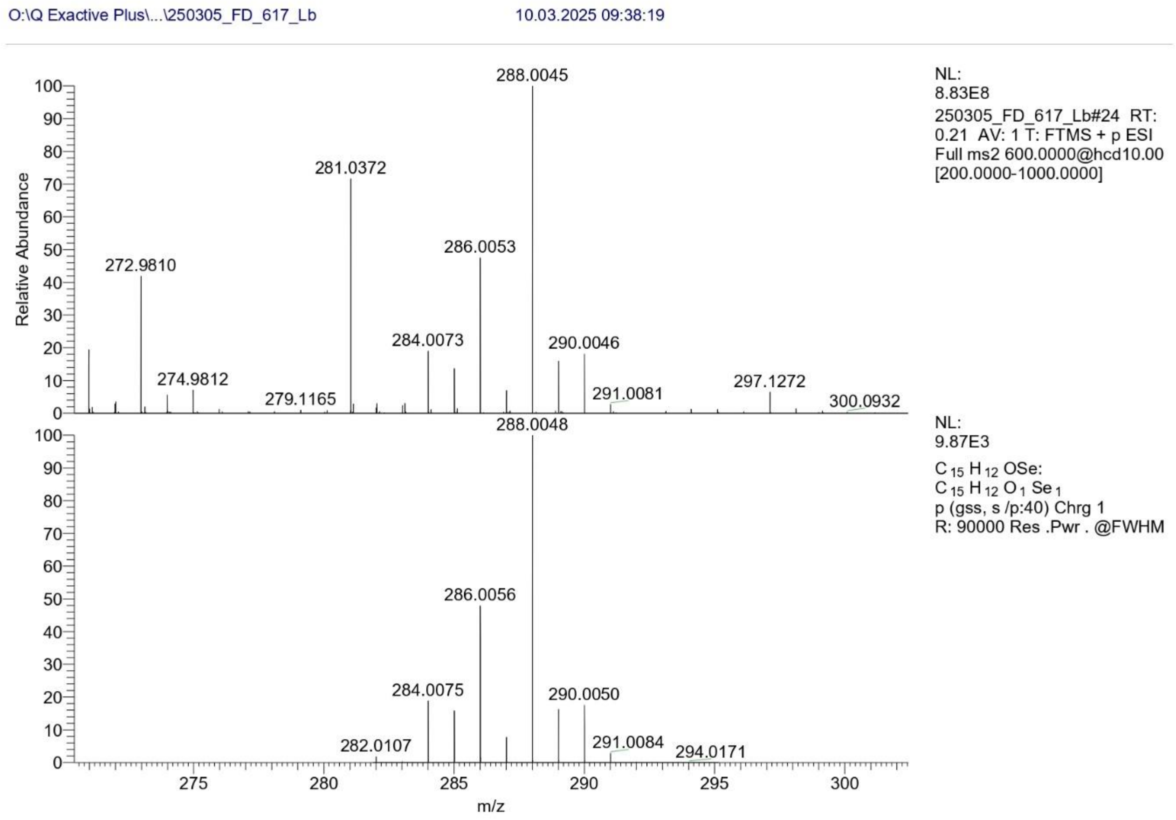

Figure S88. HR-MS spectrum of **39**.

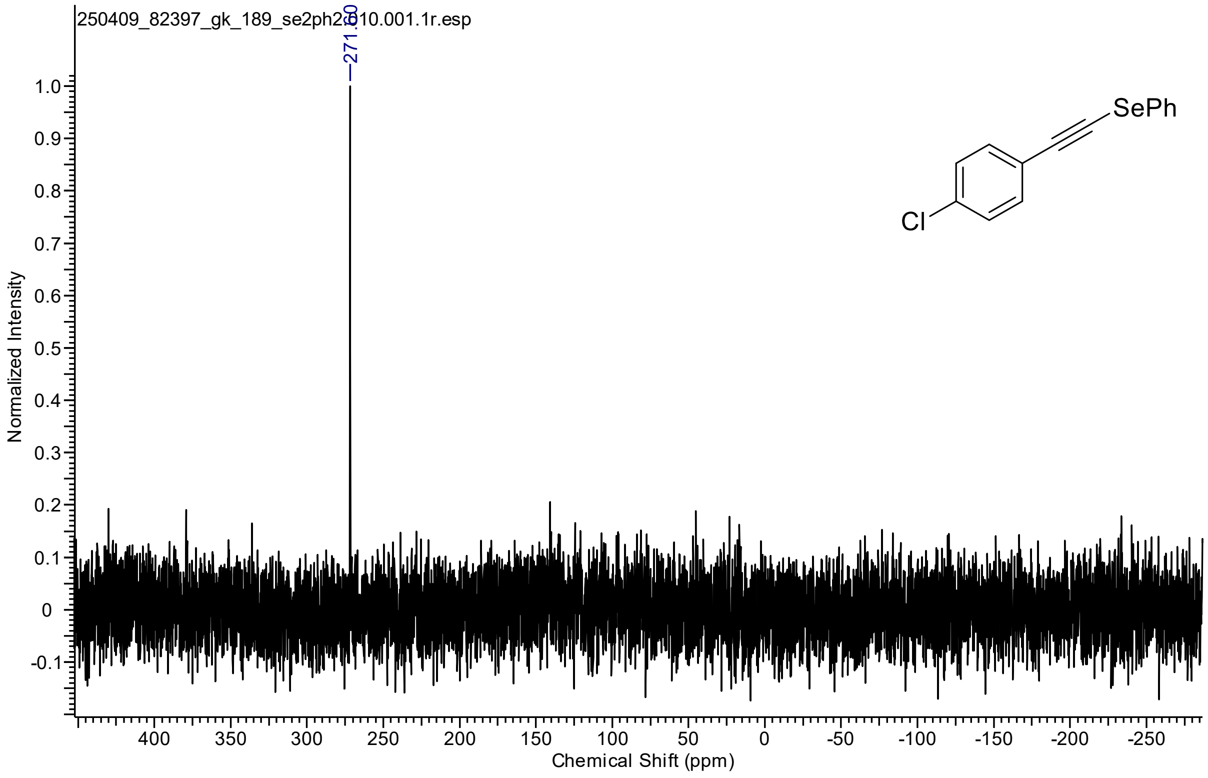

Figure S89. ^{77}Se NMR spectrum of **40** in CD_2Cl_2 (isolated).

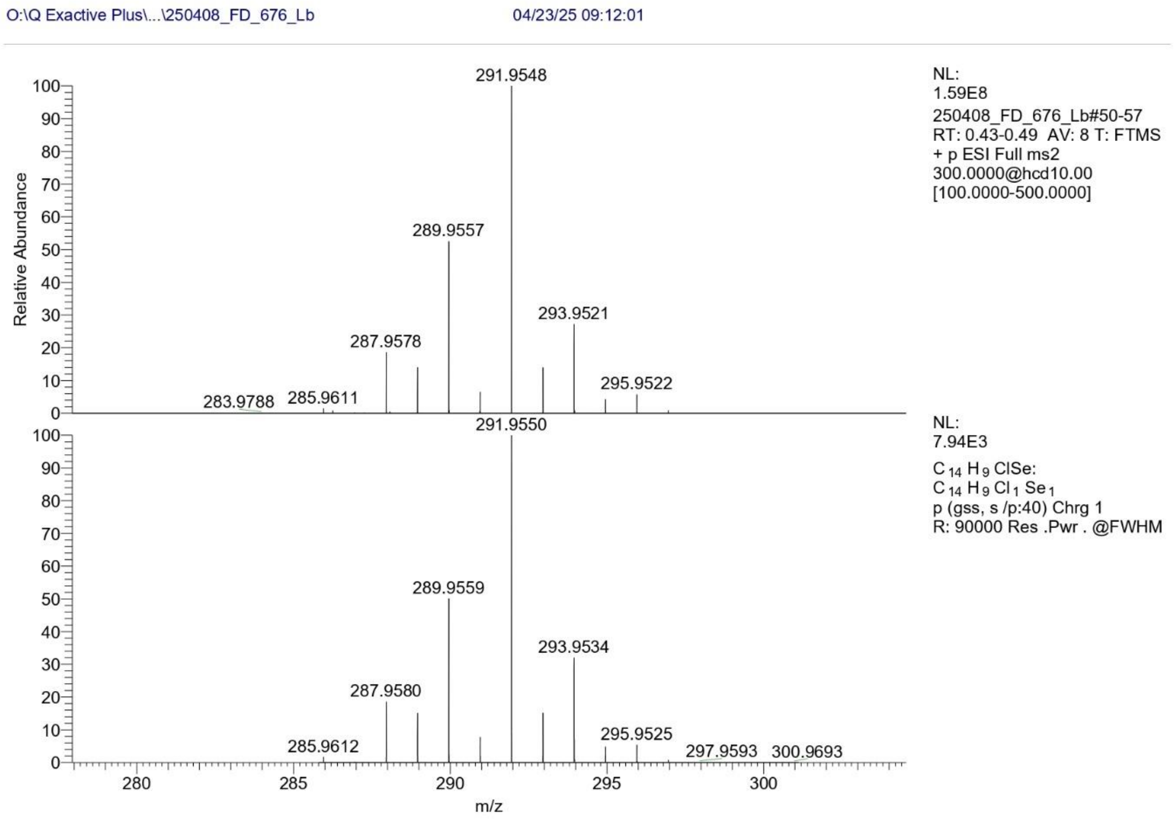

Figure S90. HR-MS spectrum of **40**.

Reacting Bi(C≡CPh)$_3$ (**4**) with three equivalents of $(SePh)_2$ in the presence of TEMPO (2,2,6,6-tetramethyl-piperidinyl-1-oxyl) gave evidence of the formation of Bi(SePh)$_2$(TEMPO), detected as m/z = 679.0295 (agrees with [Bi(SePh)$_2$(TEMPO)]$^{\bullet+}$) by high-resolution mass spectrometric analysis, which would be in congruency with the formation of a trapped intermediate [Bi(SePh)$_2$]$^{\bullet}$.

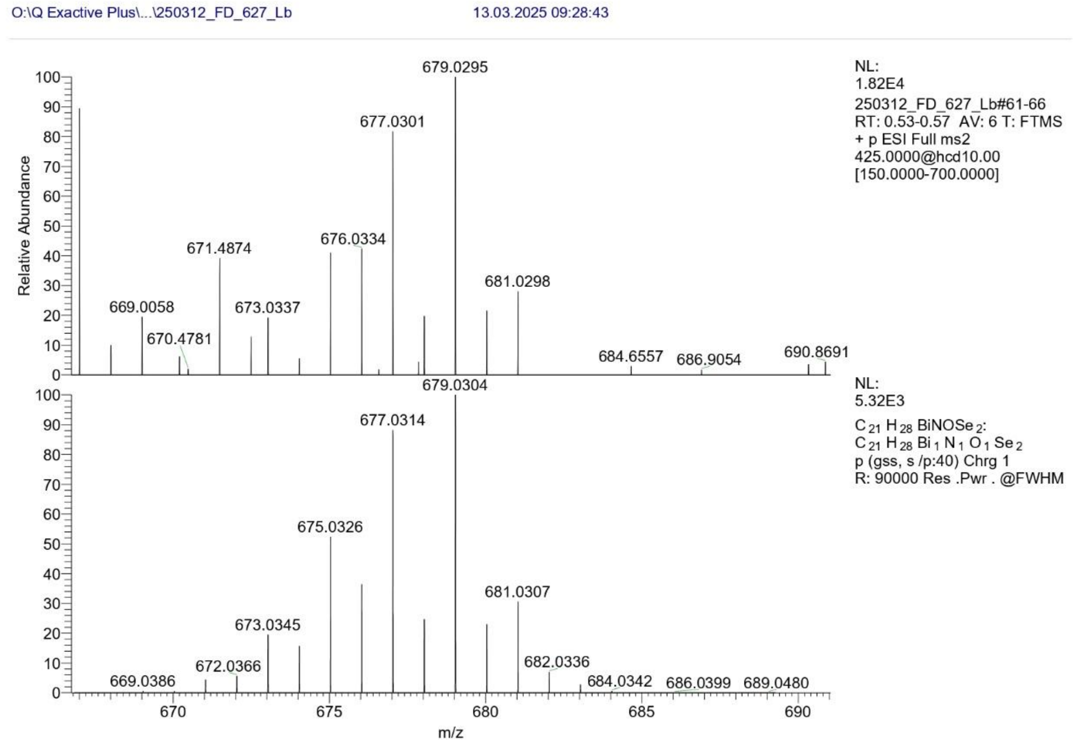

Figure S91. HR-MS spectrum of the reaction of Bi(C≡CPh)$_3$ (**4**) with three equivalents of $(SePh)_2$ in the presence of TEMPO.

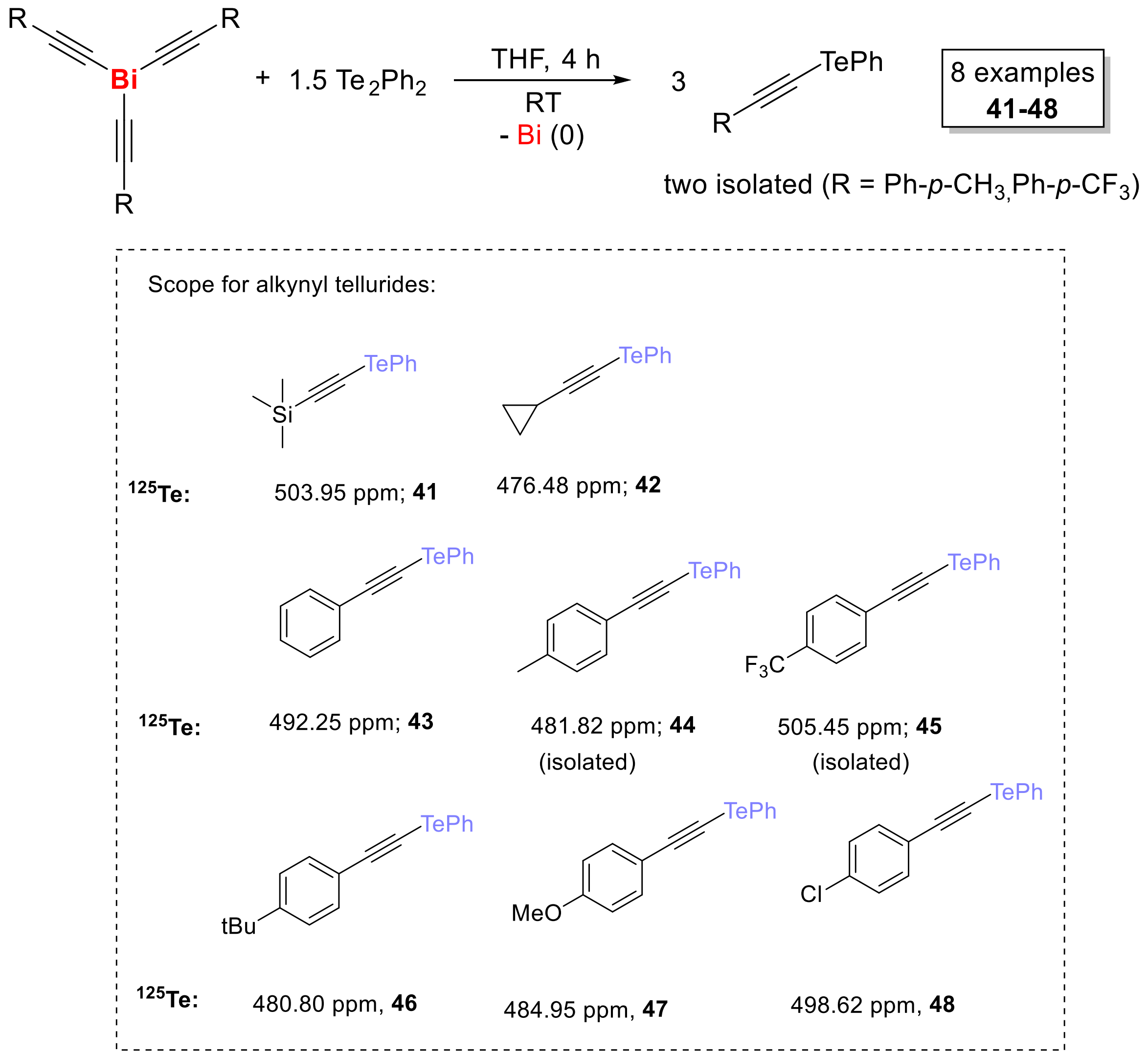

Scheme S7. Synthesis of alkynyl aryl telluro ethers (RC≡C–TePh).

General procedure for the synthesis of alkynyl aryl telluro ethers:

In an argon-filled glovebox, a 20 ml vial was loaded with $Bi(C{\equiv}CR)_3$ (0.0025 mmol, 1.00 equiv.), Te_2Ph_2 (0.375 mmol, 1.50 equiv.) and THF (3 ml). An immediate black precipitate of Bi(0) was observed. The reaction mixture was further stirred for 4 hours at room temperature for complete conversion. After the reaction was completed, the reaction mixture was filtered and dried *in vacuo* to yield the respective telluro ether product RC≡C–TePh. Unlike the analog reactions with Se_2Ph_2, where the formation of $Bi(SePh)_3$ is observed, the tellurium analogue $Bi(TePh)_3$ does not form in this case. The final products were characterized by ^{77}Te NMR and high resolution mass spectrometry.[75,101] Importantly, Compound **42** and **45** are first time synthesize here. The compounds **44** and **45** were also characterized by ^{1}H, ^{13}C and ^{19}F NMR.

Representative NMR spectra and mass spectra of the alkynyl telluro ethers (E= Te) (41-48).

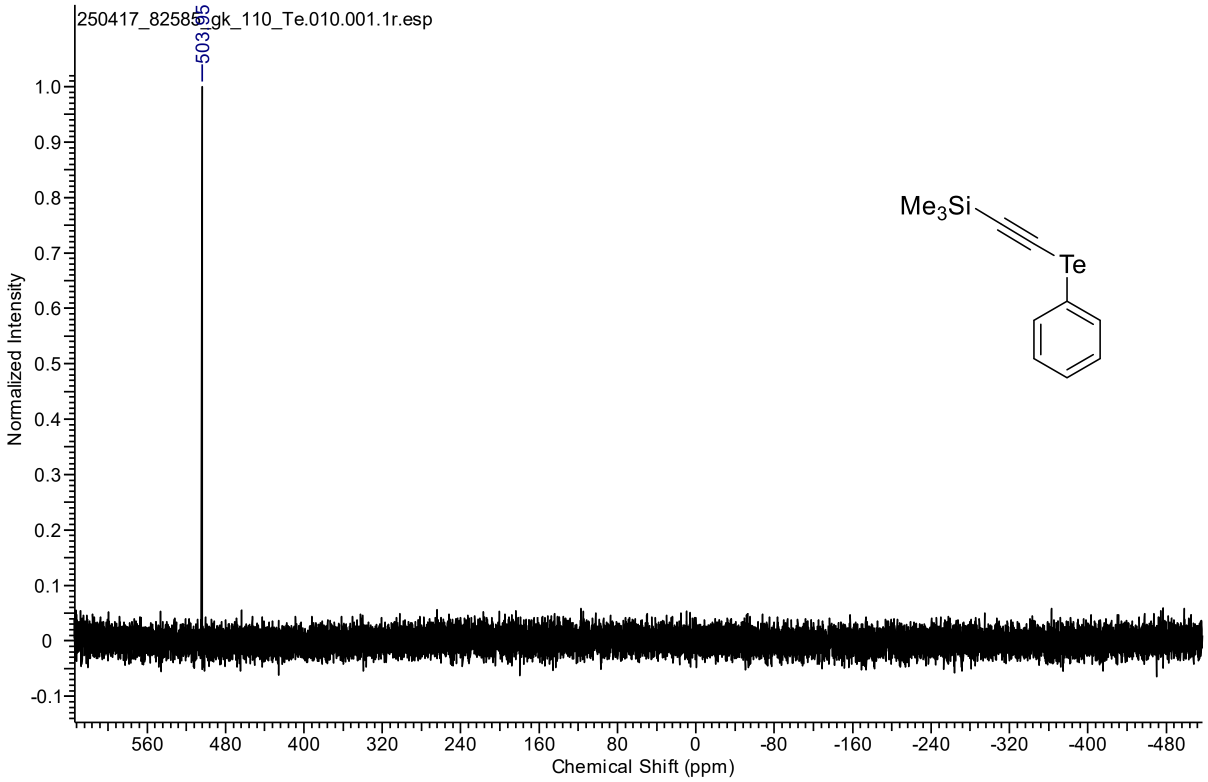

Figure S92. ^{125}Te NMR spectrum of **41** in THF.

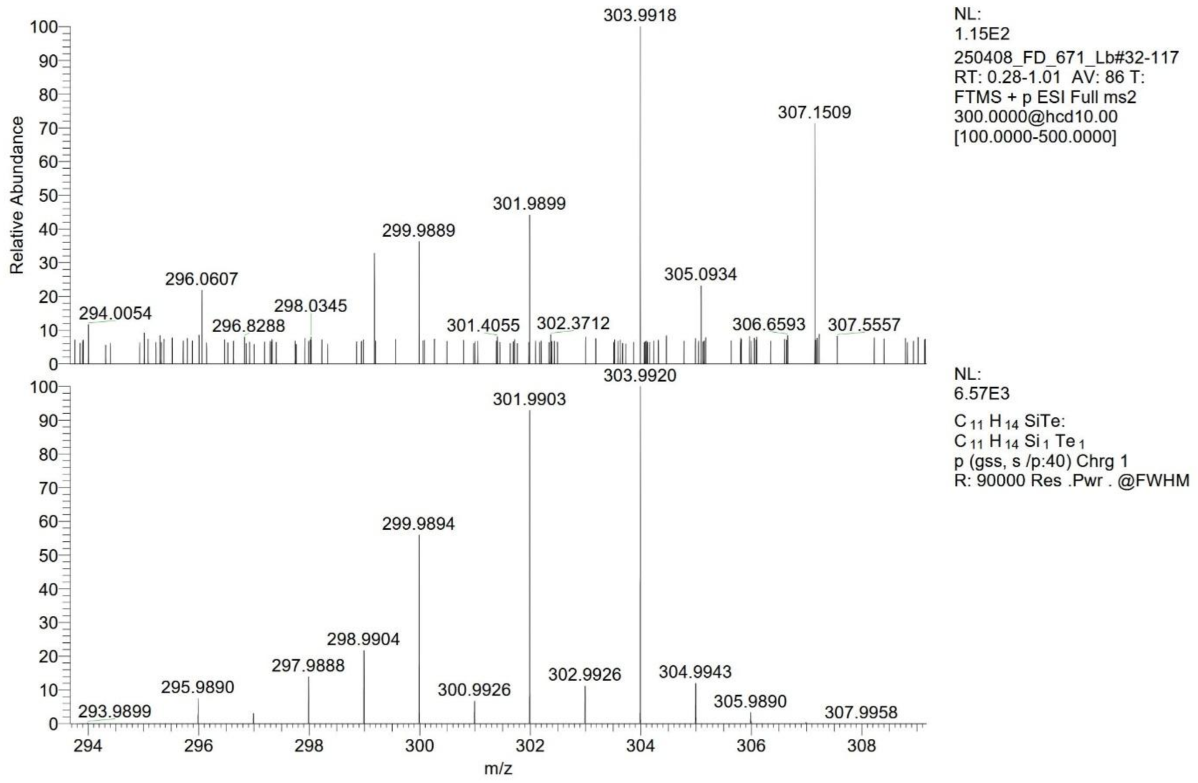

Figure S93. HR-MS spectrum of **41**.

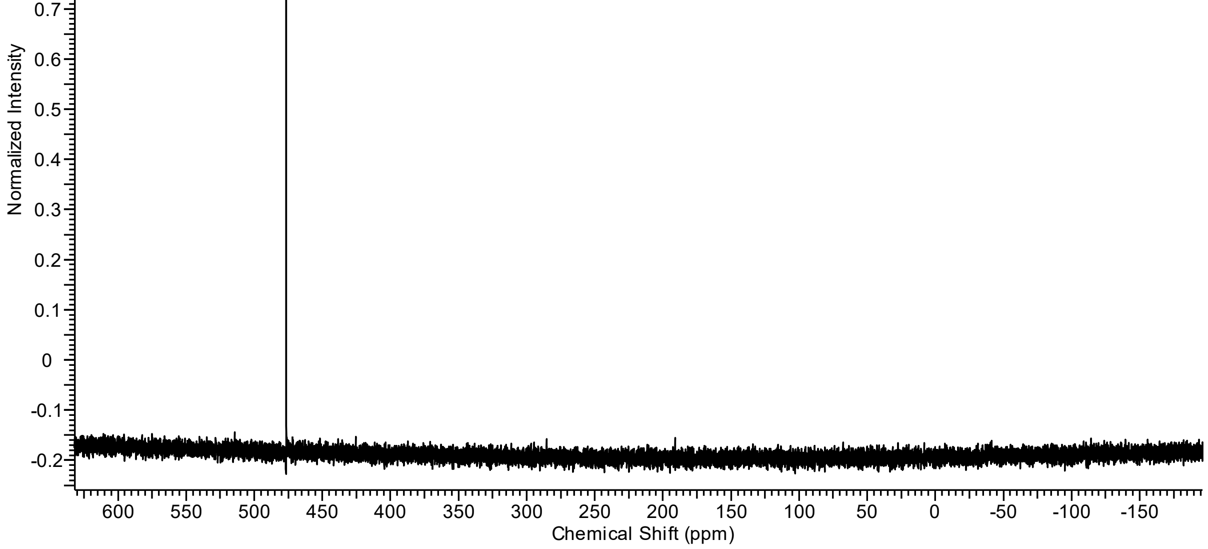

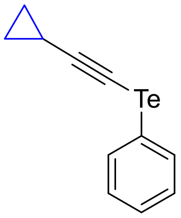

Figure S94. ^{125}Te NMR spectrum of **42** in THF.

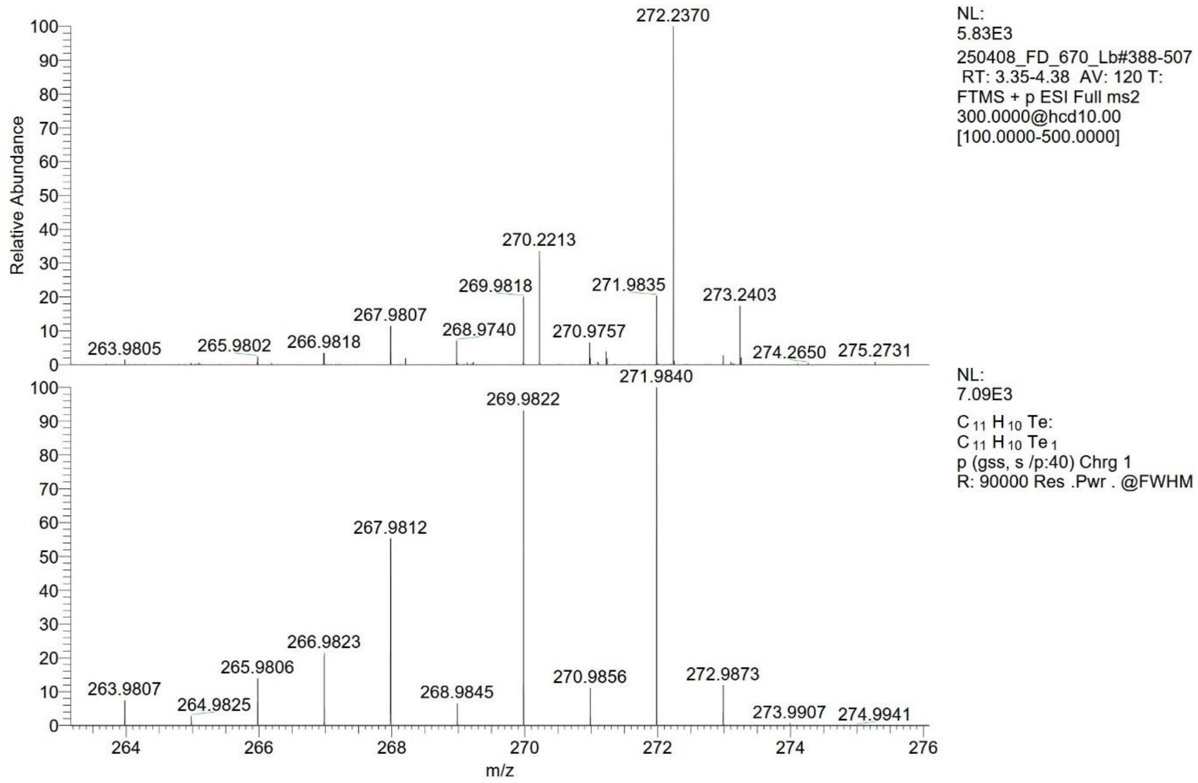

Figure S95. HR-MS spectrum of **42**.

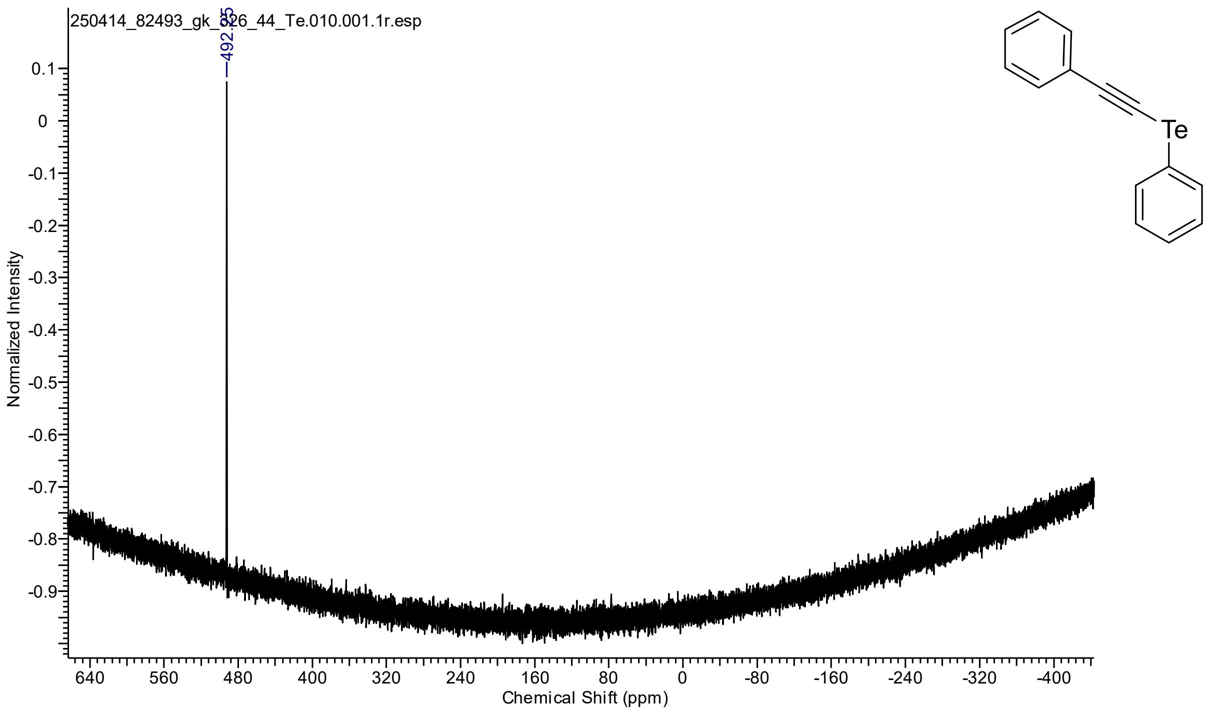

Figure S96. ^{125}Te NMR spectrum of **43** in THF.

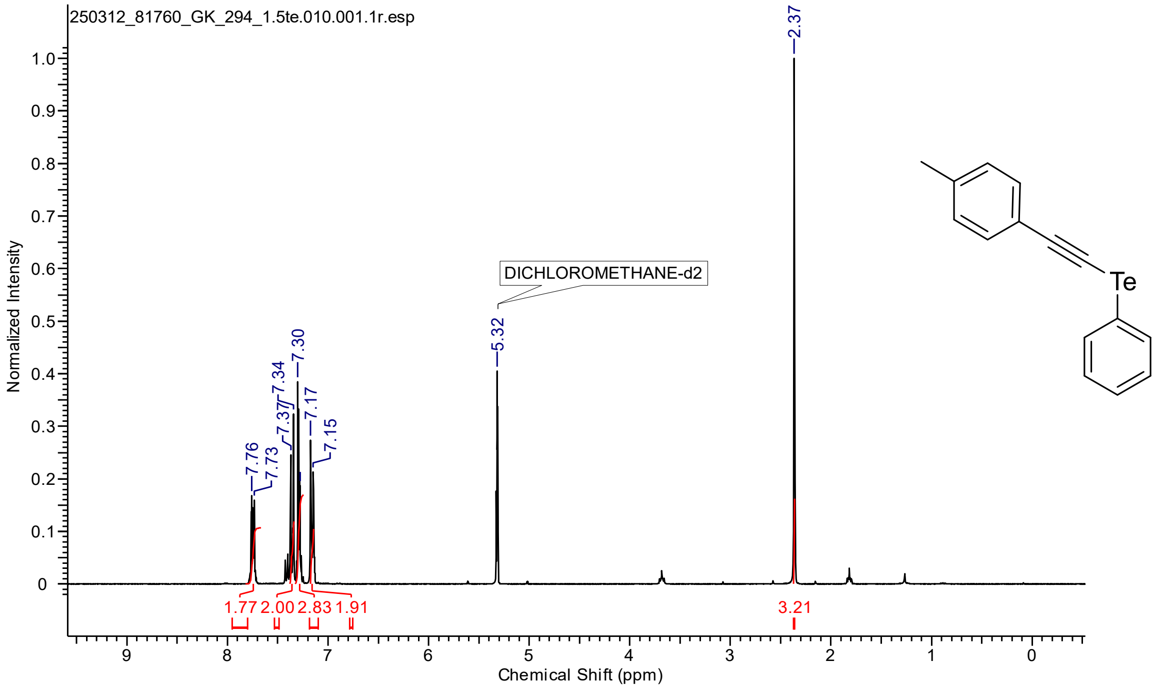

Figure S97. ^{1}H NMR spectrum of **44** in CD_2Cl_2.

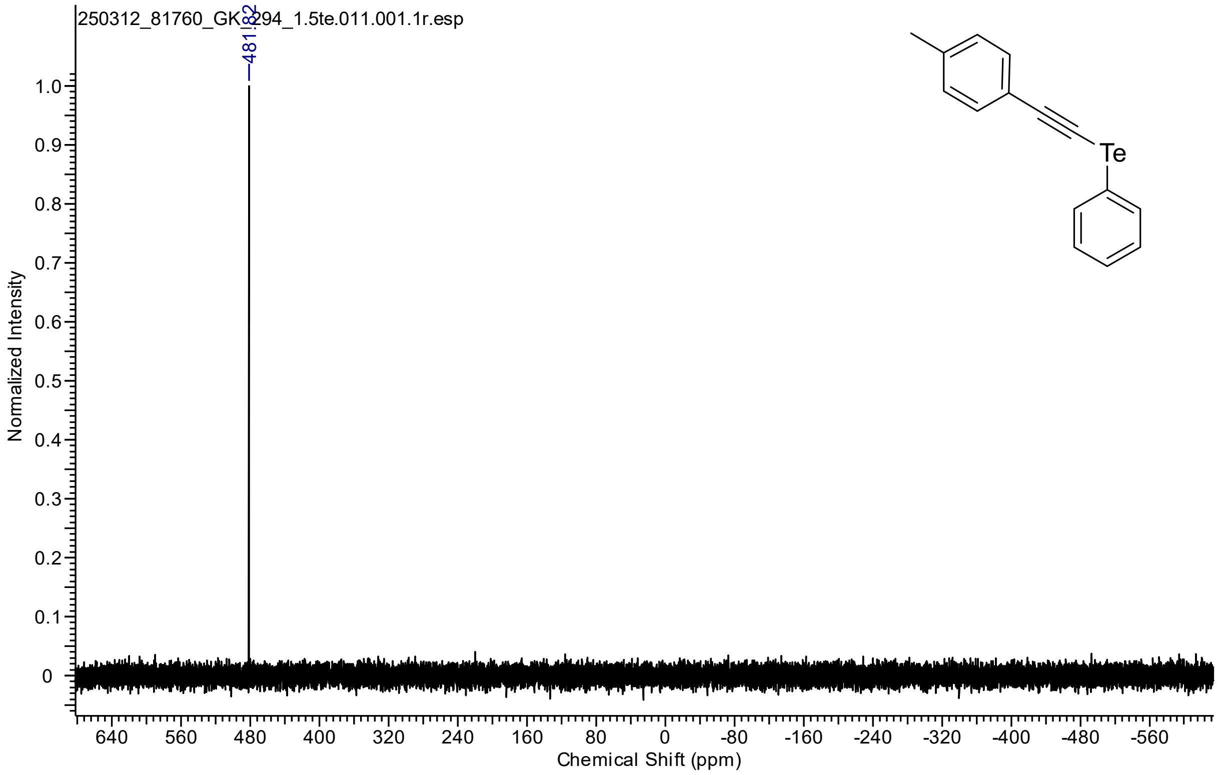

Figure S98. ^{125}Te NMR spectrum of **44** in CD_2Cl_2.

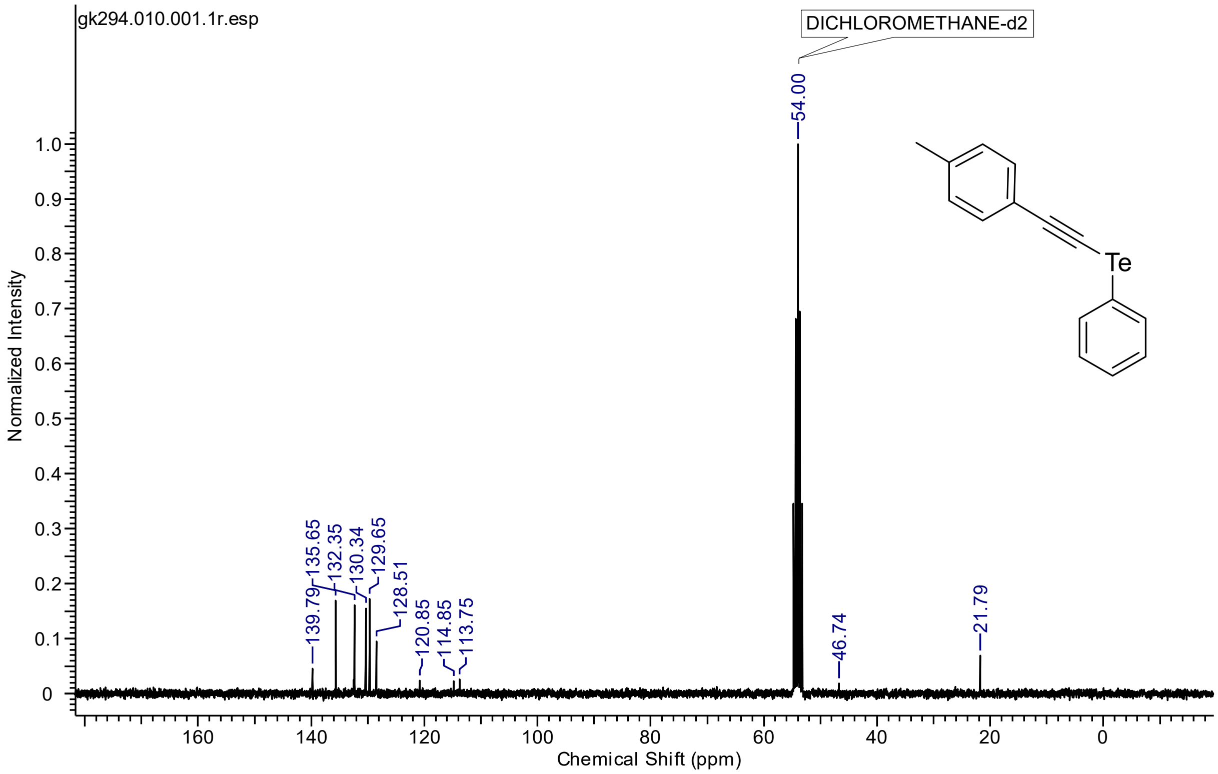

Figure S99. ^{13}C NMR spectrum of **44** in CD_2Cl_2.

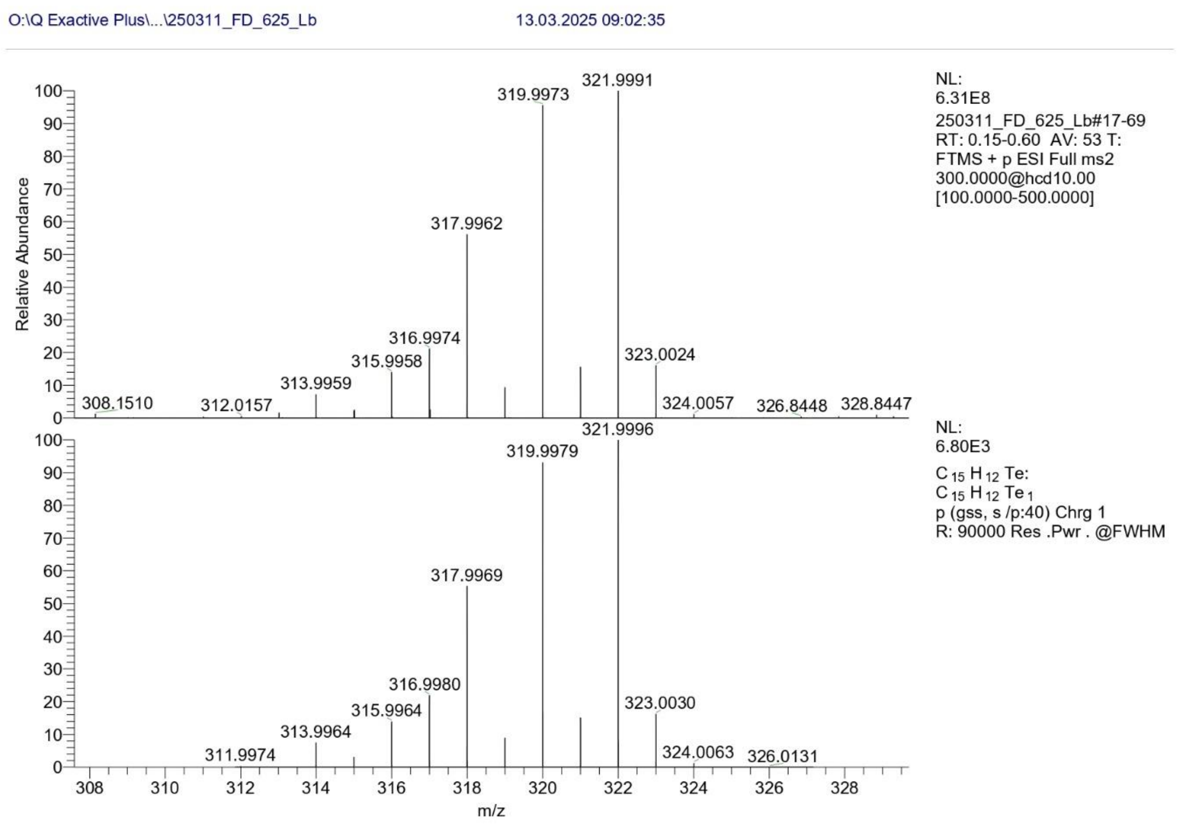

Figure S100. HR-MS spectrum of **44**.

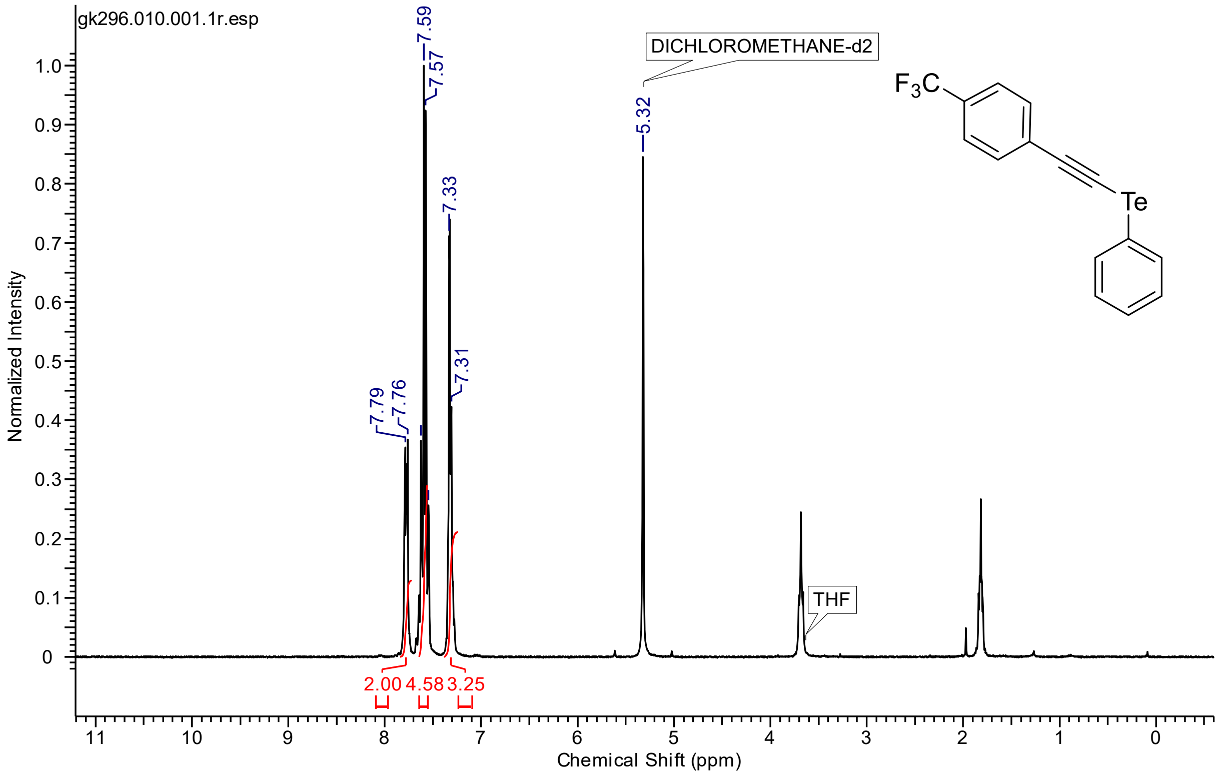

Figure S101. ^{1}H NMR spectrum of **45** in CD_2Cl_2.

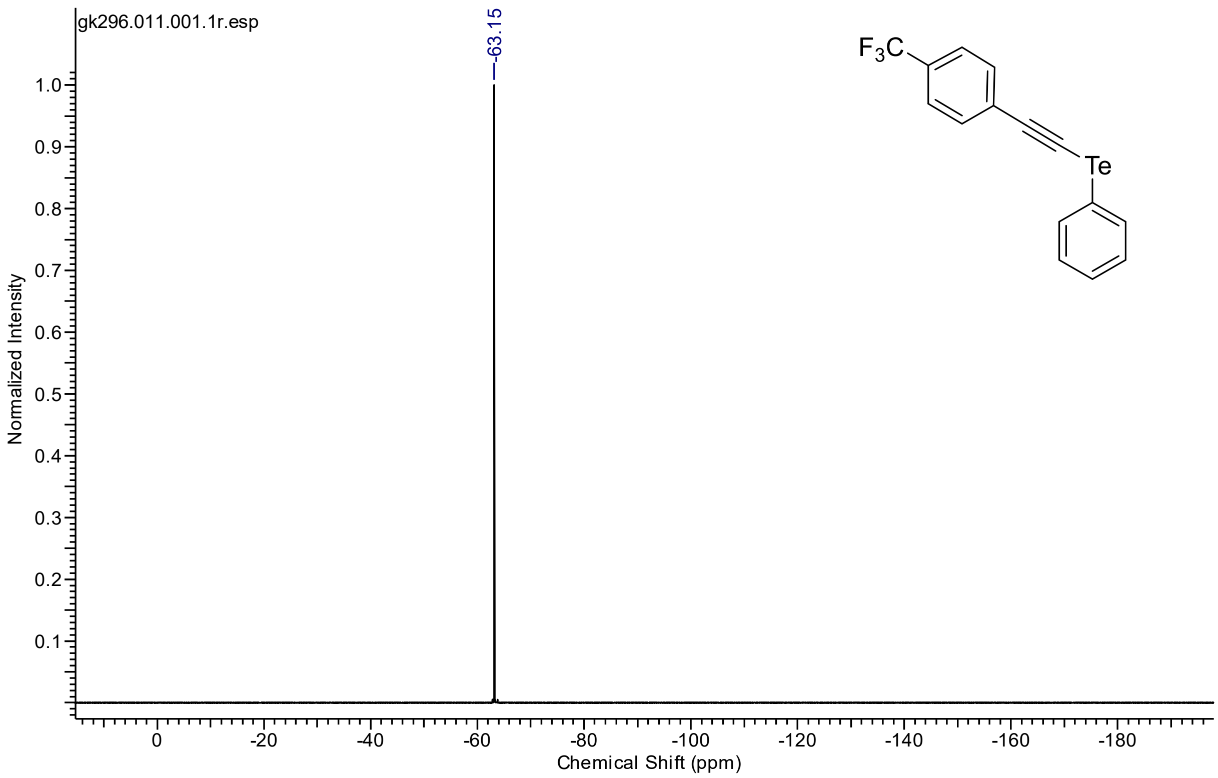

Figure S102. ^{19}F NMR spectrum of **45** in CD_2Cl_2.

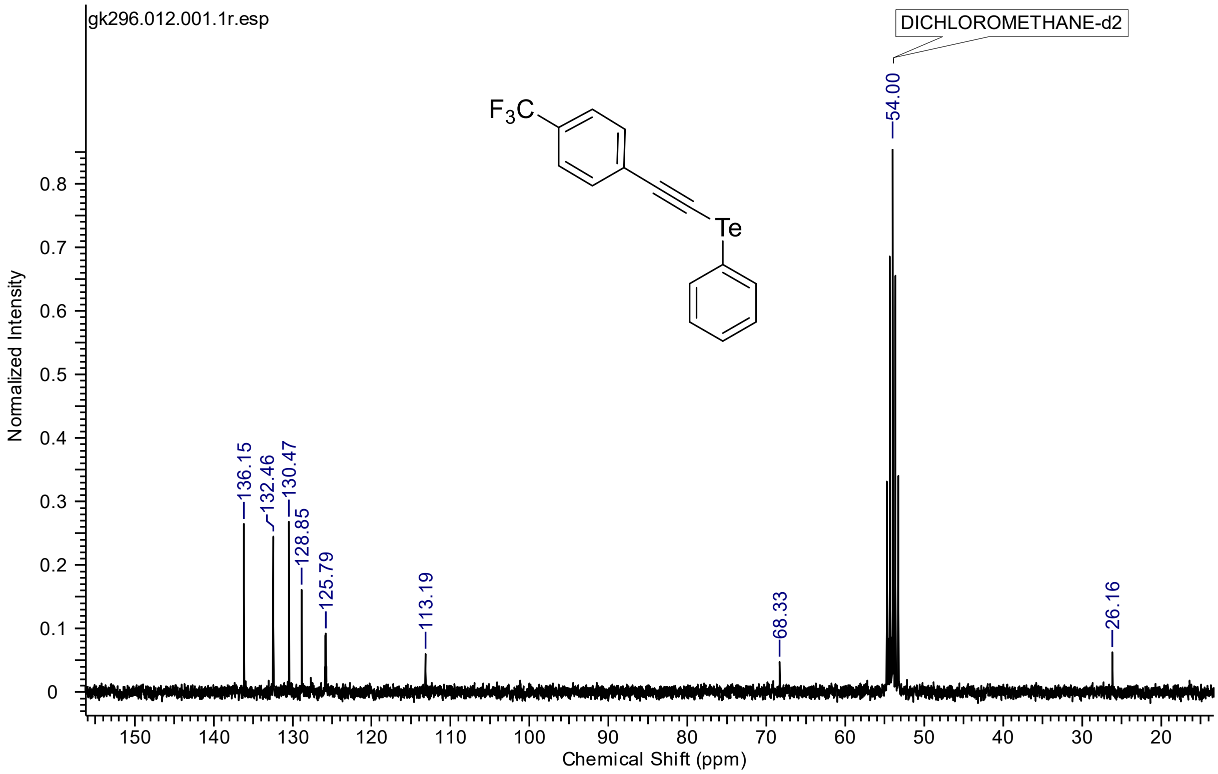

Figure S103. ^{13}C NMR spectrum of **45** in CD_2Cl_2.

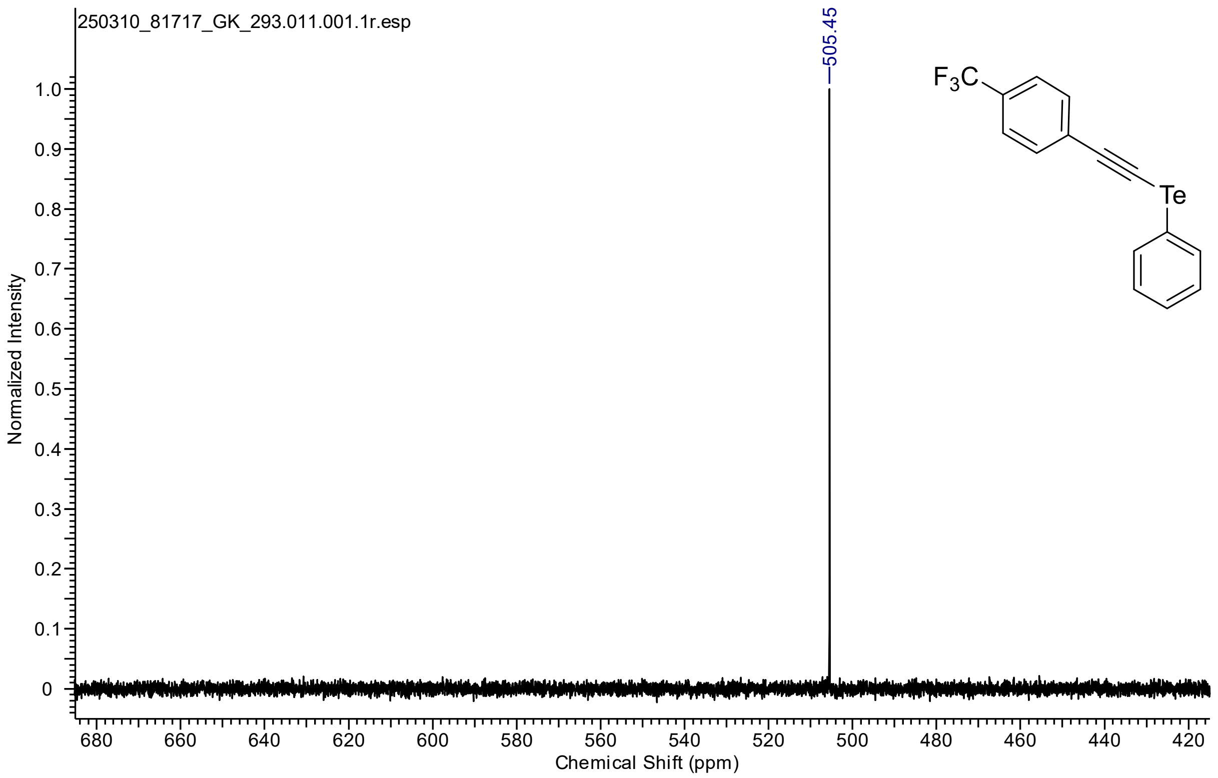

Figure S104. ^{125}Te NMR spectrum of **45** in CD_2Cl_2.

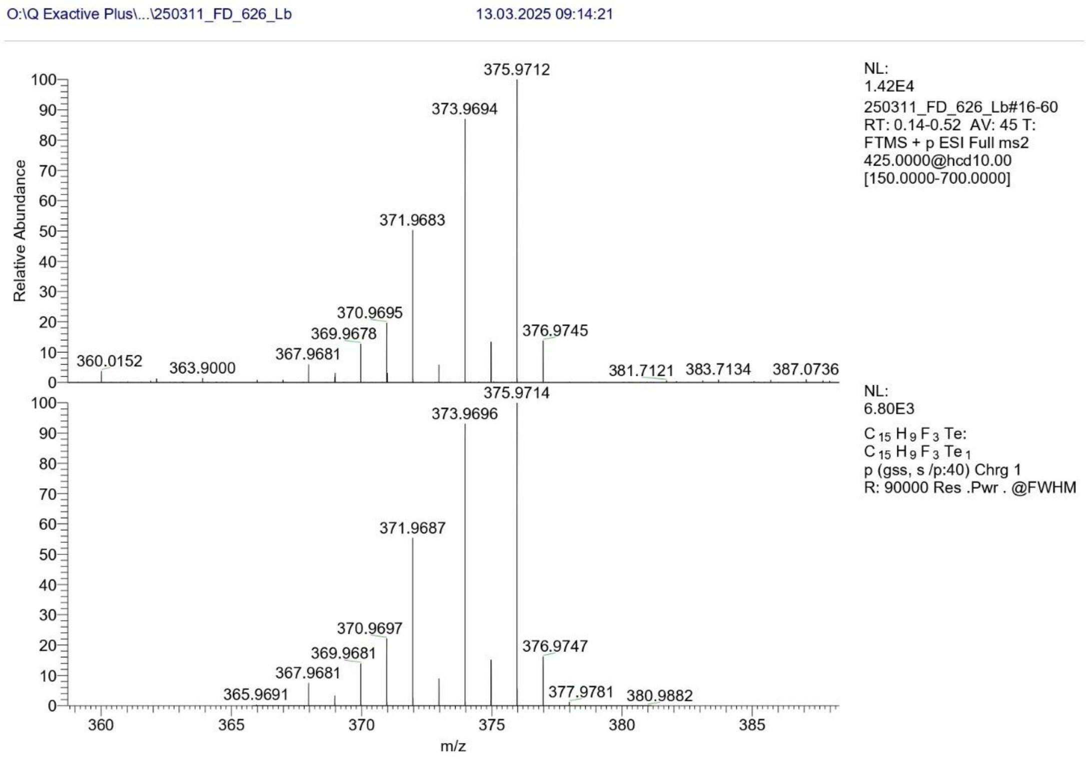

Figure S105. HR-MS spectrum of **45**.

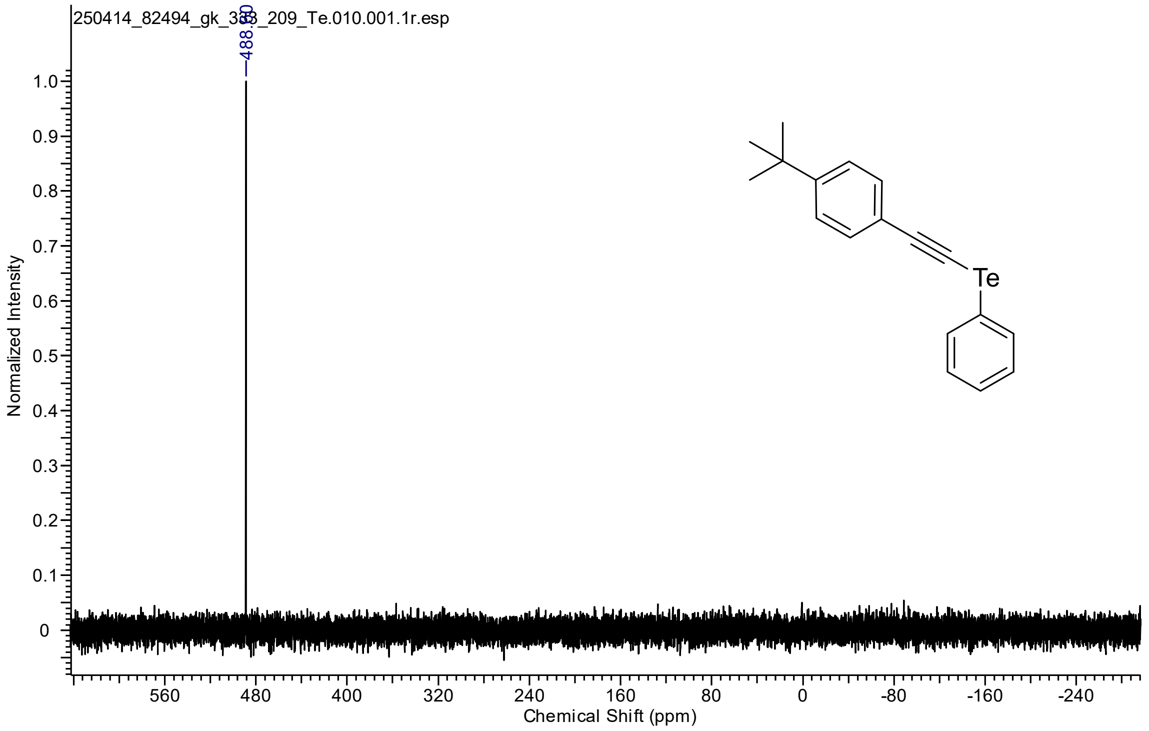

Figure S106. ^{125}Te NMR spectrum of **46** in THF.

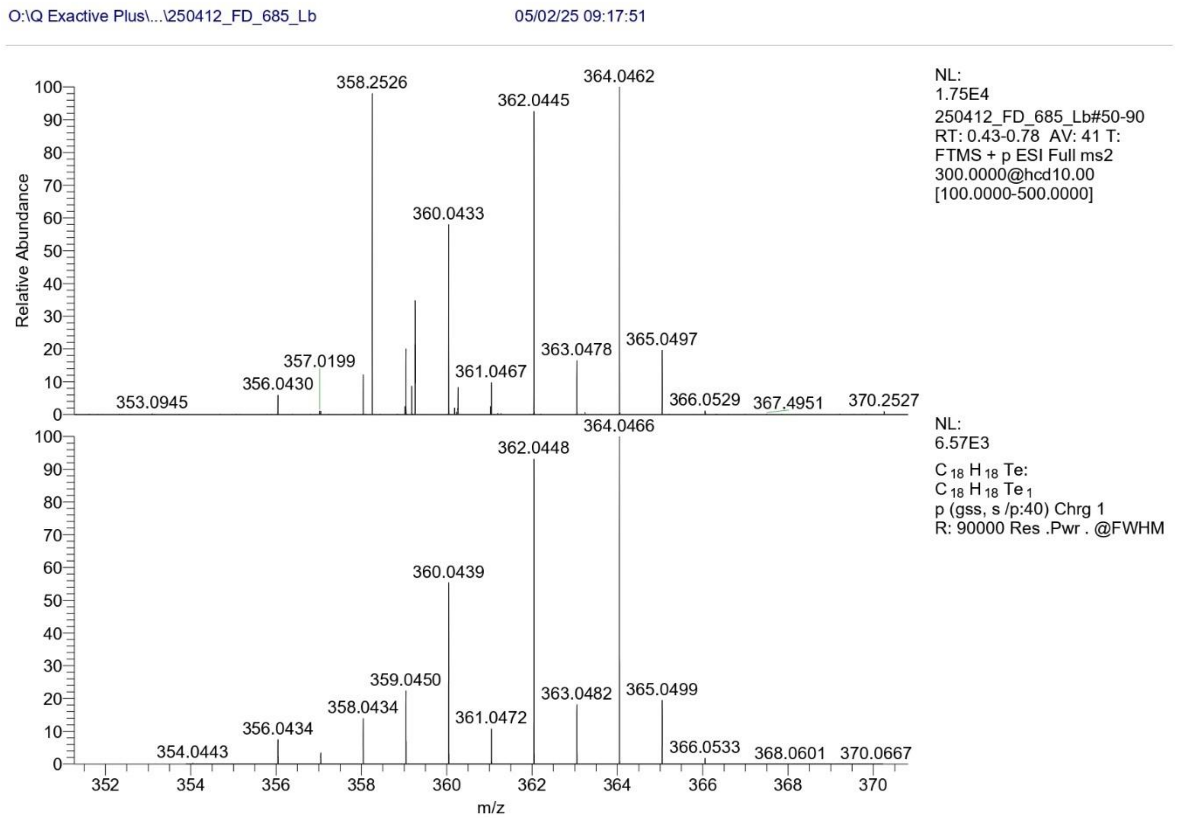

Figure S107. HR-MS spectrum of **46**.

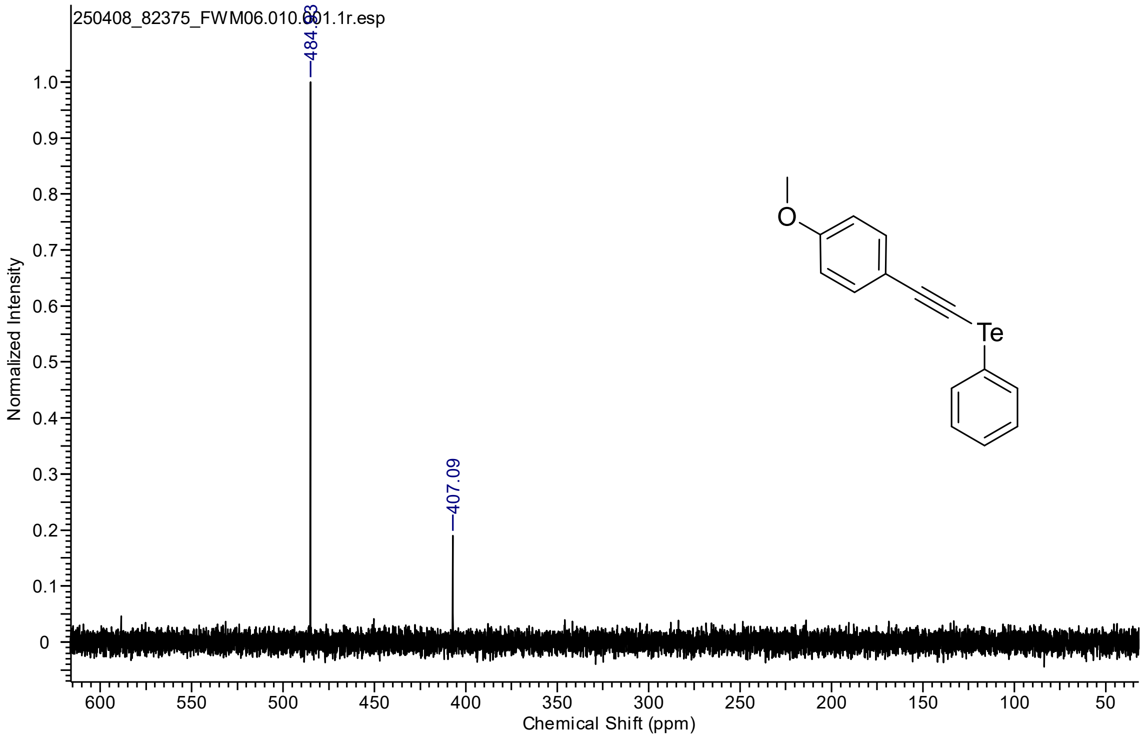

Figure S108. ^{125}Te NMR spectrum of **47** in THF (excess Te_2Ph_2 has been added, which shows a peak at 407.09 ppm in the ^{125}Te NMR).

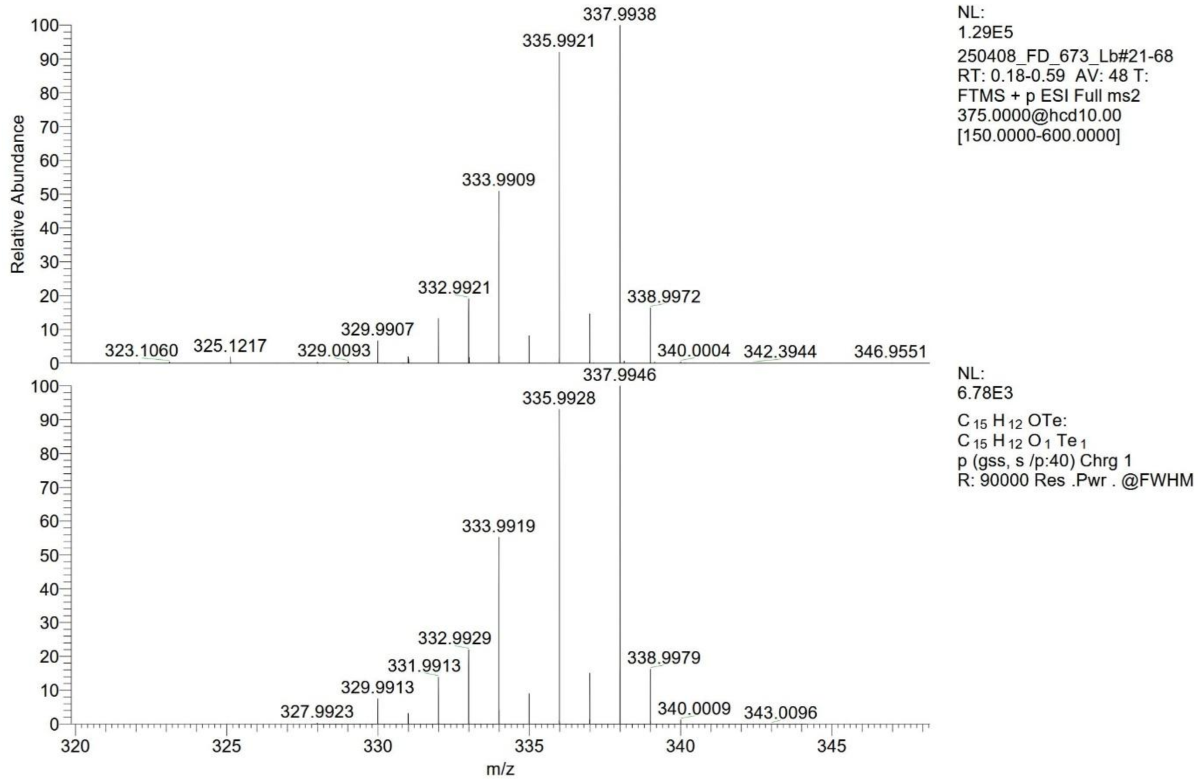

Figure S109. HR-MS spectrum of **47**.

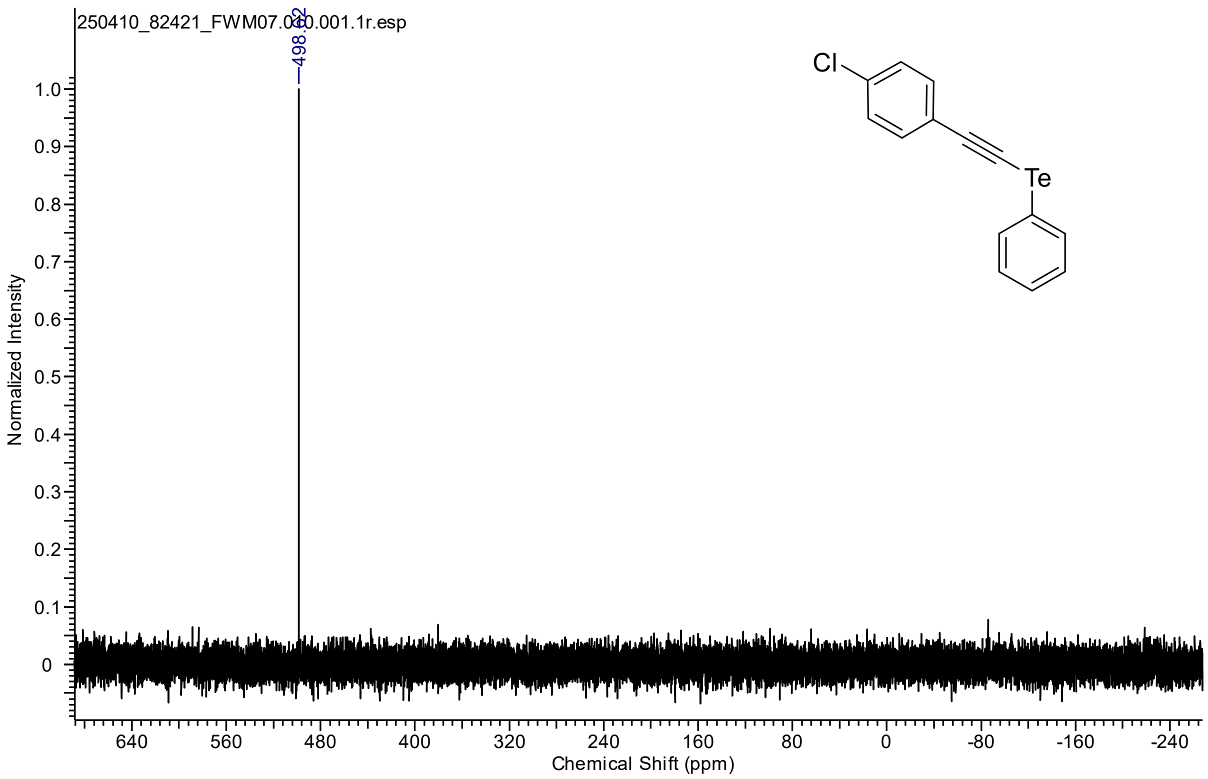

Figure S110. ^{125}Te NMR spectrum of **48** in THF.

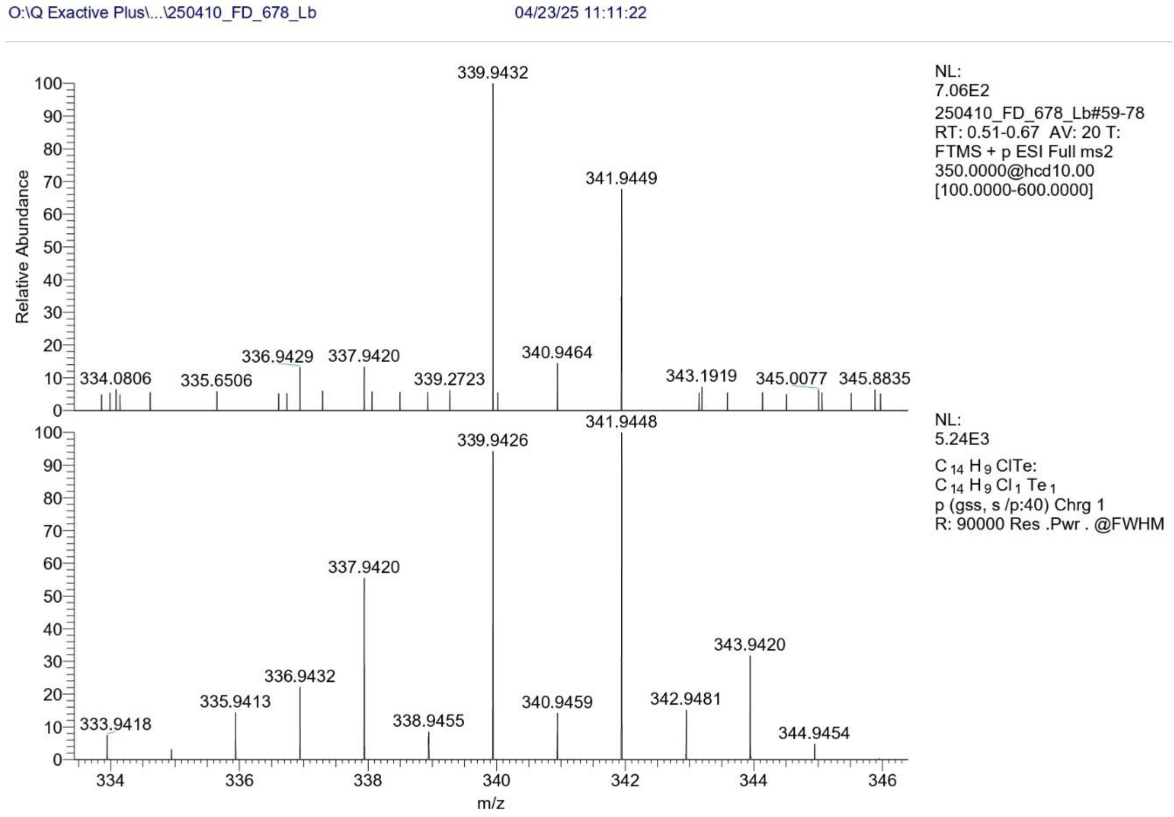

Figure S111. HR-MS spectrum of **48**.

10. Reactions of alkynyl bismuth compounds with HBpin

Synthesis and purification of alkynyl boronic esters

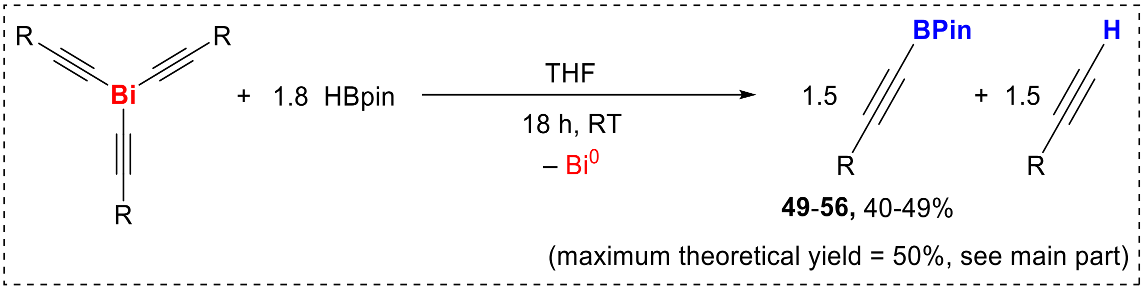

Scheme S8. Synthesis of alkynyl boronic esters (**49-56**) under thermal conditions.

In an argon-filled glovebox, a J. Young NMR tube was loaded with Bi(C≡CR)$_3$ (20 mg, 0.040 mmol), HBpin (9 mg, 0.070 mmol) and THF (0.5 mL). The reaction was run for 18 h at room temperature. After the reaction was completed, all volatiles were removed from the solution under reduced pressure to obtain the alkynyl boronic esters in an inert atmosphere. The resulting solid/liquid was submitted to NMR spectroscopic analyses in a deuterated solvent of choice without further purification being necessary. The spectra are in good agreement with the literature reported data.[102,103] The isolated products were also analyzed mass spectrometry. It should be noted that the ^{11}B NMR spectra of some alkynyl borates exhibit a peak in the 21−22 ppm range, indicating a small amount of B$_2$pin$_3$ (sometimes also noted as B(OR)$_3$ in the literature) as a side product.[102,104] The yields are reported in the main part.

General procedure for the photochemical synthesis of alkynyl boronic esters:

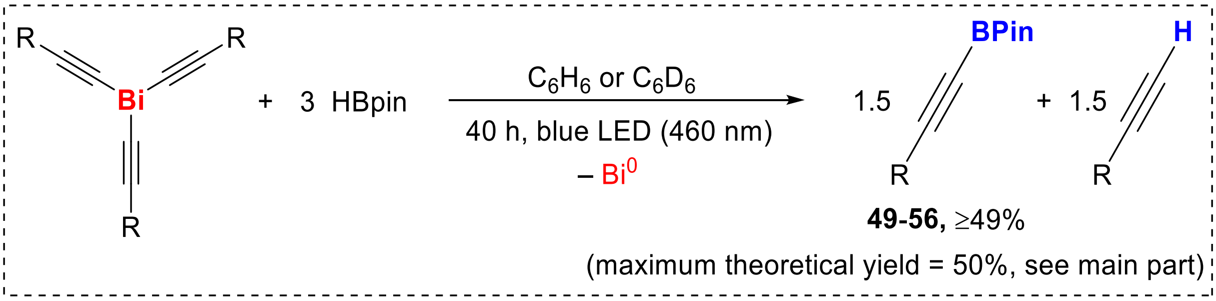

Scheme S9. Synthesis of alkynyl boronic esters (**49-56**) under photochemical conditions.

We also investigated the borylation of compounds **2-9** in benzene (or benzene-d$_6$). At room temperature, the reaction proceeded slowly; however, upon irradiation with a blue LED (λ = 460 nm) for 40 hours in the presence of four equivalents of HBpin, the desired borylated product (**49-56**) was obtained, along with the corresponding free alkyne in equimolar amounts. The reaction was monitored by the ^{1}H and ^{11}B NMR spectroscopy. Under these conditions, full conversion of the bismuth starting material to equimolar amounts of (RC≡C)Bpin (**49-56**) and the corresponding free alkyne (HC≡CR) was observed. I.e. the maximum theoretical yield

of **49-56** and the corresponding free alkynes is 50% each (with respect to bismuth) and spectroscopic yields of ≥49% were observed. Interestingly no C–C coupling products were observed under these conditions.

Notably, when 1.8 equivalents of HBpin were used, the reaction proceeded sluggishly. Even after 40 hours, unreacted bismuth alkyne and HBpin were detected, along with the formation of the decomposition product B_2pin_3 (sometimes also noted as $B(OR)_3$ in the literature).[102,104] This prompted the use of excess HBpin as described above.

Representative analytical data of the alkynyl boronic esters (49-53).[102,103,105]

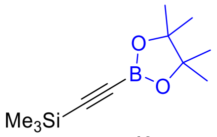

49

trimethyl((4,4,5,5-tetramethyl-1,3,2-dioxaborolan-2-yl)ethynyl)silane

Trimethyl((4,4,5,5-tetramethyl-1,3,2-dioxaborolan-2-yl)ethynyl)silane (49): Colorless amorphous solid, isolated yield 45%

^{1}H NMR, 300 MHz, C_6D_6: δ = 0.08 (s, 9H, $SiMe_3$), 0.96 (s, 12 H, Bpin) ppm.

^{11}B NMR, 128 MHz, C_6D_6: δ = 23.6 (s br) ppm.

^{13}C{^{1}H} NMR, 100.6 MHz, $CDCl_3$: δ = -0.2, 24.9, 84.4, 95.3, 111.1 ppm.

^{29}Si NMR, 128 MHz, C_6D_6: δ = –17.9 ppm.

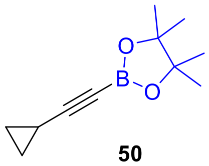

50

2-(cyclopropylethynyl)-4,4,5,5-tetramethyl-1,3,2-dioxaborolane

2-(Cyclopropylethynyl)-4,4,5,5-tetramethyl-1,3,2-dioxaborolane (50): Colorless amorphous solid, isolated yield 47%.

^{1}H NMR, 300 MHz, C_6D_6: δ = 0.23 (m, 2 H, CH_2), 0.52 (m, 2 H, CH_2), 0.92 (m, 1 H, CH) 0.99 (s, 12 H, Bpin) ppm.

^{11}B NMR, 128 MHz, C_6D_6: δ = 24.1 (s br) ppm.

^{13}C{^{1}H} NMR, 100.6 MHz, C_6D_6: δ = 0.9, 9.1, 25.0, 64.4, 83.9, 107.9 (bs) ppm.

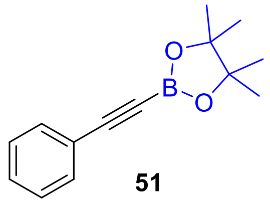

51

4,4,5,5-tetramethyl-2-(phenylethynyl)-1,3,2-dioxaborolane

4,4,5,5-Tetramethyl-2-(phenylethynyl)-1,3,2-dioxaborolane (51): Pale yellow amorphous solid, isolated yield 44%.

^{1}H NMR, 300 MHz, C_6D_6: δ = 1.03 (s, 12 H, Bpin), 6.82-6.91 (m, 3 H), 7.38-7.41 (m, 2 H) ppm.

^{11}B NMR, 128 MHz, C_6D_6: δ = 24.9 (s br) ppm.

^{13}C{^{1}H} NMR, 100.6 MHz, $CDCl_3$: δ = 25.0, 84.4, 102.3 (bs), 114.7, 123.1, 129.6, 133.1 ppm (the resonance of one quaternary carbon atom was not detected due to low signal intensity and/or signal broadening).[105]

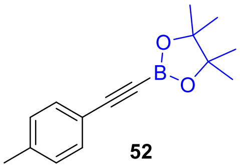

52

4,4,5,5-tetramethyl-2-(*p*-tolylethynyl)-1,3,2-dioxaborolane

4,4,5,5-Tetramethyl-2-(p-tolylethynyl)-1,3,2-dioxaborolane (52): Pale yellow amorphous solid, isolated yield 48%.

^{1}H NMR, 300 MHz, C_6D_6: δ = 1.04 (s, 12 H, Bpin), 1.88 (s, 3 H, C*H*$_3$), 6.65 (d, *J* = 7.9Hz, 2 H, *m*-Ar-H), 7.33 (d, *J* = 7.9 Hz, 2 H, *o*-Ar-H) ppm.

^{11}B NMR, 128 MHz, C_6D_6: δ = 24.7 (s br) ppm.

^{13}C{^{1}H} NMR, 100.6 MHz, $CDCl_3$: δ = 21.6, 25.1, 84.4, 102.63, 120.2, 129.7, 133.1, 139.8 ppm (the resonance of one quaternary carbon atom was not detected due to low signal intensity and/or signal broadening).[105]

HR-MS (LIFDI, pos.): Calc. for $(^{12}C_{15}{}^{1}H_{19}{}^{10}B^{16}O_2)^+$ ([**52**]$^+$): m/z = 242.1473, found m/z = 242.1472.

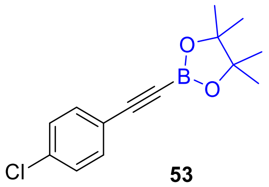

53

2-((4-chlorophenyl)ethynyl)-4,4,5,5-tetramethyl-1,3,2-dioxaborolane

2-((4-Chlorophenyl)ethynyl)-4,4,5,5-tetramethyl-1,3,2-dioxaborolane (53): Pale yellow amorphous solid, isolated yield 47%.

^{1}H NMR, 300 MHz, C_6D_6: δ = 1.03 (s, 12 H, Bpin), 6.73 (m, 2 H, *m*-Ar-H), 7.02 (m, 2 H, *o*-Ar-H) ppm.

^{11}B NMR, 128 MHz, C_6D_6: δ = 24.8 (s br) ppm.

^{13}C{^{1}H} NMR, 100.6 MHz, $CDCl_3$: δ = 25.0, 84.6, 100.9 (bs), 121.3, 129.2, 134.2, 135.9 ppm (the resonance of one quaternary carbon atom was not detected due to low signal intensity and/or signal broadening).[105]

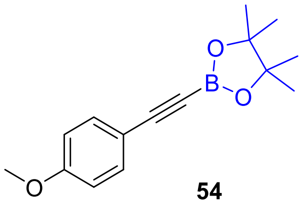

54

2-((4-methoxyphenyl)ethynyl)-4,4,5,5-tetramethyl-1,3,2-dioxaborolane

2-((4-Methoxyphenyl)ethynyl)-4,4,5,5-tetramethyl-1,3,2-dioxaborolane (54): colorless oil, isolated yield 48%.

^{1}H NMR, 300 MHz, C_6D_6: δ = 1.05 (s, 12 H, Bpin), 3.10 (s, 3 H, OCH_3), 6.44 (d, J = 8.2 Hz, 2 H, *m*-Ar-H), 7.35 (d, J= 9.4 Hz, 2 H, *o*-Ar-H) ppm.

^{11}B NMR, 128 MHz, C_6D_6: δ = 24.8 (s br) ppm.

^{13}C{^{1}H} NMR, 100.6 MHz, C_6D_6: δ = 25.1, 55.0, 76.8, 84.3, 102.7 (bs), 114.7, 134.2, 134.8, 161.1 ppm.

HR-MS (LIFDI, pos.): Calc. for $(^{12}C_{15}{}^{1}H_{19}{}^{10}B^{16}O_3)^+$ ([**54**]$^+$): m/z = 258.1422, found m/z = 258.1422.

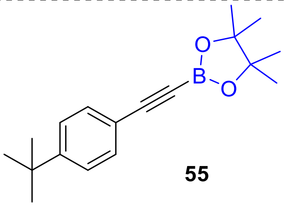

55

2-((4-(*tert*-butyl)phenyl)ethynyl)-4,4,5,5-tetramethyl-1,3,2-dioxaborolane

2-((4-(*tert*-Butyl)phenyl)ethynyl)-4,4,5,5-tetramethyl-1,3,2-dioxaborolane (55): Pale yellow amorphous solid, isolated yield 40%.

^{1}H NMR, 300 MHz, C_6D_6: δ = 1.04 (s, 12 H, Bpin), 1.08 (s, 9 H, *t*-Bu), 6.97 (m, 2 H), 7.43 (m, 2 H) ppm.

^{11}B NMR, 128 MHz, C_6D_6: δ = 24.2 (s br) ppm.

^{13}C{^{1}H} NMR, 100.6 MHz, C_6D_6: δ = 25.1, 31.4, 35.0, 84.3, 102.4 (bs) 114.7, 120.3, 125.0, 133.0, 152.8 ppm.

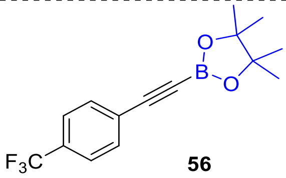

56

4,4,5,5-tetramethyl-2-((4-(trifluoromethyl)phenyl)ethynyl)-1,3,2-dioxaborolane

4,4,5,5-Tetramethyl-2-((4-(trifluoromethyl)phenyl)ethynyl)-1,3,2-dioxaborolane (56): Pale yellow amorphous solid, isolated yield 49%.

^{1}H NMR, 300 MHz, C_6D_6: δ = 1.03 (s, 12 H, Bpin), 6.97 (d, *J* = 8.0 Hz, 2 H, *m*-Ar-H), 7.13 (d, *J*= 8.0 Hz, 2 H; *o*-Ar-H) ppm.

^{11}B NMR, 128 MHz, C_6D_6: δ = 24.7 (s br) ppm.

^{19}F NMR, 128 MHz, C_6D_6: δ = 62.8 (s) ppm

^{13}C{^{1}H} NMR, 100.6 MHz, C_6D_6: δ = 25.0, 80.4, 84.7, 100.3, 124.7 (q, $^{1}J_{C\text{-}F}$ = 272.8 Hz, CF_3), 125.7 (q, $^{3}J_{C\text{-}F}$ = 3.8 Hz, *m*-Ar-*C*), 126.4, 131.1 (q, $^{2}J_{C\text{-}F}$ = 32.4 Hz, *p*-Ar-*C*), 133.2 (*o*-Ar-*C*) ppm.

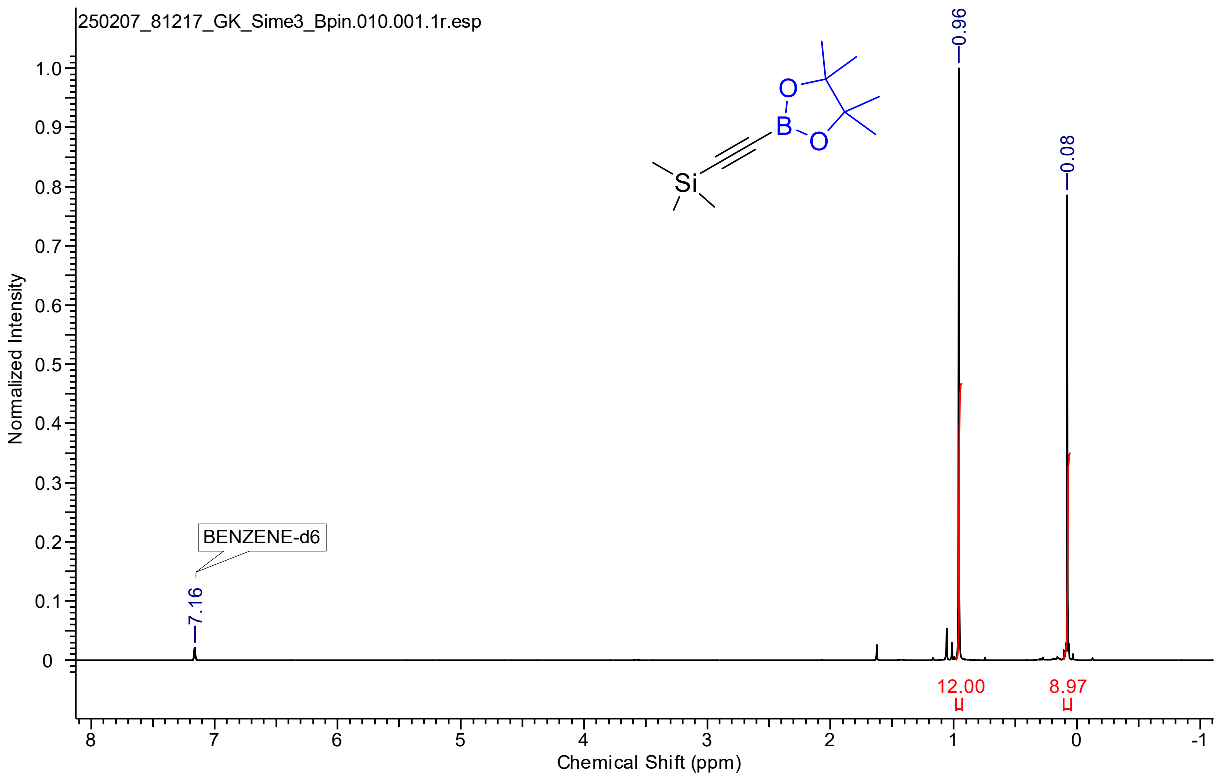

Figure S112. ^{1}H NMR spectrum of **49** in C_6D_6.

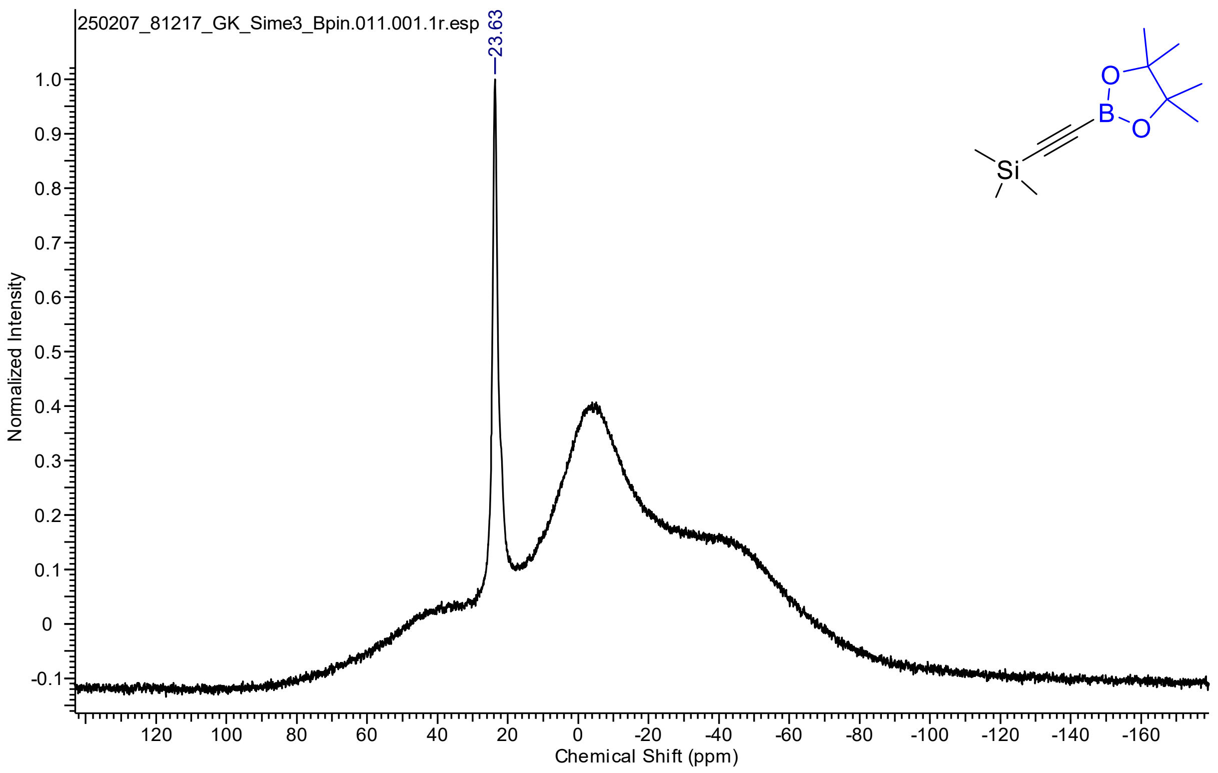

Figure S113. ^{11}B NMR spectrum of **49** in C_6D_6.

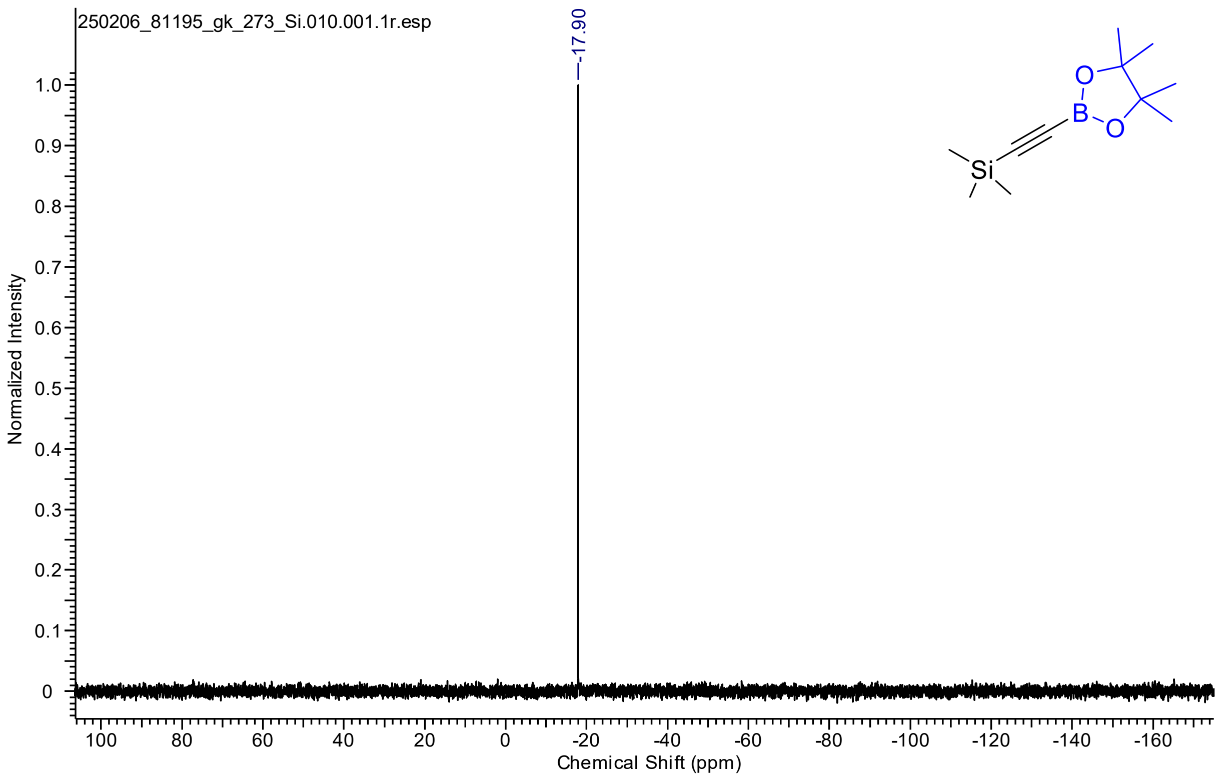

Figure S114. ^{29}Si NMR spectrum of **49** in C_6D_6.

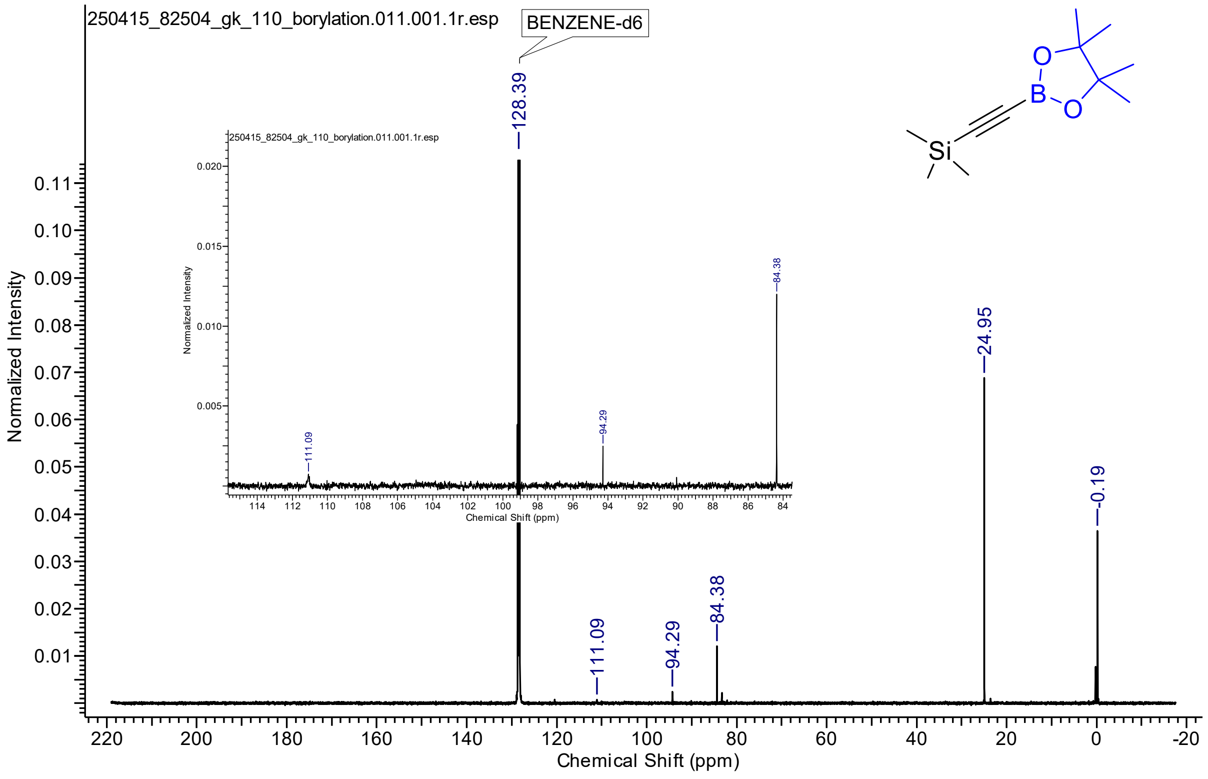

Figure S115. ^{13}C NMR Spectrum of **49** in C_6D_6.

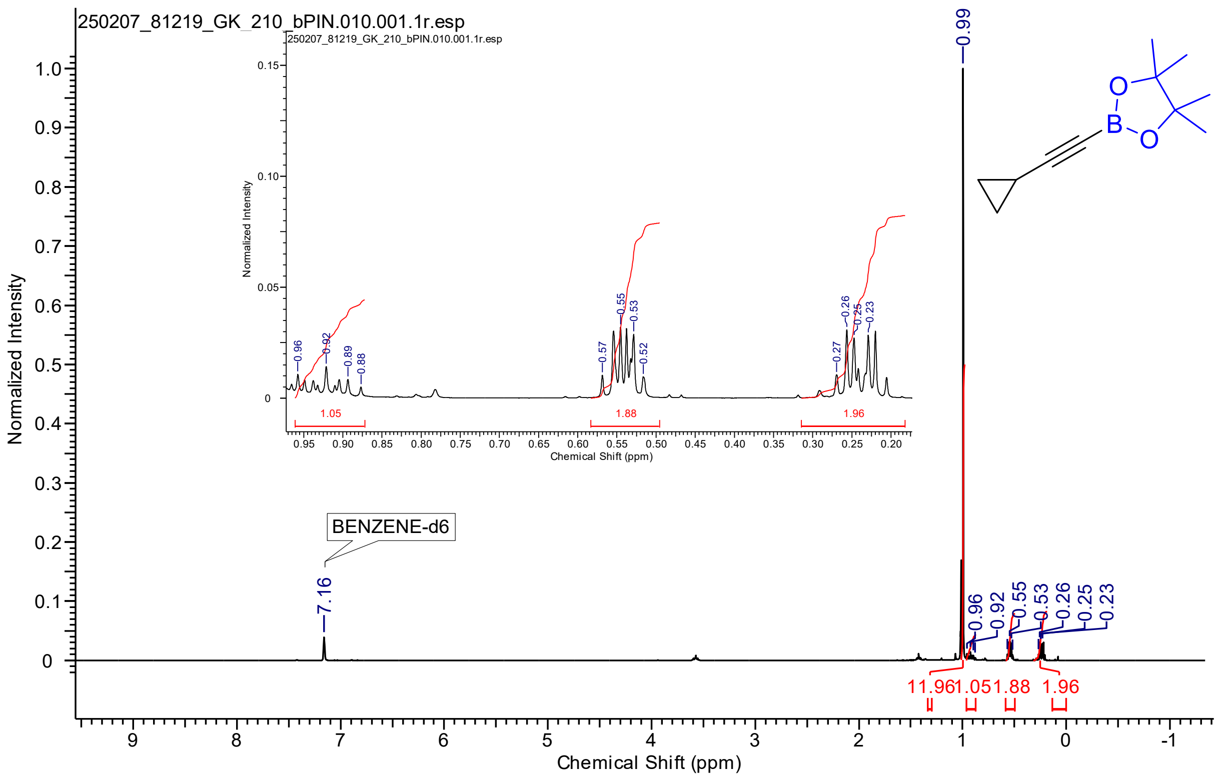

Figure S116. ^{1}H NMR spectrum of **50** in C_6D_6.

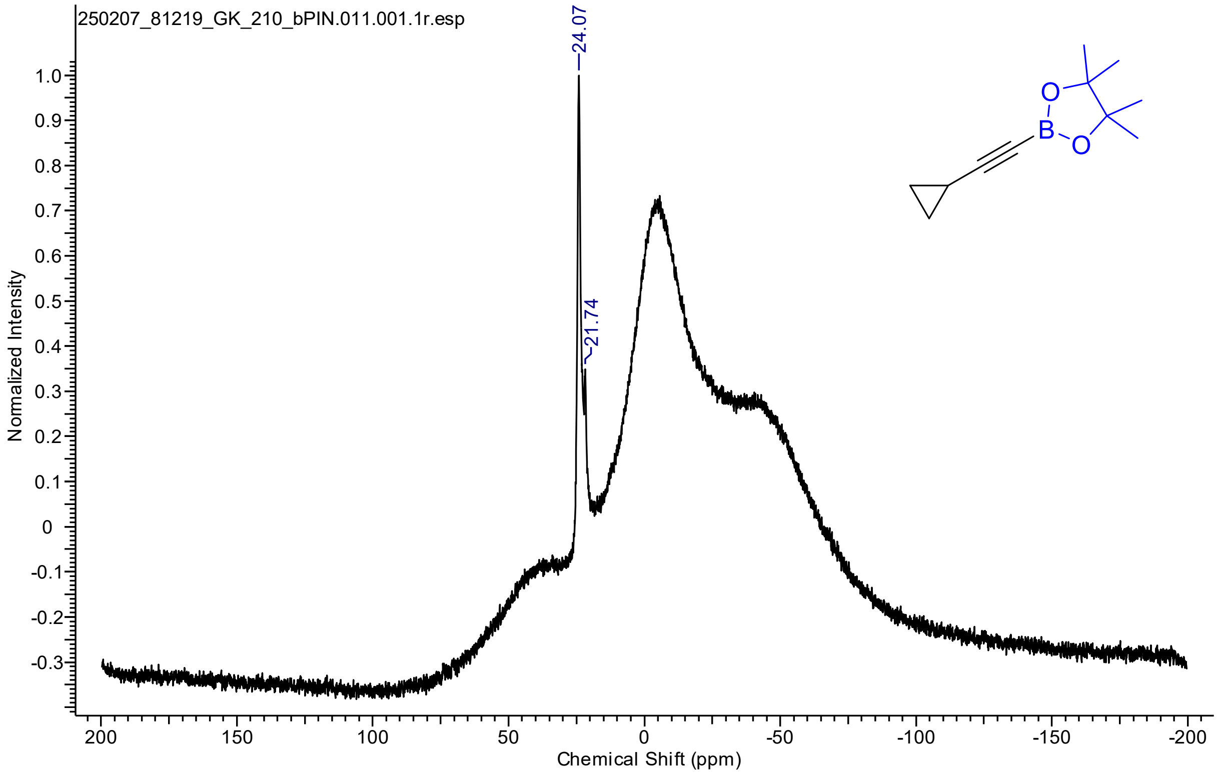

Figure S117. ^{11}B NMR spectrum of **50** in C_6D_6.

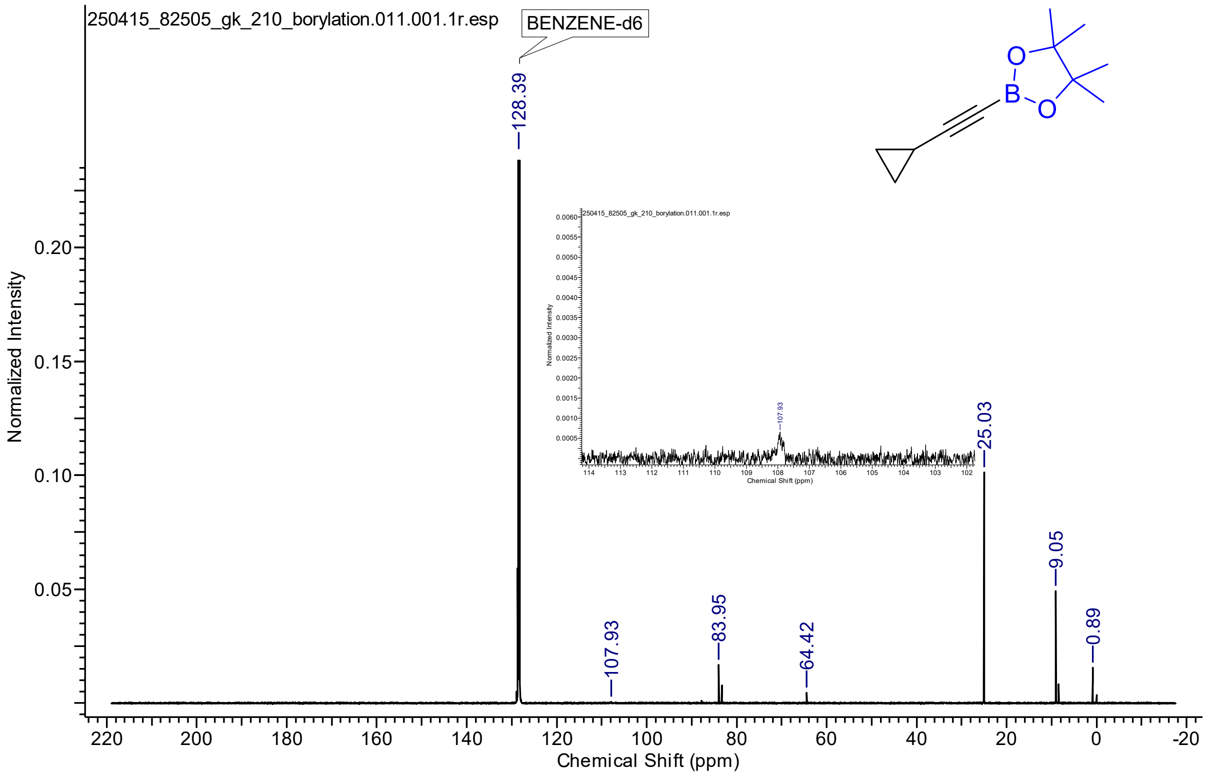

Figure S118. ^{13}C NMR spectrum of **50** in C_6D_6.

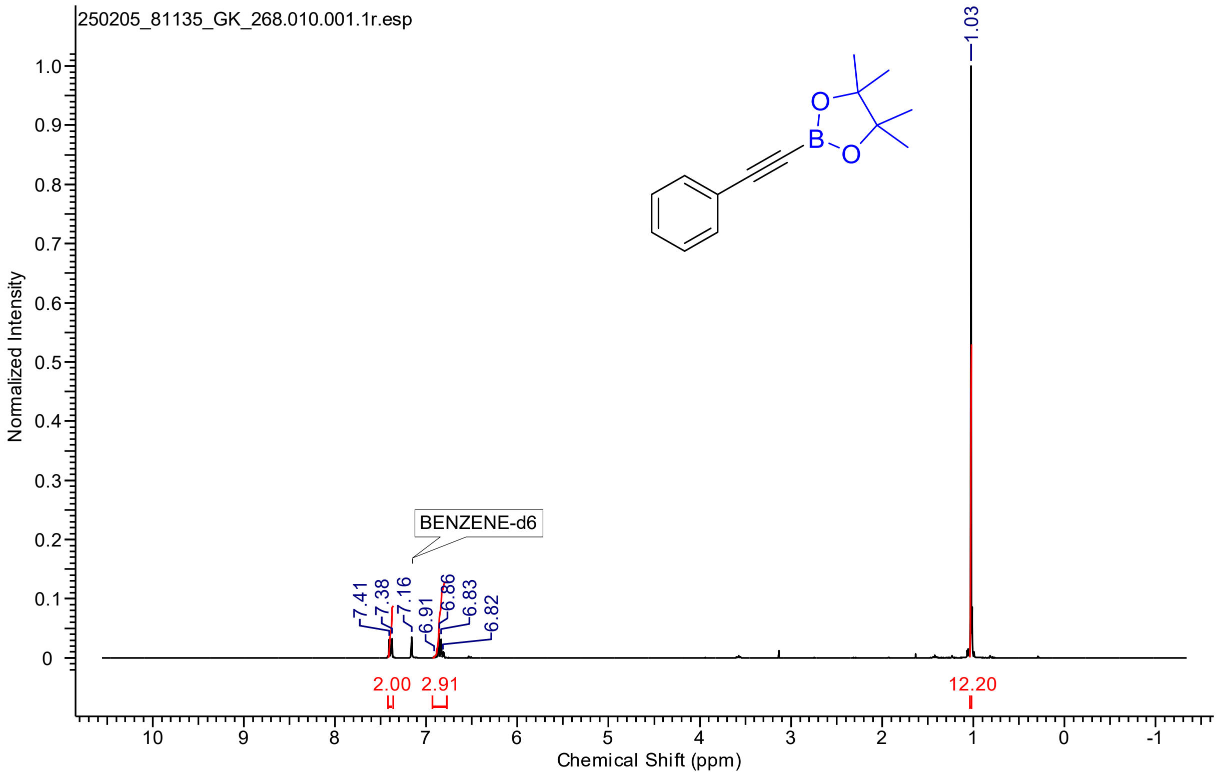

Figure S119. ^{1}H NMR spectrum of **51** in C_6D_6.

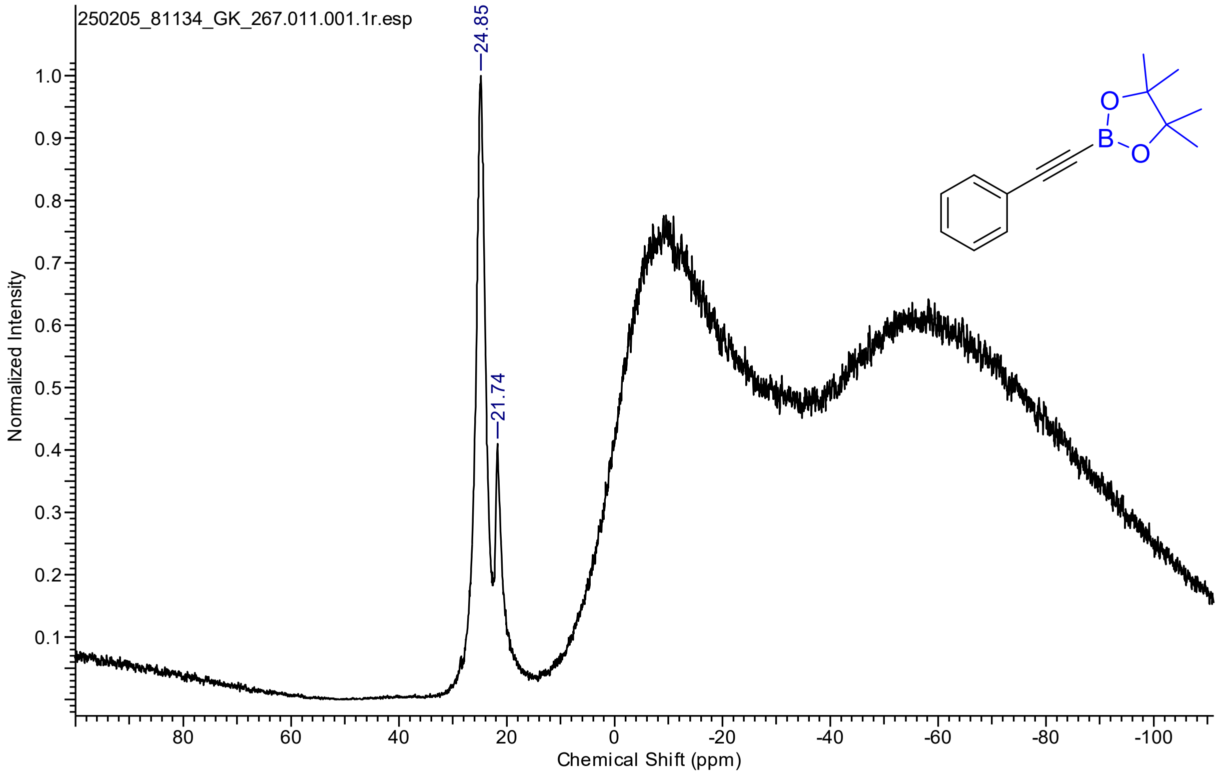

Figure S120. ^{11}B NMR spectrum of **51** in C_6D_6.

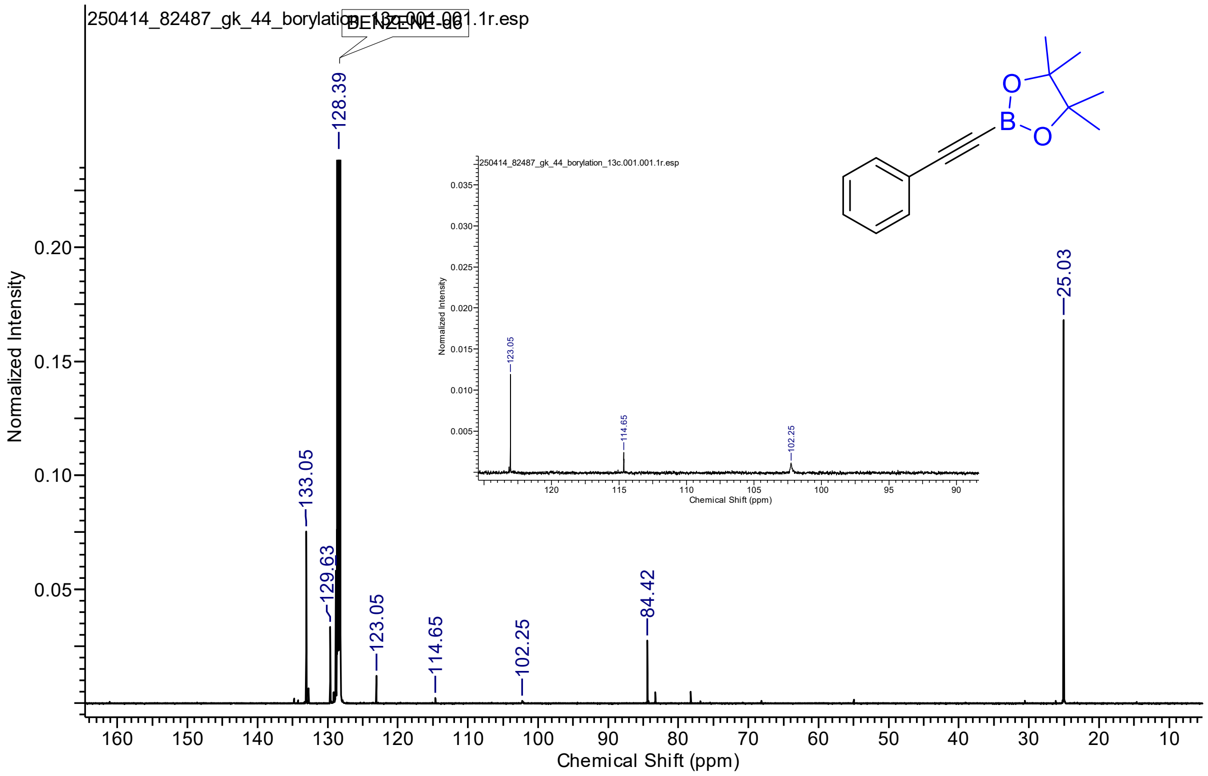

Figure S121. ^{13}C NMR spectrum of **51** in C_6D_6.

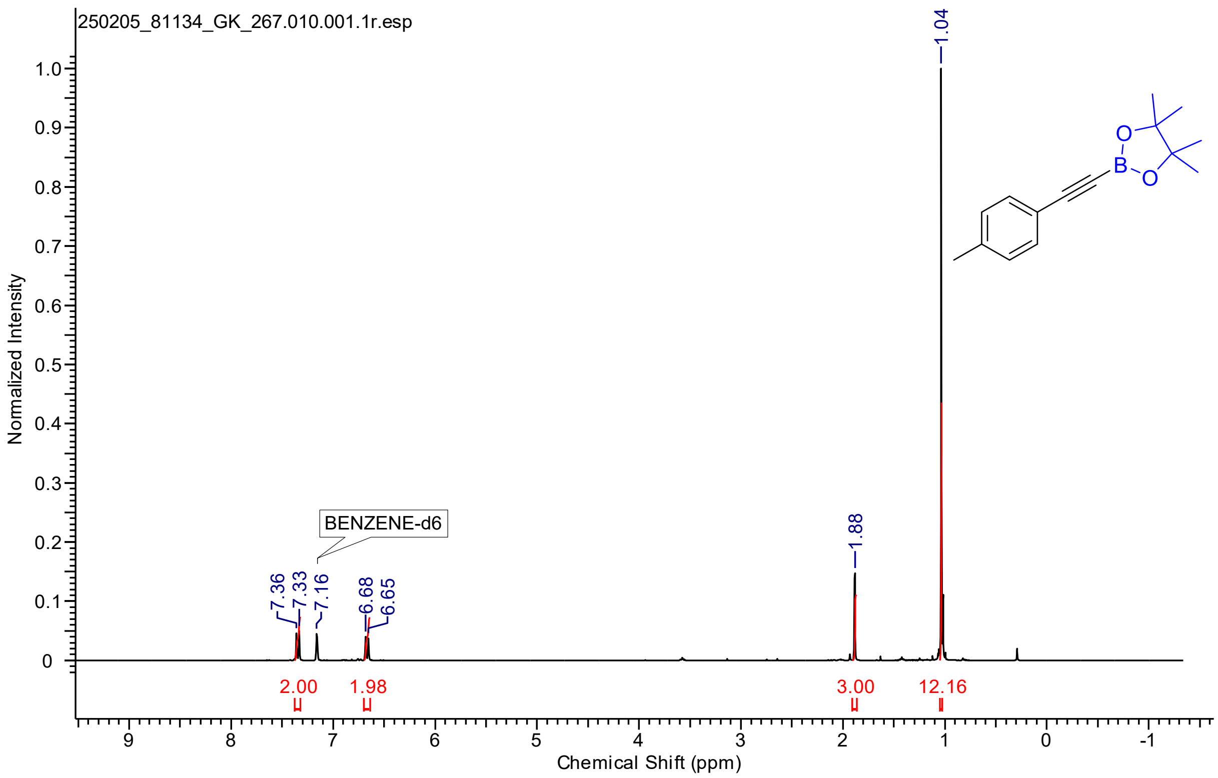

Figure S122. ^{1}H NMR spectrum of **52** in C_6D_6.

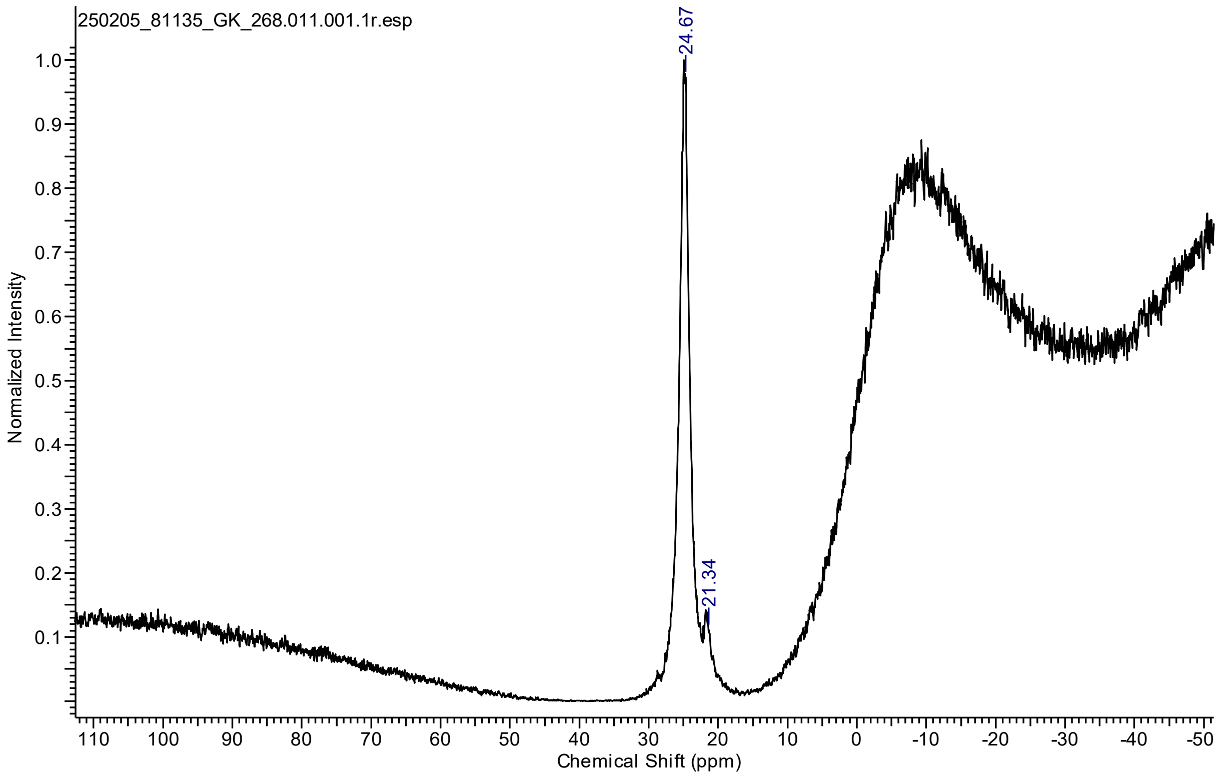

Figure S123. ^{11}B NMR spectrum of **52** in C_6D_6.

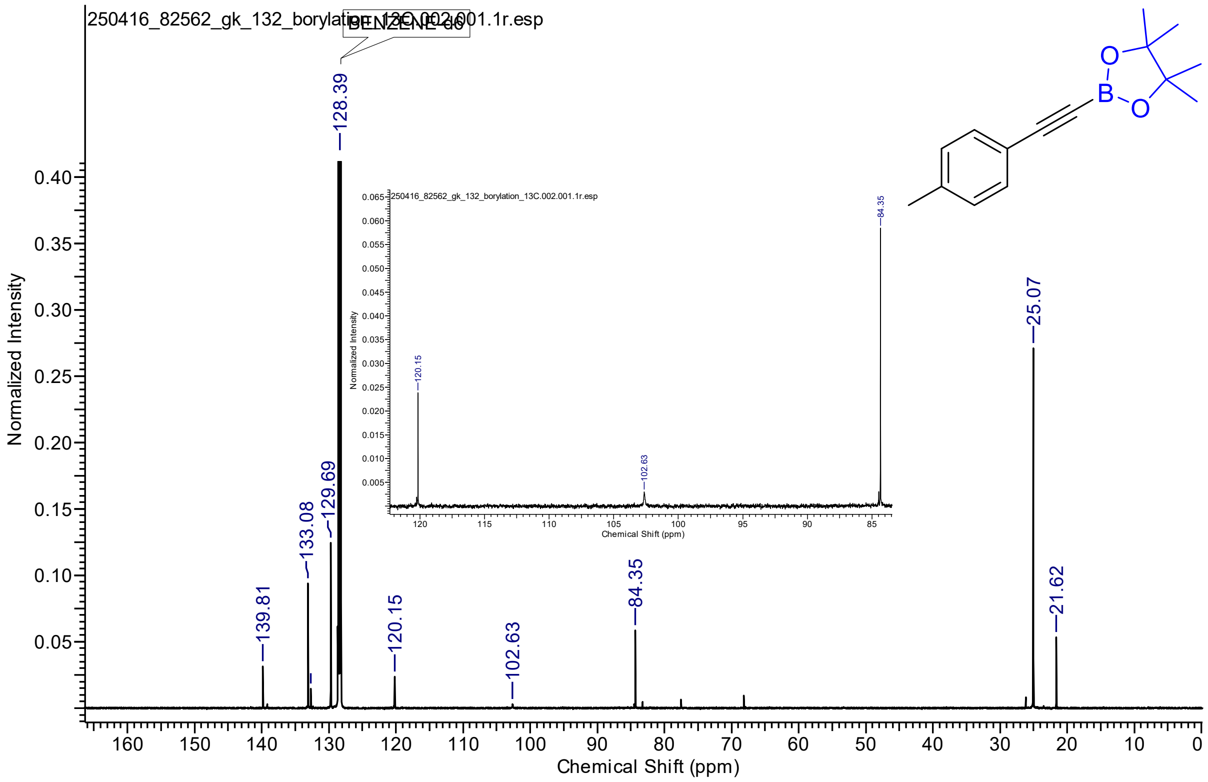

Figure S124. ^{13}C NMR spectrum of **52** in C_6D_6.

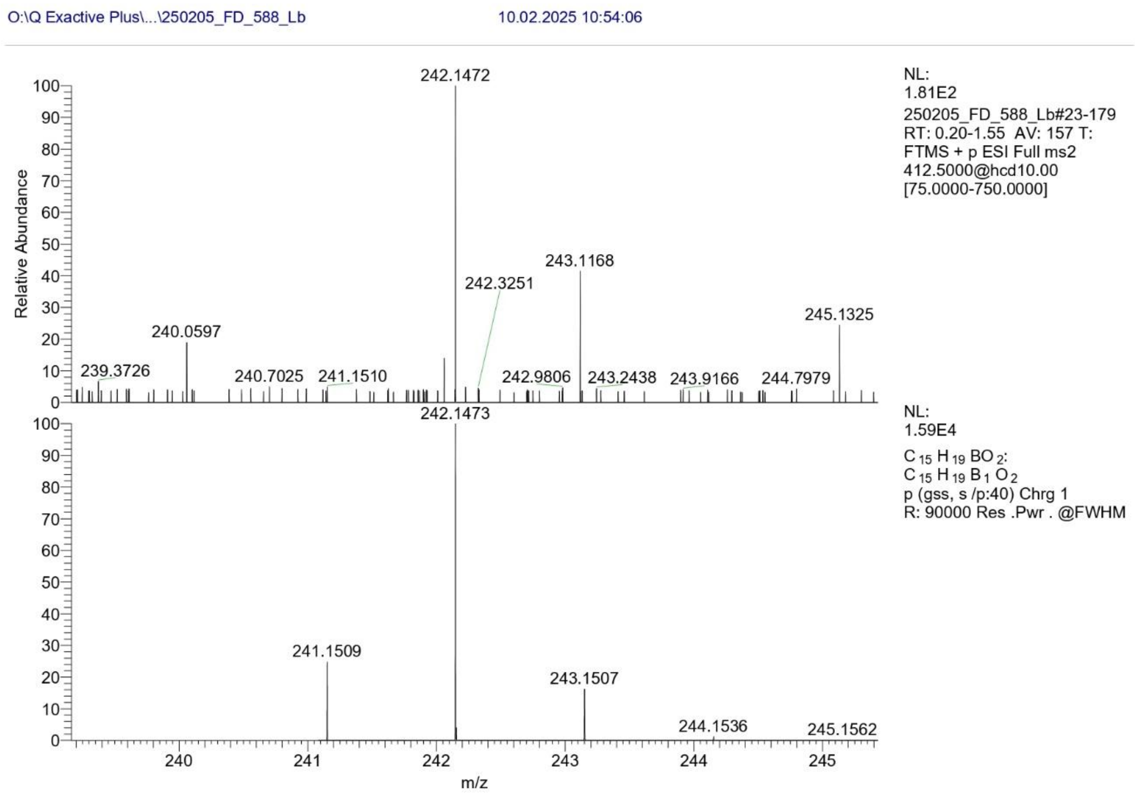

Figure S125. HR-MS spectrum of **52**.

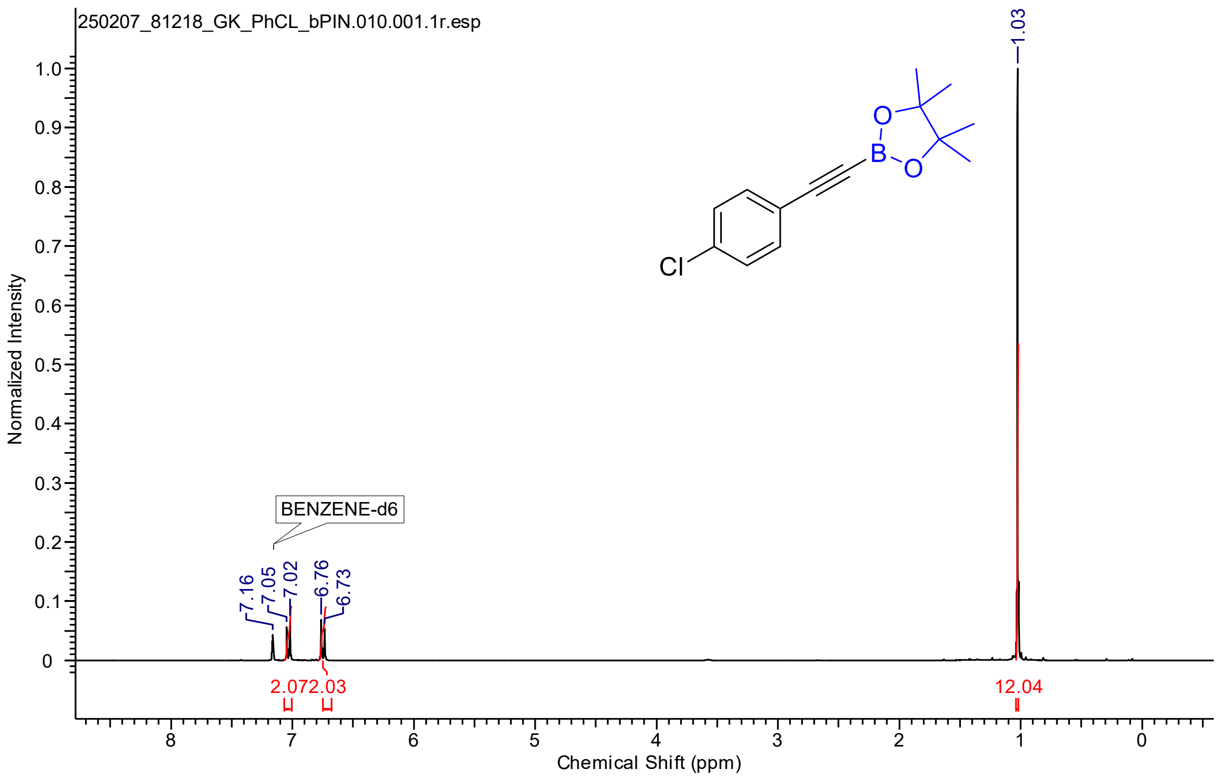

Figure S126. ^{1}H NMR spectrum of **53** in C_6D_6.

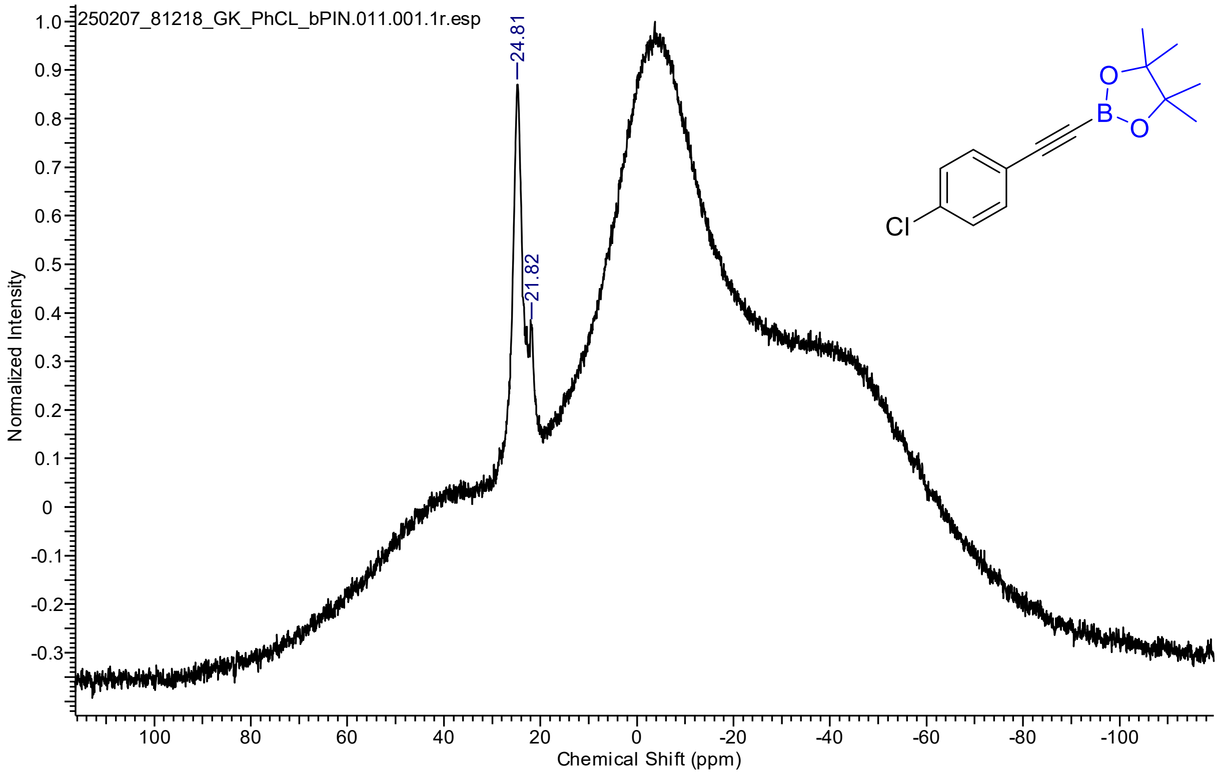

Figure S127. ^{11}B NMR spectrum of **53** in C_6D_6.

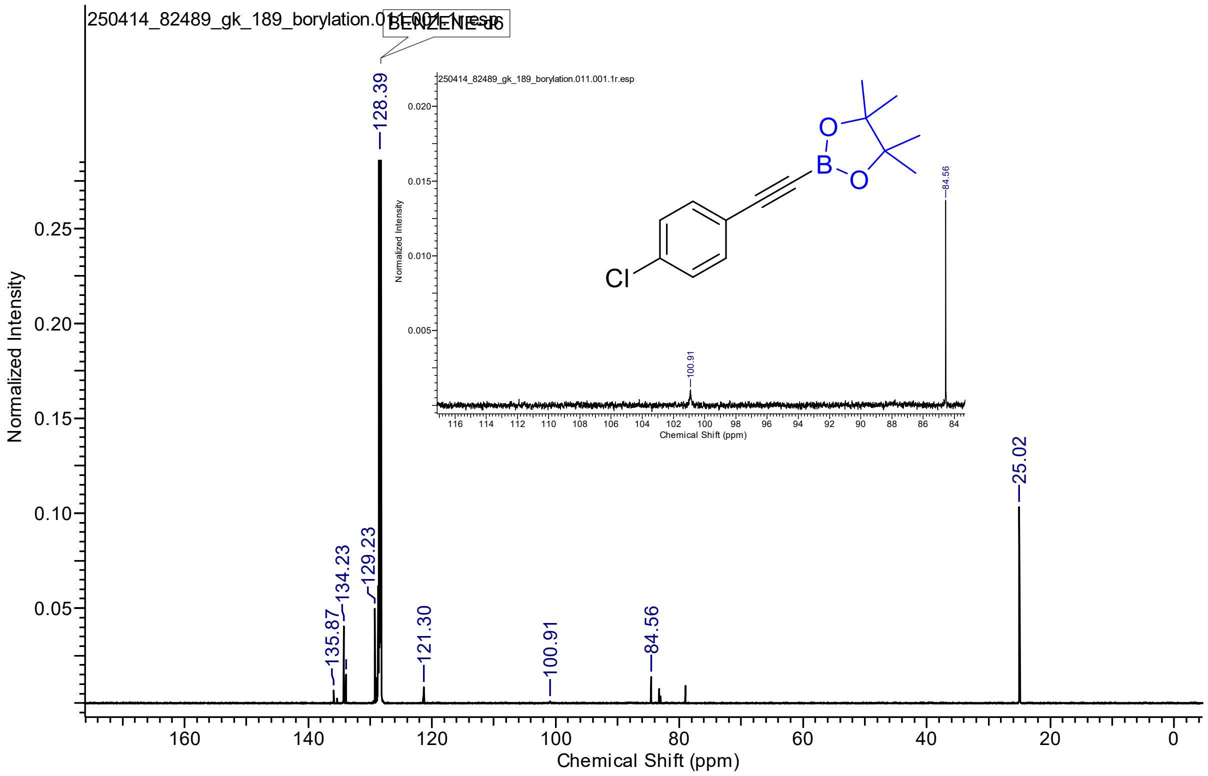

Figure S128. ^{13}C NMR spectrum of **53** in C_6D_6.

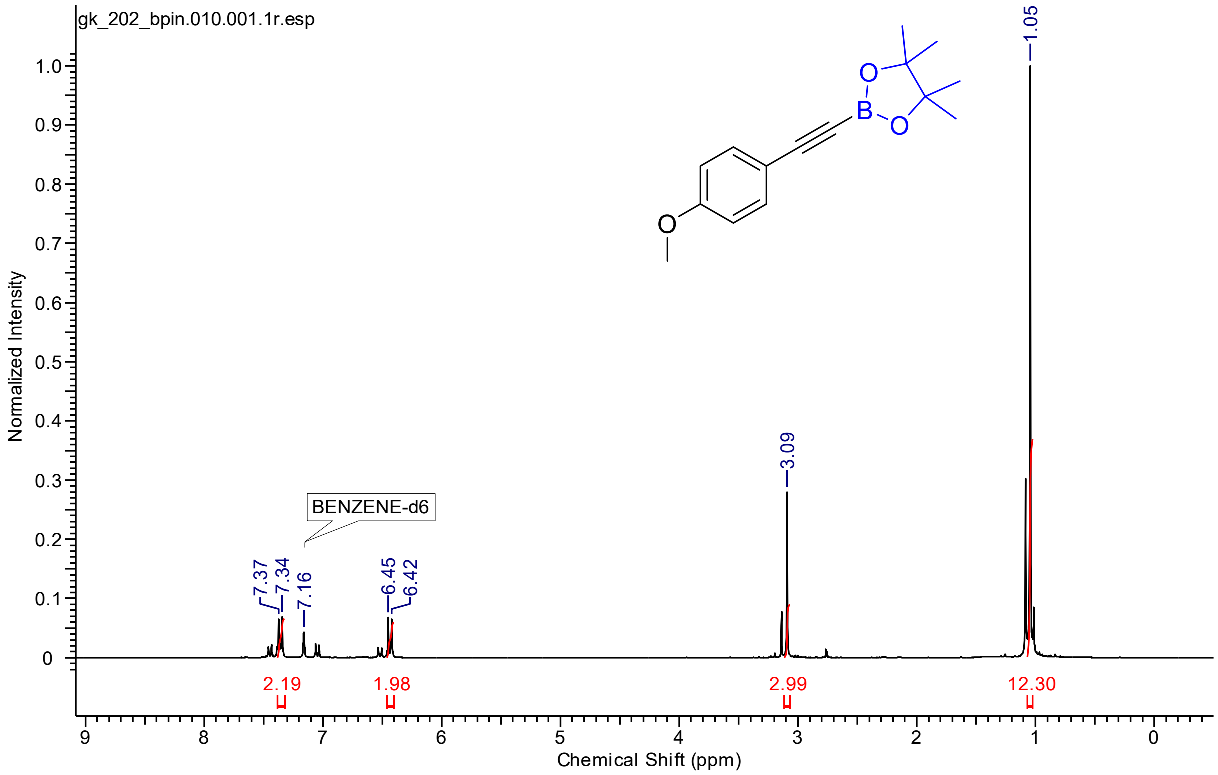

Figure S129. ^{1}H NMR spectrum of **54** in C_6D_6.

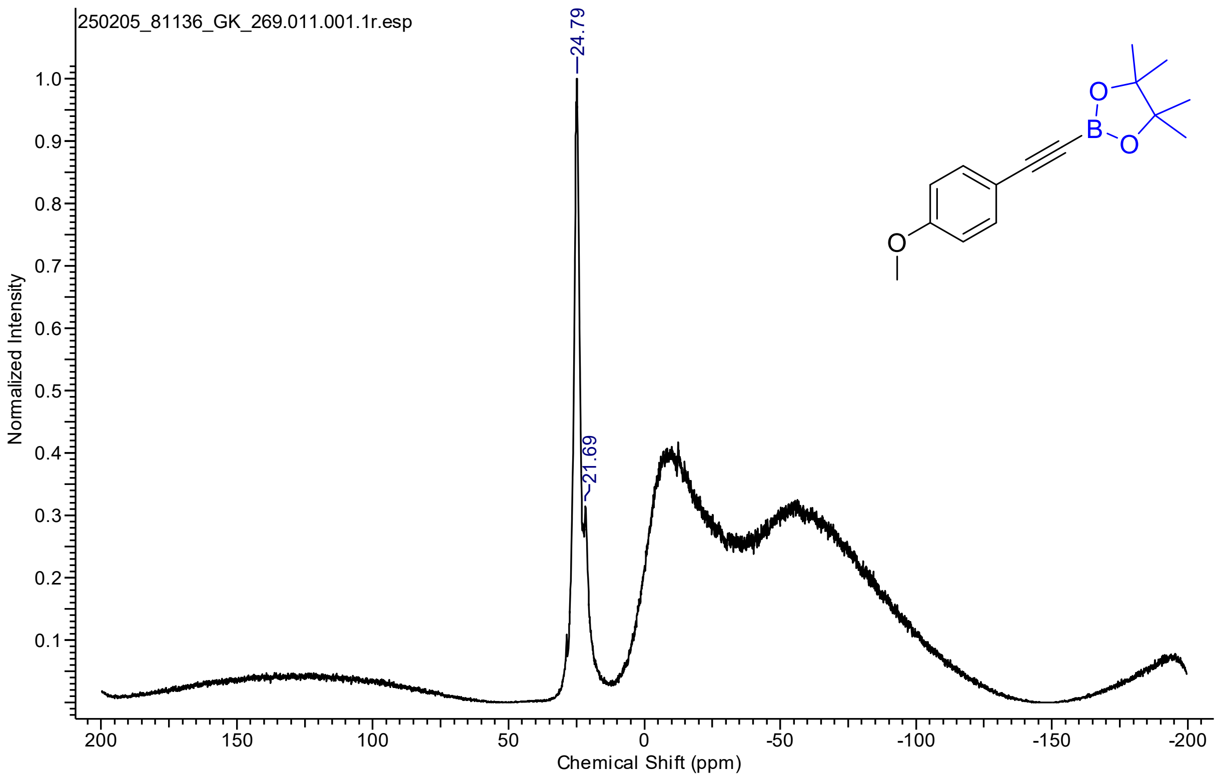

Figure S130. ^{11}B NMR spectrum of **54** in C_6D_6.

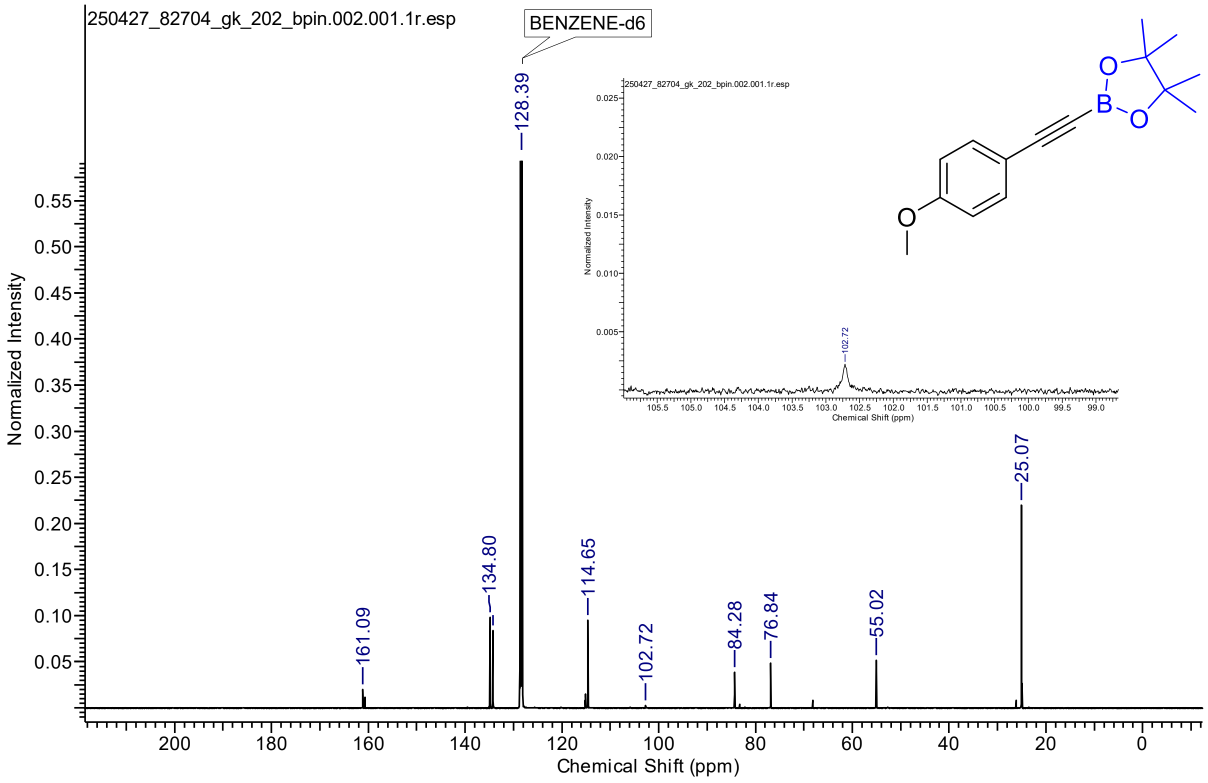

Figure S131. ^{13}C NMR spectrum of **54** in C_6D_6.

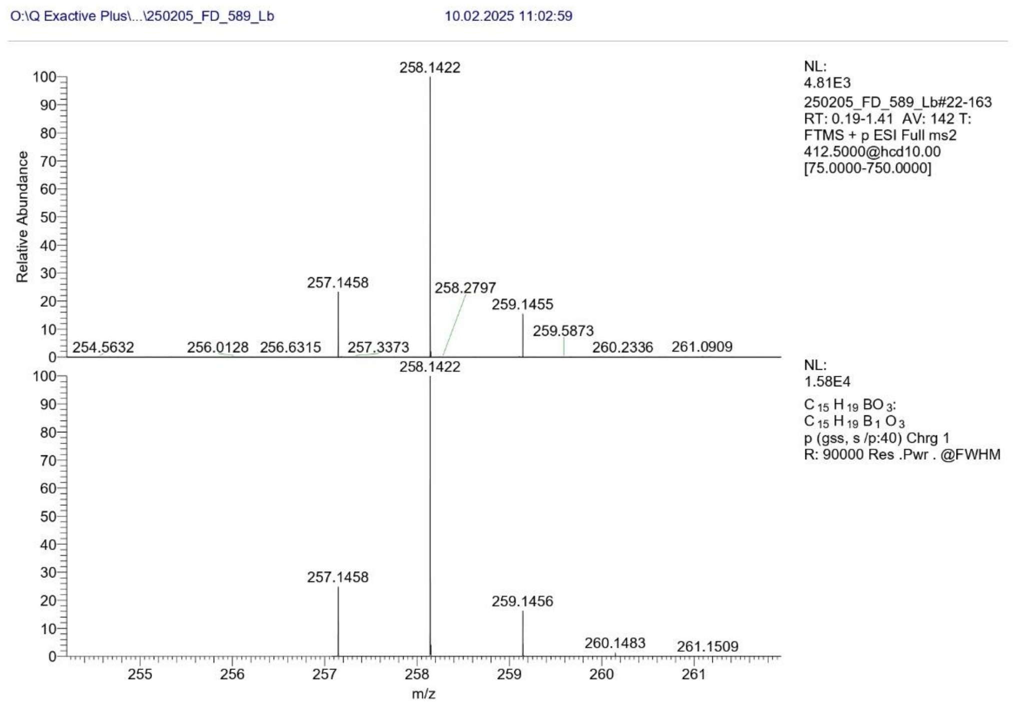

Figure S132. HR-MS spectrum of **54**.

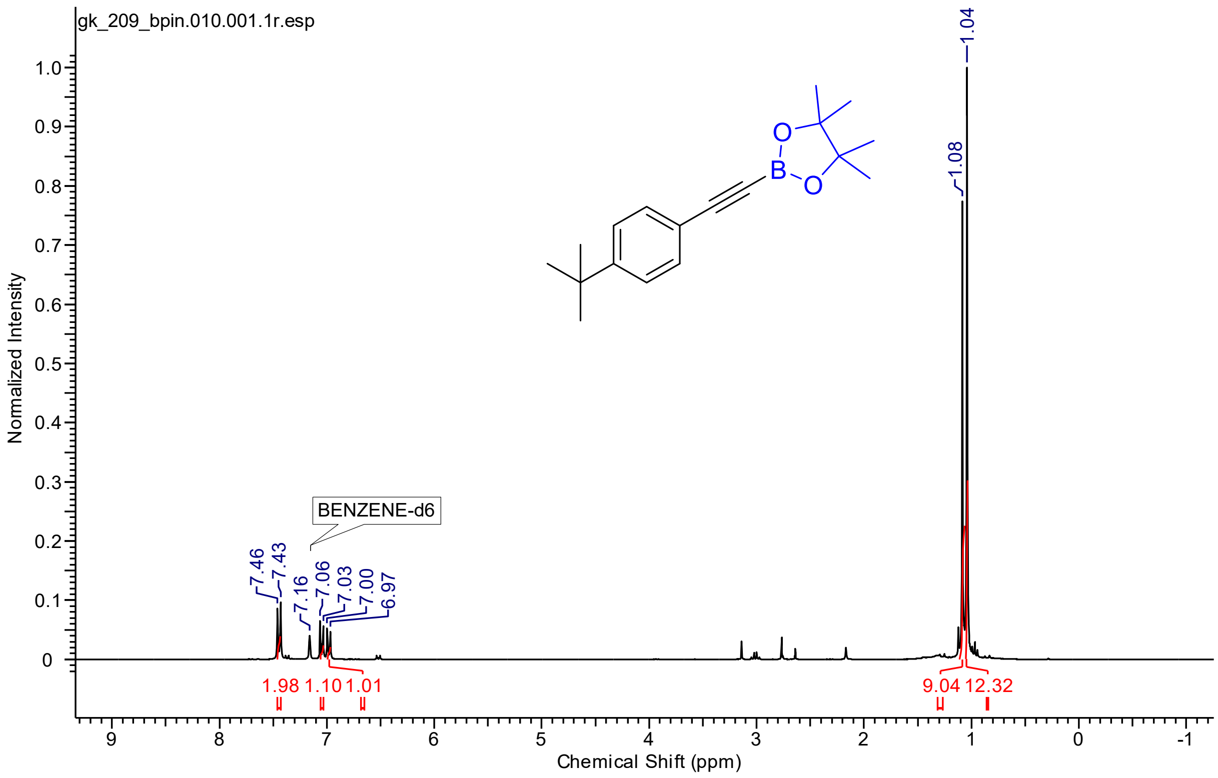

Figure S133. ¹H NMR spectrum of **55** in C_6D_6.

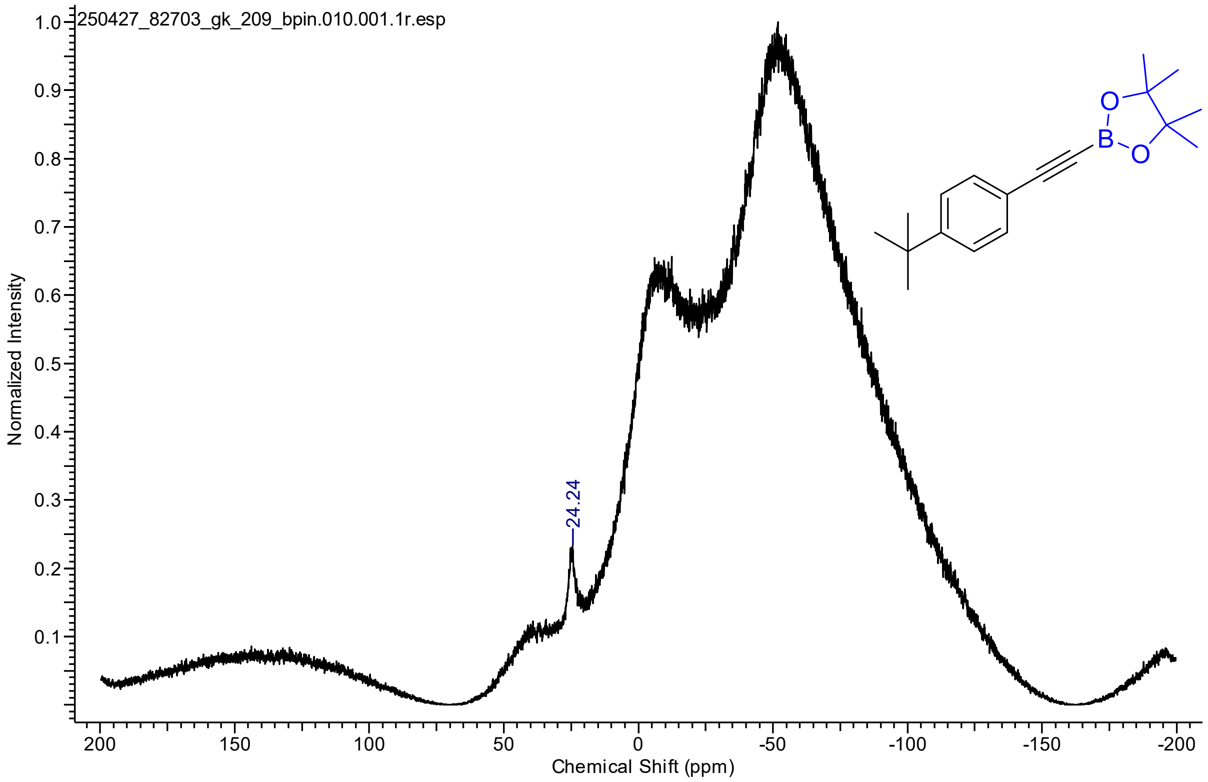

Figure S134. ^{11}B NMR spectrum of **55** in C_6D_6.

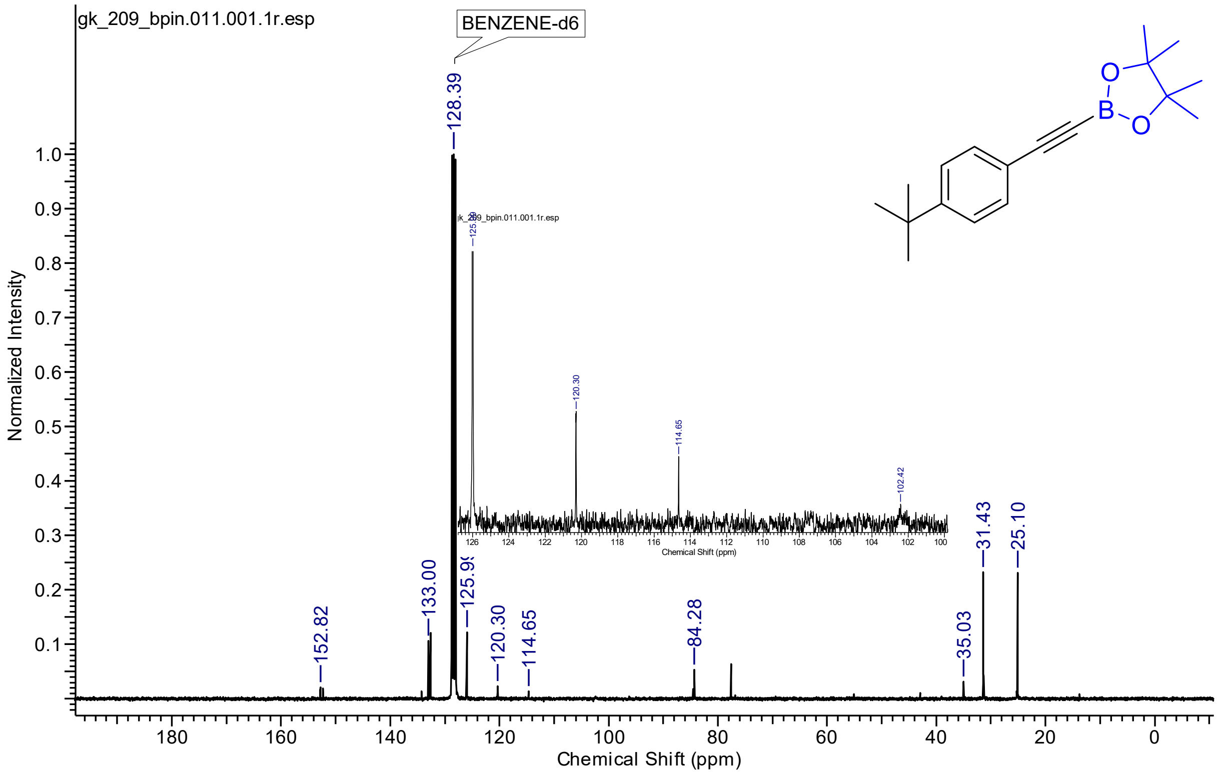

Figure S135. ^{13}C NMR spectrum of **55** in C_6D_6.

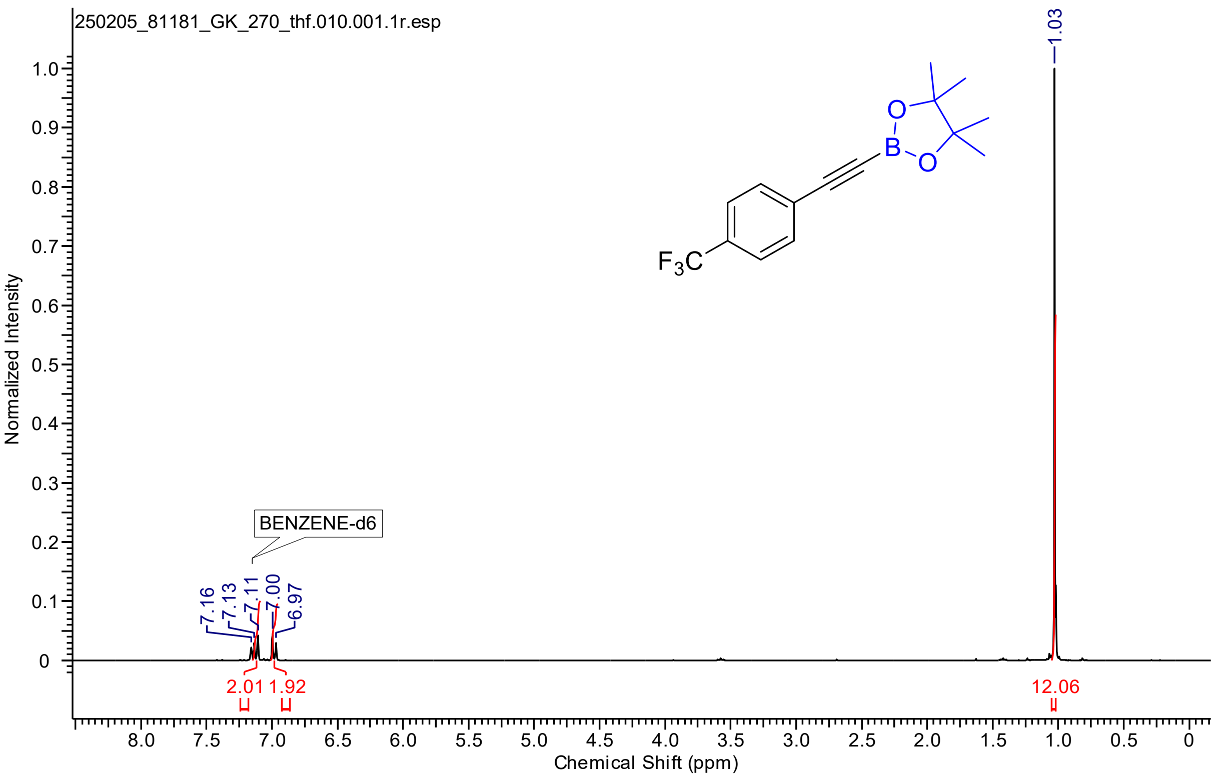

Figure S136. ^{1}H NMR spectrum of **56** in C_6D_6.

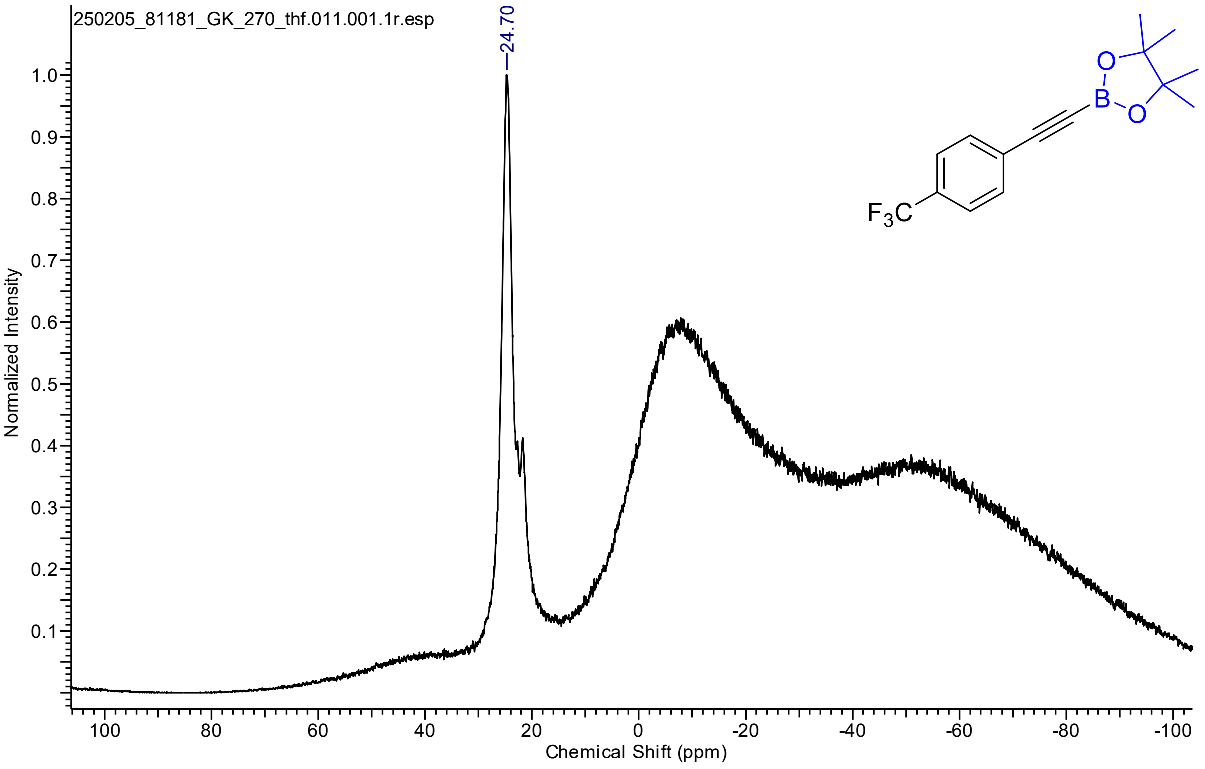

Figure S137. ^{11}B NMR spectrum of **56** in C_6D_6.

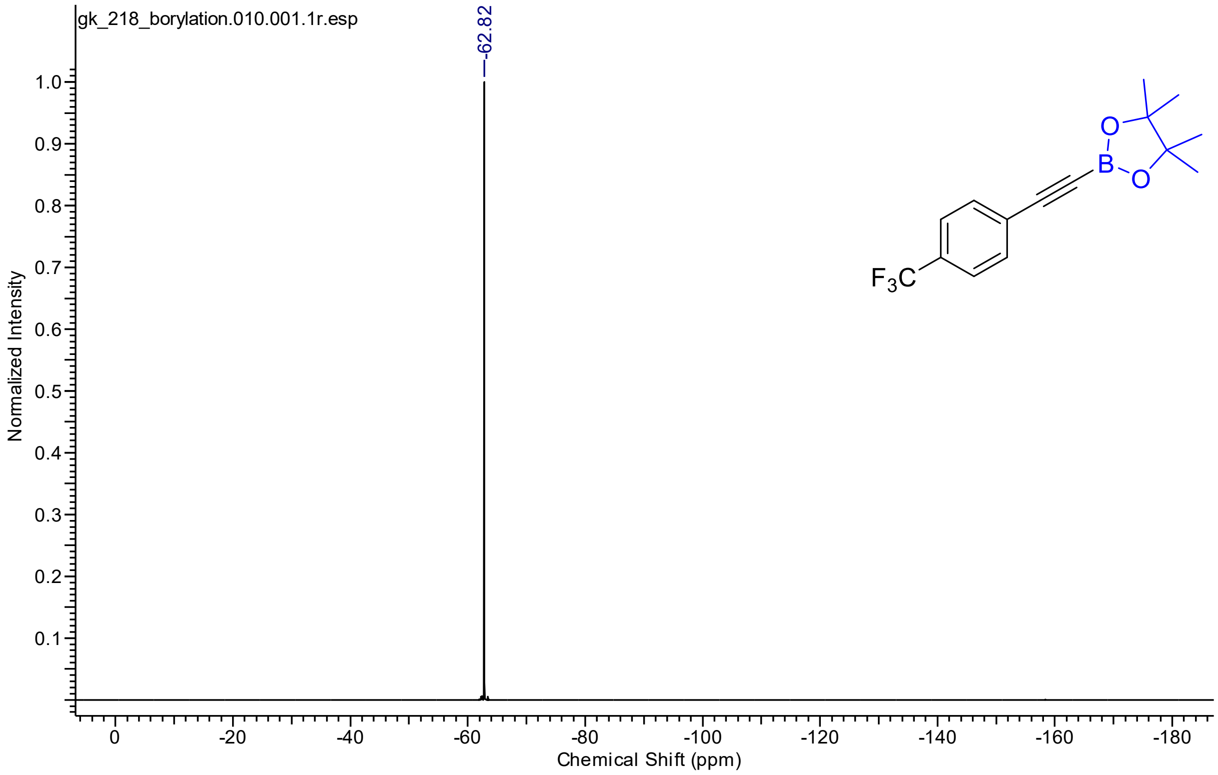

Figure S138. ^{19}F NMR spectrum of **56** in C_6D_6.

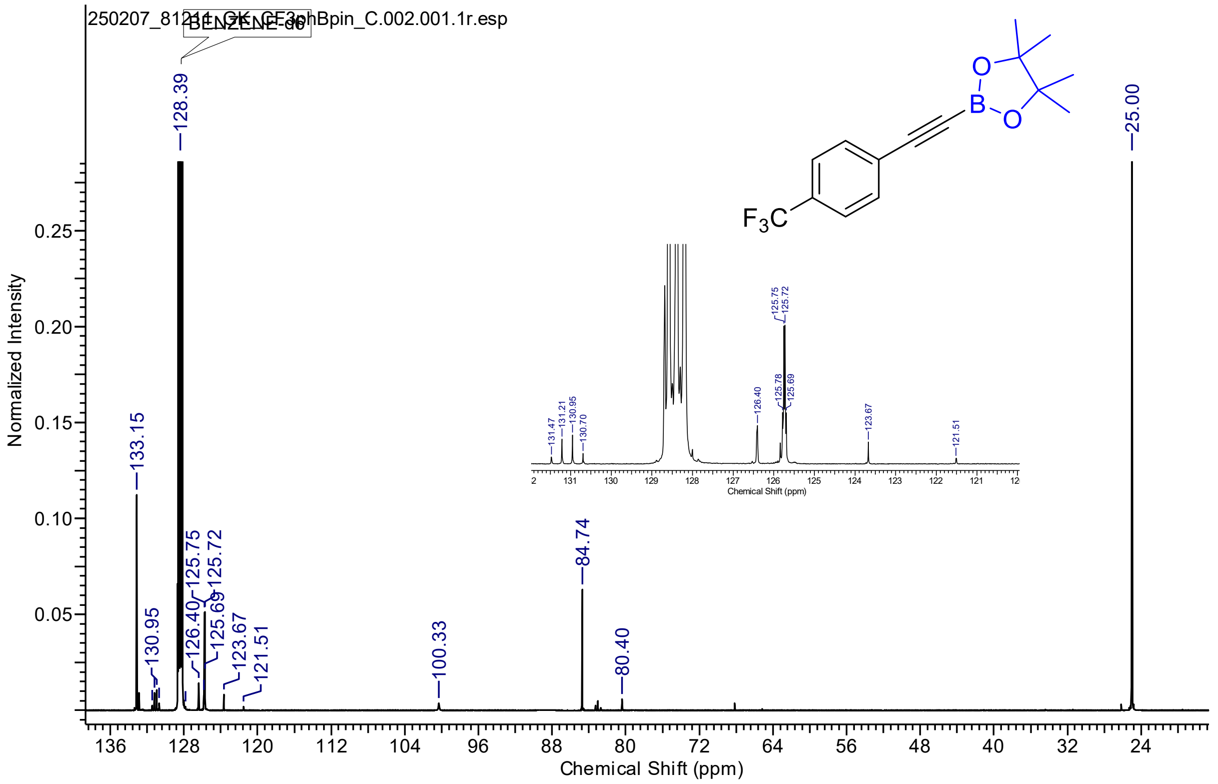

Figure S139. ^{13}C NMR spectrum of **56** in C_6D_6.

EPR spectroscopy

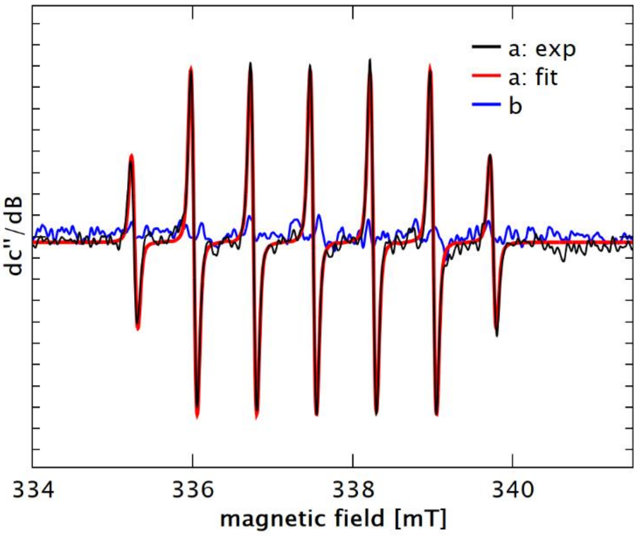

Figure S140. a: Experimental (black) and simulated (red) continuous-wave (CW) X-band EPR spectra of a solution containing 1 equiv. Bi(C≡CSiMe$_3$)$_3$ (c = 2.67·10^{-2} mol/L), 1.5 equiv. HBpin, and 3 equiv. PBN in THF, measured immediately after preparation. The observed resonance shows coupling constants of a(1 × ^{14}N) = 41.9 MHz (14.9 G, 1.49 mT), a(2 × ^{1}H) = 20.8 MHz (7.43 G, 0.743 mT), and a g_{iso} value of 2.0051. Spectrometer settings: microwave frequency = 9.473329 GHz, 0.05 mT modulation amplitude at 100 kHz, microwave power = 10 mW, number of accumulated scans = 1, conversion time = 2 ms. b: Experimental (blue) continuous-wave (CW) X-band EPR spectra of a solution containing 1 equiv. HBpin (c = 7.99·10^{-2} mol/L) and 2 equiv. PBN in THF, measured immediately after preparation. Spectrometer settings: microwave frequency = 9.473051 GHz, 0.05 mT modulation amplitude at 100 kHz, microwave power = 10 mW, number of accumulated scans = 1, conversion time = 2 ms.

These data for the spin adduct between an H atom and PBN are in agreement with the literature.[89]

11. Bonding parameters of from single-crystal XRD data

Table S6. Bond lengths for **2**.

Atom	Atom	Length/Å		Atom	Atom	Length/Å
Bi1	C1^1	2.206(13)		Si1	C4	1.844(13)

Bi1	C1	2.206(13)		Si1	C5	1.846(13)
Bi1	C1[2]	2.206(13)		Si1	C3	1.851(14)
Si1	C2	1.864(13)		C1	C2	1.208(16)

Table S7. Bond angles for **2**.

Atom	Atom	Atom	Angle/˚		Atom	Atom	Atom	Angle/˚
C1[1]	Bi1	C1[2]	90.2(5)		C3	Si1	C2	109.1(6)
C1[1]	Bi1	C1	90.2(5)		C3	Si1	C4	110.4(10)
C1[2]	Bi1	C1	90.2(5)		C3	Si1	C5	112.4(7)
C4	Si1	C2	107.8(7)		C1	C2	Si1	178.1(13)
C4	Si1	C5	110.4(7)		C2	C1	Bi1	174.5(11)
C5	Si1	C2	106.4(6)					

Table S8. Bond lengths for **3**.

Atom	Atom	Length/Å		Atom	Atom	Length/Å
Bi1	C2[1]	2.216(4)		C3	C4	1.442(5)
Bi1	C2	2.216(4)		C4	C5	1.513(4)
Bi1	C2[2]	2.216(4)		C4	C5[3]	1.513(4)
C2	C3	1.204(5)		C5	C5[3]	1.498(7)

Table S9. Bond angles for **3**.

Atom	Atom	Atom	Angle/˚		Atom	Atom	Atom	Angle/˚
C2[1]	Bi1	C2[2]	91.29(13)		C3	C4	C5	120.0(3)
C2[1]	Bi1	C2	91.29(13)		C3	C4	C5[3]	120.0(3)
C2[2]	Bi1	C2	91.29(13)		C5[3]	C4	C5	59.3(3)
C2	C3	C4	178.6(4)		C4[3]	C5	C5[3]	60.33(14)
C3	C2	Bi1	175.5(3)					

**Table S10.** Bond lengths for **4**.						
**Atom**	**Atom**	**Length/Å**		**Atom**	**Atom**	**Length/Å**
Bi1	C9	2.2154(19)		C14	C15	1.386(3)
Bi1	C17	2.220(2)		C13	C14	1.385(3)
Bi1	C1	2.2208(19)		C12	C13	1.385(3)
O1	C28	1.447(2)		C25	C26	1.498(3)
O1	C25	1.449(2)		C19	C20	1.401(3)
C9	C10	1.206(3)		C20	C21	1.385(3)
C17	C18	1.202(3)		C15	C16	1.386(3)
C10	C11	1.440(3)		C4	C5	1.386(3)
C2	C3	1.438(3)		C19	C24	1.397(3)
C1	C2	1.206(3)		C23	C24	1.383(3)
C11	C12	1.400(3)		C26	C27	1.528(3)
C11	C16	1.399(3)		C5	C6	1.384(3)
C27	C28	1.510(3)		C22	C23	1.382(3)
C3	C4	1.396(3)		C21	C22	1.385(3)
C3	C8	1.406(3)		C7	C8	1.383(3)
C18	C19	1.440(3)		C6	C7	1.384(3)

**Table S11.** Bond angles for **4**.								
**Atom**	**Atom**	**Atom**	**Angle/˚**		**Atom**	**Atom**	**Atom**	**Angle/˚**
C9	Bi1	C17	94.79(7)		O1	C25	C26	107.11(16)
C1	Bi1	C9	91.46(7)		C19	C20	C21	120.35(19)
C1	Bi1	C17	89.34(7)		C14	C15	C16	120.38(19)
C25	O1	C28	108.82(14)		C3	C4	C5	120.63(19)
C10	C9	Bi1	164.22(16)		C18	C19	C20	120.14(18)
C18	C17	Bi1	168.29(17)		C18	C19	C24	121.14(18)
C9	C10	C11	178.7(2)		C20	C19	C24	118.71(18)
C1	C2	C3	177.4(2)		C12	C13	C14	120.22(19)
C10	C11	C12	120.41(17)		C19	C24	C23	120.37(19)
C10	C11	C16	120.30(17)		C25	C26	C27	102.11(17)
C12	C11	C16	119.28(17)		C4	C5	C6	120.0(2)
O1	C28	C27	105.07(15)		C11	C16	C15	119.92(18)
C2	C3	C4	120.16(17)		C26	C27	C28	102.03(16)
C4	C3	C8	118.87(18)		C22	C23	C24	120.5(2)
C2	C3	C8	120.97(18)		C20	C21	C22	120.24(19)
C2	C1	Bi1	169.40(17)		C3	C8	C7	119.9(2)
C17	C18	C19	179.4(2)		C21	C22	C23	119.83(19)
C13	C14	C15	120.02(19)		C5	C6	C7	120.06(19)
C11	C12	C13	120.16(19)		C6	C7	C8	120.6(2)

**Table S12.** Bond lengths for **6**.						
**Atom**	**Atom**	**Length/Å**		**Atom**	**Atom**	**Length/Å**
Bi1	C1	2.211(3)		C18	C19	1.439(4)
Bi1	C9	2.219(3)		C25	C26	1.480(5)
Bi1	C17	2.219(3)		C19	C20	1.400(4)
C6	C11	1.735(3)		C20	C21	1.384(4)
Cl2	C14	1.736(3)		C3	C8	1.394(4)
Cl3	C22	1.744(3)		C5	C6	1.379(5)
O1	C28	1.449(4)		C19	C24	1.389(5)
O1	C25	1.447(4)		C21	C22	1.379(5)
C1	C2	1.203(4)		C22	C23	1.381(5)
C9	C10	1.203(4)		C11	C16	1.394(5)
C17	C18	1.200(4)		C11	C12	1.398(5)
C10	C11	1.442(4)		C23	C24	1.390(5)
C27	C28	1.497(5)		C14	C15	1.380(5)
C7	C8	1.382(4)		C15	C16	1.387(5)
C6	C7	1.383(5)		C13	C14	1.378(5)
C3	C4	1.392(4)		C12	C13	1.389(5)
C4	C5	1.390(4)		C26	C27	1.404(6)
C2	C3	1.450(4)				

**Table S13.** Bond angles for **6**.								
**Atom**	**Atom**	**Atom**	**Angle/˚**		**Atom**	**Atom**	**Atom**	**Angle/˚**
C1	Bi1	C9	93.28(12)		C2	C3	C4	119.8(3)
C1	Bi1	C17	92.50(11)		C4	C3	C8	119.4(3)
C9	Bi1	C17	88.77(12)		C2	C3	C8	120.8(3)
C25	O1	C28	108.1(2)		C13	C22	C22	119.3(3)
C2	C1	Bi1	166.9(3)		C21	C22	C23	121.6(3)
C10	C9	Bi1	172.4(3)		C13	C22	C23	119.1(3)
C18	C17	Bi1	169.4(3)		C10	C11	C16	120.5(3)
C9	C10	C11	178.0(3)		C12	C11	C16	119.3(3)
O1	C28	C27	105.5(3)		C10	C11	C12	120.2(3)
C6	C7	C8	118.9(3)		C20	C21	C22	119.2(3)
C3	C4	C5	120.1(3)		C4	C5	C6	119.3(3)
C1	C2	C3	179.6(3)		C19	C24	C23	120.7(3)
C17	C18	C19	178.5(3)		C22	C23	C24	119.0(3)
O1	C25	C26	106.5(3)		C14	C15	C16	119.3(3)
C19	C20	C21	120.6(3)		C12	C14	Cl5	119.5(3)
C3	C8	C7	120.7(3)		C12	C14	Cl3	119.0(3)
C7	C6	Cl1	119.3(3)		C13	C14	C15	121.5(3)
C5	C6	Cl1	119.1(3)		C11	C16	C15	120.4(3)
C5	C6	C7	121.5(3)		C11	C12	C13	120.2(3)
C18	C19	C20	119.4(3)		C12	C13	C14	119.4(3)
C18	C19	C24	121.6(3)		C26	C27	C28	106.1(3)
C20	C19	C24	119.0(3)		C25	C26	C27	107.9(4)

Table S14. Bond lengths for 7.						
Atom	Atom	Length/Å		Atom	Atom	Length/Å
Bi1	C19	2.244(4)		C3	C4	1.394(5)
Bi1	C10	2.199(4)		C3	C8	1.397(5)
Bi1	C1	2.242(4)		C25	C26	1.375(5)
O2	C18	1.428(5)		C24	C25	1.398(5)
O2	C15	1.368(4)		C4	C5	1.385(5)
O3	C27	1.428(5)		C5	C6	1.388(5)
O3	C24	1.361(4)		C12	C13	1.390(6)
C19	C20	1.201(5)		C12	C17	1.392(6)
O1	C9	1.429(5)		C23	C24	1.388(5)
O1	C6	1.360(5)		C22	C23	1.387(5)
C10	C11	1.199(5)		C6	C7	1.391(5)
C1	C2	1.202(6)		C13	C14	1.391(5)
C2	C3	1.442(6)		C14	C15	1.376(6)
C20	C21	1.439(5)		C15	C16	1.391(5)
C11	C12	1.445(5)		C16	C17	1.380(5)
C21	C26	1.400(5)		C7	C8	1.375(6)
C21	C22	1.395(5)				

Table S15. Bond angles for 7.								
Atom	Atom	Atom	Angle/˚		Atom	Atom	Atom	Angle/˚
C10	Bi1	C19	93.15(14)		C4	C5	C6	119.2(4)
C10	Bi1	C1	92.86(14)		C11	C12	C13	120.5(4)
C1	Bi1	C19	91.80(14)		C13	C12	C17	118.7(4)
C15	O2	C18	117.3(3)		C11	C12	C17	120.8(4)
C24	O3	C27	118.4(3)		O3	C24	C25	115.2(3)
C20	C19	Bi1	161.7(3)		O3	C24	C23	125.1(4)
C6	O1	C9	118.2(3)		C23	C24	C25	119.7(4)
C11	C10	Bi1	168.5(3)		C22	C23	C24	119.5(4)
C2	C1	Bi1	163.8(3)		O1	C6	C5	124.5(4)
C1	C2	C3	179.9(5)		O1	C6	C7	115.6(4)
C19	C20	C21	176.4(4)		C5	C6	C7	119.8(4)
C10	C11	C12	178.6(5)		C21	C22	C23	121.5(4)
C20	C21	C26	119.0(4)		C12	C13	C14	120.9(4)
C20	C21	C22	122.9(4)		O2	C15	C14	124.6(4)
C22	C21	C26	118.1(4)		O2	C15	C16	115.1(4)
C2	C3	C4	120.7(4)		C14	C15	C16	120.2(4)
C4	C3	C8	118.4(4)		C13	C14	C15	119.5(4)
C2	C3	C8	120.9(4)		C12	C17	C16	120.5(4)
C24	C25	C26	120.3(4)		C3	C8	C7	120.3(4)
C21	C26	C25	120.9(4)		C6	C7	C8	120.7(4)
C3	C4	C5	121.6(4)		C15	C16	C17	120.1(4)

Table S16. Bond lengths for **27**.

Atom	Atom	Length/Å		Atom	Atom	Length/Å
Si1	C22	1.898(3)		C2	C3	1.526(4)
Si1	C25	1.872(3)		C10	C11	1.528(4)
Si1	C23	1.857(3)		C10	C14	1.388(4)
Si1	C24	1.869(3)		C5	C6	1.539(4)
N1	C1	1.407(3)		C6	C7	1.524(4)
N1	C9	1.438(3)		C6	C8	1.540(4)
N1	C6	1.483(3)		C11	C13	1.529(4)
C22	C22[1]	1.522(5)		C11	C12	1.534(4)
C22	C21	1.318(4)		C16	C17	1.391(4)
C1	C21	1.317(4)		C17	C18	1.520(4)
C1	C2	1.544(3)		C15	C16	1.378(4)
C9	C10	1.408(4)		C14	C15	1.384(4)
C9	C17	1.409(4)		C18	C19	1.530(4)
C2	C5	1.539(4)		C18	C20	1.527(4)
C2	C4	1.531(4)				

Table S17. Bond angles for **27**.

Atom	Atom	Atom	Angle/°		Atom	Atom	Atom	Angle/°
C25	Si1	C22	110.73(12)		C3	C2	C4	108.1(2)
C23	Si1	C22	108.10(13)		C9	C10	C11	122.6(2)
C23	Si1	C25	110.30(15)		C9	C10	C14	118.7(2)
C23	Si1	C24	106.36(17)		C11	C10	C14	118.6(2)
C24	Si1	C22	114.15(14)		N1	C6	C5	101.40(19)
C24	Si1	C25	107.10(14)		N1	C6	C7	112.2(2)
C1	N1	C9	122.0(2)		N1	C6	C8	111.3(2)
C1	N1	C6	111.2(2)		C5	C6	C8	110.9(2)
C6	N1	C9	125.5(2)		C5	C6	C7	112.5(2)
C22[1]	C22	Si1	119.31(18)		C7	C6	C8	108.4(2)
C21	C22	Si1	116.75(19)		C10	C11	C13	111.0(2)
C21	C22	C22[1]	123.9(2)		C10	C11	C12	112.4(2)
N1	C1	C2	108.6(2)		C12	C11	C13	110.5(2)
C21	C1	N1	127.3(2)		C9	C17	C18	122.0(2)
C2	C1	C21	124.1(2)		C9	C17	C16	118.5(2)
C1	C21	C22	168.9(3)		C16	C17	C18	119.5(2)
C10	C9	N1	119.7(2)		C15	C16	C17	121.6(3)
C10	C9	C17	120.4(2)		C10	C14	C15	121.3(3)
C17	C9	N1	119.9(2)		C17	C18	C19	111.9(2)
C1	C2	C5	102.8(2)		C17	C18	C20	111.1(2)
C1	C2	C4	111.3(2)		C19	C18	C20	110.6(3)
C4	C2	C5	111.0(2)		C2	C5	C6	107.5(2)
C1	C2	C3	111.4(2)		C14	C15	C16	119.5(3)
C3	C2	C5	112.2(2)					

Table S18. Bond lengths for **29**.

Atom	Atom	Length/Å		Atom	Atom	Length/Å
N2	C40	1.434(2)		C8	C9	1.527(3)
N2	C32	1.395(2)		C41	C42	1.518(3)
N2	C37	1.489(2)		C41	C45	1.397(3)
N1	C3	1.391(2)		C24	C25	1.394(3)
N1	C11	1.431(2)		C25	C26	1.382(3)
N1	C8	1.492(2)		C53	C54	1.390(3)
C2	C3	1.320(3)		C54	C55	1.378(3)
C3	C4	1.530(2)		C26	C27	1.503(3)
C19	C20	1.523(3)		C55	C57	1.397(3)
C20	C22	1.535(3)		C55	C56	1.508(3)
C20	C21	1.533(3)		C17	C18	1.384(3)
C1	C2	1.321(3)		C48	C49	1.524(3)
C30	C52	1.488(3)		C47	C48	1.395(3)
C52	C53	1.392(3)		C57	C58	1.379(3)
C52	C58	1.400(3)		C33	C36	1.541(3)
C1	C1	1.497(3)		C33	C34	1.538(3)
C23	C29	1.397(3)		C33	C35	1.529(3)
C23	C24	1.393(3)		C16	C17	1.381(3)
C4	C5	1.531(3)		C42	C44	1.528(3)
C4	C6	1.534(3)		C42	C43	1.535(3)
C4	C7	1.538(3)		C36	C37	1.532(3)
C31	C32	1.312(3)		C37	C38	1.530(3)
C30	C31	1.318(3)		C37	C39	1.533(3)
C40	C41	1.407(3)		C49	C50	1.535(3)
C40	C48	1.407(3)		C49	C51	1.528(3)
C11	C12	1.412(3)		C13	C14	1.529(3)
C11	C19	1.409(3)		C13	C15	1.529(3)
C1	C30	1.518(3)		C45	C46	1.383(3)
C32	C33	1.532(3)		C46	C47	1.377(3)
C28	C29	1.380(3)		C62[1]	C64	1.386(3)
C12	C16	1.393(3)		C63	C64	1.382(3)
C12	C13	1.520(3)		C62	C63	1.377(4)
C18	C19	1.391(3)		C60	C61	1.380(4)
C26	C28	1.397(3)		C59	C60	1.383(3)
C7	C8	1.538(3)		C59[2]	C61	1.376(4)
C8	C10	1.534(3)				

**Table S19.** Bond Angles for **29**.								
**Atom**	**Atom**	**Atom**	**Angle/˚**		**Atom**	**Atom**	**Atom**	**Angle/˚**
C37	N2	C40	125.77(15)		C9	C8	C10	107.63(17)
C32	N2	C40	122.55(15)		C40	C41	C42	122.66(17)
C32	N2	C37	111.38(15)		C45	C41	C40	118.31(19)
C3	N1	C11	123.41(15)		C45	C41	C42	118.99(18)
C3	N1	C8	113.61(15)		C26	C25	C24	121.29(19)
C8	N1	C11	122.38(15)		C54	C53	C52	121.31(18)
N1	C3	C4	109.01(15)		C23	C24	C25	120.86(18)
C2	C3	N1	126.95(17)		C55	C54	C53	121.50(19)
C2	C3	C4	124.03(17)		C28	C26	C27	120.72(19)
C19	C20	C22	113.23(15)		C25	C26	C28	117.82(18)
C19	C20	C21	109.43(16)		C25	C26	C27	121.44(19)
C21	C20	C22	109.81(16)		C54	C55	C57	117.34(19)
C1	C2	C3	165.19(19)		C54	C55	C56	121.7(2)
C30	C52	C53	121.49(17)		C56	C55	C57	121.0(2)
C53	C52	C58	117.22(18)		C17	C18	C19	121.49(18)
C30	C52	C58	121.23(17)		C40	C48	C49	123.37(17)
C1	C23	C29	120.41(17)		C40	C48	C47	118.36(19)
C1	C23	C24	121.70(17)		C47	C48	C49	118.21(18)
C24	C23	C29	117.66(17)		C52	C58	C57	120.97(19)
C3	C4	C5	110.94(15)		C4	C7	C8	108.12(15)
C3	C4	C6	112.05(15)		C32	C33	C36	102.42(15)
C3	C4	C7	101.55(15)		C32	C33	C34	111.41(16)
C5	C4	C6	108.89(16)		C34	C33	C36	112.09(17)
C5	C4	C7	112.21(16)		C32	C33	C35	109.46(16)
C6	C4	C7	111.11(15)		C35	C33	C36	112.04(17)
C30	C31	C32	172.48(19)		C34	C33	C35	109.27(17)
C41	C40	N2	117.84(17)		C12	C16	C17	121.10(19)
C41	C40	C48	120.88(17)		C16	C17	C18	119.58(19)
C48	C40	N2	121.24(17)		C41	C42	C44	110.72(17)
C12	C11	N1	119.96(16)		C41	C42	C43	112.45(18)
C19	C11	N1	120.08(16)		C43	C42	C44	110.48(18)
C12	C11	C19	119.96(17)		N2	C37	C36	100.61(15)
C2	C1	C23	121.47(17)		N2	C37	C38	112.35(16)
C2	C1	C30	120.24(17)		N2	C37	C39	111.65(16)
C23	C1	C30	118.23(15)		C36	C37	C39	111.96(17)
N2	C32	C33	108.94(15)		C36	C37	C39	112.23(17)
C31	C32	N2	127.99(17)		C38	C37	C39	108.01(17)
C31	C32	C33	123.05(17)		C48	C49	C50	111.01(17)
C1	C30	C52	117.41(16)		C48	C49	C51	111.67(18)
C31	C30	C52	122.22(17)		C50	C49	C51	109.40(17)
C1	C30	C31	120.37(17)		C33	C36	C37	107.56(15)
C23	C29	C28	121.16(18)		C12	C13	C14	108.97(17)
C11	C12	C13	122.90(17)		C12	C13	C15	112.93(17)
C11	C12	C16	118.91(18)		C14	C13	C15	110.33(18)
C13	C12	C16	118.06(17)		C41	C45	C46	121.1(2)
C11	C19	C20	123.10(17)		C55	C57	C58	121.67(19)
C18	C19	C20	118.06(17)		C45	C46	C47	120.1(2)
C11	C19	C18	118.65(17)		C46	C47	C48	121.2(2)
C26	C28	C29	121.17(18)		C62[1]	C64	C63	120.1(2)

N1	C8	C7	101.29(15)		C63	C62	C64[1]	120.1(2)
N1	C8	C10	113.22(16)		C59	C60	C61	119.8(2)
N1	C8	C9	110.99(16)		C62	C63	C64	119.7(2)
C7	C8	C10	112.59(17)		C59[2]	C61	C60	119.7(2)
C7	C8	C9	111.12(16)		C60	C59	C61[1]	120.5(2)

**Table S20.** Bond Lengths for **36**.						
**Atom**	**Atom**	**Length/Å**		**Atom**	**Atom**	**Length/Å**
Se1	C1	1.830(4)		C6	C7	1.507(6)
Se1	C10	1.929(4)		C10	C15	1.385(6)
C4	C5	1.382(7)		C1	C2	1.200(6)
C5	C6	1.382(8)		C6	C8	1.399(6)
C8	C9	1.383(6)		C2	C3	1.438(6)
C3	C9	1.396(6)		C10	C11	1.391(6)
C3	C4	1.396(6)		C11	C12	1.383(6)
C14	C15	1.386(6)		C12	C13	1.393(6)
C13	C14	1.372(7)				

**Table S21.** Bond angles for **36**.								
**Atom**	**Atom**	**Atom**	**Angle/˚**		**Atom**	**Atom**	**Atom**	**Angle/˚**
C1	Se1	C10	99.26(18)		C2	C3	C4	120.5(4)
C4	C5	C6	121.8(4)		C10	C11	C12	119.2(4)
C3	C9	C8	120.6(4)		C15	C10	Se1	115.8(3)
C3	C4	C5	120.1(4)		C15	C10	C11	120.9(4)
C13	C14	C15	121.0(4)		C11	C10	Se1	123.3(3)
C10	C15	C14	119.0(4)		C11	C12	C13	120.3(4)
C2	C1	Se1	179.2(4)		C5	C6	C7	120.9(3)
C6	C8	C9	120.8(4)		C5	C6	C8	118.1(4)
C1	C2	C3	178.6(5)		C7	C6	C8	121.0(4)
C4	C3	C9	118.6(4)		C12	C13	C14	119.7(4)
C2	C3	C9	120.9(4)					

**Table S22.** Bond Lengths for **37**.						
**Atom**	**Atom**	**Length/Å**		**Atom**	**Atom**	**Length/Å**
Se1	C1	1.8250(17)		C3	C8	1.405(2)
Se1	C9	1.9202(16)		C4	C5	1.383(2)
F1	C00I	1.340(2)		C5	C6	1.393(2)
F3	C00I	1.331(2)		C10	C11	1.390(2)
F2	C00I	1.332(2)		C13	C14	1.388(2)
C1	C2	1.199(2)		C11	C12	1.388(3)
C9	C10	1.390(2)		C6	C7	1.391(2)
C9	C14	1.389(2)		C6	C00I	1.499(2)
C2	C3	1.431(2)		C7	C8	1.384(2)
C3	C4	1.401(2)		C12	C13	1.385(3)

**Table S23.** Bond angles for **37**.								
**Atom**	**Atom**	**Atom**	**Angle/˚**		**Atom**	**Atom**	**Atom**	**Angle/˚**
C1	Se1	C9	99.39(7)		C10	C11	C12	120.42(16)
C2	C1	Se1	175.35(15)		C5	C6	C00I	119.36(14)
C10	C9	Se1	122.79(12)		C5	C6	C7	120.69(15)
C14	C9	Se1	116.39(12)		C7	C6	C00I	119.94(14)
C10	C9	C14	120.81(15)		C6	C7	C8	119.77(15)
C1	C2	C3	175.92(18)		C11	C12	C13	120.12(16)
C2	C3	C4	120.12(15)		C3	C8	C7	120.15(15)
C4	C3	C8	119.31(14)		F1	C00I	C6	112.65(14)
C2	C3	C8	120.49(15)		F3	C00I	F1	105.61(14)
C3	C4	C5	120.41(15)		F3	C00I	F2	106.66(15)
C4	C5	C6	119.61(15)		F3	C00I	C6	112.80(14)
C9	C10	C11	119.02(15)		F2	C00I	F1	105.83(14)
C9	C14	C13	119.61(16)		F2	C00I	C6	112.71(14)

**Table S24.** Bond lengths for **56**.						
**Atom**	**Atom**	**Length/Å**		**Atom**	**Atom**	**Length/Å**
O1	C13	1.475(2)		C10	C13	1.564(2)
O1	B1	1.349(2)		C13	C15	1.512(3)
O2	C10	1.468(2)		C13	C14	1.516(2)
O2	B1	1.360(2)		C8	C9	1.384(2)
F1	C7	1.366(3)		C10	C11	1.525(3)
F2	C7	1.307(3)		C10	C12	1.512(3)
F3	C7	1.331(3)		C4	C5	1.382(2)
C3	C9	1.395(2)		C1	C2	1.205(2)
C3	C4	1.394(2)		C1	B1	1.534(3)
C2	C3	1.438(2)		C7	F6	1.358(9)
C5	C6	1.385(3)		C7	F5	1.305(13)
C6	C8	1.389(2)		C7	F4	1.183(10)
C6	C7	1.495(2)				

Table S25. Bond angles for **56**.								
**Atom**	**Atom**	**Atom**	**Angle/˚**		**Atom**	**Atom**	**Atom**	**Angle/˚**
B1	O1	C13	106.92(13)		C4	C5	C6	119.67(16)
B1	O2	C10	106.55(14)		C3	C4	C5	120.63(17)
C9	C3	C2	120.30(15)		C1	C2	C3	179.4(2)
C4	C3	C9	119.34(15)		C6	C8	C9	120.11(17)
C2	C3	C4	120.35(16)		C2	C1	B1	176.8(2)
C5	C6	C8	120.27(16)		F1	C7	C6	111.05(18)
C5	C6	C7	120.09(16)		F2	C7	F1	104.9(3)
C7	C6	C8	119.64(16)		F2	C7	F3	109.8(3)
O1	C13	C10	102.56(13)		F2	C7	C6	114.1(3)
O1	C13	C15	106.48(14)		F3	C7	F1	102.8(2)
O1	C13	C14	107.55(14)		F3	C7	C6	113.25(17)
C10	C13	C15	113.87(14)		F6	C7	C6	107.6(4)
C14	C13	C15	110.27(16)		F5	C7	C6	113.0(11)
C14	C13	C10	115.25(15)		F5	C7	F6	93.0(11)
C3	C9	C8	119.97(16)		F4	C7	C6	120.6(5)
O2	C10	C13	102.63(12)		F4	C7	F6	109.0(8)
O2	C10	C11	107.15(15)		F4	C7	F5	110.1(12)
O2	C10	C12	106.58(15)		O1	B1	O2	115.12(16)
C11	C10	C13	114.86(15)		O1	B1	C1	122.19(16)
C12	C10	C13	113.99(16)		O2	B1	C1	122.69(17)
C11	C10	C12	110.71(16)					

12. Crystallographic details

Single-crystals suitable for X-ray diffraction were coated with polyisobutylene or perfluorinated polyether oil, transferred to a nylon loop, and then mounted on the goniometer of a diffractometer equipped with a molybdenum (λ = 0.71073Å) X-ray tube. Diffraction data were collected at 100 K (**2**, **3**, **4**, **6**, **7**, **13**, **15**, **16**, **27**, **29**, **36**, **37**, and **56**). In Olex2,[106] the structures were solved using intrinsic phasing methods and expanded using Fourier techniques.[107,108] All nonhydrogen atoms were refined anisotropically. Hydrogen atoms were included in structure factors calculations. All hydrogen atoms were assigned to idealized geometric positions. CCDC numbers 2483707-2483719 contain the supplementary crystallographic data for this paper: 2483707 (**2**), 2483714 (**3**), 2483709 (**4**), 2483718 (**6**), 2483715 (**7**), 2483719 (**13**), 2483708 (**15**), 2483716 (**16**), 2483717 (**27**), 2483711 (**29**), 2483712 (**36**), 2483710 (**37**), and 2483713 (**56**). These data are provided free of charge by the joint Cambridge Crystallographic Data Centre and Fachinformationszentrum Karlsruhe Access Structures service.

In compounds **2**-**4**, **6**, and **7**, Bi–C–C angles of 164.6-175.5° are observed, showing relevant deviation from linearity of this structural motif. This phenomenon has previously been observed, for instance when alkynyl groups are bound to heavier central atoms including examples such as Fe, Ru, or Sn.[109–111] We suggest that the M–C–C angle is less decisive when a larger atom M with larger and more diffuse atomic orbitals is involved in bond formation. Even at varying angles, the more compact C-centered orbital of the alkynyl group will find sufficient overlap with the large and diffuse M-centered orbital and thus secondary bonding interactions start to also play an important role in determining the M–C–C angle. As an additional aspect, donation of π-electron density to a central atom may come into play, when relevant geometric requirements are met.

**Identification code**	**2**	**3**	**4**
**Empirical formula**	$C_{15}H_{27}BiSi_3$	$C_{15}H_{15}Bi$	$C_{28}H_{23}BiO$
**Formula weight**	500.61	404.25	584.44
**Temperature/K**	100.00	100.00	100.00
**Crystal system**	trigonal	trigonal	monoclinic
**Space group**	R3c	*R*3*m*	$P2_1/c$
**a/Å**	22.3779(16)	16.1111(6)	7.9748(3)
**b/Å**	22.3779(16)	16.1111(6)	18.1019(8)
**c/Å**	7.9427(9)	4.2985(3)	15.6687(6)
**α/°**	90	90	90
**β/°**	90	90	100.9010(10)
**γ/°**	120	120	90
**Volume/Å^3**	3444.6(6)	966.27(10)	2221.10(15)
**Z**	6	3	4
**ρ_{calc}g/cm^3**	1.448	2.084	1.748
**μ/mm^{-1}**	7.824	13.655	7.954
**F(000)**	1452.0	564.0	1128.0
**Crystal size/mm^3**	0.26 × 0.057 × 0.033	0.132 × 0.035 × 0.013	0.507 × 0.087 × 0.065
**Radiation**	MoKα (λ = 0.71073)	MoKα (λ = 0.71073)	MoKα (λ = 0.71073)
**2Θ range for data collection/°**	6.306 to 54.284	5.056 to 60.038	5.202 to 57.456
**Index ranges**	-28 ≤ h ≤ 24, -28 ≤ k ≤ 28, -10 ≤ l ≤ 10	-22 ≤ h ≤ 22, -22 ≤ k ≤ 22, -6 ≤ l ≤ 6	-10 ≤ h ≤ 10, -24 ≤ k ≤ 24, -20 ≤ l ≤ 21
**Reflections collected**	8796	9991	56659
**Independent reflections**	1687 [R_{int} = 0.0695, R_{sigma} = 0.0580]	693 [R_{int} = 0.0362, R_{sigma} = 0.0135]	5748 [R_{int} = 0.0327, R_{sigma} = 0.0167]
**Data / restraints / parameters**	1687/7/62	693/1/32	5748/0/271
**Goodness-of-fit on F^2**	1.044	1.112	1.083
**Final R indexes [I>=2σ (I)]**	R_1 = 0.0314, wR_2 = 0.0668	R_1 = 0.0077, wR_2 = 0.0179	R_1 = 0.0147, wR_2 = 0.0301
**Final R indexes [all data]**	R_1 = 0.0619, wR_2 = 0.0748	R_1 = 0.0077, wR_2 = 0.0179	R_1 = 0.0167, wR_2 = 0.0306
**Largest diff. peak/hole / e Å^{-3}**	1.38/-0.68	0.81/-0.21	0.40/-0.76

Identification code	6	7	13	15
Empirical formula	$C_{32}H_{28}BiCl_3O_2$	$C_{27}H_{21}BiO_3$	$C_{18}H_{14}$	$C_{18}H_{14}O_2$
Formula weight	759.87	602.42	230.29	262.29
Temperature/K	100.00	100.00	100	100
Crystal system	triclinic	monoclinic	triclinic	monoclinic
Space group	$P\bar{1}$	$P2_1/n$	$P\bar{1}$	$P2_1/c$
a/Å	8.1115(12)	13.5893(4)	7.7924(5)	7.0619(8)
b/Å	12.089(3)	7.9050(3)	9.0567(6)	15.6606(17)
c/Å	15.026(4)	20.9549(8)	9.4018(6)	12.3437(14)
α/°	103.619(12)	90	95.597(2)	90
β/°	97.794(13)	91.4330(10)	107.344(2)	90.737(4)
γ/°	93.475(9)	90	97.788(3)	90
Volume/Å^3	1412.2(6)	2250.34(14)	620.80(7)	1365.0(3)
Z	2	4	2	4
ρ_{calc}g/cm^3	1.787	1.778	1.232	1.276
μ/mm^{-1}	6.555	7.860	0.070	0.082
F(000)	740.0	1160.0	244.0	552.0
Crystal size/mm^3	0.102 × 0.057 × 0.048	0.07 × 0.06 × 0.044	0.112 × 0.068 × 0.065	0.254 × 0.18 × 0.084
Radiation	MoKα (λ = 0.71073)	MoKα (λ = 0.71073)	MoKα (λ = 0.71073)	MoKα (λ = 0.71073)
2Θ range for data collection/°	3.906 to 60.088	5.508 to 60.288	4.588 to 50.054	4.202 to 57.434
Index ranges	-11 ≤ h ≤ 11, -17 ≤ k ≤ 17, -21 ≤ l ≤ 20	-17 ≤ h ≤ 19, -11 ≤ k ≤ 11, -29 ≤ l ≤ 29	-9 ≤ h ≤ 8, -10 ≤ k ≤ 10, 0 ≤ l ≤ 11	-9 ≤ h ≤ 8, -20 ≤ k ≤ 21, -16 ≤ l ≤ 16
Reflections collected	48277	60577	2192	29334
Independent reflections	8235 [R_{int} = 0.0500, R_{sigma} = 0.0367]	60577 [R_{int} = 0.0315 R_{sigma} = 0.0427]	2192 [R_{int} = 0.0548, R_{sigma} = 0.0322]	3454 [R_{int} = 0.0656, R_{sigma} = 0.0449]
Data / restraints / parameters	8235/0/298	60577/0/284	2192/12/187	3454/0/184
Goodness-of-fit on F^2	1.041	1.036	1.052	1.048
Final R indexes [I>=2σ (I)]	R_1 = 0.0267, wR_2 = 0.0523	R_1 = 0.0299, wR_2 = 0.0590	R_1 = 0.0581, wR_2 = 0.1542	R_1 = 0.0660, wR_2 = 0.1701
Final R indexes [all data]	R_1 = 0.0321, wR_2 = 0.0540	R_1 = 0.0352, wR_2 = 0.0607	R_1 = 0.1070, wR_2 = 0.1887	R_1 = 0.0985, wR_2 = 0.1937
Largest diff. peak/hole / e Å^{-3}	1.21/-1.23	1.70/-2.52	0.21/-0.18	0.24/-0.36

Identification code	16	27	29
Empirical formula	$C_{24}H_{26}$	$C_{50}H_{80}N_2Si_2$	$C_{64}H_{82}N_2$
Formula weight	314.45	765.34	879.31
Temperature/K	100.00	100.00	100
Crystal system	monoclinic	monoclinic	triclinic
Space group	$P2_1/c$	$C2/c$	$P\bar{1}$
a/Å	12.200(4)	24.663(2)	11.0813(5)
b/Å	9.837(3)	9.6465(8)	13.8443(7)
c/Å	15.844(3)	20.3871(16)	19.7040(10)
α/°	90)	90	78.765(2)
β/°	94.667(13)	99.512(2)	75.396(2)
γ/°	90	90	67.1510(10)
Volume/Å^3	1895.2(9)	4783.6(7)	2679.7(2)
Z	4	4	2
ρ_{calc}g/cm^3	1.102	1.063	1.090
μ/mm^{-1}	0.062	0.10	0.062
F(000)	680.0	1688.0	960.0
Crystal size/mm^3	0.317 × 0.054 × 0.028	0.132 × 0.065 × 0.058	0.255 × 0.174 × 0.064
Radiation	MoKα (λ = 0.71073)	MoKα (λ = 0.71073)	MoKα (λ = 0.71073)
2Θ range for data collection/°	4.878 to 52.11	4.052 to 50.19	4.054 to 57.474
Index ranges	-15 ≤ h ≤ 15, -12 ≤ k ≤ 12, -19 ≤ l ≤ 19	-29 ≤ h ≤ 29, -11 ≤ k ≤ 11, -24 ≤ l ≤ 24	-14 ≤ h ≤ 14, -18 ≤ k ≤ 18, -26 ≤ l ≤ 26
Reflections collected	35264	40306	52924
Independent reflections	3730 [R_{int} = 0.1177, R_{sigma} = 0.0579]	4239 [R_{int} = 0.0694 R_{sigma} = 0.0342]	13114 [R_{int} = 0.0558, R_{sigma} = 0.0552]
Data / restraints / parameters	3730/0/224	4239/0/255	13114/0/613
Goodness-of-fit on F^2	1.121	1.179	1.120
Final R indexes [I>=2σ (I)]	R_1 = 0.0828, wR_2 = 0.1595	R_1 = 0.0639, wR_2 = 0.1399	R_1 = 0.0715, wR_2 = 0.1292
Final R indexes [all data]	R_1 = 0.1223, wR_2 = 0.1747	R_1 = 0.0779, wR_2 = 0.1461	R_1 = 0.1018, wR_2 = 0.1406
Largest diff. peak/hole / e Å^{-3}	0.26/-0.3	0.52/-0.28	0.29/-0.30

**Identification code**	**36**	**37**	**56**
**Empirical formula**	$C_{15}H_{12}Se$	$C_{15}H_9F_3Se$	$C_{15}H_{16}BF_3O_2$
**Formula weight**	271.21	325.18	296.09
**Temperature/K**	100	100.00	100
**Crystal system**	monoclinic	monoclinic	monoclinic
**Space group**	$P2_1$	$P2_1/c$	C2/c
**a/Å**	7.3234(11)	14.6838(5)	18.528(7)
**b/Å**	5.7311(9)	14.7113(5)	6.648(2)
**c/Å**	14.555(2)	5.9011(2)	25.250(9)
**α/°**	90	90	90
**β/°**	101.722(5)	93.1460(10)	107.188(12)
**γ/°**	90	90	90
**Volume/Å^3**	598.17(16)	1272.82(7)	2970.9(18)
**Z**	2	4	8
**ρ_{calc}g/cm^3**	1.506	1.697	1.324
**μ/mm^{-1}**	3.106	2.967	0.110
**F(000)**	272.0	640.0	1232.0
**Crystal size/mm^3**	0.275 × 0.042 × 0.018	0.121 × 0.065 × 0.057	0.203 × 0.094 × 0.07
**Radiation**	MoKα (λ = 0.71073)	MoKα (λ = 0.71073)	MoKα (λ = 0.71073)
**2Θ range for data collection/°**	5.682 to 57.268	3.922 to 57.44	4.602 to 57.418
**Index ranges**	-9 ≤ h ≤ 9, -7 ≤ k ≤ 7, -17 ≤ l ≤ 19	-19 ≤ h ≤ 19, -19 ≤ k ≤ 19, -7 ≤ l ≤ 7	-24 ≤ h ≤ 24, -8 ≤ k ≤ 8, -32 ≤ l ≤ 33
**Reflections collected**	7821	31618	32646
**Independent reflections**	2773 [R_{int} = 0.0440, R_{sigma} = 0.0567]	3280 [R_{int} = 0.0346, R_{sigma} = 0.0186]	3799 [R_{int} = 0.0646, R_{sigma} = 0.0395]
**Data/restraints/parameters**	2773/1/147	3280/0/172	3799/21/222
**Goodness-of-fit on F^2**	1.082	1.107	1.028
**Final R indexes [I>=2σ (I)]**	R_1 = 0.0338, wR_2 = 0.0662	R_1 = 0.0249, wR_2 = 0.0530	R_1 = 0.0556, wR_2 = 0.1224
**Final R indexes [all data]**	R_1 = 0.0405, wR_2 = 0.0682	R_1 = 0.0302, wR_2 = 0.0547	R_1 = 0.0839, wR_2 = 0.1357
**Largest diff. peak/hole / e Å^{-3}**	0.55/-0.57	0.43/-0.29	0.35/-0.23

13. Molecular structures of 13-16, 36, 37, 56

The bond lengths and angles of compounds **13-16**, **36**, **37**, and **56** are listed in the tables above and are unexceptional. Therefore, a detailed discussion is not presented here.

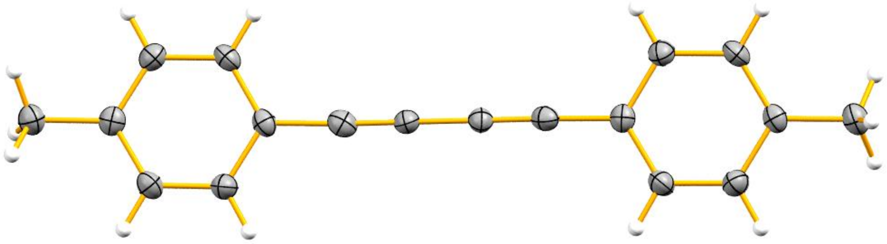

Figure S141. Molecular structure of **13** in the solid state.

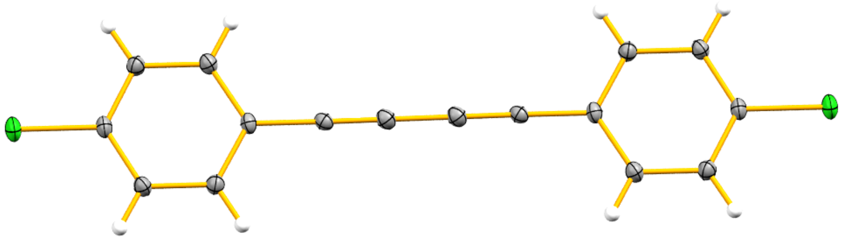

Figure S142. Molecular structure of **14** in the solid state**.**

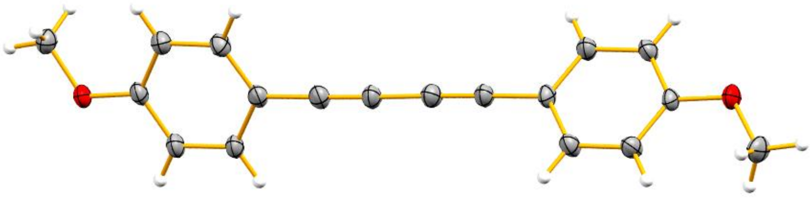

Figure S143. Molecular structure of **15** in the solid state.

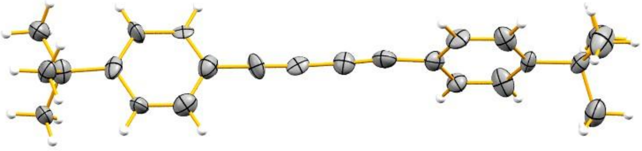

Figure S144. Molecular structure of **16** in the solid state.

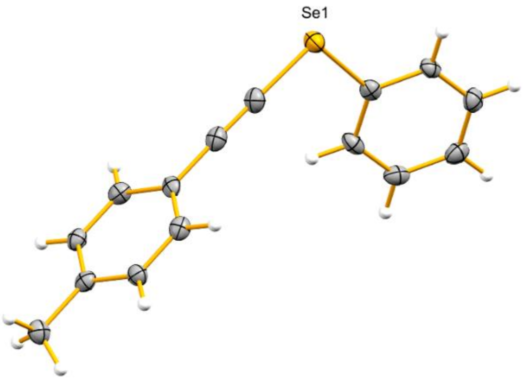

Figure S145. Molecular structure of **36** in the solid state.

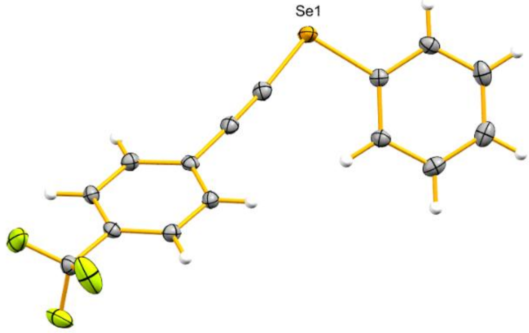

Figure S146. Molecular structure of **37** in the solid state.

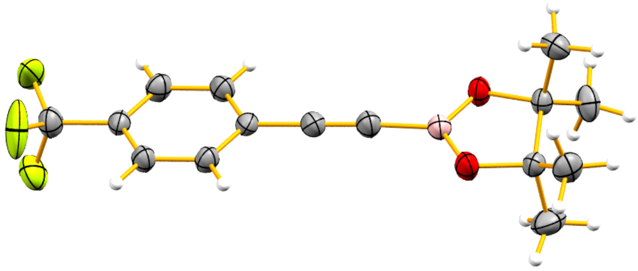

Figure S147. Molecular structure of **56** in the solid state.

14. Computational details

All geometry optimizations and single point energy calculations were performed using the Gaussian16[112] suite of programs at the B3LYP+GD3/def2-TZVP$_{(Benzene,PCM)}$//B3LYP+GD3/def2-SVP level of theory (i.e. geometry optimization at the B3LYP+GD3/def2-SVP level of theory followed by single point calculations at the B3LYP+GD3/def2-TZVP$_{(Benzene,PCM)}$ level of theory).[113–119]

Homolytic Bi–C bond dissociation energies of compounds **2-9** were determined by DFT calculations to give values ranging between 55 and 69 kcal·mol^{-1} (Table S26). A full geometry optimization and frequency analysis at the B3LYP+GD3/def2-TZVP level of theory was exemplarily performed for compounds **4**, **4X**, and **4Y**, yielding a homolytic Bi–C bond dissociation energy deviating by only 0.5 kcal·mol^{-1} from the value obtained using the approach described above, which safes computational resources. These values are expectedly much lower than those of alkynes.[10] They are also lower than the homolytic C–I bond dissociation energy of I–C≡CSiMe$_3$ (77.8 kcal·mol^{-1}), calculated at the same level of theory, underlining the potential of compounds **2-9** to be engaged in selective low-energy radical reaction pathways. The values determined for **2-9** together with the stability trends of **2-9** (*vide supra*) suggest that reactions via free alkynyl radicals are not necessarily the dominating reaction pathways in the transformations of these compounds.

Table S26. **Δ***G* values of homolytic Bi–C bond dissociation reactions of **2-9** as obtained from DFT calculations at the level of theory noted above (also see Table S27).

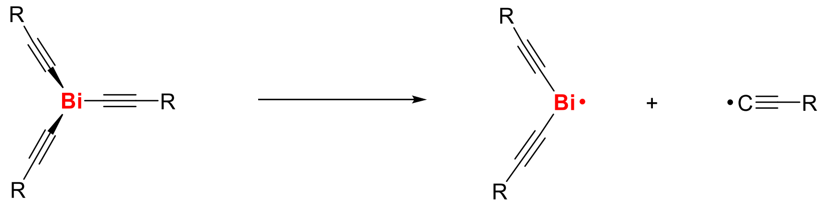

2-9 **2X-9X** **2Y-9Y**

Compound	R	ΔG [kcal/mol]
**2**	SiMe$_3$	68.7
**3**	*cyclo*-C$_3$H$_5$	59.8
**4**	Ph	65.0
**5**	*p*-C$_6$H$_4$-Me	62.6
**6**	*p*-C$_6$H$_4$-Cl	60.3
**7**	*p*-C$_6$H$_4$-OMe	55.0
**8**	*p*-C$_6$H$_4$-*t*Bu	59.6
**9**	*p*-C$_6$H$_4$-CF$_3$	63.4

Table S27. G_{corr} is the thermal correction to Gibbs Free Energy and $G_{298\ gas\ phase}$ is the sum of electronic and thermal free energies at the B3LYP+GD3/def2svp level of theory in gas phase (values in hartree); $E_{0\ benzene}$ is the electronic energy obtained at the B3LYP+GD3/def2tzvp/PCM(solvent=benzene) level of theory (values in hartree); $G_{298\ benzene}$ is the sum of $E_{0\ benzene}$ and G_{corr} (values in hartree).

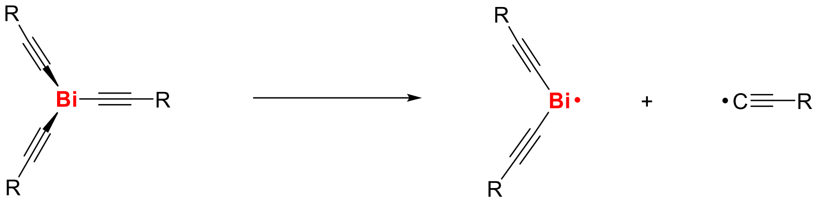

2-9 **2X-9X** **2Y-9Y**

2, **2X**, **2Y**: R = $SiMe_3$
3, **3X**, **3Y**: R = *cyclo*-C_3H_5
4, **4X**, **4Y**: R = Ph
5, **5X**, **5Y**: R = *p*-C_6H_4-CH_3
6, **6X**, **6Y**: R = R = *p*-C_6H_4-Cl
7, **7X**, **7Y**: R = *p*-C_6H_4-OMe
8, **8X**, **8Y**: R = *p*-C_6H_4-*t*Bu
9, **9X**, **9Y**: R = *p*-C_6H_4-CF_3

Compound	G_{corr}	G_{298} gas phase	E_0 benzene	G_{298} benzene
**2**	0.284838	-1670.077222	-1671.36505724	-1671.080219
**2X**	0.181823	-1184.955332	-1185.80805211	-1185.626229
**2Y**	0.082472	-485.015129	-485.426943145	-485.3444711
**3**	0.186262	-794.437169	-795.2825927	-795.0963307
**3X**	0.113794	-601.19803	-601.7533714	-601.6395774
**3Y**	0.049074	-193.14056	-193.4104738	-193.3613998
**4**	0.23332	-1137.228363	-1138.482086	-1138.248766
**4X**	0.148326	-829.723882	-830.554597	-830.406271
**4Y**	0.067698	-307.392038	-307.8066047	-307.7389067
**5**	0.303878	-1255.033423	-1256.485662	-1256.181784
**5X**	0.199534	-908.256567	-909.2237625	-909.0242285
**5Y**	0.091545	-346.672954	-347.1493323	-347.0577873
**6**	0.19847	-2515.667642	-2517.36535	-2517.16688
**6X**	0.124802	-1748.68376	-1749.810176	-1749.685374
**6Y**	0.054252	-766.884647	-767.439607	-767.385355
**7**	0.320787	-1480.469589	-1482.209096	-1481.888309
**7X**	0.206641	-1058.551797	-1059.706281	-1059.49964
**7Y**	0.094728	-421.824804	-422.3957039	-422.3009759
**8**	0.548202	-1608.38046	-1610.459575	-1609.911373
**8X**	0.359124	-1143.824533	-1145.20635	-1144.847226
**8Y**	0.171404	0.171404	-465.1405657	-464.9691617
**9**	0.22819	-2147.609429	-2150.077393	-2149.849203
**9X**	0.144708	-1503.31149	-1504.951601	-1504.806893
**9Y**	0.063995	-644.194971	-645.0053246	-644.9413296
**I**	-0.017503	-297.785973	-297.7790119	-297.7965149
**ICCTMS**	0.081454	-782.923063	-783.3464561	-783.2650021

The B–H and C–H bond dissociation energies of selected compounds relevant to this work were determined by DFT calculations and are summarized in Scheme S10. Compounds **Int-2-TMS** and **Int-2-Ph** are potential reaction intermediates and thus, the energy necessary to generate these species should also be taken into account, when comparing their BDEs with those of HBpin in the context of this work. The reaction of **2** with HBpin to give $[Bi(C{\equiv}CSiMe_3)_2]^{\bullet}$ (**2X**), and **Int-2-TMS** was calculated to be associated with a Gibbs energy of ΔG = +11.7 kcal·mol^{-1}. The reaction of **4** with HBpin to give $[Bi(C{\equiv}CPh)_2]^{\bullet}$ (**4X**), and **Int-2-Ph** was calculated to be associated with a Gibbs energy of ΔG = +3.5 kcal·mol^{-1}. These values would need to be added to the BDEs of **Int-2-TMS** and **Int-2-Ph**, respectively, in order to arrive at values that can better be compared to those calculated for HBpin in the context of this work. It is important to note that in the reactions between alkynyl bismuth compounds **2-9** with HBpin to give the alkynyl boronic esters **49-56**, the BDEs of compounds such as **Int-2-TMS** and **Int-2-Ph** are very unlikely to be the energy barriers that need to be overcome in the course of the reaction, as the calculated BDEs involve the generation of *free* H atoms as high energy species, which can be assumed not to play an important role in these reactions.

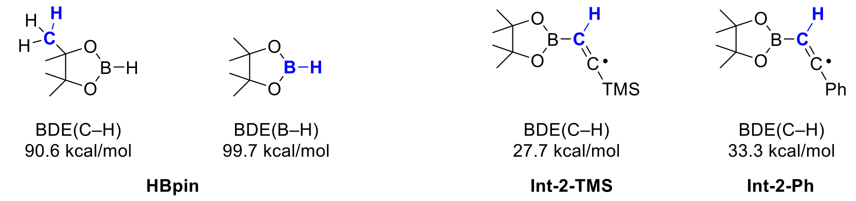

Scheme S10. Homolytic bond dissociation energies (BDEs) for selected bonds (highlighted in blue) of selected species relevant to this work. The values were determined by DFT calculations at the level of theory noted above.

The reaction pathway (thermodynamic values only) calculated for the reaction between HBpin and **2** (Scheme 6d) was also calculated for compound **4** and is presented in Scheme S11.

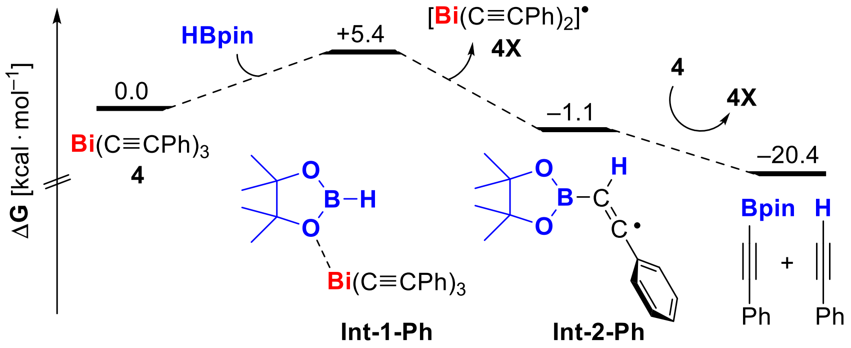

Scheme S11. Thermodynamic reaction profile for the reaction of **4** with HBpin, as determined by DFT calculations.

Reactions of alkynyl bismuth compounds with B_2pin_2 at reaction temperatures of up to 80 °C or under photochemical conditions (blue LED, λ = 460 nm) showed either no conversion or showed the C–C homocoupling product as the main species, with B_2pin_2 remaining unreacted. This is in congruency with the course of the reaction suggested for the substrate HBpin, because the formation of an intermediate analogous to **Int-2-TMS** or **Int-2-Ph** would be more difficult to generate in the case of B_2pin_2 due to the lower mobility of the Bpin group (compared to an H atom).

Table S28. G_{corr} is the thermal correction to Gibbs Free Energy and $G_{298\ gas\ phase}$ is the sum of electronic and thermal free energies at the B3LYP+GD3/def2svp level of theory in gas phase (values in hartree); $E_{0\ benzene}$ is the electronic energy obtained at the B3LYP+GD3/def2tzvp/PCM(solvent=benzene) level of theory (values in hartree); $G_{298\ benzene}$ is the sum of $E_{0\ benzene}$ and G_{corr} (values in hartree).

Compound	G_{corr}	G_{298} gas phase	E_0 benzene	G_{298} benzene
**Int-1-TMS**	0.46494	-2081.512567	-2083.436932	-2082.971992
**Int-2-TMS**	0.264035	-896.547642	-897.5969133	-897.3328783
**Bpin(CCTMS)**	0.256364	-895.987712	-897.0322964	-896.7759324
**HCCTMS**	0.095627	-485.720886	-486.148266	-486.052639
**Int-1-Ph**	0.414576	-1548.661343	-1550.553599	-1550.139023
**Int-2-Ph**	0.245063	-718.940797	-719.9795102	-719.7344472
**Bpin(CCPh)**	0.237506	-718.374399	-719.4060602	-719.1685542
**HCCPh**	0.079346	-308.103256	-308.5202384	-308.4408924
**HBpin**	0.157773	-411.432964	-412.0552785	-411.8975055
**(Bpin)•**	0.146518	-410.762326	-411.372286	-411.225768
**$HB(OCMe_2CMeCH_2O)^•$**	0.141007	-410.776551	-411.3812996	-411.2402926
**H•**	-0.010654	-0.511913	-0.50216632	-0.51282032

Table S29. Cartesian coordinates (Å) of molecular structures obtained from geometry optimizations with DFT calculations as detailed above.

2

Bi	0.00016000	0.00000800	-1.64238500
C	1.53738200	-1.09435400	-0.47118400
Si	3.75568200	-2.67297300	0.96404600
C	2.43227400	-1.73151000	0.07589900
C	4.77132400	-3.63103200	-0.30567900
H	4.13847100	-4.33939600	-0.86436900
H	5.57156800	-4.20527100	0.19083100
H	5.24085300	-2.94848600	-1.03238700
C	4.84713600	-1.43979300	1.88374200
H	5.31659100	-0.73011800	1.18360200
H	5.64876200	-1.96273600	2.43199400
H	4.25620000	-0.85864900	2.60978300
C	2.92983100	-3.85933300	2.17561400
H	2.31591900	-3.30867800	2.90632400
H	3.68667700	-4.43997500	2.72941000
H	2.27243700	-4.56803000	1.64668000
C	-1.71639400	-0.78419800	-0.47152600
Si	-4.19311300	-1.91573900	0.96315600
C	-2.71569500	-1.24060600	0.07546600
C	-5.52957900	-2.31776100	-0.30719800
H	-5.82657000	-1.41605200	-0.86678400
H	-6.42719700	-2.72365500	0.18898100
H	-5.17241600	-3.06604800	-1.03310200
C	-3.67094200	-3.47666200	1.88442700
H	-3.29031200	-4.23846100	1.18513900
H	-4.52481900	-3.90932600	2.43246400
H	-2.87275600	-3.25458200	2.61082100
C	-4.80886100	-0.60664900	2.17331900
H	-4.02561200	-0.34954900	2.90439300
H	-5.69039100	-0.97169000	2.72676900
H	-5.09388600	0.31655400	1.64356000
C	0.17919100	1.87851900	-0.47121200
Si	0.43715700	4.58875000	0.96436900
C	0.28352400	2.97204100	0.07598500
C	0.76958500	5.94599900	-0.30406800
H	1.70183700	5.74927500	-0.85780500
H	0.86696600	6.92603900	0.19262700
H	-0.05226300	6.01346700	-1.03512500
C	-1.18041100	4.92228800	1.87548800
H	-2.02591700	4.97626600	1.17090900
H	-1.12849400	5.87796800	2.42378400

H	-1.39431900	4.12072700	2.60061500
C	1.87070400	4.46289600	2.18357000
H	1.69453600	3.65664300	2.91361900
H	1.99498500	5.40848200	2.73771200
H	2.81529800	4.24497500	1.65970300

2X

Bi	-0.00000300	-1.85947200	-0.00001000
C	-1.62061800	-0.36862700	0.00017500
Si	-3.98781500	1.60333200	0.00004600
C	-2.57920200	0.40373400	0.00018300
C	-5.00546500	1.33608300	-1.56660400
H	-4.39181000	1.49756100	-2.46746500
H	-5.85604100	2.03756700	-1.60059300
H	-5.40586000	0.31024700	-1.60756500
C	-5.04727500	1.28754900	1.52951600
H	-5.44809900	0.26106300	1.52755500
H	-5.89885300	1.98787200	1.56239500
H	-4.45834400	1.42063000	2.45128300
C	-3.28129100	3.35165600	0.03697400
H	-2.66831800	3.50872600	0.93902100
H	-4.09207500	4.09950900	0.03710000
H	-2.64324000	3.53616300	-0.84219500
C	1.62062000	-0.36863700	-0.00008200
Si	3.98783000	1.60330700	-0.00010600
C	2.57922800	0.40369500	-0.00022100
C	5.09998200	1.22584900	-1.47729200
H	5.49947300	0.20047200	-1.41943300
H	5.95289700	1.92460000	-1.50881300
H	4.54391800	1.32100100	-2.42384600
C	3.28432000	3.34815500	-0.13336900
H	2.70365100	3.46786500	-1.06212900
H	4.09514900	4.09595300	-0.13545600
H	2.61580400	3.56791800	0.71449500
C	4.94972300	1.40128200	1.61091300
H	4.30503800	1.59967800	2.48224000
H	5.79940600	2.10382000	1.64565400
H	5.34720700	0.37812200	1.70823900

2Y

C	2.84296500	0.00003200	0.00016900
Si	-0.24387000	-0.00006600	0.00002700
C	1.61694100	-0.00009700	-0.00009100
C	-0.82367700	-1.79148100	0.05654500

H	-0.45697000	-2.35299300	-0.81754500
H	-1.92572000	-1.83897500	0.05774100
H	-0.45747300	-2.29657900	0.96460500
C	-0.82368700	0.94478200	1.52304800
H	-0.45754200	0.46845200	2.44654100
H	-1.92573200	0.97015200	1.56318000
H	-0.45694400	1.98354900	1.50648400
C	-0.82353100	0.84679000	-1.57966700
H	-0.45765100	1.88485900	-1.62817300
H	-1.92556200	0.86861900	-1.62209300
H	-0.45628800	0.31368500	-2.47114300

3

Bi	0.96429100	0.00083100	0.00000000
C	-0.70055900	-1.50143800	2.59960300
C	-0.18237500	-0.94713400	1.64142600
C	-1.33887400	-2.14814500	3.71778300
H	-2.43468000	-2.12933000	3.68564600
C	-0.71448300	-3.38206400	4.35337500
H	-1.39493000	-4.17563300	4.67491800
H	0.22911300	-3.73475500	3.92876800
C	-0.71420400	-2.08423400	5.10417800
H	-1.39445800	-1.96717400	5.95253000
H	0.22958500	-1.54063700	5.19809100
C	-0.70159200	3.00262400	0.00000000
C	-0.18305100	1.89583600	0.00000000
C	-1.33982700	4.29438000	0.00000000
H	-2.43563700	4.25728700	0.00000000
C	-0.71522200	5.46240800	0.74950300
H	-1.39550800	6.13812800	1.27563700
H	0.22832000	5.27138300	1.26751500
C	-0.71522200	5.46240800	-0.74950300
H	-1.39550800	6.13812800	-1.27563700
H	0.22832000	5.27138300	-1.26751500
C	-0.70055900	-1.50143800	-2.59960300
C	-0.18237500	-0.94713400	-1.64142600
C	-1.33887400	-2.14814500	-3.71778300
H	-2.43468000	-2.12933000	-3.68564600
C	-0.71420400	-2.08423400	-5.10417800
H	-1.39445800	-1.96717400	-5.95253000
H	0.22958500	-1.54063700	-5.19809100
C	-0.71448300	-3.38206400	-4.35337500
H	-1.39493000	-4.17563300	-4.67491800
H	0.22911300	-3.73475500	-3.92876800

3X

Bi	-1.17152600	0.00000300	0.00000000
C	1.06468600	0.00003500	2.58902100
C	0.30810200	-0.00010300	1.62449800
C	1.96870700	0.00005800	3.70879200
H	3.03007200	0.00012600	3.43354800
C	1.61491100	0.74926400	4.98711900
H	2.42741400	1.27576200	5.49587600
H	0.65231700	1.26697100	5.00711800
C	1.61500900	-0.74927900	4.98707400
H	2.42757300	-1.27571700	5.49579200
H	0.65247900	-1.26710900	5.00700700
C	1.06468600	0.00003500	-2.58902100
C	0.30810200	-0.00010300	-1.62449800
C	1.96870700	0.00005800	-3.70879200
H	3.03007200	0.00012600	-3.43354800
C	1.61500900	-0.74927900	-4.98707400
H	2.42757300	-1.27571700	-5.49579200
H	0.65247900	-1.26710900	-5.00700700
C	1.61491100	0.74926400	-4.98711900
H	2.42741400	1.27576200	-5.49587600
H	0.65231700	1.26697100	-5.00711800

3Y

C	1.19127400	0.12535500	0.13241900
C	2.38425000	-0.08178000	-0.27765600
C	-0.14294000	0.07929300	0.59371200
H	-0.30519800	0.16718700	1.67426500
C	-1.25908400	0.68230500	-0.30351700
H	-2.03304000	1.22897900	0.24211000
H	-0.92650000	1.14626900	-1.23478400
C	-1.18075300	-0.78379500	-0.16968800
H	-1.90101400	-1.30000500	0.47089100
H	-0.79073200	-1.37069900	-1.00410900

4

Bi	0.00057400	0.00027500	-1.75823800
C	-1.20310400	1.45393900	-0.59374800
C	-0.65699900	-1.76888700	-0.59389400
C	-1.88345100	2.31666300	-0.05526400
C	2.94849600	0.47274300	-0.05481800
C	-2.66658000	3.31770500	0.60202400
C	4.20719300	0.64973500	0.60226400
C	1.86107400	0.31541600	-0.59323500

C	-1.06468700	-2.78924800	-0.05557300
C	-4.20137400	5.28715900	1.90018900
H	-4.79750900	6.05189200	2.40448800
C	-3.95037800	3.65691100	0.12480800
H	-4.34342600	3.14455500	-0.75584000
C	-1.19182400	-5.24930100	0.12596500
H	-0.54989900	-5.33404800	-0.75340300
C	-2.92902100	4.95836200	2.38119300
H	-2.52924100	5.46608500	3.26269200
C	5.14004700	1.59703700	0.12968400
H	4.89050100	2.19818300	-0.74717800
C	-1.54104800	-3.96762400	0.60162900
C	-4.70810300	4.63326600	0.77163600
H	-5.70160000	4.88624000	0.39262800
C	-2.36994100	-3.86491000	1.73925300
H	-2.64147400	-2.87468800	2.11083000
C	6.36469900	1.76425100	0.77627200
H	7.07832400	2.50216400	0.40090000
C	-2.16490700	3.98254800	1.74136000
H	-1.17221900	3.72194500	2.11417900
C	-2.83406000	-5.01417600	2.37896300
H	-3.47543100	-4.92130500	3.25911500
C	-1.65960100	-6.39331000	0.77269100
H	-1.38103700	-7.38041100	0.39491600
C	4.53520000	-0.12311200	1.73667900
H	3.81536100	-0.85649500	2.10589400
C	-2.48148000	-6.28075200	1.89954900
H	-2.84652900	-7.17908400	2.40378100
C	6.68069200	0.99252900	1.89991700
H	7.64121900	1.12580800	2.40403400
C	5.76258200	0.04991500	2.37625800
H	6.00479600	-0.55483200	3.25390300

4X

Bi	0.00001100	-2.02143000	-0.00001700
C	1.61678200	-0.53695700	0.00004800
C	2.57386700	0.23237200	0.00006400
C	-2.57392400	0.23230600	0.00000300
C	3.67073000	1.14502400	0.00003400
C	-3.67076900	1.14497700	0.00000700
C	-1.61691500	-0.53711900	0.00000200
C	5.83094900	2.95100100	0.00000100
H	6.66943000	3.65197900	-0.00001500
C	5.00236000	0.67356700	0.00000300
H	5.18508400	-0.40303900	-0.00000800

C	4.51548600	3.42949300	0.00003300
H	4.32512600	4.50579300	0.00004500
C	-5.00241200	0.67358200	0.00013800
H	-5.18519600	-0.40301500	0.00024400
C	6.06887700	1.57169200	-0.00001300
H	7.09440900	1.19361500	-0.00003700
C	-6.06889300	1.57176000	0.00013600
H	-7.09444200	1.19371700	0.00024300
C	3.44247700	2.53931800	0.00005400
H	2.41485300	2.90844700	0.00008700
C	-3.44245200	2.53927400	-0.00014000
H	-2.41480500	2.90834600	-0.00025000
C	-5.83090700	2.95105300	0.00000000
H	-6.66935300	3.65207200	-0.00000300
C	-4.51541500	3.42949300	-0.00014100
H	-4.32501700	4.50578900	-0.00025300

4Y

C	-3.32730500	-0.00007500	0.00001700
C	-2.10908800	0.00009900	0.00000600
C	-0.67401800	-0.00002100	-0.00011500
C	2.13565300	0.00002200	-0.00003400
H	3.22848600	0.00004200	-0.00000300
C	0.03944000	-1.21604800	0.00001300
H	-0.51144200	-2.15871900	0.00000000
C	1.43442100	1.21074600	0.00001500
H	1.97837400	2.15860600	-0.00007200
C	1.43444400	-1.21075600	0.00002700
H	1.97843500	-2.15859300	0.00013700
C	0.03937500	1.21601700	0.00002800
H	-0.51138400	2.15875600	0.00019800

5

Bi	0.00005400	0.00005500	-1.99466900
C	0.10040100	-1.88565300	-0.83287200
C	-1.68342300	0.85616900	-0.83333300
C	1.58309300	1.03000900	-0.83323500
C	-2.65173400	1.37925600	-0.29811300
C	-0.55128500	-5.71873300	2.16804400
H	-1.13561500	-5.86759000	3.08080600
C	0.93886600	-5.31770100	-0.15302200
H	1.52678000	-5.16026500	-1.05981900
C	0.13128900	-2.98570300	-0.29748500
C	2.52038200	1.60683900	-0.29801400

C	4.13753800	3.47112300	-0.15496000
H	3.70814200	3.90074900	-1.06256700
C	-0.58478300	-4.47969200	1.53249000
H	-1.18739800	-3.66598900	1.94144800
C	0.21970800	-6.77924400	1.66268400
C	3.60662800	2.26900600	0.35540400
C	0.16167200	-4.25739700	0.35614000
C	5.76189300	3.58010400	1.66153500
C	-3.76841200	1.98857500	0.35542500
C	5.19421100	4.11011500	0.49162000
H	5.58728700	5.04404900	0.07957600
C	0.96401700	-6.55216100	0.49374600
H	1.57754000	-7.35910000	0.08268200
C	4.17127100	1.73471900	1.53278500
H	3.76691400	0.80681300	1.94265200
C	5.22769700	2.38318200	2.16810600
H	5.64793000	1.95238300	3.08165000
C	-4.67883000	3.33018800	2.17146600
H	-4.51655000	3.90570200	3.08753800
C	-5.98173300	3.19882500	1.66180600
C	-3.58890600	2.73984600	1.53603400
H	-2.58373600	2.85010800	1.94831900
C	-5.07420000	1.85179600	-0.15807100
H	-5.23089200	1.26890900	-1.06823500
C	-6.15603700	2.44701200	0.48864300
H	-7.16081100	2.32399200	0.07413000
C	-7.14961500	3.87090600	2.33975800
H	-7.02720100	3.88744900	3.43404700
H	-7.24627700	4.91996900	2.00823200
H	-8.09985400	3.36593100	2.10832800
C	6.92808300	4.25522500	2.33938100
H	6.88216200	4.13943400	3.43356400
H	7.88488800	3.81532800	2.00652600
H	6.96509900	5.33097100	2.10939200
C	0.22150100	-8.12686100	2.34031600
H	0.13427100	-8.02989500	3.43378500
H	-0.63206500	-8.73941700	1.99973000
H	1.13916500	-8.69258000	2.11805000

5X

Bi	-0.00000900	2.33838300	0.00024800
C	1.61755700	0.85581500	0.00032500
C	-1.61746100	0.85568200	-0.00036600
C	2.57642900	0.08828100	0.00044400
C	-2.57624000	0.08803200	-0.00077700

C	-5.00558900	-0.35710500	-0.00279000
H	-5.19425200	0.71853800	-0.00360400
C	-3.67384900	-0.82232900	-0.00167800
C	-5.85584500	-2.64319500	-0.00542400
C	3.67413700	-0.82196000	0.00090700
C	-6.07113500	-1.25604500	-0.00501600
H	-7.09512200	-0.87183800	-0.00736800
C	-3.45361700	-2.21832200	-0.00388500
H	-2.42814800	-2.59368600	-0.00557200
C	-4.52690000	-3.10370200	-0.00623400
H	-4.33307500	-4.18044000	-0.00984800
C	4.52705300	-3.10335000	0.00267900
H	4.33337800	-4.18014600	0.00408400
C	5.85613600	-2.64275400	0.00280300
C	3.45387900	-2.21811700	0.00148600
H	2.42838100	-2.59341600	0.00190900
C	5.00572300	-0.35676400	0.00198900
H	5.19444400	0.71886500	0.00279300
C	6.07142300	-1.25579700	0.00310600
H	7.09538400	-0.87158500	0.00467800
C	7.00415900	-3.62092300	-0.00552400
H	6.92118300	-4.34017000	0.82598400
H	7.01587700	-4.20985500	-0.93845400
H	7.97517700	-3.11178700	0.08191100
C	-7.00499500	-3.61992200	0.01138500
H	-6.87639400	-4.40259100	-0.75381300
H	-7.07325100	-4.13246400	0.98648800
H	-7.96798600	-3.12033900	-0.17033800

5Y

C	3.86379900	-0.00038700	0.10490400
C	2.59427600	0.00022100	-0.05403200
C	1.19444400	0.00014500	-0.03062600
C	-0.92529800	1.21331600	-0.01427500
H	-1.47237900	2.15989400	-0.01312600
C	-1.64668900	0.00002800	-0.00727500
C	0.46023300	1.22258300	-0.02350300
H	1.01363000	2.16360800	-0.02865300
C	0.46032900	-1.22237200	-0.02351000
H	1.01381100	-2.16334700	-0.02869300
C	-0.92517800	-1.21322100	-0.01430500
H	-1.47219400	-2.15984200	-0.01319000
C	-3.14942900	-0.00018600	0.04167600
H	-3.57240300	-0.89288400	-0.44307200
H	-3.49675100	-0.00320400	1.09058600

H	-3.57263200	0.89501400	-0.43818000

6

Bi	0.00057800	-0.00034500	-2.23541200
Cl	-5.38347700	-6.26854600	2.28485100
Cl	-2.73991500	7.79516900	2.28467200
Cl	8.12143600	-1.52539900	2.28527200
C	-1.19444400	-1.45783300	-1.06523100
C	-0.66471700	1.76330000	-1.06554200
C	1.86016300	-0.30604100	-1.06489300
C	-1.02542400	2.79649100	-0.51834900
C	-4.27011600	-3.80185400	1.96238600
H	-4.82721400	-3.52043700	2.85737600
C	-2.85523800	-4.56124700	-0.33201800
H	-2.30219400	-4.85527200	-1.22630000
C	-1.90925200	-2.28649700	-0.51806900
C	2.93523800	-0.51042700	-0.51766500
C	5.37790600	-0.19044400	-0.33116000
H	5.35556700	0.43600600	-1.22508800
C	-3.45965300	-2.87741600	1.30711200
H	-3.37688100	-1.85822300	1.68963600
C	-4.36852500	-5.10622500	1.46533600
C	4.17627900	-0.75150100	0.14992100
C	-2.73901500	-3.24036100	0.14947900
C	6.60719000	-1.22840400	1.46577900
C	-1.43728200	3.99177600	0.14922000
C	6.58687700	-0.42485400	0.32050600
H	7.51572900	0.01017000	-0.05169500
C	-3.66326500	-5.49058800	0.31964300
H	-3.75194400	-6.51230900	-0.05287400
C	4.22284000	-1.55761400	1.30713200
H	3.29914500	-1.99651100	1.68934300
C	5.42879600	-1.79662100	1.96239600
H	5.46405900	-2.42022700	2.85706500
C	-1.15964400	5.59807400	1.96274600
H	-0.63810100	5.93969900	2.85820500
C	-2.23987100	6.33537100	1.46512600
C	-0.76356800	4.43422900	1.30744900
H	0.07756800	3.85305100	1.69040400
C	-2.52288700	4.75279100	-0.33288300
H	-3.05329100	4.42099900	-1.22766400
C	-2.92444700	5.91695200	0.31881500
H	-3.76481100	6.50452200	-0.05414000

6X

Bi	-0.00002100	-2.62213900	-0.00001400
Cl	-7.14689400	3.48470200	-0.00015400
Cl	7.14689300	3.48471200	-0.00009400
C	-1.61469700	-1.13415300	0.00022600
C	1.61472200	-1.13421000	0.00012600
C	-2.56945200	-0.36197500	0.00011200
C	2.56950400	-0.36205200	0.00012600
C	4.99665700	0.08941300	-0.00033700
H	5.18850400	-0.98538400	-0.00067800
C	3.66252300	0.55312600	0.00010800
C	5.81026300	2.36018100	-0.00004800
C	-3.66247800	0.55317700	0.00009600
C	6.06415900	0.98374200	-0.00043900
H	7.09429400	0.62398600	-0.00081400
C	3.43364900	1.94715200	0.00053400
H	2.40703800	2.31851400	0.00086600
C	4.49739200	2.84571700	0.00043600
H	4.31913500	3.92221400	0.00072500
C	-4.49738100	2.84575000	-0.00002900
H	-4.31914100	3.92225000	-0.00006800
C	-5.81024700	2.36019100	-0.00003900
C	-3.43362100	1.94720500	0.00000600
H	-2.40701700	2.31859100	0.00002300
C	-4.99659700	0.08943700	0.00002000
H	-5.18843700	-0.98536200	0.00004300
C	-6.06411900	0.98375100	-0.00001600
H	-7.09424600	0.62397100	-0.00003800

6Y

C	3.86379900	-0.00038700	0.10490400
C	2.59427600	0.00022100	-0.05403200
C	1.19444400	0.00014500	-0.03062600
C	-0.92529800	1.21331600	-0.01427500
H	-1.47237900	2.15989400	-0.01312600
C	-1.64668900	0.00002800	-0.00727500
C	0.46023300	1.22258300	-0.02350300
H	1.01363000	2.16360800	-0.02865300
C	0.46032900	-1.22237200	-0.02351000
H	1.01381100	-2.16334700	-0.02869300
C	-0.92517800	-1.21322100	-0.01430500
H	-1.47219400	-2.15984200	-0.01319000
C	-3.14942900	-0.00018600	0.04167600
H	-3.57240300	-0.89288400	-0.44307200

H	-3.49675100	-0.00320400	1.09058600
H	-3.57263200	0.89501400	-0.43818000

7

Bi	-0.05121200	-0.03423000	2.18272000
O	-7.89007800	-0.47043100	-2.30492800
O	3.36697300	7.37906300	-1.70562000
C	0.73411900	1.73128600	1.09778300
O	4.50145400	-6.44784200	-2.28289900
C	-1.90756300	-0.19639600	0.98291200
C	1.08271500	-1.52477900	0.99622600
C	1.71796800	-2.41479900	0.44584100
C	1.21145900	2.74388500	0.60244800
C	-2.99697300	-0.26501200	0.42882900
C	1.76512100	3.91316400	-0.00477200
C	2.44686000	-3.44352200	-0.22729000
C	3.88573500	7.40732500	-3.01715600
H	4.23241900	8.43432300	-3.19493900
H	3.11663800	7.15422200	-3.76991100
H	4.73987000	6.71577200	-3.13736700
C	2.36239700	6.27424400	0.10000900
H	2.41124000	7.22635900	0.63196300
C	1.82450100	5.14146300	0.69374600
H	1.43894200	5.19295500	1.71414200
C	3.62302900	-3.98382900	0.32474300
H	3.98188400	-3.60686600	1.28486200
C	4.33911400	-4.98794200	-0.32999700
H	5.24610500	-5.37887700	0.13093900
C	-4.25381100	-0.33686400	-0.24780800
C	-8.95749600	-1.29893900	-1.90041200
H	-9.77347000	-1.13092000	-2.61629600
H	-9.31459700	-1.04487000	-0.88537800
H	-8.67918100	-2.36874300	-1.92026200
C	5.68641400	-7.02935000	-1.78579700
H	6.49336000	-6.28298700	-1.66694100
H	6.00173400	-7.77869800	-2.52443900
H	5.52303500	-7.53163600	-0.81451400
C	2.86208900	6.22133200	-1.21458100
C	2.81141800	5.00922900	-1.92283300
H	3.19038700	4.93687900	-2.94217800
C	3.88451700	-5.47753000	-1.56551600
C	2.26800700	3.87399600	-1.31888900
H	2.23032800	2.93404100	-1.87342700
C	-5.28019400	-1.18389500	0.20946200
H	-5.10865100	-1.79597800	1.09750700

C	-6.73719800	-0.47492200	-1.59253700
C	-6.50909300	-1.25838100	-0.44928000
H	-7.27761400	-1.92808000	-0.06354300
C	-4.50035800	0.44475300	-1.40092600
H	-3.71555600	1.10766700	-1.77093900
C	2.00364100	-3.94791100	-1.47252400
H	1.09316100	-3.54053800	-1.91677800
C	2.70901900	-4.94626200	-2.12846200
H	2.37379100	-5.34115300	-3.08950100
C	-5.71916500	0.37713300	-2.06002800
H	-5.91767500	0.97702500	-2.95032200

7X

Bi	-0.00000200	-2.67075300	0.00003600
O	6.98233000	3.04439300	-0.00005600
C	1.61868400	-1.19014100	-0.00009200
O	-6.98228900	3.04445600	-0.00014200
C	-1.61870000	-1.19015400	0.00013000
C	-2.58177000	-0.42727700	0.00013000
C	2.58170500	-0.42720200	-0.00010800
C	3.68530200	0.47411100	-0.00009800
C	-3.68534000	0.47407000	0.00008000
C	6.84763600	4.44889800	0.00015200
H	7.86569500	4.86112800	0.00014900
H	6.31500800	4.81113200	0.89858600
H	6.31490000	4.81138600	-0.89811600
C	6.09042000	0.87791900	-0.00021800
H	7.12130000	0.51853500	-0.00032500
C	5.01884200	-0.00215800	-0.00023500
H	5.19875600	-1.07919700	-0.00035600
C	-3.48170200	1.86856700	0.00028600
H	-2.46029400	2.25474400	0.00049900
C	-4.55527400	2.75980400	0.00021800
H	-4.35475300	3.83112600	0.00038500
C	-6.84755500	4.44895900	-0.00006100
H	-6.31503100	4.81126700	0.89840600
H	-7.86560100	4.86121700	-0.00023500
H	-6.31469200	4.81133700	-0.89829700
C	5.87199100	2.26880700	-0.00006100
C	4.55530700	2.75981800	0.00007500
H	4.35482500	3.83114700	0.00019400
C	-5.87197200	2.26883700	-0.00006000
C	3.48170800	1.86861700	0.00005600
H	2.46031200	2.25482500	0.00016300
C	-5.01889700	-0.00215600	-0.00019400

H	-5.19884400	-1.07918900	-0.00035700
C	-6.09044500	0.87795600	-0.00026400
H	-7.12133800	0.51860800	-0.00048000

7Y

O	2.50938000	0.51534700	0.00575300
C	-4.27847000	-0.33106900	0.03723000
C	-3.00675200	-0.18696100	-0.02148700
C	-1.62023600	-0.03228100	-0.01196200
C	-0.75427600	-1.16589800	-0.00956800
H	-1.20388000	-2.16071500	-0.01232600
C	0.62390700	-1.02535800	-0.00409800
H	1.25455500	-1.91401200	-0.00284900
C	3.44029000	-0.55560400	0.00761100
H	3.32751000	-1.18585800	0.90648300
H	4.43680200	-0.09622400	0.01176200
H	3.33388900	-1.18345500	-0.89372000
C	1.19317300	0.26852500	0.00026400
C	-1.01590100	1.26540700	-0.00609700
H	-1.66670200	2.14172900	-0.00630600
C	0.35397200	1.41032400	-0.00064000
H	0.82854400	2.39325400	0.00341100

8

Bi	-0.00078000	0.00067700	-2.58107500
C	1.16343400	1.48727200	-1.41903500
C	0.70513000	-1.75099400	-1.41959500
C	-1.87025400	0.26505600	-1.41881000
C	1.09161500	-2.78108600	-0.88370000
C	4.17942200	3.94506200	1.57791100
H	4.71787500	3.65385200	2.48258900
C	2.78524900	4.62477000	-0.72692200
H	2.23903000	4.89853200	-1.63220800
C	1.86271400	2.33653200	-0.88293300
C	-2.95540500	0.44537100	-0.88254700
C	-5.39878700	0.10234700	-0.72897200
H	-5.36290900	-0.50471800	-1.63618100
C	3.39096800	2.99018900	0.94430800
H	3.31916100	1.97887000	1.35001400
C	4.29663700	5.26050000	1.08668500
C	-4.20816100	0.65913800	-0.22705700
C	2.67476900	3.31411100	-0.22744200
C	-6.70498200	1.08896600	1.08706900
C	1.53294000	-3.97296700	-0.22840300

C	-6.61711900	0.31536000	-0.08172900
H	-7.50988800	-0.13912300	-0.51098100
C	3.57950300	5.57286300	-0.07968900
H	3.63016800	6.57420400	-0.50700100
C	-4.28555400	1.43769900	0.94713000
H	-3.37358200	1.87893200	1.35473400
C	-5.50685900	1.64234900	1.58071100
H	-5.52375900	2.25142400	2.48730900
C	1.33160400	-5.58899000	1.57972100
H	0.81314900	-5.90785900	2.48675200
C	2.40927600	-6.35033600	1.08535700
C	0.89811900	-4.42890300	0.94632700
H	0.06052100	-3.85938400	1.35449300
C	2.60979100	-4.72613100	-0.73105100
H	3.11700200	-4.39182700	-1.63869700
C	3.03457000	-5.88780600	-0.08398000
H	3.87402300	-6.43407400	-0.51381300
C	2.84986000	-7.62564400	1.82501100
C	-8.02960000	1.34514900	1.82691500
C	5.18159800	6.27897500	1.82639500
C	1.66958100	-8.62289100	1.87126100
H	1.34867900	-8.89844300	0.85415400
H	1.96580700	-9.54430700	2.39922000
H	0.79800900	-8.20340200	2.39608500
C	4.03867700	-8.31879000	1.13654700
H	3.79211400	-8.62997000	0.10932800
H	4.92507200	-7.66655000	1.09572300
H	4.31893500	-9.22359400	1.69838600
C	3.27223700	-7.25992400	3.26638800
H	4.11248800	-6.54765700	3.26018900
H	2.44732200	-6.80086600	3.83191900
H	3.59104800	-8.16226500	3.81378500
C	-7.92351500	0.79740300	3.26859700
H	-7.72653300	-0.28636400	3.26299800
H	-7.11344800	1.28281900	3.83358800
H	-8.86425200	0.97257400	3.81616500
C	-9.22429100	0.66143900	1.13918500
H	-9.37083700	1.02987100	0.11176800
H	-9.10237700	-0.43231800	1.09902400
H	-10.14791600	0.87126300	1.70111700
C	-8.30349800	2.86587700	1.87233300
H	-8.38204700	3.28094700	0.85500300
H	-9.24950100	3.07010500	2.40041000
H	-7.50443700	3.41145700	2.39663800
C	5.18343700	7.65694600	1.14161900

H	5.57304900	7.60229000	0.11301900
H	4.17466200	8.09720200	1.10514600
H	5.82771800	8.35142500	1.70329400
C	6.63625000	5.75738400	1.86682300
H	7.03243500	5.62054400	0.84813500
H	7.28670200	6.47413000	2.39472800
H	6.71169600	4.79153300	2.38883200
C	4.65781300	6.45726900	3.26985200
H	3.62030600	6.82735000	3.26779700
H	4.67577000	5.51184100	3.83278200
H	5.28048500	7.18394600	3.81732100

8X

Bi	0.00000100	3.11882300	0.00000300
C	1.61744300	1.63616500	-0.00001400
C	-1.61744100	1.63616500	-0.00001600
C	2.57590600	0.86816000	-0.00002400
C	-2.57590400	0.86816100	-0.00002200
C	-5.00431400	0.41571200	-0.00004400
H	-5.19746400	1.49063400	-0.00008000
C	-3.67251500	-0.04316000	-0.00001000
C	-5.86489700	-1.87286000	-0.00000200
C	3.67251700	-0.04316000	-0.00002000
C	-6.07134300	-0.48327700	-0.00004500
H	-7.08228200	-0.07607100	-0.00008300
C	-3.45520800	-1.43888800	0.00004400
H	-2.43104200	-1.81776500	0.00008500
C	-4.52889900	-2.32218800	0.00004300
H	-4.31746600	-3.39384500	0.00009500
C	4.52889400	-2.32219100	-0.00011100
H	4.31746000	-3.39384800	-0.00019200
C	5.86489500	-1.87286500	-0.00000700
C	3.45520500	-1.43888900	-0.00011800
H	2.43103800	-1.81776300	-0.00020200
C	5.00431500	0.41570700	0.00007500
H	5.19747100	1.49062900	0.00014700
C	6.07134400	-0.48328400	0.00008200
H	7.08228500	-0.07608100	0.00016000
C	7.01654200	-2.89288500	0.00000700
C	-7.01654500	-2.89288100	0.00000800
C	6.91349900	-3.77941600	-1.26230100
H	6.98286900	-3.16947100	-2.17691400
H	7.73116700	-4.51870800	-1.27984200
H	5.96293700	-4.33271800	-1.29906900
C	6.91336500	-3.77951700	1.26223000

H	6.98267700	-3.16965600	2.17690500
H	5.96278500	-4.33279600	1.29887100
H	7.73100700	-4.51883600	1.27978100
C	8.39762500	-2.21441600	0.00010600
H	8.54503000	-1.58604000	-0.89219100
H	8.54494500	-1.58611200	0.89247000
H	9.18900600	-2.98026600	0.00011600
C	-6.91338300	-3.77955200	-1.26218400
H	-6.98275800	-3.16973600	-2.17688600
H	-5.96278700	-4.33280300	-1.29884500
H	-7.73099400	-4.51890600	-1.27967500
C	-6.91348300	-3.77937400	1.26234700
H	-7.73114900	-4.51866900	1.27992300
H	-5.96291200	-4.33266300	1.29910900
H	-6.98283800	-3.16939900	2.17693900
C	-8.39763000	-2.21442200	-0.00010200
H	-8.54504400	-1.58603700	0.89218500
H	-8.54495400	-1.58613300	-0.89247800
H	-9.18900400	-2.98028000	-0.00010000

8Y

C	4.94299300	-0.01487700	0.10064200
C	3.67320800	-0.00510000	-0.05569100
C	2.27349300	0.00590000	-0.03160100
C	0.15899400	1.23489300	-0.01097600
H	-0.35392700	2.19587100	-0.00539500
C	-0.58195400	0.03420400	-0.00528900
C	1.54709400	1.22955100	-0.02233300
H	2.10523800	2.16788100	-0.02493100
C	1.52503500	-1.20785500	-0.02274300
H	2.06643200	-2.15585900	-0.02581700
C	0.14206200	-1.18310300	-0.01128900
H	-0.39797400	-2.13173800	-0.00587500
C	-2.11623000	0.00417300	0.00844200
C	-2.73171700	1.41436700	0.01365400
H	-2.43308400	1.98938800	0.90394600
H	-2.44848100	1.98924600	-0.88175900
H	-3.82983200	1.33847200	0.02314700
C	-2.59502200	-0.74119800	1.27689600
H	-2.24469900	-0.23256300	2.18875900
H	-3.69615100	-0.77135800	1.30307200
H	-2.23268000	-1.77957400	1.30855600
C	-2.61743600	-0.74169600	-1.25106200
H	-2.28341700	-0.23338000	-2.16921900
H	-2.25568400	-1.78007900	-1.28868700

H	-3.71886000	-0.77185400	-1.25770600

9

Bi	-0.00042300	-0.00045200	2.58426900
C	0.52843900	1.80605600	1.40794700
C	-1.82921700	-0.44569500	1.40776800
C	1.29972900	-1.36155000	1.40787700
C	-2.88388800	-0.72681900	0.85565200
C	0.66879800	5.71184500	-1.55986700
H	0.06715700	6.04774400	-2.40634700
C	2.23915200	4.85760600	0.59368900
H	2.85129100	4.51757500	1.43104200
C	0.81232100	2.86000800	0.85586800
C	2.07084600	-2.13407200	0.85580500
C	3.08610300	-4.36890000	0.59141300
H	2.48393600	-4.73000900	1.42717000
C	0.35486600	4.52398300	-0.90461500
H	-0.49645300	3.92365500	-1.23059800
C	1.76507300	6.47516700	-1.14097900
C	2.96659100	-3.02351500	0.18396300
C	1.13529700	4.08036300	0.18410800
C	4.72651400	-4.76533500	-1.14128400
C	-4.10218800	-1.05725200	0.18378900
C	3.95872600	-5.23119100	-0.06747900
H	4.04213900	-6.27348800	0.24551700
C	2.55024800	6.04434000	-0.06512400
H	3.41003300	6.63853300	0.24953700
C	3.74309700	-2.56814900	-0.90271300
H	3.65016200	-1.53008800	-1.22705000
C	4.61526400	-3.43346700	-1.55805400
H	5.20861900	-3.07935500	-2.40295900
C	-5.28203900	-2.27866100	-1.55888600
H	-5.27229800	-2.96898800	-2.40427500
C	-6.49093500	-1.70916000	-1.14152800
C	-4.09640600	-1.95665400	-0.90351500
H	-3.15107600	-2.39512400	-1.22831900
C	-5.32692400	-0.48815000	0.59183500
H	-5.33831700	0.21332500	1.42807600
C	-6.51016200	-0.81205700	-0.06709100
H	-7.45439600	-0.36320200	0.24637100
C	-7.78116900	-2.10387400	-1.81523100
C	5.71397200	-5.68486400	-1.81491000
C	2.06826200	7.78994700	-1.81463500
F	-8.69106500	-1.11420300	-1.77096600
F	-8.33876300	-3.17663900	-1.21953800

F	-7.59206200	-2.42699300	-3.10694800
F	5.89988600	-5.35887300	-3.10636800
F	5.31202400	-6.96778000	-1.77148200
F	6.92148300	-5.63146700	-1.21854300
F	3.37995500	8.08429100	-1.76836200
F	1.69595000	7.78696700	-3.10694900
F	1.41615200	8.80883500	-1.22032600

9X

Bi	-0.00006600	3.09656200	0.00009400
C	1.61359100	1.60561100	0.01059400
C	-1.61343900	1.60530300	-0.01116500
C	2.56678000	0.83206300	0.01669500
C	-2.56649300	0.83159100	-0.01762400
C	-4.99207400	0.38034600	-0.03589600
H	-5.18215200	1.45518200	-0.04201400
C	-3.65863700	-0.08527500	-0.02431000
C	-5.80706000	-1.89720600	-0.03885800
C	3.65884100	-0.08490000	0.02372700
C	-6.05446500	-0.51896900	-0.04320700
H	-7.08304100	-0.15413500	-0.05974000
C	-3.42453500	-1.47843400	-0.02366800
H	-2.39673000	-1.84572900	-0.02029400
C	-4.49021000	-2.37364400	-0.03098500
H	-4.30418200	-3.44915100	-0.03801200
C	4.49033200	-2.37329100	0.03041000
H	4.30420000	-3.44878400	0.03718900
C	5.80721900	-1.89691800	0.03897100
C	3.42468500	-1.47804800	0.02271500
H	2.39687800	-1.84532700	0.01879500
C	4.99229200	0.38065400	0.03592900
H	5.18238400	1.45548700	0.04228900
C	6.05465400	-0.51870100	0.04358100
H	7.08323800	-0.15393000	0.06053000
C	6.95915900	-2.86845800	-0.01208100
C	-6.95922400	-2.86839400	0.01237300
F	8.05644600	-2.37501300	0.58961800
F	6.65608900	-4.03490700	0.58525600
F	-7.30638700	-3.15392400	1.28309600
F	-8.05551900	-2.37562100	-0.59170700
F	7.30455600	-3.15656000	-1.28270400
F	-6.65558700	-4.03600200	-0.58242100

9Y

C	4.86782900	-0.00030500	0.17655700
C	3.61666500	0.00003800	-0.06824900
C	2.21045500	0.00007100	-0.04849900
C	0.09756200	1.22033400	-0.04739000
H	-0.45931200	2.15877300	-0.05482800
C	-0.59666400	0.00018200	-0.04541000
C	1.48541200	1.22476800	-0.04660100
H	2.04041700	2.16443800	-0.04770400
C	1.48532100	-1.22458600	-0.04660800
H	2.04025600	-2.16429700	-0.04773300
C	0.09749100	-1.22003900	-0.04742200
H	-0.45944700	-2.15844900	-0.05492400
C	-2.10808200	0.00001100	0.01515900
F	-2.62687500	1.08643700	-0.57880600
F	-2.53515100	-0.00251000	1.29018400
F	-2.62662400	-1.08429400	-0.58293800

ICCTMS

C	0.80694900	0.00002300	-0.00001300
Si	2.65925200	0.00000200	-0.00000100
C	3.25250600	1.78974700	-0.01647700
H	2.88947400	2.33449800	0.86985700
H	4.35472200	1.83153500	-0.01688300
H	2.88944300	2.31809600	-0.91267200
C	3.25251800	-0.90915900	-1.54171200
H	4.35473400	-0.93045200	-1.57765000
H	2.88941100	-1.94944500	-1.55119500
H	2.88954900	-0.41393100	-2.45666300
C	3.25248200	-0.88060900	1.55820800
H	2.88944400	-1.92056900	1.58680600
H	4.35469800	-0.90116300	1.59459500
H	2.88942700	-0.36865500	2.46387100
C	-0.41649800	0.00004400	-0.00001400
I	-2.42487100	-0.00000400	0.00000000

Int-1-TMS

Bi	-0.80388000	0.08873500	1.24767500
C	-0.00807400	1.54118400	-0.23673600
Si	1.60890300	3.64364700	-1.79534500
C	0.60776000	2.39498000	-0.86806000
C	2.97699100	4.25853200	-0.65184900
H	2.54944100	4.77861200	0.22082200
H	3.63700900	4.96635600	-1.18114200
H	3.59024100	3.42599200	-0.27229800

C	2.35187600	2.80718000	-3.31708000
H	3.02574400	1.98344600	-3.03241200
H	2.93357900	3.53294100	-3.90987200
H	1.56229100	2.38975600	-3.96236100
C	0.48569800	5.06504900	-2.31853000
H	-0.32513800	4.70611300	-2.97264800
H	1.05778800	5.83137600	-2.86810800
H	0.02520400	5.54531600	-1.44019700
C	-2.92889800	0.30373100	0.58718400
Si	-5.91278700	0.62686100	-0.08614300
C	-4.12522000	0.43492500	0.34378400
C	-6.90056700	0.85731000	1.50619900
H	-6.78214100	-0.01184200	2.17332900
H	-7.97468100	0.97479700	1.28432800
H	-6.56424200	1.75315100	2.05287900
C	-6.10414700	2.13935000	-1.19779600
H	-5.75482500	3.05060400	-0.68607000
H	-7.16066600	2.28616300	-1.47878200
H	-5.51498900	2.02339000	-2.12168800
C	-6.48321400	-0.92930400	-0.98827400
H	-5.89812000	-1.08484000	-1.90894200
H	-7.54802300	-0.85165700	-1.26557700
H	-6.35777700	-1.82088800	-0.35293500
C	-0.44535800	-1.72404000	-0.00000400
Si	0.38949100	-4.29242300	-1.47061800
C	-0.13781800	-2.76694400	-0.56990600
C	0.77432300	-5.63299400	-0.19906500
H	1.57649000	-5.30799900	0.48327600
H	1.10346100	-6.56069900	-0.69663400
H	-0.11408800	-5.86709700	0.40935900
C	-0.99958200	-4.84260000	-2.61917800
H	-1.91615200	-5.06417500	-2.04916700
H	-0.71100700	-5.75126700	-3.17373500
H	-1.23847600	-4.05395800	-3.35060200
C	1.94380100	-3.87379400	-2.45824500
H	1.74973100	-3.05540600	-3.16993300
H	2.29161300	-4.75166000	-3.02804000
H	2.75790700	-3.55388600	-1.78902100
C	4.63290100	0.18086200	0.94275900
C	3.33384400	-0.64314500	0.57987900
B	2.78782600	1.26466400	1.71737400
O	4.08459700	1.46329800	1.35624200
O	2.32449700	0.01389600	1.41114800
C	5.59013100	0.41605800	-0.21965400
H	6.45453600	1.00122300	0.12763600

H	5.95894500	-0.54154600	-0.61839100
H	5.10949900	0.97421900	-1.03292500
C	2.91037700	-0.47189100	-0.87927100
H	3.61198200	-0.98296500	-1.55445600
H	1.91193000	-0.90244000	-1.02367200
H	2.85653500	0.58885600	-1.15294100
C	3.37126100	-2.11981800	0.95340300
H	2.41215900	-2.58304100	0.68162700
H	4.17627700	-2.63533400	0.40689800
H	3.52674200	-2.26494200	2.03016000
C	5.39007300	-0.38177200	2.14893100
H	5.89436300	-1.32781500	1.90291200
H	6.14871000	0.34860400	2.46597500
H	4.71197500	-0.55630700	2.99713600
H	2.12083000	2.09260000	2.27176100

Int-2-TMS

C	-0.70571700	-1.97584600	-0.38924900
C	-1.92539900	-1.47540500	-0.27549700
H	-0.54689100	-3.04733300	-0.60547900
C	1.79115700	0.84211200	-0.04917900
C	2.71791600	-0.43142100	0.08594500
C	1.99494100	1.90429500	1.02507100
H	3.02514600	2.29226500	0.99839900
H	1.30669700	2.74460100	0.84915700
H	1.79217400	1.50841200	2.02836400
C	1.84412600	1.47910200	-1.44186500
H	1.03588800	2.22057200	-1.52575600
H	2.80296600	1.98764300	-1.62065500
H	1.69398500	0.72461900	-2.22795500
C	3.09519700	-0.74741700	1.53688100
H	3.54772000	-1.74910300	1.57492800
H	3.81787200	-0.02077900	1.93673300
H	2.20615400	-0.75258700	2.18449800
C	3.96277800	-0.41760300	-0.79361900
H	4.60571300	0.44071300	-0.54337400
H	4.54074500	-1.33952300	-0.63102500
H	3.70397600	-0.36726100	-1.85895600
O	0.46996600	0.27125100	0.10297200
O	1.84118000	-1.49578800	-0.35987000
B	0.54987100	-1.06535400	-0.21552800
Si	-2.97335100	0.03063400	0.03655500
C	-2.54193700	0.70409000	1.74768600
H	-1.47005300	0.95370600	1.77639200
H	-3.12614600	1.61246800	1.97243200

H	-2.74853400	-0.04007100	2.53375900
C	-4.79751400	-0.44901600	-0.04314000
H	-5.43874800	0.43021600	0.13759100
H	-5.05647400	-0.86236000	-1.03156300
H	-5.04089900	-1.21012200	0.71603700
C	-2.57127900	1.30981400	-1.29490900
H	-2.79630500	0.92396800	-2.30220600
H	-3.15336100	2.23436500	-1.14249600
H	-1.49884600	1.55354500	-1.24653600

Bpin(CCTMS)

C	-0.75577100	-0.00050300	-0.00412100
C	-1.98073900	-0.00037200	-0.00462500
C	2.90726900	0.78874900	0.05084400
C	2.90763800	-0.78858000	-0.05121300
C	3.80973700	1.36389400	1.13643100
H	4.85932600	1.08314600	0.95710400
H	3.74095900	2.46188400	1.13105900
H	3.51514100	1.01398100	2.13396600
C	3.18135600	1.47917400	-1.28914700
H	2.95412200	2.55050100	-1.18839000
H	4.23254700	1.37033500	-1.59439700
H	2.54005000	1.07039000	-2.08375000
C	3.17898500	-1.47895800	1.28936200
H	2.95236400	-2.55036400	1.18805300
H	4.22945700	-1.36976900	1.59694900
H	2.53576900	-1.07043700	2.08255500
C	3.81266700	-1.36339900	-1.13483700
H	4.86177300	-1.08234200	-0.95318200
H	3.74420200	-2.46140800	-1.12968000
H	3.52016000	-1.01352100	-2.13299900
O	1.52700900	1.07266700	0.39201300
O	1.52822200	-1.07293700	-0.39541000
B	0.77477800	-0.00029900	-0.00242200
Si	-3.83306700	0.00001400	-0.00087900
C	-4.41670900	-0.36699900	1.75506600
H	-4.04915700	0.39557200	2.46033300
H	-5.51868200	-0.37548400	1.80208700
H	-4.05154500	-1.34939500	2.09505500
C	-4.43297200	1.70006900	-0.55519900
H	-4.07495100	1.93301900	-1.57094400
H	-5.53527700	1.73891400	-0.56312500
H	-4.06634900	2.48735000	0.12288600
C	-4.43686600	-1.33297100	-1.19062900
H	-4.07848000	-1.13955900	-2.21450600

H	-4.07253500	-2.32716500	-0.88585600
H	-5.53924700	-1.36281700	-1.21321800

HCCTMS

C	1.54825800	0.00016700	0.00011700
Si	-0.30515300	-0.00001600	-0.00000400
C	-0.90078800	-1.78713200	-0.08685700
H	-0.53847300	-2.36694300	0.77729700
H	-2.00302300	-1.82784800	-0.08896100
H	-0.53827400	-2.28047700	-1.00305000
C	-0.90103300	0.96865500	-1.50423500
H	-2.00327700	0.99062000	-1.53833000
H	-0.53872200	2.00884400	-1.47341000
H	-0.53872900	0.51026200	-2.43848700
C	-0.90121400	0.81827800	1.59097200
H	-0.53903100	1.85660300	1.66111200
H	-2.00346200	0.83670100	1.62696200
H	-0.53890300	0.27155500	2.47643200
C	2.76680900	0.00025700	0.00016600
H	3.84185100	-0.00044900	-0.00048100

Int-1-Ph

Bi	0.81961900	-0.07938300	-1.41749100
C	2.91985500	0.04217200	-0.68193500
C	0.05245600	1.46309500	-0.01458700
C	4.10786100	0.09915300	-0.39275700
C	-0.22964200	-2.62697300	0.64108100
C	5.48910000	0.16048500	-0.02406400
C	-0.85104900	-3.62780100	1.45175300
C	0.26120400	-1.74160700	-0.04629100
C	-0.44895800	2.32717500	0.69227300
C	8.20500200	0.27766200	0.70797000
H	9.25933500	0.32339200	0.99232500
C	6.42493300	0.82970700	-0.84113900
H	6.08246200	1.30403000	-1.76325900
C	-2.34642200	3.77324700	1.33565800
H	-2.93400500	3.34787400	0.52064500
C	7.28533800	-0.38901200	1.52537400
H	7.62033000	-0.86536500	2.45038000
C	-0.42919100	-4.97285300	1.40348700
H	0.39636100	-5.24828500	0.74394700
C	-1.02289800	3.32671700	1.54103500
C	7.76968300	0.88601400	-0.47455500

H	8.48389400	1.40853800	-1.11615000
C	-0.26934400	3.87639600	2.60003600
H	0.75437500	3.53124300	2.75925500
C	-1.05976800	-5.93758000	2.18964500
H	-0.72292700	-6.97640000	2.14428100
C	5.93872300	-0.44917100	1.16685500
H	5.21767100	-0.96722300	1.80251700
C	-0.82749900	4.84717500	3.43163300
H	-0.23410100	5.26584800	4.24855000
C	-2.89432000	4.74411000	2.17520700
H	-3.91998200	5.08322900	2.00790300
C	-1.91788200	-3.27756700	2.30779700
H	-2.24322400	-2.23607300	2.35106100
C	-2.14062000	5.28407000	3.22317900
H	-2.57520300	6.04483200	3.87676000
C	-2.11820600	-5.58115600	3.03278000
H	-2.60952800	-6.34012100	3.64679100
C	-2.54383300	-4.24913800	3.08856200
H	-3.36801000	-3.96493000	3.74790100
C	-3.58902200	-0.43849900	-1.79772700
C	-4.16123900	0.50930400	-0.67688400
C	-3.21171700	-1.83355300	-1.32409500
H	-4.09814600	-2.35393800	-0.92957200
H	-2.81817400	-2.42178700	-2.16662600
H	-2.44180000	-1.80936600	-0.54615300
C	-4.48885100	-0.52164300	-3.03482600
H	-3.92969800	-1.00857600	-3.84707300
H	-5.39569000	-1.11049300	-2.83351500
H	-4.78906000	0.47869400	-3.38019100
C	-3.56343800	0.23085000	0.70559800
H	-3.83897700	1.04838100	1.38730600
H	-3.94780100	-0.71313000	1.11925400
H	-2.46621800	0.18224200	0.66786200
C	-5.68177900	0.56642400	-0.60014300
H	-6.09453400	-0.43134400	-0.38431300
H	-5.98550300	1.24587100	0.21007000
H	-6.12230200	0.93750700	-1.53442100
O	-2.38660500	0.28161000	-2.21246300
O	-3.66977600	1.81209600	-1.10678100
B	-2.58410500	1.60336500	-1.90320800
H	-1.88721700	2.48449300	-2.31932000

Int-2-Ph

C	0.33847100	-1.74413900	0.55801300
C	1.55420900	-1.27906900	0.40056200

H	0.18623700	-2.78354900	0.89643700
C	-2.21801600	0.95903100	-0.10304300
C	-3.11310800	-0.34407500	-0.05979900
C	-2.46358900	1.86881300	-1.30136600
H	-3.50411200	2.22930600	-1.30941200
H	-1.79662800	2.74194100	-1.24475800
H	-2.26017300	1.35270700	-2.24827800
C	-2.27131700	1.76847700	1.19703500
H	-1.47259800	2.52424400	1.17532500
H	-3.23701000	2.28110600	1.31868900
H	-2.10291600	1.12339900	2.07194700
C	-3.50440400	-0.85105500	-1.45168500
H	-3.93186800	-1.85978400	-1.35420100
H	-4.25123700	-0.19816800	-1.92715600
H	-2.62559700	-0.91736900	-2.10978800
C	-4.34451700	-0.24996100	0.83377000
H	-5.01394200	0.55347200	0.48876300
H	-4.90001000	-1.19909500	0.80003600
H	-4.07121000	-0.05788400	1.87915100
O	-0.88249700	0.40866900	-0.19957100
O	-2.20072200	-1.32168000	0.50020100
B	-0.92573700	-0.87185100	0.28161900
C	2.72743400	-0.59959000	0.17481300
C	3.37444400	0.13573900	1.22617900
C	3.35972200	-0.61586100	-1.11537100
C	4.56478100	0.80527000	0.98767300
H	2.90990200	0.16077100	2.21403200
C	4.55025400	0.06299600	-1.32453400
H	2.88343700	-1.16807200	-1.92803200
C	5.16660400	0.77820500	-0.28269000
H	5.03858600	1.36200700	1.80080500
H	5.01262300	0.04098500	-2.31516200
H	6.10513700	1.30864000	-0.45886400

Bpin(CCPh)

C	0.54396700	0.00001600	-0.00005500
C	1.76527900	0.00001300	-0.00003500
C	-3.11553300	-0.78447400	-0.09547100
C	-3.11554900	0.78446700	0.09548900
C	-4.02110100	-1.54957400	0.86289600
H	-5.07015300	-1.23964900	0.73606600
H	-3.95280100	-2.62775000	0.65489200
H	-3.72881200	-1.39028000	1.90861300
C	-3.38687600	-1.21489000	-1.54074900
H	-3.15958500	-2.28640500	-1.63956400

H	-4.43745800	-1.05133900	-1.82289300
H	-2.74392400	-0.66617600	-2.24452600
C	-3.38683100	1.21488300	1.54077700
H	-3.15960100	2.28641300	1.63956500
H	-4.43738700	1.05127000	1.82298300
H	-2.74380400	0.66621700	2.24452300
C	-4.02117700	1.54954300	-0.86284100
H	-5.07022000	1.23961700	-0.73594500
H	-3.95287100	2.62772400	-0.65486500
H	-3.72894700	1.39022700	-1.90857200
O	-1.73670200	-1.12703000	0.19074100
O	-1.73673200	1.12705600	-0.19078600
B	-0.98239200	0.00002200	-0.00004000
C	3.19362600	0.00000600	-0.00001200
C	3.91079300	1.20347900	-0.17337700
C	3.91077400	-1.20347500	0.17336400
C	5.30536900	1.19876500	-0.17296600
H	3.35894100	2.13607900	-0.30765700
C	5.30535200	-1.19877600	0.17299500
H	3.35891200	-2.13607200	0.30762300
C	6.00650000	-0.00001000	0.00002600
H	5.85007500	2.13657400	-0.30829700
H	5.85004100	-2.13659300	0.30833800
H	7.09946800	-0.00001500	0.00004100

HCCPh

C	-2.02833700	-0.00005500	0.00002800
C	-0.59562300	-0.00005500	0.00001200
C	0.12074400	1.21500400	0.00000500
C	0.12081800	-1.21504700	0.00000500
C	1.51575700	1.21048600	-0.00000400
H	-0.42975600	2.15805400	0.00000900
C	1.51584100	-1.21042700	-0.00000500
H	-0.42959900	-2.15814800	0.00000900
C	2.21761500	0.00004900	-0.00000900
H	2.05958300	2.15851600	-0.00000800
H	2.05973000	-2.15842100	-0.00000800
H	3.31050500	0.00008400	-0.00001700
C	-3.24251700	0.00000600	-0.00001600
H	-4.31625000	0.00014200	-0.00008100

Bpin_radical

C	0.00000000	0.79054300	-0.14896700
C	0.00000000	-0.79054300	-0.14896700

C	1.06030600	1.43427600	-1.03256200
H	0.91140800	1.14845100	-2.08578100
H	0.98735100	2.52945400	-0.95967000
H	2.07347000	1.14371000	-0.72750600
C	-1.37480700	1.39915400	-0.43368800
H	-1.34050700	2.47458000	-0.20675900
H	-1.66192500	1.27206500	-1.48817900
H	-2.14988600	0.94408500	0.19967700
C	1.37480700	-1.39915400	-0.43368800
H	1.34050700	-2.47458000	-0.20675900
H	1.66192500	-1.27206500	-1.48817900
H	2.14988600	-0.94408500	0.19967700
C	-1.06030600	-1.43427600	-1.03256200
H	-0.91140800	-1.14845100	-2.08578100
H	-0.98735100	-2.52945400	-0.95967000
H	-2.07347000	-1.14371000	-0.72750600
O	0.31204600	1.10033300	1.25189000
O	-0.31204600	-1.10033300	1.25189000
B	0.00000000	0.00000000	1.97776000

HB(OCMe2CMeCH2O)_radical

C	0.80364000	-0.24283300	0.12830100
C	-0.78152700	-0.16160700	-0.05334900
C	1.24202400	-1.04321200	1.30332700
H	1.41814800	-2.11616800	1.20530900
H	1.29994100	-0.57647200	2.28714600
C	1.53280900	-0.70088500	-1.14236600
H	2.61186800	-0.53822400	-1.00909700
H	1.35906100	-1.76791900	-1.34440500
H	1.20169800	-0.11749800	-2.01408000
C	-1.51960400	-0.28131300	1.28262900
H	-2.57371100	-0.00113800	1.13750800
H	-1.47414100	-1.30899800	1.67278800
H	-1.08510800	0.39495700	2.03338900
C	-1.36026500	-1.12715600	-1.07824700
H	-1.14745000	-2.16884200	-0.79247000
H	-2.45201800	-0.99925400	-1.12879300
H	-0.95373200	-0.94396600	-2.08121400
O	1.14637300	1.15378200	0.33471400
O	-0.96753900	1.19501900	-0.52352800
B	0.13201000	1.91704100	-0.16416700
H	0.20227100	3.10994900	-0.27649700
